# Supplementary material for: Nickel-catalyzed β-arylation and benzylation of 2′-hydroxychalcones to access warfarin analogues
Source: Commun Chem. 2025 Nov 27;8:382. doi: 10.1038/s42004-025-01767-w (PMC12660831; doi:10.1038/s42004-025-01767-w)
Supplement: Supplementary file 2 — Supplementary Information [file 42004_2025_1767_MOESM2_ESM.pdf]

Supporting Information

Nickel-catalyzed *β*-arylation and benzylation of 2′-hydroxychalcones to access warfarin analogues

Bo-Cheng Tang[1], Xiaochun Su[1,2], Jiang Liu[1,2], Xiao Yang[2,3] and Cong Ma[1,2]**

[1]State Key Laboratory of Chemical Biology and Drug Discovery, and Department of Applied Biology and Chemical Technology, The Hong Kong Polytechnic University, Kowloon, Hong Kong SAR, China. Email: cong.ma@polyu.edu.hk

[2]Marshall Research Centre for Medical Microbial Biotechnology, The Hong Kong Polytechnic University, Kowloon, Hong Kong SAR, China

[3]Department of Microbiology, The Chinese University of Hong Kong, Prince of Wales Hospital, Shatin, Hong Kong SAR, China. Email: xiaoyang@cuhk.edu.hk

Table of Contents

Supporting Information S1
Supplementary Note 1. General information S2
Supplementary Note 2. Optimization of the Reaction Conditions S2
Supplementary Method 1. General procedure for the synthesis of 1-57 (1 as an example). S4
Supplementary Note 3. Mechanistic study S4
Supplementary Note 4. Characterization data of compounds S6
Supplementary Note 5. Crystallographic data and molecular structures of 1, 8, 29, 56, 58 .... S28
Supplementary Note 6. Materials for Biological Studies S33
Supplementary Method 2. Analytical Methods for Biological Samples S33
Supplementary Method 3. Rapid Equilibrium Dialysis Assay S34
Supplementary Method 4. Plasma Stability Assay S35
Supplementary Method 5. Animal and Ethics Statement S35
Supplementary Method 6. Drug Preparation S35
Supplementary Method 7. *In vivo* Anticoagulation Assay S35
Supplementary Method 8. Statistical Analysis S36
Supplementary Note 7. Plasma Protein Binding Rates of Ten Tested Compounds S37
Supplementary Note 8. Plasma Stability of Ten Tested Compounds S38
Supplementary Note 9. NMR spectra of compounds S39

Supplementary Note 1. General information

All the materials and solvents were commercially available and used without further purification. TLC analysis was performed using pre-coated glass plates. Column chromatography was performed using silica gel (100–200 mesh). ^{1}H spectra were recorded in $CDCl_3$ and DMSO-d_6 on 600 MHz NMR spectrometers and resonances (δ) are given in parts per million relative to tetramethylsilane. Data are reported as follows: chemical shift, multiplicity (s = singlet, d = doublet, t = triplet, m = multiplet, q = quartet), coupling constants (Hz) and integration. ^{13}C spectra were recorded in $CDCl_3$ and DMSO-d_6 on 150 MHz NMR spectrometers and resonances (δ) are given in ppm. ^{19}F spectra were recorded in $CDCl_3$ and DMSO-d_6 on 564 MHz NMR using TMS as internal standard. High-resolution mass spectra (HRMS) were obtained by electrospray ionization (ESI) on a TOF mass analyzer. The X-ray crystal-structure determinations of **1, 8, 29 and 56** were obtained on a Bruker SMART APEX CCD system.

Supplementary Note 2. Optimization of the Reaction Conditions

Table S1. Optimization of Reaction Conditions with Different Temperatures, Ni Catalysts and Solvents

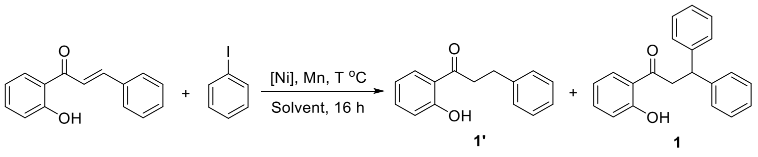

Entry	Temp.(°C)	[Ni]	Solvent	**1'** [%][a]	**1** [%][a]
1	110	$Ni(OAc)_2·4H_2O$	DMF	trace	18
2	20	$Ni(OAc)_2·4H_2O$	DMF	n.d.	<5
3	60	$Ni(OAc)_2·4H_2O$	DMF	n.d.	<5
4	110	$Ni(cod)_2$	DMF	26	n.d.
5	110	$Ni(acac)_2$	DMF	35	n.d.
6	110	$NiCl_6·6H_2O$	DMF	16	23
7	110	$Ni(DME)Cl_2$	DMF	10	27
8	110	$Ni(dppp)Cl_2$	DMF	<5	<5
9	110	$Ni(PCy_3)Cl_2$	DMF	14	21
10	110	$NiBr_2$	DMF	17	31
11	110	$Ni(DME)Br_2$	DMF	<5	48
12	110	$Ni(PPh_3)Br_2$	DMF	<5	38
**13**	**110**	**NiI_2**	**DMF**	**trace**	**56**
14	110	$Ni(OTf)_2$	DMF	trace	55
15	110	$Ni(TFA)_2$	DMF	trace	51
16	110	$Ni(OH)_2$	DMF	n.d.	n.d.
17	110	NiI_2	nBuOH	n.d.	n.d.
18	110	NiI_2	toluene	n.d.	n.d.
19	110	NiI_2	dioxane	n.d.	n.d.
20	110	NiI_2	NMP	n.d.	42
21	80	NiI_2	THF	n.d.	46
22	80	NiI_2	DME	n.d.	n.d.
23	80	NiI_2	ACN	n.d.	n.d.

Reaction conditions: **2'-hydroxychalcone** (0.5 mmol), **Iodobenzene** (1.5 mmol), [Ni] (10 mol%), Mn (1.5 mmol) solvent (4.0 ml), T °C, 16 h. [a]Yields of isolated products based on **2'-hydroxychalcone**.

Table S2. Optimization of Reaction Conditions with Different Additives and Ligands

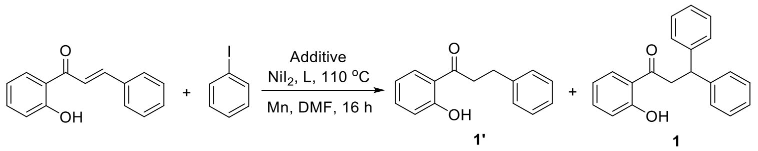

Entry	Mn (x eq)	Additive (1.5 eq)	ligand	**1'** [%][a]	**1** [%][a]
1	0	-	-	trace	n.d.
2	1.0	-	-	trace	33
3	1.5	-	-	trace	38
4	2.5	-	-	trace	53
5	3.0	-	-	trace	56
6	4.0	-	-	trace	45
7	3.0	H_2O	-	11	34
8	3.0	HOAc	-	16	n.d.
9	3.0	MeOH	-	trace	27
10	3.0	HCOOK	-	trace	n.d.
11	3.0	$HSiEt_3$	-	trace	49
12	3.0	$PhSiH_3$	-	61	21
13	3.0	Bu_4NI	-	trace	43
14	3.0	Me_4NOAc	-	trace	<10
15	3.0	LiCl	-	trace	n.d.
16	3.0	-	Dimethyl maleate	trace	64
17	3.0	-	PPh_3	trace	65
18	3.0	-	1,10-Phen	n.d.	trace
19	3.0	-	bpy	n.d.	trace
20	3.0	-	$P(O)Ph_3$	trace	58
21	3.0	-	L1	trace	57
22	3.0	-	L2	trace	60
23	3.0	-	L3	trace	56
24	3.0	-	L4	trace	66
25	3.0	-	L5	n.d.	69
26	3.0	-	L6	trace	67
**27**	**3.0**	**-**	**L7**	**n.d.**	**78**
28	3.0	-	L8	n.d.	76
29	3.0	-	L9	trace	75
30	3.0	-	L10	trace	72
31	3.0	-	L11	trace	61
32	3.0	-	L12	trace	72

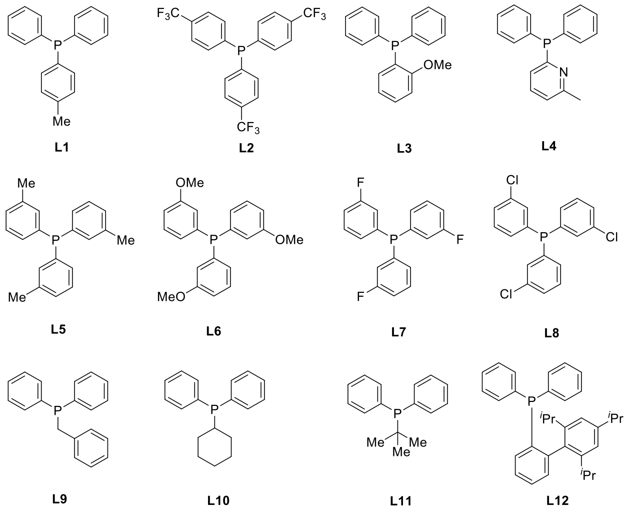

Reaction conditions: **2'-hydroxychalcone** (0.5 mmol), **Iodobenzene** (1.5 mmol), NiI_2 (10 mol%), ligand (22 mol%), Mn (x eq), DMF (4.0 ml), 110 °C, 16 h. [a]Yields of isolated products based on **2'-hydroxychalcone**.

Supplementary Method 1. General procedure for the synthesis of 1-57 (1 as an example).

A 25 ml Schlenk-type tube equipped with a magnetic stir bar was charged with the mixture of alkene (0.5 mmol), Mn (1.25 mmol, 3.0 eq), NiI_2 (10 mol%), $P(3\text{-}F\text{-}Ph)_3$ (22 mmol%). The tube was evacuated and backfilled with nitrogen (10 times) before the iodobenzene (1.5 mmol, 3.0 eq) in DMF (4 mL) was added to the tube using syringe. The mixture was stirred at 110 °C for 16 hours. After cooling to room temperature, the mixture was quenched with water, extracted with EtOAc, the combined organic layers were washed with brine, dried over anhydrous Na_2SO_4 and concentrated under reduced pressure. The crude product was purified by column chromatography on silica gel (eluent: nHex/EtOAc = 100/1, v/v) to afford the products **1**.

Supplementary Note 3. Mechanistic study

(a)

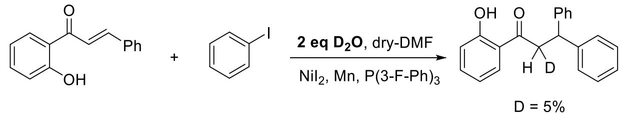

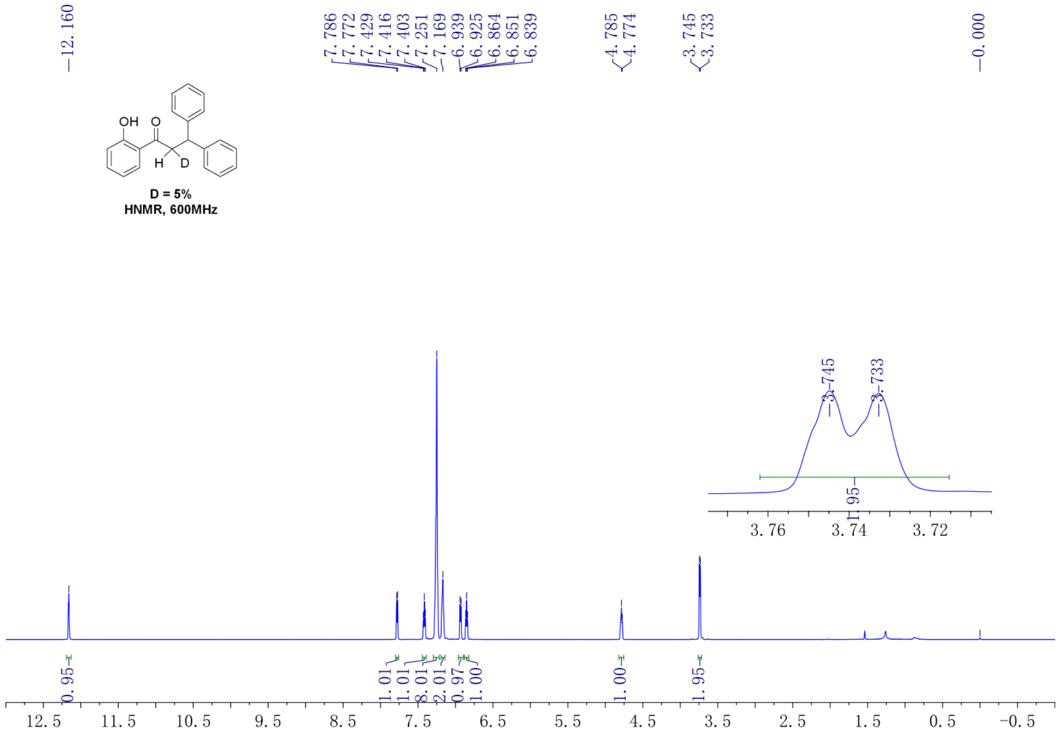

(b)

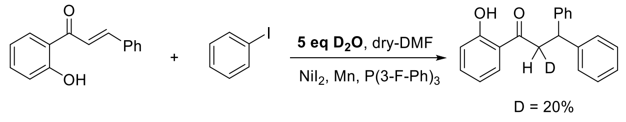

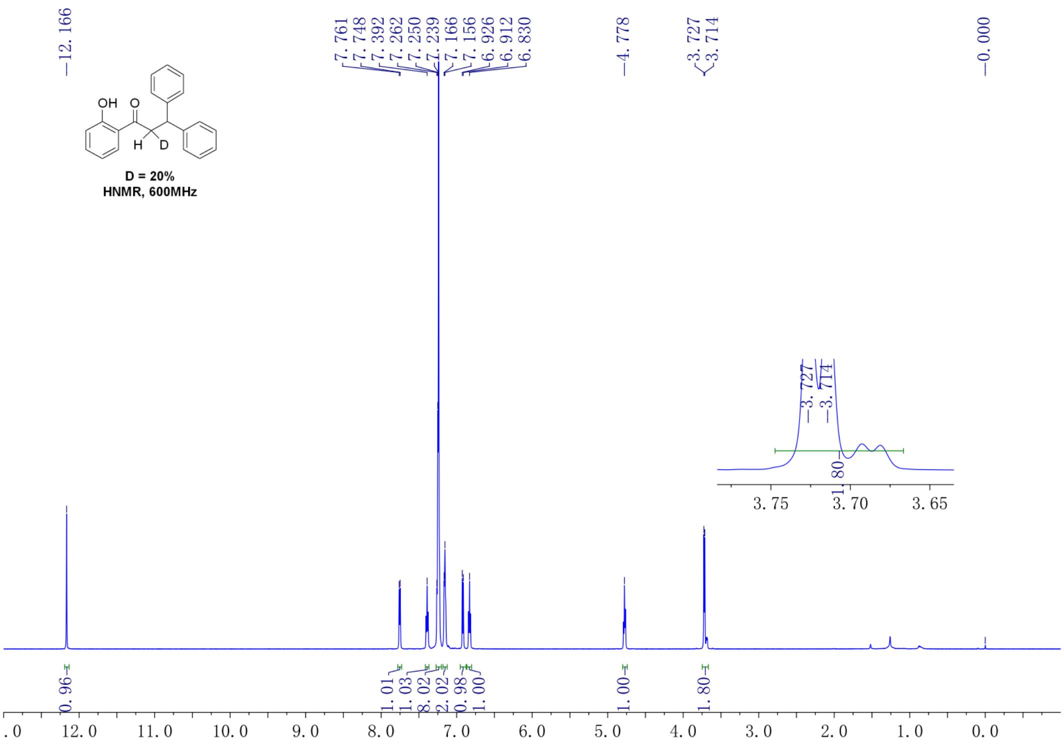
D = 20%
HNMR, 600MHz

(c)

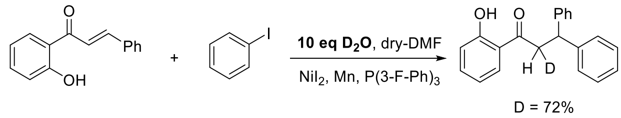
10 eq D2O, dry-DMF
NiI2, Mn, P(3-F-Ph)3
D = 72%

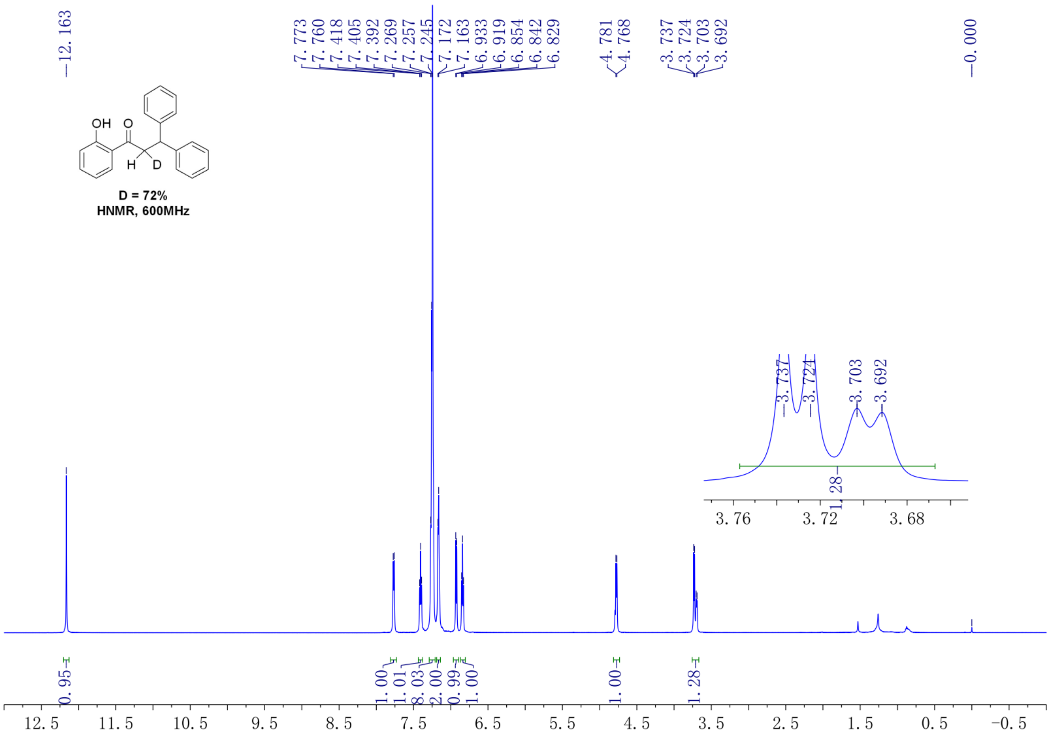
D = 72%
HNMR, 600MHz

Supplementary Note 4. Characterization data of compounds

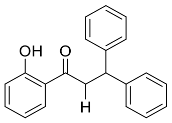

1-(2-hydroxyphenyl)-3,3-diphenylpropan-1-one (1):
Yield: 73%; 110.4 mg; white solid; m.p. 91-93 °C; TLC (nHex:EtOAc, 100:1 v/v): R_f = 0.3; ^{1}H NMR (600 MHz, $CDCl_3$) δ = 12.15 (s, 1H), 7.81 (dd, *J* = 8.1, 1.4 Hz, 1H), 7.47 -7.42 (m, 1H), 7.29-7.25 (m, 7H), 7.25 (s, 1H), 7.21-7.16 (m, 2H), 6.95 (dd, *J* = 8.4, 0.7 Hz, 1H), 6.90-6.85 (m, 1H), 4.80 (t, *J* = 7.3 Hz, 1H), 3.76 (d, *J* = 7.3 Hz, 2H). ^{13}C NMR (150 MHz, $CDCl_3$) δ = 204.0, 162.4, 143.7, 136.4, 129.7, 128.6, 127.7, 126.5, 119.4, 118.9, 118.6, 45.7, 44.1. HRMS (ESI) m/z calcd for : $C_{21}H_{17}O_2^-$ $(M-H)^-$ 301.1229, found 301.1228.

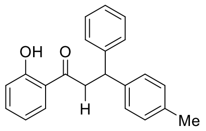

1-(2-hydroxyphenyl)-3-phenyl-3-(*p*-tolyl)propan-1-one (2):
Yield: 75%; 118.3 mg; yellow oil; TLC (nHex:EtOAc, 100:1 v/v): R_f = 0.3; ^{1}H NMR (600 MHz, CDCl3) δ = 12.07 (s, 1H), 7.64 (d, J = 8.0 Hz, 1H), 7.27 (t, J = 7.7 Hz, 1H), 7.14 – 7.10 (m, 4H), 7.03 (dd, J = 12.4, 7.1 Hz, 3H), 6.95 (d, J = 7.8 Hz, 2H), 6.80 (d, J = 8.4 Hz, 1H), 6.71 (t, J = 7.6 Hz, 1H), 4.63 (t, J = 7.3 Hz, 1H), 3.58 (d, J = 7.2 Hz, 2H), 2.15 (s, 3H). ^{13}C NMR (150 MHz, $CDCl_3$) δ = 204.0, 162.4, 143.9, 140.6, 136.3, 135.9, 129.6, 129.2, 128.5, 127.6, 127.5, 126.4, 119.3, 118.8, 118.4, 45.3, 44.0, 20.9. DEPT-135 (150 MHz, $CDCl_3$) δ = 136.5, 129.9, 129.5, 128.8, 127.9, 127.8, 126.6, 119.1, 118.7, 45.5, 44.3 (neg.), 21.2. HRMS (ESI) m/z calcd for : $C_{22}H_{19}O_2^-$ $(M-H)^-$ 315.1385, found 315.1385.

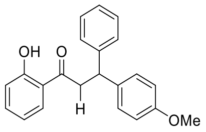

1-(2-hydroxyphenyl)-3-(4-methoxyphenyl)-3-phenylpropan-1-one (3):
Yield: 64%; 106.1 mg; yellow oil; TLC (nHex:EtOAc, 30:1 v/v): R_f = 0.3; ^{1}H NMR (600 MHz, $CDCl_3$) δ = 12.19 (s, 1H), 7.75 (d, J = 8.0 Hz, 1H), 7.38 (t, J = 7.7 Hz, 1H), 7.23 (q, J = 8.0 Hz, 4H), 7.14 (t, J = 6.6 Hz, 3H), 6.91 (d, J = 8.3 Hz, 1H), 6.82 (t, J = 7.6 Hz, 1H), 6.78 (d, J = 8.5 Hz, 2H), 4.72 (t, J = 7.3 Hz, 1H), 3.68 (d, J = 6.7 Hz, 5H). ^{13}C NMR (150 MHz, $CDCl_3$) δ = 204.1, 162.3, 158.0, 144.0, 136.3, 135.7, 129.6, 128.6, 128.5, 127.5, 126.3, 119.3, 118.8, 118.4, 113.9, 55.0, 44.9, 44.1. DEPT-135 (150 MHz, $CDCl_3$) δ = 136.5, 129.9, 128.8, 128.7, 127.8, 126.6, 119.1, 118.7, 114.1, 55.3, 45.2, 44.4 (neg.). HRMS (ESI) m/z calcd for : $C_{22}H_{19}O_3^-$ $(M-H)^-$ 331.1334, found 331.1344.

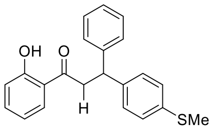

1-(2-hydroxyphenyl)-3-(4-(methylthio)phenyl)-3-phenylpropan-1-one (4):

Yield: 65%; 113.0 mg; yellow solid; m.p. 86-88 ºC; TLC (nHex:EtOAc, 50:1 v/v): R_f = 0.3; ^{1}H NMR (600 MHz, $CDCl_3$) δ = 12.14 (s, 1H), 7.79 (d, *J* = 8.0 Hz, 1H), 7.44 (t, *J* = 7.7 Hz, 1H), 7.27 (t, *J* = 7.3 Hz, 2H), 7.23 (d, *J* = 7.8 Hz, 2H), 7.19 – 7.15 (m, 5H), 6.94 (d, *J* = 8.3 Hz, 1H), 6.87 (t, *J* = 7.5 Hz, 1H), 4.75 (t, *J* = 7.2 Hz, 1H), 3.72 (d, *J* = 7.2 Hz, 2H), 2.41 (s, 3H). ^{13}C NMR (150 MHz, $CDCl_3$) δ = 203.8, 162.4, 143.6, 140.6, 136.4, 136.3, 129.6, 128.6, 128.2, 127.6, 126.9, 126.5, 119.3, 118.9, 118.6, 45.2, 44.0, 15.9. DEPT-135 (150 MHz, $CDCl_3$) δ = 136.5, 129.8, 128.8, 128.3, 127.7, 127.0, 126.7, 119.0, 118.7, 45.3, 44.1 (neg.), 16.0. HRMS (ESI) m/z calcd for : $C_{22}H_{19}O_2S^-$ $(M-H)^-$ 347.1106, found 347.1113.

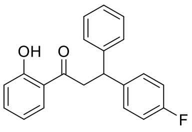

3-(4-fluorophenyl)-1-(2-hydroxyphenyl)-3-phenylpropan-1-one (5):
Yield: 74%; 118.6 mg; yellow oil; TLC (nHex:EtOAc, 100:1 v/v): R_f = 0.3; ^{1}H NMR (600 MHz, $CDCl_3$) δ = 12.04 (s, 1H), 7.67 (d, *J* = 8.0 Hz, 1H), 7.31 (t, *J* = 7.7 Hz, 1H), 7.17 (t, *J* = 7.4 Hz, 2H), 7.13 – 7.06 (m, 5H), 6.83 (t, *J* = 7.5 Hz, 3H), 6.75 (t, *J* = 7.6 Hz, 1H), 4.67 (t, *J* = 7.3 Hz, 1H), 3.61 (d, *J* = 7.3 Hz, 2H). ^{13}C NMR (150 MHz, $CDCl_3$) δ = 203.7, 162.4, 162.2, 160.6, 143.5, 139.4, 136.4, 129.6, 129.1, 128.7, 127.6, 126.6, 119.3, 118.9, 118.5, 115.4, 44.9, 44.1. DEPT-135 (150 MHz, $CDCl_3$) δ = 136.6, 129.8, 129.3, 128.8, 127.7, 126.8, 119.1, 118.7, 115.5, 45.1, 44.2 (neg.). ^{19}F NMR (564 MHz, $CDCl_3$) δ = -116.24. HRMS (ESI) m/z calcd for : $C_{21}H_{16}FO_2^-$ $(M-H)^-$ 319.1134, found 319.1142.

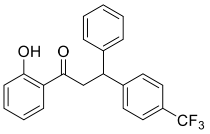

1-(2-hydroxyphenyl)-3-phenyl-3-(4-(trifluoromethyl)phenyl)propan-1-one (6):
Yield: 54%; 100.2 mg; yellow oil; TLC (nHex:EtOAc, 100:1 v/v): R_f = 0.3; ^{1}H NMR (600 MHz, $CDCl_3$) δ =12.09 (s, 1H), 7.79 (d, *J* = 8.0 Hz, 1H), 7.52 (d, *J* = 8.0 Hz, 2H), 7.43 (t, *J* = 7.7 Hz, 1H), 7.37 (d, *J* = 7.9 Hz, 2H), 7.29 (t, *J* = 7.4 Hz, 2H), 7.24 (d, *J* = 7.5 Hz, 2H), 7.21 (d, *J* = 7.3 Hz, 1H), 6.94 (d, *J* = 8.4 Hz, 1H), 6.87 (t, *J* = 7.6 Hz, 1H), 4.85 (t, *J* = 7.2 Hz, 1H), 3.76 (d, *J* = 7.3 Hz, 2H). ^{13}C NMR (150 MHz, $CDCl_3$) δ = 203.3, 162.4, 147.8, 142.8, 136.6, 129.6, 128.8, 128.1, 127.7, 126.9, 125.5, 125.0, 123.2, 119.3, 119.0, 118.6, 45.4, 43.7. DEPT-135 (150 MHz, $CDCl_3$) δ = 136.6, 129.6, 128.8, 128.1, 127.7, 126.9, 125.5, 119.0, 118.6, 45.4, 43.7 (neg.). ^{19}F NMR (564 MHz, $CDCl_3$) δ = -62.29. HRMS (ESI) m/z calcd for : $C_{22}H_{16}F_3O_2^-$ $(M-H)^-$ 369.1102, found 369.1120.

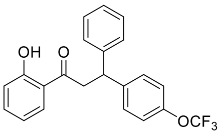

1-(2-hydroxyphenyl)-3-phenyl-3-(4-(trifluoromethoxy)phenyl)propan-1-one (7):
Yield: 60%; 115.9 mg; yellow oil; TLC (nHex:EtOAc, 100:1 v/v): R_f = 0.3; ^{1}H NMR (600 MHz, $CDCl_3$) δ =12.12 (s, 1H), 7.76 (d, *J* = 8.0 Hz, 1H), 7.41 (t, *J* = 7.7 Hz, 1H), 7.25 (dt, *J* = 13.7, 7.5 Hz, 6H), 7.18 (t, *J* = 7.3 Hz, 1H), 7.10 (d, *J* = 8.2 Hz, 2H), 6.93 (d, *J* = 8.4 Hz, 1H), 6.85 (t, *J* = 7.6 Hz, 1H), 4.80 (t, *J* = 7.2 Hz, 1H), 3.72 (d, *J* = 5.5 Hz, 2H). ^{13}C NMR (150 MHz,

CDCl3) δ = 203.5, 162.4, 147.7, 143.1, 142.5, 136.5, 129.6, 129.0, 128.7, 127.6, 126.8, 121.0, 119.3, 118.9, 118.6, 45.0, 44.0. DEPT-135 (150 MHz, $CDCl_3$) δ = 136.7, 129.7, 129.2, 128.9, 127.8, 126.9, 121.2, 119.1, 118.7, 45.2, 44.1 (neg.). ^{19}F NMR (564 MHz, $CDCl_3$) δ = -57.72. HRMS (ESI) m/z calcd for : $C_{22}H_{16}F_3O_3^-$ $(M-H)^-$ 385.1052, found 385.1076.

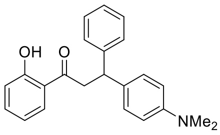

3-(4-(dimethylamino)phenyl)-1-(2-hydroxyphenyl)-3-phenylpropan-1-one (8):
Yield: 62%; 107.1 mg; yellow solid; m.p. 119-120 ºC; TLC (nHex:EtOAc, 30:1 v/v): R_f = 0.3; ^{1}H NMR (600 MHz, $CDCl_3$) δ =12.22 (s, 1H), 7.79 (d, *J* = 8.0 Hz, 1H), 7.42 (t, *J* = 7.7 Hz, 1H), 7.24 (s, 4H), 7.15 (s, 1H), 7.10 (d, *J* = 7.8 Hz, 2H), 6.93 (d, *J* = 8.3 Hz, 1H), 6.85 (t, *J* = 7.5 Hz, 1H), 6.64 (d, *J* = 7.7 Hz, 2H), 4.69 (t, *J* = 7.2 Hz, 1H), 3.70 (d, *J* = 7.2 Hz, 2H), 2.87 (s, 6H). ^{13}C NMR (150 MHz, $CDCl_3$) δ = 204.4, 162.4, 149.2, 144.5, 136.2, 131.5, 129.7, 128.5, 128.2, 127.6, 126.2, 119.4, 118.8, 118.5, 112.7, 44.9, 44.4, 40.5. DEPT-135 (150 MHz, $CDCl_3$) δ = 136.4, 129.9, 128.6, 128.4, 127.7, 126.4, 119.0, 118.6, 112.9, 45.0, 44.5 (neg.), 40.7. HRMS (ESI) m/z calcd for : $C_{23}H_{22}NO_2^-$ $(M-H)^-$ 344.1651, found 344.1653.

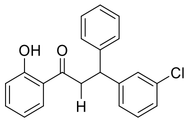

3-(3-chlorophenyl)-1-(2-hydroxyphenyl)-3-phenylpropan-1-one (9):
Yield: 67%; 112.5 mg; yellow oil; TLC (nHex:EtOAc, 100:1 v/v): R_f = 0.3; ^{1}H NMR (600 MHz, $CDCl_3$) δ = 12.10 (s, 1H), 7.77 (dd, J = 8.0, 1.1 Hz, 1H), 7.42 (s, 1H), 7.27 (t, J = 7.5 Hz, 2H), 7.25 – 7.21 (m, 3H), 7.18 (dd, J = 12.0, 7.5 Hz, 2H), 7.14 (dd, J = 8.9, 4.4 Hz, 2H), 6.94 (d, J = 8.3 Hz, 1H), 6.86 (s, 1H), 4.75 (s, 1H), 3.72 (dd, J = 8.7, 7.6 Hz, 2H). ^{13}C NMR (150 MHz, $CDCl_3$) δ = 203.4, 162.4, 145.8, 142.9, 136.5, 134.4, 129.8, 129.6, 128.7, 127.9, 127.6, 126.8, 126.7, 125.9, 119.3, 118.9, 118.6, 45.3, 43.7. DEPT-135 (150 MHz, $CDCl_3$) δ = 136.6, 130.0, 129.7, 128.9, 128.0, 127.8, 126.9, 126.8, 126.1, 119.1, 118.7, 45.4, 43.9 (neg.). HRMS (ESI) m/z calcd for : $C_{21}H_{16}ClO_2^-$ $(M-H)^-$ 335.0839, found 335.0842.

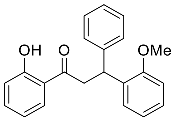

1-(2-hydroxyphenyl)-3-(2-methoxyphenyl)-3-phenylpropan-1-one (10):
Yield: 65%; 108.0 mg; yellow solid; m.p. 125-127 ºC; TLC (nHex:EtOAc, 70:1 v/v): R_f = 0.3; ^{1}H NMR (600 MHz, $CDCl_3$) δ = 12.21 (s, 1H), 7.84 (d, *J* = 7.5 Hz, 1H), 7.45 (s, 1H), 7.28 (d, *J* = 3.8 Hz, 4H), 7.19 (t, *J* = 7.0 Hz, 2H), 7.07 (d, *J* = 7.0 Hz, 1H), 6.95 (d, *J* = 8.3 Hz, 1H), 6.90 – 6.82 (m, 3H), 5.15 (s, 1H), 3.77 (s, 3H), 3.72 (d, *J* = 7.4 Hz, 2H). ^{13}C NMR (150 MHz, $CDCl_3$) δ = 204.6, 162.4, 156.7, 143.1, 136.2, 132.2, 129.8, 128.4, 128.1, 128.0, 127.6, 126.3, 120.6, 119.5, 118.8, 118.5, 110.7, 55.3, 43.2, 39.6. DEPT-135 (150 MHz, $CDCl_3$) δ = 136.2, 129.9, 128.4, 128.1, 128.0, 127.7, 126.3, 120.6, 118.9, 118.5, 110.8, 55.4, 43.2 (neg.), 39.7. HRMS (ESI) m/z calcd for : $C_{22}H_{19}O_3^-$ $(M-H)^-$ 331.1334, found 331.1340.

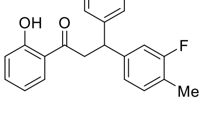

3-(3-fluoro-4-methylphenyl)-1-(2-hydroxyphenyl)-3-phenylpropan-1-one (11):
Yield: 71%; 118.8 mg; yellow oil; TLC (nHex:EtOAc, 100:1 v/v): R_f = 0.3; ^{1}H NMR (600 MHz, $CDCl_3$) δ = 12.04 (s, 1H), 7.71 (d, *J* = 8.0 Hz, 1H), 7.35 (d, *J* = 7.6 Hz, 1H), 7.19 (t, *J* = 7.3 Hz, 2H), 7.15 (d, *J* = 7.5 Hz, 2H), 7.10 (s, 1H), 6.98 (s, 1H), 6.85 (t, *J* = 8.0 Hz, 2H), 6.80 (d, *J* = 13.6 Hz, 2H), 4.66 (s, 1H), 3.63 (d, *J* = 7.1 Hz, 2H), 2.11 (s, 3H). ^{13}C NMR (150 MHz, $CDCl_3$) δ = 203.8, 162.5, 162.2, 160.6, 143.5, 143.4, 136.6, 131.6, 129.7, 128.8, 127.7, 126.8, 123.1, 123.0, 119.4, 118.9, 114.4, 45.2, 44.0, 14.2. DEPT-135 (150 MHz, $CDCl_3$) δ = 136.6, 131.6, 129.7, 128.8, 127.7, 126.8, 123.1, 119.0, 118.7, 114.4, 45.2, 44.0 (neg.), 14.2. ^{19}F NMR (564 MHz, $CDCl_3$) δ = -116.89. HRMS (ESI) m/z calcd for : $C_{22}H_{18}FO_2^-$ (M-H)$^-$ 333.1291, found 333.1299.

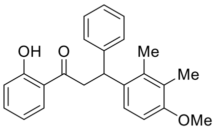

1-(2-hydroxyphenyl)-3-(4-methoxy-2,3-dimethylphenyl)-3-phenylpropan-1-one (12):
Yield: 70%; 126.3 mg; yellow oil; TLC (nHex:EtOAc, 70:1 v/v): R_f = 0.3; ^{1}H NMR (600 MHz, $CDCl_3$) δ = 12.20 (s, 1H), 7.80 (d, *J* = 7.9 Hz, 1H), 7.44 (dd, *J* = 11.4, 4.1 Hz, 1H), 7.24 (t, *J* = 7.4 Hz, 2H), 7.16 (dd, *J* = 15.9, 7.5 Hz, 3H), 7.02 (d, *J* = 8.5 Hz, 1H), 6.95 (d, *J* = 8.3 Hz, 1H), 6.87 (t, *J* = 7.5 Hz, 1H), 6.68 (d, *J* = 8.5 Hz, 1H), 4.99 (t, *J* = 7.3 Hz, 1H), 3.79 – 3.61 (m, 5H), 2.20 (s, 3H), 2.14 (s, 3H). ^{13}C NMR (150 MHz, $CDCl_3$) δ = 204.3, 162.4, 156.0, 144.1, 136.3, 136.2, 133.3, 129.7, 128.5, 127.9, 126.2, 125.8, 124.1, 119.5, 118.9, 118.5, 107.4, 55.4, 44.7, 41.8, 15.6, 12.2. DEPT-135 (150 MHz, $CDCl_3$) δ = 136.4, 129.8, 128.6, 128.0, 126.3, 124.2, 119.0, 118.6, 107.5, 55.5, 44.8 (neg.), 41.9, 15.7, 12.3. HRMS (ESI) m/z calcd for : $C_{24}H_{23}O_3^-$ (M-H)$^-$ 359.1647, found 359.1655.

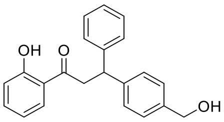

3-(4-(hydroxymethyl)phenyl)-1-(2-hydroxyphenyl)-3-phenylpropan-1-one (13):
Yield: 55%; 91.5 mg; yellow oil; TLC (nHex:EtOAc, 2:1 v/v): R_f = 0.3; ^{1}H NMR (600 MHz, $CDCl_3$) δ = 12.14 (s, 1H), 7.78 (d, *J* = 8.0 Hz, 1H), 7.42 (t, *J* = 7.7 Hz, 1H), 7.27 – 7.21 (m, 8H), 7.17 (d, *J* = 6.9 Hz, 1H), 6.92 (d, *J* = 8.4 Hz, 1H), 6.86 (t, *J* = 7.6 Hz, 1H), 4.77 (t, *J* = 7.3 Hz, 1H), 4.56 (s, 2H), 3.77 – 3.68 (m, 2H). ^{13}C NMR (150 MHz, $CDCl_3$) δ = 203.9, 162.3, 143.6, 143.0, 139.1, 136.4, 129.6, 128.6, 127.8, 127.6, 127.3, 126.5, 119.3, 118.9, 118.5, 64.8, 45.4, 43.9. DEPT-135 (150 MHz, $CDCl_3$) δ = 136.6, 129.8, 128.8, 128.0, 127.8, 127.4, 126.7, 119.1, 118.7, 64.9 (neg.), 45.6, 44.1 (neg.). HRMS (ESI) m/z calcd for : $C_{22}H_{19}O_3^-$ (M-H)$^-$ 331.1334, found 331.1337.

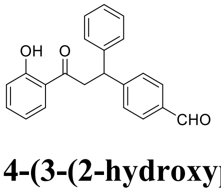

4-(3-(2-hydroxyphenyl)-3-oxo-1-phenylpropyl)benzaldehyde (14):
Yield: 69%; 113.9 mg; yellow oil; TLC (nHex:EtOAc, 8:1 v/v): R_f = 0.3; ^{1}H NMR (600 MHz, $CDCl_3$) δ = 12.08 (s, 1H), 9.89 (s, 1H), 7.80 (d, *J* = 8.0 Hz, 1H), 7.76 (d, *J* = 7.9 Hz, 2H), 7.41 (t, *J* = 7.2 Hz, 3H), 7.29 – 7.23 (m, 4H), 7.19 (d, *J* = 7.0 Hz, 1H), 6.92 (d, *J* = 8.4 Hz, 1H), 6.86

(s, 1H), 4.85 (s, 1H), 3.79 (t, *J* = 7.6 Hz, 2H). ^{13}C NMR (150 MHz, $CDCl_3$) δ = 203.2, 191.6, 162.3, 150.6, 142.5, 136.5, 134.7, 130.0, 129.5, 128.7, 128.3, 127.6, 126.8, 119.1, 118.9, 118.4, 45.6, 43.4. DEPT-135 (150 MHz, $CDCl_3$) δ = 136.7, 130.2, 129.8, 129.0, 128.6, 127.8, 127.1, 119.2, 118.7, 45.9, 43.7 (neg). HRMS (ESI) m/z calcd for : $C_{22}H_{17}O_3^-$ (M-H)$^-$ 329.1178, found 329.1181.

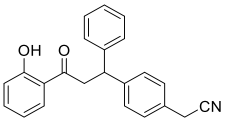

2-(4-(3-(2-hydroxyphenyl)-3-oxo-1-phenylpropyl)phenyl)acetonitrile (15):
Yield: 46%; 78.5 mg; yellow oil; TLC (nHex:EtOAc, 6:1 v/v): R_f = 0.3; ^{1}H NMR (600 MHz, $CDCl_3$) δ = 12.11 (s, 1H), 7.78 (d, *J* = 8.0 Hz, 1H), 7.42 (t, *J* = 7.7 Hz, 1H), 7.28 – 7.22 (m, 6H), 7.18 (dd, *J* = 13.7, 7.6 Hz, 3H), 6.92 (d, *J* = 8.4 Hz, 1H), 6.86 (t, *J* = 7.6 Hz, 1H), 4.77 (s, 1H), 3.73 (t, *J* = 7.3 Hz, 2H), 3.61 (s, 2H). ^{13}C NMR (150 MHz, $CDCl_3$) δ = 203.6, 162.3, 143.6, 143.2, 136.4, 129.6, 128.6, 128.3, 128.1, 128.0, 127.5, 126.6, 119.2, 118.9, 118.4, 117.7, 45.2, 43.7, 22.9. DEPT-135 (150 MHz, $CDCl_3$) δ = 136.6, 129.8, 128.9, 128.6, 128.3, 127.8, 126.8, 119.1, 118.7, 45.4, 44.0 (neg.), 23.2 (neg.). HRMS (ESI) m/z calcd for : $C_{23}H_{18}NO_2^-$ (M-H)$^-$ 340.1338, found 340.1337.

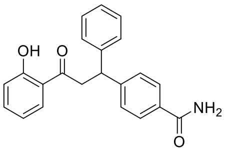

4-(3-(2-hydroxyphenyl)-3-oxo-1-phenylpropyl)benzamide (16):
Yield: 72%; 124.6 mg; white solid; m.p. 167-168 °C; TLC (nHex:EtOAc, 1:1 v/v): R_f = 0.3; ^{1}H NMR (600 MHz, DMSO-*d*$_6$) δ = 11.83 (s, 1H), 8.07 (d, *J* = 7.6 Hz, 1H), 7.96 (s, 1H), 7.83 (d, *J* = 7.6 Hz, 2H), 7.50 (d, *J* = 7.5 Hz, 3H), 7.42 (d, *J* = 7.2 Hz, 2H), 7.36 (s, 1H), 7.27 (t, *J* = 7.0 Hz, 2H), 7.16 (d, *J* = 6.9 Hz, 1H), 6.96 (t, *J* = 7.9 Hz, 2H), 4.75 (s, 1H), 4.03 (dd, *J* = 11.9, 7.7 Hz, 2H). ^{13}C NMR (150 MHz, DMSO-*d*$_6$) δ = 204.0, 167.9, 160.6, 147.8, 144.0, 136.2, 132.3, 131.0, 128.5, 127.7, 127.5, 126.3, 120.5, 119.3, 117.7, 45.5, 43.8. DEPT-135 (150 MHz, DMSO-*d*$_6$) δ = 136.6, 131.4, 129.0, 128.2, 128.0, 126.8, 119.7, 118.1, 45.9, 44.2 (neg.). HRMS (ESI) m/z calcd for : $C_{22}H_{18}NO_3^-$ (M-H)$^-$ 344.1287, found 344.1290.

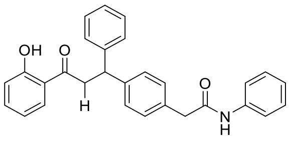

2-(4-(3-(2-hydroxyphenyl)-3-oxo-1-phenylpropyl)phenyl)-N-phenylacetamide (17):
Yield: 66%; 143.7 mg; yellow oil; TLC (nHex:EtOAc, 4:1 v/v): R_f = 0.3; ^{1}H NMR (600 MHz, $CDCl_3$) δ = 12.15 (s, 1H), 7.77 (dd, *J* = 8.0, 1.2 Hz, 1H), 7.65 (s, 1H), 7.43 – 7.37 (m, 3H), 7.27 – 7.15 (m, 11H), 7.02 (t, *J* = 7.4 Hz, 1H), 6.92 (d, *J* = 8.4 Hz, 1H), 6.87 – 6.83 (m, 1H), 4.76 (t, *J* = 7.3 Hz, 1H), 3.75 – 3.67 (m, 2H), 3.57 (s, 2H). ^{13}C NMR (150 MHz, $CDCl_3$) δ = 203.9, 169.3, 162.3, 143.3, 142.9, 137.6, 136.4, 132.6, 129.6, 129.5, 128.8, 128.6, 128.3, 127.6, 126.5, 124.3, 119.9, 119.3, 118.9, 118.4, 45.3, 44.0, 43.9. DEPT-135 (150 MHz, $CDCl_3$) δ = 136.6, 129.9, 129.8, 129.0, 128.8, 128.5, 127.8, 126.7, 124.5, 120.1, 119.1, 118.6, 45.5, 44.2 (neg), 44.1 (neg.). HRMS (ESI) m/z calcd for : $C_{29}H_{24}NO_3^-$ (M-H)$^-$ 434.1756, found 434.1758.

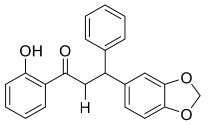

3-(benzo[d][1,3]dioxol-5-yl)-1-(2-hydroxyphenyl)-3-phenylpropan-1-one (18):
Yield: 68%; 117.8 mg; yellow oil; TLC (nHex:EtOAc, 50:1 v/v): $R_f = 0.3$; ^{1}H NMR (600 MHz, $CDCl_3$) δ = 12.17 (s, 1H), 7.75 (d, J = 7.9 Hz, 1H), 7.39 (t, J = 7.7 Hz, 1H), 7.24 (dt, J = 14.3, 7.4 Hz, 4H), 7.16 (d, J = 6.8 Hz, 1H), 6.92 (d, J = 8.3 Hz, 1H), 6.83 (t, J = 7.5 Hz, 1H), 6.68 (dd, J = 16.7, 6.6 Hz, 3H), 5.80 (s, 2H), 4.69 (t, J = 7.2 Hz, 1H), 3.66 (d, J = 7.3 Hz, 2H). ^{13}C NMR (150 MHz, $CDCl_3$) δ = 203.8, 162.3, 147.7, 146.0, 143.7, 137.6, 136.3, 129.6, 128.5, 127.4, 126.4, 120.4, 119.3, 118.8, 118.4, 108.2, 108.1, 100.8, 45.3, 44.0. DEPT-135 (150 MHz, $CDCl_3$) δ = 136.6, 129.8, 128.8, 127.7, 126.7, 120.7, 119.1, 118.7, 108.4, 108.3, 101.1 (neg.), 45.5, 44.3 (neg.). HRMS (ESI) m/z calcd for : $C_{22}H_{17}O_4^-$ $(M-H)^-$ 345.1127, found 345.1128.

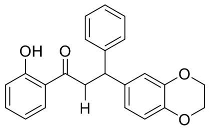

3-(2,3-dihydrobenzo[b][1,4]dioxin-6-yl)-1-(2-hydroxyphenyl)-3-phenylpropan-1-one (19):
Yield: 66%; 118.9 mg; yellow oil; TLC (nHex:EtOAc, 15:1 v/v): $R_f = 0.3$; ^{1}H NMR (600 MHz, $CDCl_3$) δ = 12.17 (s, 1H), 7.76 (s, 1H), 7.41 (s, 1H), 7.20 (d, J = 48.8 Hz, 5H), 6.81 (dd, J = 87.4, 33.5 Hz, 5H), 4.66 (s, 1H), 4.14 (s, 4H), 3.68 (s, 2H). ^{13}C NMR (150 MHz, $CDCl_3$) δ = 203.9, 162.3, 143.8, 143.3, 142.0, 137.0, 136.3, 129.6, 128.5, 127.5, 126.4, 120.5, 119.3, 118.8, 118.4, 117.2, 116.3, 64.2, 64.1, 44.9, 44.0. DEPT-135 (150 MHz, $CDCl_3$) δ = 136.5, 129.8, 128.7, 127.7, 126.6, 120.7, 119.0, 118.6, 117.4, 116.5, 64.4 (neg), 64.3 (neg), 45.1, 44.2 (neg). HRMS (ESI) m/z calcd for : $C_{23}H_{19}O_4^-$ $(M-H)^-$ 359.1283, found 359.1285.

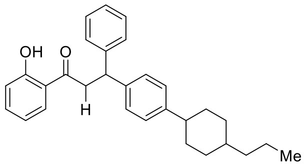

1-(2-hydroxyphenyl)-3-phenyl-3-(4-(4-propylcyclohexyl)phenyl)propan-1-one (20):
Yield: 73%; 155.5 mg; yellow oil; TLC (nHex:EtOAc, 100:1 v/v): $R_f = 0.3$; ^{1}H NMR (600 MHz, $CDCl_3$) δ = 12.18 (s, 1H), 7.74 (d, J = 8.0 Hz, 1H), 7.38 (s, 1H), 7.24 (d, J = 3.8 Hz, 4H), 7.15 (d, J = 7.5 Hz, 3H), 7.10 (d, J = 7.8 Hz, 2H), 6.91 (d, J = 8.3 Hz, 1H), 6.82 (s, 1H), 4.74 (t, J = 7.2 Hz, 1H), 3.71 (d, J = 4.1 Hz, 2H), 2.39 (t, J = 12.1 Hz, 1H), 1.83 (t, J = 10.4 Hz, 4H), 1.35 (dd, J = 36.2, 9.8 Hz, 4H), 1.26 (s, 1H), 1.21 – 1.15 (m, 2H), 1.00 (d, J = 12.1 Hz, 2H), 0.88 (t, J = 7.3 Hz, 3H). ^{13}C NMR (150 MHz, $CDCl_3$) δ = 204.0, 162.4, 145.9, 143.8, 141.0, 136.2, 129.7, 128.5, 127.7, 127.5, 127.0, 126.4, 119.4, 118.8, 118.5, 45.3, 44.2, 44.0, 39.6, 36.9, 34.2, 33.5, 20.0, 14.4. DEPT-135 (150 MHz, $CDCl_3$) δ = 136.4, 129.9, 128.7, 127.9, 127.7, 127.2, 126.6, 119.0, 118.7, 45.5, 44.4 (neg.), 44.2, 39.9 (neg.), 37.1, 34.4 (neg.), 33.7 (neg.), 20.2 (neg.), 14.6. HRMS (ESI) m/z calcd for : $C_{30}H_{33}O_2^-$ $(M-H)^-$ 425.2481, found 425.2483.

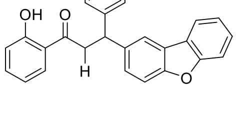

3-(dibenzo[b,d]furan-2-yl)-1-(2-hydroxyphenyl)-3-phenylpropan-1-one (21):

Yield: 61%; 119.4 mg; white solid; mp 140-141 °C; TLC (nHex:EtOAc, 50:1 v/v): R_f = 0.3; ^{1}H NMR (600 MHz, $CDCl_3$) δ = 12.17 (s, 1H), 7.85 (d, J = 7.6 Hz, 1H), 7.83 – 7.77 (m, 2H), 7.49 (d, J = 8.2 Hz, 1H), 7.43 (d, J = 8.5 Hz, 1H), 7.38 (q, J = 8.0 Hz, 2H), 7.33 – 7.25 (m, 6H), 7.18 (d, J = 6.7 Hz, 1H), 6.92 (d, J = 8.3 Hz, 1H), 6.83 (t, J = 7.5 Hz, 1H), 4.95 (t, J = 7.2 Hz, 1H), 3.81 (d, J = 7.3 Hz, 2H). ^{13}C NMR (150 MHz, $CDCl_3$) δ = 203.9, 162.4, 156.5, 154.8, 143.9, 138.4, 136.4, 129.6, 128.7, 127.7, 127.1, 126.9, 126.5, 124.4, 124.0, 122.6, 120.6, 119.6, 119.4, 118.9, 118.5, 111.6, 111.5, 45.6, 44.4. DEPT-135 (150 MHz, $CDCl_3$) δ = 136.6, 129.8, 128.8, 127.8, 127.3, 127.1, 126.7, 122.7, 120.8, 119.7, 119.1, 118.7, 111.8, 111.7, 45.8, 44.6 (neg). HRMS (ESI) m/z calcd for : $C_{27}H_{19}O_3^-$ $(M-H)^-$ 391.1334, found 391.1335.

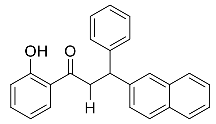

1-(2-hydroxyphenyl)-3-(naphthalen-2-yl)-3-phenylpropan-1-one (22):

Yield: 73%; 128.3 mg; yellow oil; TLC (nHex:EtOAc, 50:1 v/v): R_f = 0.3; ^{1}H NMR (600 MHz, $CDCl_3$) δ = 12.19 (s, 1H), 7.70 (dd, J = 22.8, 10.2 Hz, 5H), 7.34 (dt, J = 16.0, 8.3 Hz, 4H), 7.23 (dt, J = 15.3, 7.6 Hz, 4H), 7.14 (d, J = 7.0 Hz, 1H), 6.89 (d, J = 8.4 Hz, 1H), 6.77 (t, J = 7.6 Hz, 1H), 4.92 (t, J = 7.2 Hz, 1H), 3.76 (t, J = 6.1 Hz, 2H). ^{13}C NMR (150 MHz, $CDCl_3$) δ = 203.8, 162.4, 143.5, 141.1, 136.3, 133.3, 132.1, 129.6, 128.6, 128.3, 127.8, 127.6, 127.5, 126.5, 126.5, 126.0, 125.6, 125.5, 119.3, 118.8, 118.4, 45.7, 43.8. DEPT-135 (150 MHz, $CDCl_3$) δ = 136.6, 129.9, 128.9, 128.6, 128.1, 128.0, 127.8, 126.8, 126.8, 126.4, 125.9, 125.9, 119.1, 118.8, 46.0, 44.1 (neg.). HRMS (ESI) m/z calcd for : $C_{25}H_{19}O_2^-$ $(M-H)^-$ 351.1385, found 351.1396.

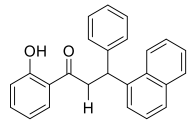

1-(2-hydroxyphenyl)-3-(naphthalen-1-yl)-3-phenylpropan-1-one (23):

Yield: 70%; 123.2 mg; white solid; mp 127-129 °C; TLC (nHex:EtOAc, 50:1 v/v): R_f = 0.3; ^{1}H NMR (600 MHz, $CDCl_3$) δ = 12.18 (s, 1H), 8.14 (d, J = 8.1 Hz, 1H), 7.80 (d, J = 7.8 Hz, 1H), 7.71 (dd, J = 10.8, 8.8 Hz, 2H), 7.44 – 7.35 (m, 4H), 7.32 (d, J = 7.1 Hz, 1H), 7.25 (d, J = 7.7 Hz, 2H), 7.21 (t, J = 7.5 Hz, 2H), 7.12 (t, J = 7.1 Hz, 1H), 6.92 (d, J = 8.4 Hz, 1H), 6.78 (t, J = 7.6 Hz, 1H), 5.61 (t, J = 7.1 Hz, 1H), 3.82 (t, J = 6.1 Hz, 2H). ^{13}C NMR (150 MHz, $CDCl_3$) δ = 203.8, 162.4, 143.4, 139.1, 136.4, 134.1, 131.4, 129.6, 128.9, 128.6, 127.9, 127.4, 126.5, 126.3, 125.6, 125.2, 124.2, 123.6, 119.4, 118.9, 118.5, 44.3, 41.1. DEPT-135 (150 MHz, $CDCl_3$) δ = 136.6, 129.8, 129.1, 128.8, 128.1, 127.6, 126.7, 126.5, 125.8, 125.4, 124.4, 123.8, 119.1, 118.7, 44.5 (neg.), 41.3. HRMS (ESI) m/z calcd for : $C_{25}H_{19}O_2^-$ $(M-H)^-$ 351.1385, found 351.1391.

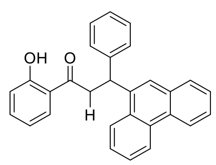

1-(2-hydroxyphenyl)-3-(phenanthren-9-yl)-3-phenylpropan-1-one (24):

Yield: 60%; 120.6 mg; white solid; mp 153-156 °C; TLC (nHex:EtOAc, 50:1 v/v): R_f = 0.3; ^{1}H NMR (600 MHz, $CDCl_3$) δ = 12.19 (s, 1H), 8.67 (d, J = 8.2 Hz, 1H), 8.60 (d, J = 8.2 Hz, 1H), 8.17 (d, J = 8.2 Hz, 1H), 7.77 (dd, J = 11.1, 8.4 Hz, 2H), 7.57 (dd, J = 14.0, 6.3 Hz, 3H),

7.52 (t, J = 7.2 Hz, 2H), 7.39 (t, J = 7.8 Hz, 1H), 7.31 (d, J = 7.7 Hz, 2H), 7.22 (t, J = 7.5 Hz, 2H), 7.14 (t, J = 7.3 Hz, 1H), 6.93 (d, J = 8.4 Hz, 1H), 6.81 (t, J = 7.6 Hz, 1H), 5.61 (t, J = 7.0 Hz, 1H), 3.89 (ddd, J = 25.1, 17.4, 7.1 Hz, 2H). ^{13}C NMR (150 MHz, $CDCl_3$) δ = 203.9, 162.5, 143.2, 137.3, 136.4, 131.3, 131.0, 130.6, 129.8, 129.6, 128.7, 128.5, 128.0, 126.8, 126.7, 126.6, 126.5, 126.3, 125.1, 124.5, 123.2, 122.4, 119.4, 118.9, 118.6, 44.3, 41.5. DEPT-135 (150 MHz, $CDCl_3$) δ = 136.6, 129.8, 128.9, 128.6, 128.1, 127.0, 126.8, 126.8, 126.7, 126.5, 125.2, 124.6, 123.4, 122.6, 119.1, 118.7, 44.5 (neg.), 41.6. HRMS (ESI) m/z calcd for : $C_{29}H_{21}O_2^-$ $(M-H)^-$ 401.1542, found 401.1550.

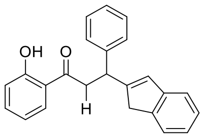

1-(2-hydroxyphenyl)-3-(1H-inden-2-yl)-3-phenylpropan-1-one (25):

Yield: 53%; 90.5 mg; yellow oil; TLC (nHex:EtOAc, 100:1 v/v): $R_f = 0.3$; ^{1}H NMR (600 MHz, $CDCl_3$) δ = 12.22 (d, *J* = 15.4 Hz, 1H), 7.79 (dd, *J* = 17.7, 7.6 Hz, 1H), 7.47-7.40 (m, 2H), 7.35-7.26 (m, 4H), 7.22-7.07 (m, 4H), 6.96 (d, *J* = 8.3 Hz, 1H), 6.87 (dt, *J* = 15.5, 7.6 Hz, 1H), 6.63-6.31 (s, 1H), 4.79-4.57 (m, 1H), 3.80 (ddd, *J* = 31.4, 17.0, 7.3 Hz, 1H), 3.60-3.53 (m, 1H), 3.39-3.18 (m, 2H). ^{13}C NMR (150 MHz, $CDCl_3$) δ = 204.1, 203.9, 162.5, 162.4, 151.5, 145.9, 144.7, 144.4, 144.2, 143.1, 143.0, 142.1, 136.4, 136.3, 129.7, 129.6, 128.7, 128.6, 128.2, 127.9, 127.8, 126.8, 126.7, 126.4, 126.3, 126.0, 124.8, 124.2, 123.7, 123.5, 120.5, 119.9, 119.4, 119.3, 118.9, 118.8, 118.6, 118.5, 43.9, 43.6, 42.5, 40.5, 39.6, 37.8. DEPT-135 (150 MHz, $CDCl_3$) δ = 136.6, 136.5, 129.8, 129.8, 128.8, 128.7, 128.3, 128.0, 127.9, 126.9, 126.8, 126.5, 126.4, 126.1, 124.9, 124.3, 123.8, 123.6, 120.6, 120.0, 119.0, 118.9, 118.7, 118.6, 44.1 (neg.), 43.7 (neg.), 42.6, 40.6 (neg.), 39.7, 37.9 (neg.). HRMS (ESI) m/z calcd for : $C_{24}H_{19}O_2^-$ $(M-H)^-$ 339.1385, found 339.1387.

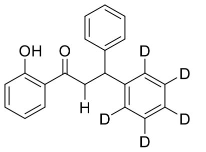

1-(2-hydroxyphenyl)-3-phenyl-3-(phenyl-*d*$_5$)propan-1-one (26):

Yield: 47%; 72.0 mg; yellow solid; m.p. 96-98 °C; TLC (nHex:EtOAc, 100:1 v/v): $R_f = 0.3$; ^{1}H NMR (600 MHz, $CDCl_3$) δ = 12.16 (s, 1H), 7.81 (dd, *J* = 8.0, 1.3 Hz, 1H), 7.47 – 7.42 (m, 1H), 7.30 – 7.25 (m, 4H), 7.19 (d, *J* = 6.9 Hz, 1H), 6.95 (d, *J* = 8.3 Hz, 1H), 6.90 – 6.85 (m, 1H), 4.80 (t, *J* = 7.3 Hz, 1H), 3.76 (d, *J* = 7.4 Hz, 2H). ^{13}C NMR (150 MHz, $CDCl_3$) δ = 204.1, 162.5, 143.8, 136.5, 129.8, 128.7, 127.8, 126.6, 119.5, 119.0, 118.7, 45.7, 44.2. DEPT-135 (150 MHz, $CDCl_3$) δ = 136.5, 129.8, 128.7, 127.8, 126.6, 119.0, 118.7, 45.7, 44.2 (neg.). HRMS (ESI) m/z calcd for : $C_{21}H_{12}D_5O_2^-$ $(M-H)^-$ 306.1542, found 306.1545.

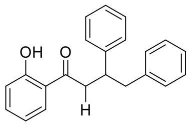

1-(2-hydroxyphenyl)-3,4-diphenylbutan-1-one (27):

Yield: 63%; 99.7 mg; yellow oil; TLC (nHex:EtOAc, 100:1 v/v): $R_f = 0.3$; ^{1}H NMR (600 MHz, $CDCl_3$) δ = 12.21 (s, 1H), 7.63 (d, J = 8.0 Hz, 1H), 7.41 (t, J = 7.7 Hz, 1H), 7.23 (dd, J = 18.2, 7.2 Hz, 4H), 7.19 – 7.15 (m, 4H), 7.08 (d, J = 7.4 Hz, 2H), 6.92 (d, J = 8.3 Hz, 1H), 6.83 (t, J

= 7.6 Hz, 1H), 3.68 – 3.60 (m, 1H), 3.38 – 3.26 (m, 2H), 2.98 (d, J = 7.5 Hz, 2H). ^{13}C NMR (150 MHz, $CDCl_3$) δ = 205.0, 162.4, 143.7, 139.5, 136.2, 129.8, 129.2, 128.4, 128.3, 127.5, 126.6, 126.2, 119.5, 118.8, 118.5, 43.5, 43.1, 43.0. DEPT-135 (150 MHz, $CDCl_3$) δ = 136.3, 129.9, 129.3, 128.5, 128.3, 127.6, 126.6, 126.3, 118.8, 118.6, 43.6 (neg.), 43.1, 43.0 (neg.). HRMS (ESI) m/z calcd for : $C_{22}H_{19}O_2^-$ (M-H)$^-$ 315.1385, found 315.1386.

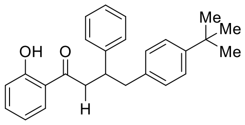

4-(4-(tert-butyl)phenyl)-1-(2-hydroxyphenyl)-3-phenylbutan-1-one (28):

Yield: 52%; 97.7 mg; yellow solid; m.p. 126-128 °C; TLC (nHex:EtOAc, 200:1 v/v): R_f = 0.3; ^{1}H NMR (600 MHz, $CDCl_3$) δ = 12.20 (s, 1H), 7.60 (d, *J* = 7.9 Hz, 1H), 7.40 (t, *J* = 7.6 Hz, 1H), 7.29 – 7.22 (m, 6H), 7.18 (t, *J* = 7.0 Hz, 1H), 7.06 (d, *J* = 7.8 Hz, 2H), 6.90 (d, *J* = 8.3 Hz, 1H), 6.81 (t, *J* = 7.5 Hz, 1H), 3.71 – 3.58 (m, 1H), 3.37 – 3.24 (m, 2H), 3.01 (dd, *J* = 13.6, 6.4 Hz, 1H), 2.90 (dd, *J* = 13.5, 8.6 Hz, 1H), 1.28 (s, 9H). ^{13}C NMR (150 MHz, $CDCl_3$) δ = 205.1, 162.4, 149.2, 144.1, 136.5, 136.2, 129.9, 128.9, 128.5, 127.5, 126.6, 125.3, 119.5, 118.7, 118.5, 43.5, 43.1, 42.6, 34.4, 31.4. DEPT-135 (150 MHz, $CDCl_3$) δ = 136.2, 129.9, 128.9, 128.5, 127.5, 126.6, 125.3, 118.8, 118.5, 43.5 (neg.), 43.1, 42.7 (neg.), 31.4. HRMS (ESI) m/z calcd for : $C_{26}H_{27}O_2^-$ (M-H)$^-$ 371.2011, found 371.2010.

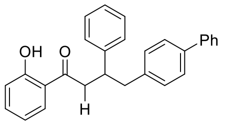

4-([1,1'-biphenyl]-4-yl)-1-(2-hydroxyphenyl)-3-phenylbutan-1-one (29):

Yield: 64%; 125.2 mg; white solid; m.p. 108-110 °C; TLC (nHex:EtOAc, 100:1 v/v): R_f = 0.3; ^{1}H NMR (600 MHz, $CDCl_3$) δ = 12.23 (s, 1H), 7.63 (d, *J* = 7.7 Hz, 1H), 7.54 (d, *J* = 7.5 Hz, 2H), 7.45 (d, *J* = 8.0 Hz, 2H), 7.39 (t, *J* = 7.6 Hz, 3H), 7.30 (t, *J* = 7.3 Hz, 1H), 7.26 (t, *J* = 7.5 Hz, 2H), 7.21 – 7.12 (m, 5H), 6.91 (d, *J* = 8.3 Hz, 1H), 6.81 (t, *J* = 7.6 Hz, 1H), 3.72 – 3.64 (m, 1H), 3.33 (qd, *J* = 16.6, 7.0 Hz, 2H), 3.01 (d, *J* = 7.4 Hz, 2H). ^{13}C NMR (150 MHz, $CDCl_3$) δ = 204.9, 162.4, 143.6, 140.8, 139.0, 138.6, 136.2, 129.8, 129.6, 128.7, 128.5, 127.5, 127.1, 126.9, 126.8, 126.6, 119.5, 118.7, 118.5, 43.5, 43.0, 42.6. DEPT-135 (150 MHz, $CDCl_3$) δ = 136.4, 129.9, 129.7, 128.8, 128.6, 127.7, 127.2, 127.1, 127.0, 126.7, 118.9, 118.6, 43.6 (neg.), 43.1, 42.7 (neg.). HRMS (ESI) m/z calcd for : $C_{28}H_{23}O_2^-$ (M-H)$^-$ 391.1698, found 391.1699.

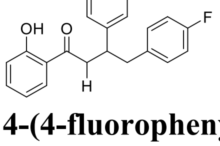

4-(4-fluorophenyl)-1-(2-hydroxyphenyl)-3-phenylbutan-1-one (30):

Yield: 67%; 111.5 mg; yellow oil; TLC (nHex:EtOAc, 100:1 v/v): R_f = 0.3; ^{1}H NMR (600 MHz, $CDCl_3$) δ = 12.23 (s, 1H), 7.65 (dd, *J* = 8.0, 1.1 Hz, 1H), 7.44 – 7.38 (m, 1H), 7.24 (t, *J* = 7.5 Hz, 2H), 7.18 – 7.12 (m, 3H), 6.98 (dd, *J* = 8.4, 5.6 Hz, 2H), 6.93 (d, *J* = 8.3 Hz, 1H), 6.85 (dt, *J* = 15.2, 8.0 Hz, 3H), 3.63 – 3.55 (m, 1H), 3.32 (ddd, *J* = 37.5, 16.7, 7.0 Hz, 2H), 2.94 (ddd, *J* = 21.7, 13.6, 7.4 Hz, 2H). ^{13}C NMR (150 MHz, $CDCl_3$) δ = 204.8, 162.4, 162.2, 160.6, 143.3, 136.3, 135.2, 130.5, 129.7, 128.4, 127.5, 126.6, 119.4, 118.8, 118.5, 115.0, 43.5, 43.1, 42.0. DEPT-135 (150 MHz, $CDCl_3$) δ = 136.4, 130.6, 129.8, 128.6, 127.6, 126.8, 118.9, 118.6, 115.1, 43.6 (neg.), 43.2, 42.2 (neg.). ^{19}F NMR (564 MHz, $CDCl_3$) δ = -116.84. HRMS (ESI) m/z calcd for : $C_{22}H_{18}FO_2^-$ (M-H)$^-$ 333.1291, found 333.1296.

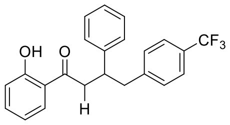

1-(2-hydroxyphenyl)-3-phenyl-4-(4-(trifluoromethyl)phenyl)butan-1-one (31):

Yield: 60%; 114.9 mg; yellow oil; TLC (nHex:EtOAc, 100:1 v/v): R_f = 0.3; ^{1}H NMR (600 MHz, $CDCl_3$) δ = 12.21 (s, 1H), 7.66 (d, *J* = 7.9 Hz, 1H), 7.42 (dd, *J* = 13.1, 7.8 Hz, 3H), 7.24 (t, *J* = 7.4 Hz, 2H), 7.19 – 7.11 (m, 5H), 6.94 (d, *J* = 8.3 Hz, 1H), 6.84 (t, *J* = 7.6 Hz, 1H), 3.69 – 3.61 (m, 1H), 3.38 (dd, *J* = 16.8, 6.9 Hz, 1H), 3.30 (dd, *J* = 16.8, 6.9 Hz, 1H), 3.09 (dd, *J* = 13.5, 6.3 Hz, 1H), 2.97 (dd, *J* = 13.4, 8.5 Hz, 1H). ^{13}C NMR (150 MHz, $CDCl_3$) δ = 204.6, 162.4, 143.7, 142.9, 136.4, 129.7, 129.5, 128.5, 128.3, 127.5, 126.8, 125.1, 123.3, 119.4, 118.8, 118.5, 43.7, 42.7, 42.5. DEPT-135 (150 MHz, $CDCl_3$) δ = 136.5, 129.8, 129.6, 128.6, 127.6, 126.9, 125.2, 119.0, 118.6, 43.8 (neg.), 42.8, 42.6 (neg.). ^{19}F NMR (564 MHz, $CDCl_3$) δ = -62.24. HRMS (ESI) m/z calcd for : $C_{23}H_{18}F_3O_2^-$ $(M-H)^-$ 383.1259, found 383.1256.

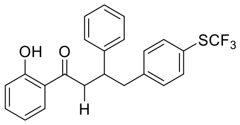

1-(2-hydroxyphenyl)-3-phenyl-4-(4-((trifluoromethyl)thio)phenyl)butan-1-one (32):

Yield: 59%; 121.8 mg; yellow oil; TLC (nHex:EtOAc, 80:1 v/v): R_f = 0.3; ^{1}H NMR (600 MHz, $CDCl_3$) δ = 12.20 (s, 1H), 7.65 (dd, *J* = 8.0, 0.9 Hz, 1H), 7.47 (d, *J* = 8.0 Hz, 2H), 7.44 – 7.40 (m, 1H), 7.24 (t, *J* = 7.5 Hz, 2H), 7.18 (d, *J* = 7.3 Hz, 1H), 7.13 (d, *J* = 7.3 Hz, 2H), 7.08 (d, *J* = 8.1 Hz, 2H), 6.94 (d, *J* = 8.4 Hz, 1H), 6.84 (t, *J* = 7.6 Hz, 1H), 3.67 – 3.60 (m, 1H), 3.34 (ddd, *J* = 41.0, 16.8, 6.9 Hz, 2H), 3.01 (ddd, *J* = 21.9, 13.5, 7.4 Hz, 2H). ^{13}C NMR (150 MHz, $CDCl_3$) δ = 204.6, 162.4, 142.9, 142.8, 136.4, 136.2, 130.3, 129.7, 128.5, 127.5, 126.8, 121.8, 121.7, 119.4, 118.8, 118.6, 43.7, 42.8, 42.5. DEPT-135 (150 MHz, $CDCl_3$) δ = 136.5, 136.3, 130.4, 129.8, 128.6, 127.6, 126.9, 118.9, 118.6, 43.8 (neg.), 42.8, 42.6 (neg.). ^{19}F NMR (564 MHz, $CDCl_3$) δ = -42.93. HRMS (ESI) m/z calcd for : $C_{23}H_{18}F_3O_2S^-$ $(M-H)^-$ 415.0980, found 415.0978.

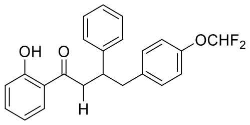

4-(4-(difluoromethoxy)phenyl)-1-(2-hydroxyphenyl)-3-phenylbutan-1-one (33):

Yield: 62%; 118.5 mg; yellow oil; TLC (nHex:EtOAc, 50:1 v/v): R_f = 0.3; ^{1}H NMR (600 MHz, $CDCl_3$) δ = 12.22 (s, 1H), 7.64 (d, *J* = 7.9 Hz, 1H), 7.40 (t, *J* = 7.6 Hz, 1H), 7.24 (t, *J* = 7.4 Hz, 2H), 7.16 (dd, *J* = 13.8, 7.3 Hz, 3H), 7.02 (d, *J* = 8.3 Hz, 2H), 6.96 – 6.87 (m, 3H), 6.83 (t, *J* = 7.6 Hz, 1H), 6.42 (t, *J* = 74.2 Hz, 1H), 3.66 – 3.55 (m, 1H), 3.31 (qd, *J* = 16.7, 6.9 Hz, 2H), 2.95 (ddd, *J* = 21.6, 13.6, 7.5 Hz, 2H). ^{13}C NMR (150 MHz, $CDCl_3$) δ = 204.7, 162.4, 149.6, 143.3, 136.7, 136.3, 130.5, 129.7, 128.5, 127.5, 126.7, 119.4, 119.2, 118.8, 118.5, 116.0, 43.6, 42.9, 42.1. DEPT-135 (150 MHz, $CDCl_3$) δ = 136.5, 130.6, 129.9, 128.6, 127.6, 126.8, 119.3, 118.9, 118.6, 116.1, 43.7 (neg.), 43.1, 42.2 (neg.). ^{19}F NMR (564 MHz, $CDCl_3$) δ = -80.33. HRMS (ESI) m/z calcd for : $C_{23}H_{19}F_2O_3^-$ $(M-H)^-$ 381.1302, found 381.1302.

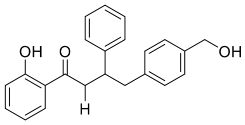

4-(4-(hydroxymethyl)phenyl)-1-(2-hydroxyphenyl)-3-phenylbutan-1-one (34):

Yield: 51%; 88.0 mg; yellow oil; TLC (nHex:EtOAc, 10:1 v/v): $R_f = 0.3$; ^{1}H NMR (600 MHz, $CDCl_3$) δ = 12.19 (s, 1H), 7.64 (d, *J* = 7.7 Hz, 1H), 7.41 (t, *J* = 7.7 Hz, 1H), 7.24 (d, *J* = 7.5 Hz, 2H), 7.18 (dd, *J* = 14.0, 8.2 Hz, 5H), 7.06 (d, *J* = 7.6 Hz, 2H), 6.91 (d, *J* = 8.3 Hz, 1H), 6.83 (t, *J* = 7.5 Hz, 1H), 4.59 (s, 2H), 3.64 (d, *J* = 7.1 Hz, 1H), 3.31 (dd, *J* = 10.6, 7.1 Hz, 2H), 2.97 (d, *J* = 7.4 Hz, 2H). ^{13}C NMR (150 MHz, $CDCl_3$) δ = 204.9, 162.3, 143.6, 138.9, 138.7, 136.2, 129.7, 129.4, 128.4, 127.5, 127.0, 126.6, 119.5, 118.8, 118.4, 65.1, 43.6, 43.0, 42.7. DEPT-135 (150 MHz, $CDCl_3$) δ = 136.3, 129.8, 129.5, 128.5, 127.6, 127.1, 126.7, 118.9, 118.5, 65.2 (neg.), 43.7 (neg.), 43.1, 42.8 (neg.). HRMS (ESI) m/z calcd for : $C_{23}H_{21}O_3^-$ (M-H)$^-$ 345.1491, found 345.1490.

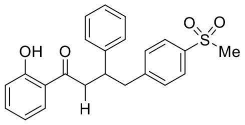

1-(2-hydroxyphenyl)-4-(4-(methylsulfonyl)phenyl)-3-phenylbutan-1-one (35):

Yield: 62%; 122.2 mg; yellow oil; TLC (nHex:EtOAc, 4:1 v/v): $R_f = 0.3$; ^{1}H NMR (600 MHz, $CDCl_3$) δ = 12.18 (s, 1H), 7.74 (d, *J* = 8.1 Hz, 2H), 7.70 (d, *J* = 7.9 Hz, 1H), 7.44 (s, 1H), 7.25 – 7.16 (m, 5H), 7.14 (d, *J* = 7.4 Hz, 2H), 6.93 (d, *J* = 8.3 Hz, 1H), 6.86 (s, 1H), 3.71 – 3.63 (m, 1H), 3.37 (qd, *J* = 17.0, 6.9 Hz, 2H), 3.15 (dd, *J* = 13.4, 6.0 Hz, 1H), 2.99 (s, 4H). ^{13}C NMR (150 MHz, $CDCl_3$) δ = 204.4, 162.3, 146.2, 142.6, 138.2, 136.4, 130.0, 129.6, 128.6, 127.4, 127.2, 126.9, 119.3, 118.9, 118.5, 44.4, 43.8, 42.5, 42.4. DEPT-135 (150 MHz, $CDCl_3$) δ = 136.6, 130.2, 129.8, 128.7, 127.6, 127.3, 127.0, 119.0, 118.6, 44.5, 43.9 (neg.), 42.7 (neg.), 42.6. HRMS (ESI) m/z calcd for : $C_{23}H_{21}O_4S^-$ (M-H)$^-$ 393.1161, found 393.1165.

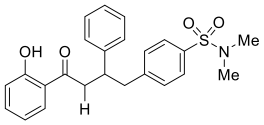

4-(4-(2-hydroxyphenyl)-4-oxo-2-phenylbutyl)-N,N-dimethylbenzenesulfonamide (36):

Yield: 65%; 137.1 mg; yellow oil; TLC (nHex:EtOAc, 4:1 v/v): $R_f = 0.3$; ^{1}H NMR (600 MHz, $CDCl_3$) δ = 12.20 (s, 1H), 7.74 – 7.69 (m, 1H), 7.57 (d, *J* = 8.2 Hz, 2H), 7.44 (d, *J* = 7.2 Hz, 1H), 7.23 (t, *J* = 7.4 Hz, 2H), 7.20 – 7.16 (m, 3H), 7.11 (d, *J* = 7.2 Hz, 2H), 6.94 (d, *J* = 8.3 Hz, 1H), 6.88 (d, *J* = 7.9 Hz, 1H), 3.69 – 3.63 (m, 1H), 3.39 (qd, *J* = 17.0, 6.9 Hz, 2H), 3.15 (dd, *J* = 13.4, 6.1 Hz, 1H), 2.97 (dd, *J* = 13.4, 8.8 Hz, 1H), 2.63 (s, 6H). ^{13}C NMR (150 MHz, $CDCl_3$) δ = 204.4, 162.3, 145.0, 142.7, 136.4, 132.9, 129.7, 129.6, 128.5, 127.5, 127.4, 126.8, 119.3, 118.9, 118.5, 43.7, 42.6, 42.5, 37.8. DEPT-135 (150 MHz, $CDCl_3$) δ = 136.6, 129.9, 129.8, 128.6, 127.7, 127.6, 126.9, 119.0, 118.6, 43.8 (neg.), 42.8 (neg.), 42.7, 38.0. HRMS (ESI) m/z calcd for : $C_{24}H_{24}NO_4S^-$ (M-H)$^-$ 422.1426, found 422.1435.

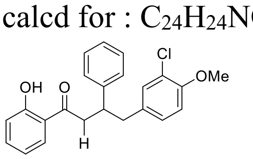

4-(3-chloro-4-methoxyphenyl)-1-(2-hydroxyphenyl)-3-phenylbutan-1-one (37):

Yield: 68%; 129.4 mg; yellow oil; TLC (nHex:EtOAc, 50:1 v/v): $R_f = 0.3$; ^{1}H NMR (600 MHz, $CDCl_3$) δ = 12.22 (s, 1H), 7.67 – 7.62 (m, 1H), 7.43 – 7.38 (m, 1H), 7.24 (t, *J* = 7.5 Hz, 2H), 7.19 – 7.13 (m, 3H), 7.07 (s, 1H), 6.92 (d, *J* = 8.4 Hz, 1H), 6.88 – 6.85 (m, 1H), 6.84 (d, *J* = 7.2 Hz, 1H), 6.73 (d, *J* = 8.4 Hz, 1H), 3.80 (s, 3H), 3.58 (p, *J* = 7.2 Hz, 1H), 3.30 (qd, *J* = 16.7, 6.9 Hz, 2H), 2.88 (ddd, *J* = 21.6, 13.7, 7.5 Hz, 2H). ^{13}C NMR (150 MHz, $CDCl_3$) δ = 204.8, 162.3, 153.3, 143.2, 136.3, 132.6, 130.8, 129.7, 128.4, 128.3, 127.5, 126.6, 121.8, 119.4, 118.8,

118.4, 111.7, 56.0, 43.4, 42.9, 41.7. DEPT-135 (150 MHz, $CDCl_3$) δ = 136.4, 130.9, 129.9, 128.6, 128.5, 127.6, 126.8, 118.9, 118.6, 111.9, 56.1, 43.6 (neg.), 43.1, 41.8 (neg.). HRMS (ESI) m/z calcd for : $C_{23}H_{20}ClO_3^-$ $(M\text{-}H)^-$ 379.1101, found 379.1105.

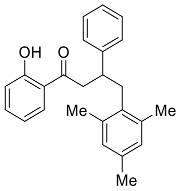

1-(2-hydroxyphenyl)-4-mesityl-3-phenylbutan-1-one (38):
Yield: 43%; 77.6 mg; yellow oil; TLC (nHex:EtOAc, 200:1 v/v): R_f = 0.3; ^{1}H NMR (600 MHz, $CDCl_3$) δ = 12.17 (s, 1H), 7.62 – 7.56 (m, 1H), 7.40 – 7.35 (m, 1H), 7.24 (t, *J* = 7.5 Hz, 2H), 7.17 (t, *J* = 6.9 Hz, 3H), 6.89 (d, *J* = 8.3 Hz, 1H), 6.82 – 6.76 (m, 3H), 3.62 – 3.54 (m, 1H), 3.45 (dd, *J* = 16.0, 8.8 Hz, 1H), 3.25 (dd, *J* = 16.0, 5.3 Hz, 1H), 3.00 (dd, *J* = 13.9, 8.3 Hz, 1H), 2.86 (dd, *J* = 13.9, 7.1 Hz, 1H), 2.21 (s, 3H), 2.18 (s, 6H). ^{13}C NMR (150 MHz, $CDCl_3$) δ = 205.0, 162.4, 144.1, 136.7, 136.1, 135.5, 133.2, 129.7, 129.1, 128.4, 127.3, 126.5, 119.3, 118.7, 118.4, 42.7, 41.5, 36.8, 20.7, 20.2. DEPT-135 (150 MHz, $CDCl_3$) δ = 136.3, 129.9, 129.3, 128.6, 127.5, 126.7, 118.9, 118.6, 42.9 (neg.), 41.7, 37.0 (neg.), 20.9, 20.3. HRMS (ESI) m/z calcd for : $C_{25}H_{25}O_2^-$ $(M\text{-}H)^-$ 357.1855, found 357.1856.

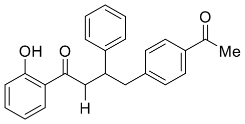

4-(4-acetylphenyl)-1-(2-hydroxyphenyl)-3-phenylbutan-1-one (39):
Yield: 55%; 99.1 mg; yellow oil; TLC (nHex:EtOAc, 10:1 v/v): R_f = 0.3; ^{1}H NMR (600 MHz, $CDCl_3$) δ = 12.21 (s, 1H), 7.78 (d, *J* = 8.2 Hz, 2H), 7.67 (dd, *J* = 8.0, 1.2 Hz, 1H), 7.45 – 7.39 (m, 1H), 7.24 (t, *J* = 7.5 Hz, 2H), 7.18 – 7.11 (m, 5H), 6.93 (d, *J* = 8.2 Hz, 1H), 6.84 (t, *J* = 7.6 Hz, 1H), 3.67 (s, 1H), 3.37 (d, *J* = 7.0 Hz, 1H), 3.32 (d, *J* = 6.9 Hz, 1H), 3.08 (d, *J* = 6.4 Hz, 1H), 3.00 – 2.96 (m, 1H), 2.53 (s, 3H). ^{13}C NMR (150 MHz, $CDCl_3$) δ = 204.6, 197.8, 162.4, 145.3, 142.9, 136.3, 135.2, 129.7, 129.3, 128.5, 128.3, 127.5, 126.7, 119.4, 118.8, 118.5, 43.7, 42.7, 42.6, 26.5. DEPT-135 (150 MHz, $CDCl_3$) δ = 136.5, 129.8, 129.5, 128.6, 128.4, 127.6, 126.8, 118.9, 118.6, 43.8 (neg.), 42.8 (neg.), 42.7, 26.6. HRMS (ESI) m/z calcd for : $C_{24}H_{21}O_3^-$ $(M\text{-}H)^-$ 357.1491, found 357.1492.

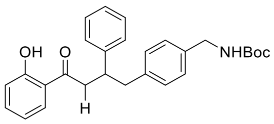

tert-butyl (4-(4-(2-hydroxyphenyl)-4-oxo-2-phenylbutyl)benzyl)carbamate (40):
Yield: 57%; 127.8 mg; yellow oil; TLC (nHex:EtOAc, 8:1 v/v): R_f = 0.3; ^{1}H NMR (600 MHz, $CDCl_3$) δ = 12.19 (s, 1H), 7.64 (d, *J* = 7.9 Hz, 1H), 7.41 (s, 1H), 7.24 (d, *J* = 7.1 Hz, 2H), 7.18 (d, *J* = 8.0 Hz, 3H), 7.11 (d, *J* = 7.6 Hz, 2H), 7.03 (d, *J* = 7.8 Hz, 2H), 6.92 (d, *J* = 8.3 Hz, 1H), 6.83 (t, *J* = 7.5 Hz, 1H), 4.92 – 4.75 (m, 1H), 4.23 (d, *J* = 5.2 Hz, 2H), 3.64 (s, 1H), 3.30 (dd, *J* = 11.3, 7.0 Hz, 2H), 2.96 (dd, *J* = 7.2, 2.0 Hz, 2H), 1.45 (s, 9H). ^{13}C NMR (150 MHz, $CDCl_3$) δ = 204.8, 162.3, 155.8, 143.6, 138.6, 136.7, 136.2, 129.7, 129.4, 128.4, 127.5, 127.4, 126.6, 119.5, 118.8, 118.4, 79.4, 44.3, 43.5, 43.0, 42.6, 28.4. DEPT-135 (150 MHz, $CDCl_3$) δ = 136.3, 129.8, 129.5, 128.5, 127.6, 127.5, 126.7, 118.9, 118.5, 44.4 (neg.), 43.6 (neg.), 43.1, 42.7 (neg.), 28.5. HRMS (ESI) m/z calcd for : $C_{28}H_{30}NO_4^-$ $(M\text{-}H)^-$ 444.2175, found 444.2201.

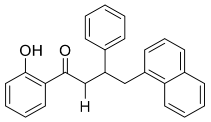

1-(2-hydroxyphenyl)-4-(naphthalen-1-yl)-3-phenylbutan-1-one (41):
Yield: 65%; 118.2 mg; yellow oil; TLC (nHex:EtOAc, 100:1 v/v): R_f = 0.3; ^{1}H NMR (600 MHz, $CDCl_3$) δ = 12.20 (s, 1H), 8.18 (d, *J* = 8.0 Hz, 1H), 7.81 (d, *J* = 8.1 Hz, 1H), 7.65 (d, *J* = 8.1 Hz, 1H), 7.52 (d, *J* = 7.7 Hz, 2H), 7.46 (t, *J* = 7.4 Hz, 1H), 7.34 (t, *J* = 7.8 Hz, 1H), 7.25 – 7.19 (m, 3H), 7.15 (d, *J* = 7.5 Hz, 3H), 7.05 (d, *J* = 6.9 Hz, 1H), 6.89 (d, *J* = 8.3 Hz, 1H), 6.72 (dd, *J* = 7.9, 7.3 Hz, 1H), 3.86 – 3.78 (m, 1H), 3.48 (dd, *J* = 13.7, 7.6 Hz, 1H), 3.41 – 3.28 (m, 3H). ^{13}C NMR (150 MHz, $CDCl_3$) δ = 204.9, 162.3, 143.9, 136.2, 135.5, 133.8, 132.0, 129.7, 128.8, 128.4, 127.5, 127.4, 127.1, 126.6, 126.0, 125.5, 125.1, 123.8, 119.3, 118.7, 118.4, 43.9, 41.9, 40.4. DEPT-135 (150 MHz, $CDCl_3$) δ = 136.3, 129.9, 128.9, 128.6, 127.7, 127.5, 127.3, 126.8, 126.2, 125.7, 125.3, 124.0, 118.9, 118.6, 44.1 (neg.), 42.1, 40.6 (neg.). HRMS (ESI) m/z calcd for : $C_{26}H_{21}O_2^-$ $(M-H)^-$ 365.1542, found 365.1544.

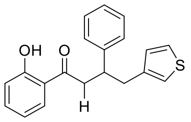

1-(2-hydroxyphenyl)-3-phenyl-4-(thiophen-3-yl)butan-1-one (42):
Yield: 67%; 108.0 mg; yellow oil; TLC (nHex:EtOAc, 100:1 v/v): R_f = 0.3; ^{1}H NMR (600 MHz, $CDCl_3$) δ = 12.23 (s, 1H), 7.65 (dd, *J* = 8.0, 1.1 Hz, 1H), 7.44 – 7.41 (m, 1H), 7.27 (t, *J* = 7.6 Hz, 2H), 7.22 – 7.16 (m, 4H), 6.93 (d, *J* = 8.4 Hz, 1H), 6.86 – 6.79 (m, 3H), 3.68 – 3.61 (m, 1H), 3.32 (dd, *J* = 11.8, 7.0 Hz, 2H), 3.02 (dd, *J* = 10.6, 7.4 Hz, 2H). ^{13}C NMR (150 MHz, $CDCl_3$) δ = 204.9, 162.4, 143.7, 139.8, 136.3, 129.8, 128.5, 128.4, 127.4, 126.6, 125.3, 121.8, 119.4, 118.8, 118.5, 43.7, 42.3, 37.2. DEPT-135 (150 MHz, $CDCl_3$) δ = 136.3, 129.9, 128.6, 128.5, 127.5, 126.7, 125.4, 121.9, 118.9, 118.6, 43.7 (neg.), 42.4, 37.3 (neg.). HRMS (ESI) m/z calcd for : $C_{20}H_{17}O_2S^-$ $(M-H)^-$ 321.0949, found 321.0957.

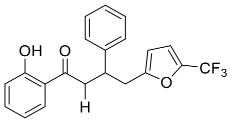

1-(2-hydroxyphenyl)-3-phenyl-4-(5-(trifluoromethyl)furan-2-yl)butan-1-one (43):
Yield: 60%; 111.9 mg; yellow oil; TLC (nHex:EtOAc, 80:1 v/v): R_f = 0.3; ^{1}H NMR (600 MHz, $CDCl_3$) δ = 12.16 (s, 1H), 7.68 (dd, *J* = 8.0, 1.1 Hz, 1H), 7.45 – 7.40 (m, 1H), 7.27 (dd, *J* = 9.7, 5.5 Hz, 2H), 7.19 (t, *J* = 6.3 Hz, 3H), 6.93 (d, *J* = 8.3 Hz, 1H), 6.85 (t, *J* = 7.6 Hz, 1H), 6.58 (d, *J* = 2.0 Hz, 1H), 5.92 (d, *J* = 3.0 Hz, 1H), 3.83 – 3.75 (m, 1H), 3.36 (dd, *J* = 6.6, 5.8 Hz, 2H), 3.06 (ddd, *J* = 44.3, 15.2, 7.4 Hz, 2H). ^{13}C NMR (150 MHz, $CDCl_3$) δ = 204.2, 162.5, 156.5, 142.7, 140.5, 136.4, 129.7, 128.6, 127.2, 127.0, 120.0, 119.3, 118.9, 118.5, 118.2, 112.3, 107.9, 43.7, 40.0, 34.7. DEPT-135 (150 MHz, $CDCl_3$) δ = 136.5, 129.8, 128.7, 127.3, 127.0, 119.0, 118.6, 112.4, 108.0, 43.8 (neg.), 40.1, 34.8 (neg.). ^{19}F NMR (564 MHz, $CDCl_3$) δ = -63.89. HRMS (ESI) m/z calcd for : $C_{21}H_{16}F_3O_3^-$ $(M-H)^-$ 373.1052, found 373.1060.

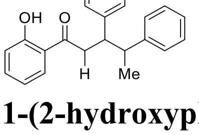

1-(2-hydroxyphenyl)-3,4-diphenylpentan-1-one (44):

Yield: 59%; 97.6 mg; yellow oil; TLC (nHex:EtOAc, 200:1 v/v): $R_f = 0.3$; ^{1}H NMR (600 MHz, $CDCl_3$) δ = 12.12 (s, 1H), 12.01 (s, 1H), 7.55 (d, J = 7.9 Hz, 1H), 7.35 – 7.29 (m, 2H), 7.24 – 7.15 (m, 9H), 7.13 – 7.02 (m, 8H), 6.91 (t, J = 7.2 Hz, 4H), 6.82 (d, J = 8.3 Hz, 1H), 6.73 (t, J = 8.1 Hz, 2H), 6.64 (t, J = 7.5 Hz, 1H), 3.56 (d, J = 7.8 Hz, 1H), 3.39 (d, J = 3.5 Hz, 1H), 3.27 (dd, J = 13.1, 7.0 Hz, 2H), 3.13 (dd, J = 16.2, 10.3 Hz, 1H), 3.09 – 3.03 (m, 1H), 2.94 – 2.85 (m, 2H), 1.23 (d, J = 7.1 Hz, 3H), 0.95 (d, J = 6.9 Hz, 3H). ^{13}C NMR (150 MHz, $CDCl_3$) δ = 205.1, 205.1, 162.4, 162.2, 145.5, 143.8, 142.8, 141.8, 136.2, 135.9, 129.7, 129.6, 128.7, 128.4, 128.3, 128.2, 128.1, 127.9, 127.8, 127.6, 126.6, 126.5, 126.3, 126.2, 119.4, 119.3, 118.7, 118.5, 118.4, 118.2, 48.6, 47.2, 45.9, 44.7, 43.5, 40.4, 20.8, 18.1. DEPT-135 (150 MHz, $CDCl_3$) δ = 136.3, 136.1, 129.9, 129.8, 128.8, 128.6, 128.5, 128.3, 128.2, 128.0, 127.9, 127.7, 126.8, 126.7, 126.5, 126.4, 118.9, 118.6, 118.5, 118.4, 48.7, 47.4, 46.0, 44.8, 43.7 (neg.), 40.5 (neg.), 21.0, 18.3. HRMS (ESI) m/z calcd for : $C_{23}H_{21}O_2^-$ $(M-H)^-$ 329.1542, found 329.1543.

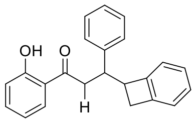

3-(bicyclo[4.2.0]octa-1(6),2,4-trien-7-yl)-1-(2-hydroxyphenyl)-3-phenylpropan-1-one (45):

Yield: 61%; 100.2 mg; yellow oil; TLC (nHex:EtOAc, 100:1 v/v): $R_f = 0.3$; ^{1}H NMR (600 MHz, $CDCl_3$) δ = 12.23 (s, 1H), 12.17 (s, 1H), 7.72 (dd, J = 19.1, 8.0 Hz, 2H), 7.40 (d, J = 8.4 Hz, 2H), 7.29 (s, 8H), 7.23 (dd, J = 10.0, 7.6 Hz, 3H), 7.17 (s, 2H), 7.13 (d, J = 7.4 Hz, 1H), 7.08 (d, J = 6.8 Hz, 1H), 7.03 (d, J = 7.2 Hz, 1H), 6.98 (s, 1H), 6.91 (dd, J = 14.5, 8.4 Hz, 2H), 6.87 – 6.76 (m, 2H), 6.24 (d, J = 7.2 Hz, 1H), 3.83 (s, 2H), 3.62 – 3.47 (m, 5H), 3.42 – 3.28 (m, 2H), 3.11 (dd, J = 14.4, 5.1 Hz, 1H), 2.91 (d, J = 14.0 Hz, 1H), 2.75 (d, J = 14.4 Hz, 1H). ^{13}C NMR (150 MHz, $CDCl_3$) δ = 204.9, 204.6, 162.4, 162.3, 147.6, 147.2, 143.8, 143.1, 143.0, 142.7, 136.3, 136.2, 129.9, 129.8, 128.5, 128.4, 127.9, 127.8, 127.7, 127.6, 126.8, 126.7, 126.5, 123.3, 122.8, 122.6, 122.2, 119.5, 118.8, 118.5, 118.4, 48.6, 48.5, 45.8, 43.3, 43.2, 35.5, 34.9. DEPT-135 (150 MHz, $CDCl_3$) δ = 136.4, 136.3, 130.0, 129.9, 128.6, 128.5, 128.1, 127.9, 127.8, 127.7, 126.9, 126.8, 126.6, 123.4, 122.9, 122.7, 122.3, 118.9, 118.6, 118.5, 48.7, 48.6, 45.9, 43.5 (neg.), 43.3 (neg.), 35.7 (neg.), 35.0 (neg.). HRMS (ESI) m/z calcd for : $C_{23}H_{19}O_2^-$ $(M-H)^-$ 327.1385, found 327.1389.

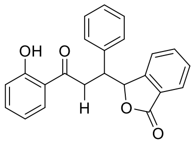

3-(3-(2-hydroxyphenyl)-3-oxo-1-phenylpropyl)isobenzofuran-1(3H)-one (46):

Yield: 64%; 115.3 mg; yellow oil; TLC (nHex:EtOAc, 8:1 v/v): $R_f = 0.3$; ^{1}H NMR (600 MHz, $CDCl_3$) δ = 12.13 (s, 2H), 11.96 (s, 1H), 7.86 (dd, J = 8.1, 1.2 Hz, 3H), 7.72 – 7.67 (m, 1H), 7.64 – 7.58 (m, 4H), 7.53 – 7.46 (m, 6H), 7.37 (ddd, J = 14.2, 10.5, 4.6 Hz, 8H), 7.09 – 7.03 (m, 10H), 6.97 (d, J = 8.0 Hz, 2H), 6.93 (t, J = 7.6 Hz, 2H), 6.89 – 6.83 (m, 2H), 6.68 – 6.63 (m, 1H), 5.85 (d, J = 2.5 Hz, 2H), 5.70 (d, J = 7.3 Hz, 1H), 4.14 (s, 2H), 3.92 (dd, J = 17.9, 7.8 Hz, 2H), 3.75 (ddd, J = 10.7, 7.4, 3.7 Hz, 1H), 3.64 (dd, J = 18.0, 6.0 Hz, 3H), 3.51 (dd, J = 17.1, 3.7 Hz, 1H). ^{13}C NMR (150 MHz, $CDCl_3$) δ = 203.8, 203.2, 170.2, 170.0, 162.3, 162.2, 148.0, 147.9, 139.2, 136.7, 136.4, 136.0, 133.8, 133.7, 129.8, 129.6, 129.5, 129.0, 128.9, 128.8, 128.7, 128.1, 127.8, 127.3, 126.2, 126.1, 125.6, 125.3, 122.9, 122.2, 119.3, 119.2, 118.9, 118.5,

118.4, 83.3, 82.5, 45.7, 43.6, 40.0, 39.9. DEPT-135 (150 MHz, $CDCl_3$) δ = 136.9, 136.5, 133.9, 133.8, 129.9, 129.8, 129.6, 129.1, 129.0, 128.9, 128.8, 128.2, 128.0, 127.4, 125.7, 125.5, 123.0, 122.3, 119.3, 119.0, 118.6, 118.5, 83.5, 82.6, 45.8, 43.7, 40.2 (neg.), 40.0 (neg.). HRMS (ESI) m/z calcd for : $C_{23}H_{17}O_4^-$ (M-H)$^-$ 357.1127, found 357.1145.

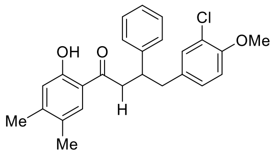

4-(3-chloro-4-methoxyphenyl)-1-(2-hydroxy-4,5-dimethylphenyl)-3-phenylbutan-1-one (47):

Yield: 69%; 141.7 mg; yellow oil; TLC (nHex:EtOAc, 50:1 v/v): R_f = 0.3; ^{1}H NMR (600 MHz, $CDCl_3$) δ = 12.08 (s, 1H), 7.32 (s, 1H), 7.24 (t, *J* = 7.5 Hz, 2H), 7.16 (dd, *J* = 12.4, 7.2 Hz, 3H), 7.07 (d, *J* = 2.0 Hz, 1H), 6.88 (dd, *J* = 8.4, 2.0 Hz, 1H), 6.75 – 6.70 (m, 2H), 3.80 (s, 3H), 3.60 – 3.51 (m, 1H), 3.25 (d, *J* = 7.0 Hz, 2H), 2.88 (ddd, *J* = 21.6, 13.7, 7.5 Hz, 2H), 2.20 (s, 3H), 2.15 (s, 3H). ^{13}C NMR (150 MHz, $CDCl_3$) δ = 204.1, 160.7, 153.3, 146.8, 143.4, 132.8, 130.8, 129.8, 128.4, 128.3, 127.5, 127.0, 126.6, 121.8, 118.9, 117.3, 111.7, 55.9, 43.4, 43.2, 41.7, 20.4, 18.8. DEPT-135 (150 MHz, $CDCl_3$) δ = 130.9, 130.0, 128.5, 128.4, 127.6, 126.7, 119.0, 111.9, 56.1, 43.5 (neg.), 43.3, 41.8 (neg.), 20.5, 19.0. HRMS (ESI) m/z calcd for : $C_{25}H_{24}ClO_3^-$ (M-H)$^-$ 407.1414, found 407.1415.

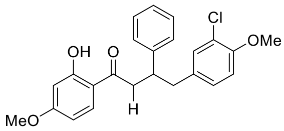

4-(3-chloro-4-methoxyphenyl)-1-(2-hydroxy-4-methoxyphenyl)-3-phenylbutan-1-one (48):

Yield: 57%; 117.0 mg; yellow oil; TLC (nHex:EtOAc, 10:1 v/v): R_f = 0.3; ^{1}H NMR (600 MHz, $CDCl_3$) δ = 12.74 (s, 1H), 7.53 (d, *J* = 8.7 Hz, 1H), 7.23 (t, *J* = 7.3 Hz, 2H), 7.15 (dd, *J* = 15.6, 7.4 Hz, 3H), 7.05 (s, 1H), 6.86 (d, *J* = 7.8 Hz, 1H), 6.72 (d, *J* = 8.3 Hz, 1H), 6.36 (d, *J* = 10.0 Hz, 2H), 3.78 (d, *J* = 17.4 Hz, 6H), 3.60 – 3.52 (m, 1H), 3.26 – 3.13 (m, 2H), 2.87 (ddd, *J* = 21.6, 13.7, 7.5 Hz, 2H). ^{13}C NMR (150 MHz, $CDCl_3$) δ = 202.8, 165.9, 165.3, 153.2, 143.3, 132.7, 131.3, 130.7, 128.4, 128.3, 127.4, 126.5, 121.8, 113.6, 111.7, 107.5, 100.8, 55.9, 55.4, 43.2, 43.1, 41.6. DEPT-135 (150 MHz, $CDCl_3$) δ = 131.5, 130.9, 128.5, 128.4, 127.6, 126.7, 111.8, 107.7, 100.9, 56.1, 55.6, 43.3, 43.2 (neg.), 41.8 (neg). HRMS (ESI) m/z calcd for : $C_{24}H_{22}ClO_4^-$ (M-H)$^-$ 409.1207, found 409.1206.

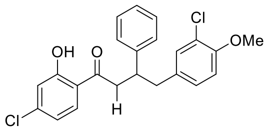

1-(4-chloro-2-hydroxyphenyl)-4-(3-chloro-4-methoxyphenyl)-3-phenylbutan-1-one (49):

Yield: 73%; 150.5 mg; yellow oil; TLC (nHex:EtOAc, 50:1 v/v): R_f = 0.3; ^{1}H NMR (600 MHz, $CDCl_3$) δ = 12.33 (s, 1H), 7.54 (d, *J* = 8.6 Hz, 1H), 7.24 (t, *J* = 7.5 Hz, 2H), 7.15 (dd, *J* = 23.9, 7.3 Hz, 3H), 7.06 (d, *J* = 1.9 Hz, 1H), 6.92 (d, *J* = 2.0 Hz, 1H), 6.87 (dd, *J* = 8.4, 1.9 Hz, 1H), 6.79 (dd, *J* = 8.6, 2.0 Hz, 1H), 6.73 (d, *J* = 8.4 Hz, 1H), 3.80 (s, 3H), 3.56 (p, *J* = 7.2 Hz, 1H), 3.24 (qd, *J* = 16.6, 6.9 Hz, 2H), 2.92 – 2.82 (m, 2H). ^{13}C NMR (150 MHz, $CDCl_3$) δ = 204.1,

163.0, 153.3, 143.0, 141.9, 132.5, 130.8, 130.7, 128.5, 128.3, 127.4, 126.7, 121.9, 119.4, 118.4, 117.9, 111.7, 56.0, 43.5, 43.0, 41.6. DEPT-135 (150 MHz, $CDCl_3$) δ = 130.9, 130.8, 128.6, 128.4, 127.6, 126.9, 119.6, 118.6, 111.9, 56.1, 43.6 (neg.), 43.2, 41.8 (neg.). HRMS (ESI) m/z calcd for : $C_{23}H_{19}Cl_2O_3^-$ (M-H)$^-$ 413.0711, found 413.0712.

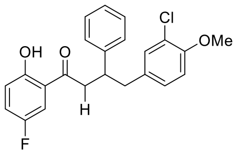

4-(3-chloro-4-methoxyphenyl)-1-(5-fluoro-2-hydroxyphenyl)-3-phenylbutan-1-one (50):
Yield: 65%; 130.0 mg; white solid; m.p. 123-125 °C; TLC (nHex:EtOAc, 50:1 v/v): R_f = 0.3; ^{1}H NMR (600 MHz, $CDCl_3$) δ = 11.92 (s, 1H), 7.30 – 7.23 (m, 3H), 7.16 (dd, *J* = 17.1, 7.6 Hz, 4H), 7.08 (d, *J* = 1.8 Hz, 1H), 6.88 (dd, *J* = 8.7, 3.8 Hz, 2H), 6.74 (d, *J* = 8.4 Hz, 1H), 3.81 (s, 3H), 3.57 (s, 1H), 3.24 (dd, *J* = 16.6, 6.9 Hz, 2H), 2.88 (t, *J* = 6.9 Hz, 2H). ^{13}C NMR (150 MHz, $CDCl_3$) δ = 203.9, 158.5, 155.4, 153.8, 153.4, 143.0, 132.5, 130.8, 128.5, 128.3, 127.4, 126.7, 123.8, 121.9, 119.7, 118.8, 114.6, 111.8, 56.0, 43.5, 42.9, 41.7. DEPT-135 (150 MHz, $CDCl_3$) δ = 130.9, 128.6, 128.4, 127.6, 126.9, 124.0, 119.9, 114.7, 111.9, 56.1, 43.6 (neg.), 43.0, 41.8 (neg.). ^{19}F NMR (564 MHz, $CDCl_3$) δ = -123.74. HRMS (ESI) m/z calcd for : $C_{23}H_{19}ClFO_3^-$ (M-H)$^-$ 397.1007, found 397.1007.

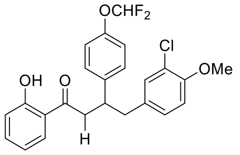

4-(3-chloro-4-methoxyphenyl)-3-(4-(difluoromethoxy)phenyl)-1-(2-hydroxyphenyl)butan-1-one (51):
Yield: 69%; 153.4 mg; yellow oil; TLC (nHex:EtOAc, 12:1 v/v): R_f = 0.3; ^{1}H NMR (600 MHz, $CDCl_3$) δ = 12.17 (s, 1H), 7.65 (dd, *J* = 8.1, 1.4 Hz, 1H), 7.44 – 7.38 (m, 1H), 7.13 (d, *J* = 8.6 Hz, 2H), 7.06 (d, *J* = 2.1 Hz, 1H), 6.99 (d, *J* = 8.5 Hz, 2H), 6.94 – 6.90 (m, 1H), 6.88 – 6.82 (m, 2H), 6.75 (d, *J* = 8.4 Hz, 1H), 6.44 (t, *J* = 74.1 Hz, 1H), 3.81 (s, 3H), 3.63 – 3.55 (m, 1H), 3.34 – 3.24 (m, 2H), 2.86 (ddd, *J* = 21.8, 13.7, 7.5 Hz, 2H). ^{13}C NMR (150 MHz, $CDCl_3$) δ = 204.4, 162.3, 153.4, 149.7, 140.5, 136.4, 132.3, 130.7, 129.6, 128.8, 128.3, 121.9, 119.4, 119.3, 118.8, 118.5, 115.9, 111.8, 55.9, 43.4, 42.2, 41.7. DEPT-135 (150 MHz, $CDCl_3$) δ = 136.5, 130.8, 129.8, 129.0, 128.4, 119.5, 119.0, 118.6, 116.1, 111.9, 56.1, 43.5 (neg.), 42.3, 41.8 (neg.). ^{19}F NMR (564 MHz, $CDCl_3$) δ = -80.42. HRMS (ESI) m/z calcd for : $C_{24}H_{20}ClF_2O_4^-$ (M-H)$^-$ 445.1018, found 445.1021.

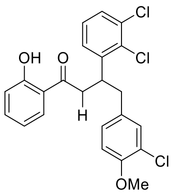

4-(3-chloro-4-methoxyphenyl)-3-(2,3-dichlorophenyl)-1-(2-hydroxyphenyl)butan-1-one (52):
Yield: 75%; 168.2 mg; yellow oil; TLC (nHex:EtOAc, 25:1 v/v): R_f = 0.3; ^{1}H NMR (600 MHz, $CDCl_3$) δ = 12.08 (s, 1H), 7.67 (d, *J* = 7.9 Hz, 1H), 7.43 (t, *J* = 7.6 Hz, 1H), 7.31 (d, *J* = 7.3 Hz, 1H), 7.14 – 7.08 (m, 3H), 6.94 (dd, *J* = 20.7, 8.3 Hz, 2H), 6.85 (t, *J* = 7.6 Hz, 1H), 6.77 (d,

J = 8.4 Hz, 1H), 4.32 – 4.19 (m, 1H), 3.83 (s, 3H), 3.40 – 3.25 (m, 2H), 2.89 (ddd, J = 71.9, 13.9, 7.4 Hz, 2H). ^{13}C NMR (150 MHz, $CDCl_3$) δ = 203.9, 162.3, 153.5, 143.0, 136.4, 133.5, 132.2, 131.7, 130.8, 129.6, 128.6, 128.3, 127.2, 125.7, 122.0, 119.2, 118.9, 118.5, 111.9, 56.0, 41.7, 39.8, 39.5. DEPT-135 (150 MHz, $CDCl_3$) δ = 136.6, 130.9, 129.7, 128.8, 128.4, 127.3, 125.9, 119.0, 118.6, 112.0, 56.1, 41.8 (neg.), 39.9 (neg.), 39.6. HRMS (ESI) m/z calcd for : $C_{23}H_{18}Cl_3O_3^-$ (M-H)$^-$ 447.0322, found 447.0323.

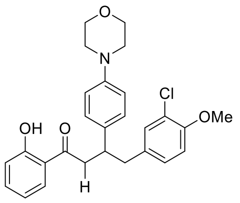

4-(3-chloro-4-methoxyphenyl)-1-(2-hydroxyphenyl)-3-(4-morpholinophenyl)butan-1-one (53):

Yield: 67%; 155.6 mg; yellow oil; TLC (nHex:EtOAc, 5:1 v/v): R_f = 0.3; ^{1}H NMR (600 MHz, $CDCl_3$) δ = 12.23 (s, 1H), 7.65 (dd, J = 8.1, 1.4 Hz, 1H), 7.45 – 7.40 (m, 1H), 7.07 (dd, J = 8.8, 5.4 Hz, 3H), 6.93 (dd, J = 8.4, 0.8 Hz, 1H), 6.90 (dd, J = 8.4, 2.0 Hz, 1H), 6.86 – 6.83 (m, 1H), 6.81 (d, J = 8.7 Hz, 2H), 6.76 (d, J = 8.4 Hz, 1H), 3.85 – 3.82 (m, 7H), 3.56 – 3.49 (m, 1H), 3.26 (qd, J = 16.5, 7.0 Hz, 2H), 3.13 – 3.08 (m, 4H), 2.86 (qd, J = 13.7, 7.5 Hz, 2H). ^{13}C NMR (150 MHz, $CDCl_3$) δ = 205.0, 162.4, 153.3, 149.8, 136.2, 134.7, 132.9, 130.8, 129.8, 128.3, 128.2, 121.9, 119.5, 118.8, 118.5, 115.7, 111.8, 66.9, 56.1, 49.3, 43.7, 42.2, 41.8. DEPT-135 (150 MHz, $CDCl_3$) δ = 136.3, 130.9, 129.9, 128.4, 128.2, 118.9, 118.5, 115.8, 111.8, 66.9 (neg.), 56.1, 49.4 (neg.), 43.8 (neg.), 42.3, 41.9 (neg.). HRMS (ESI) m/z calcd for : $C_{27}H_{29}ClNO_4^+$ (M+H)$^+$ 466.1785, found 466.1786.

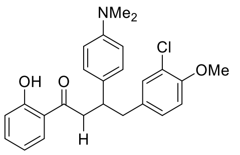

4-(3-chloro-4-methoxyphenyl)-3-(4-(dimethylamino)phenyl)-1-(2-hydroxyphenyl)butan-1-one (54):

Yield: 82%; 173.4 mg; yellow oil; TLC (nHex:EtOAc, 12:1 v/v): R_f = 0.3; ^{1}H NMR (600 MHz, $CDCl_3$) δ = 12.27 (s, 1H), 7.64 (dd, J = 8.0, 1.4 Hz, 1H), 7.43 – 7.38 (m, 1H), 7.10 (d, J = 2.0 Hz, 1H), 7.02 (d, J = 8.7 Hz, 2H), 6.93 – 6.88 (m, 2H), 6.85 – 6.81 (m, 1H), 6.74 (d, J = 8.4 Hz, 1H), 6.63 (t, J = 5.9 Hz, 2H), 3.82 (s, 3H), 3.54 – 3.46 (m, 1H), 3.24 (qd, J = 16.4, 7.0 Hz, 2H), 2.88 (s, 6H), 2.85 (dd, J = 7.4, 3.7 Hz, 2H). ^{13}C NMR (150 MHz, $CDCl_3$) δ = 205.3, 162.4, 153.2, 149.3, 136.1, 133.1, 131.1, 130.8, 129.8, 128.4, 128.0, 121.8, 119.5, 118.7, 118.4, 112.7, 111.8, 56.0, 43.9, 42.2, 41.9, 40.6. DEPT-135, 150 MHz, $CDCl_3$) δ = 136.3, 130.9, 130.0, 128.5, 128.1, 118.9, 118.5, 112.8, 111.9, 56.1, 44.0 (neg.), 42.3, 42.0 (neg.), 40.7. HRMS (ESI) m/z calcd for : $C_{25}H_{25}ClNO_3^-$ (M-H)$^-$ 422.1523, found 422.1522.

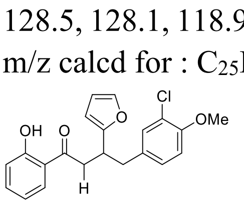

4-(3-chloro-4-methoxyphenyl)-3-(furan-2-yl)-1-(2-hydroxyphenyl)butan-1-one (55):

Yield: 69%; 126.8 mg; yellow oil; TLC (nHex:EtOAc, 25:1 v/v): R_f = 0.3; ^{1}H NMR (600 MHz, $CDCl_3$) δ = 12.21 (s, 1H), 7.67 (dd, J = 8.0, 1.1 Hz, 1H), 7.44 (s, 1H), 7.31 (d, J = 1.0 Hz, 1H),

7.05 (d, J = 2.0 Hz, 1H), 6.95 (d, J = 8.3 Hz, 1H), 6.91 – 6.83 (m, 2H), 6.77 (d, J = 8.4 Hz, 1H), 6.22 (dd, J = 3.0, 1.9 Hz, 1H), 5.91 (d, J = 3.1 Hz, 1H), 3.84 (s, 3H), 3.76 – 3.68 (m, 1H), 3.28 (ddd, J = 95.7, 16.7, 6.9 Hz, 2H), 2.93 (ddd, J = 20.4, 13.7, 7.3 Hz, 2H). ^{13}C NMR (150 MHz, $CDCl_3$) δ = 204.4, 162.4, 155.9, 153.5, 141.2, 136.4, 132.2, 130.7, 129.8, 128.2, 122.0, 119.4, 118.9, 118.5, 111.8, 110.1, 106.1, 56.0, 41.1, 38.9, 36.4. DEPT-135 (150 MHz, $CDCl_3$) δ = 141.3, 136.5, 130.8, 129.9, 128.3, 119.0, 118.6, 111.9, 110.2, 106.1, 56.1, 41.1 (neg.), 39.0 (neg.), 36.5. HRMS (ESI) m/z calcd for : $C_{21}H_{18}ClO_4^-$ (M-H)$^-$ 369.0894, found 369.0895.

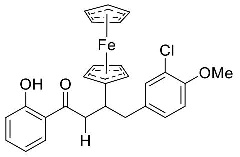

3-Ferrocenyl-4-(3-chloro-4-methoxyphenyl)-1-(2-hydroxyphenyl)butan-1-one (56):
Yield: 61%; 148.6 mg; yellow solid; m.p. 129-132 °C; TLC (nHex:EtOAc, 80:1 v/v): R_f = 0.3; ^{1}H NMR (600 MHz, $CDCl_3$) δ = 12.36 (s, 1H), 7.63 (dd, J = 8.0, 1.2 Hz, 1H), 7.45 (s, 1H), 7.07 (d, J = 2.0 Hz, 1H), 6.97 (d, J = 8.3 Hz, 1H), 6.91 (dd, J = 8.3, 2.0 Hz, 1H), 6.86 (s, 1H), 6.77 (d, J = 8.4 Hz, 1H), 4.12 – 4.03 (m, 7H), 3.98 (d, J = 1.0 Hz, 1H), 3.93 (s, 1H), 3.84 (s, 3H), 3.45 (t, J = 6.7 Hz, 1H), 3.19 (dd, J = 13.2, 6.5 Hz, 2H), 2.93 (dd, J = 13.6, 6.0 Hz, 1H), 2.73 (dd, J = 13.6, 8.0 Hz, 1H). ^{13}C NMR (150 MHz, $CDCl_3$) δ = 205.5, 162.4, 153.4, 136.3, 132.7, 131.1, 129.8, 128.6, 121.9, 119.6, 118.8, 118.5, 111.8, 92.4, 68.4, 67.3, 67.3, 67.2, 66.7, 56.1, 43.0, 41.4, 36.5. DEPT-135 (150 MHz, $CDCl_3$) δ = 136.4, 131.2, 129.9, 128.7, 118.9, 118.6, 111.9, 68.5, 67.4, 67.4, 67.3, 66.8, 56.2, 43.1 (neg.), 41.5 (neg.), 36.6. HRMS (ESI) m/z calcd for : $C_{27}H_{24}ClFeO_3^-$ (M-H)$^-$ 487.0763, found 487.0766.

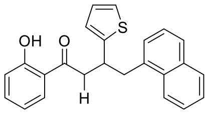

1-(2-hydroxyphenyl)-4-(naphthalen-1-yl)-3-(thiophen-2-yl)butan-1-one (57):
Yield: 71%; 132.7 mg; yellow oil; TLC (nHex:EtOAc, 100:1 v/v): R_f = 0.3; ^{1}H NMR (600 MHz, $CDCl_3$) δ = 12.18 (s, 1H), 8.17 (d, J = 8.5 Hz, 1H), 7.81 (d, J = 8.1 Hz, 1H), 7.66 (d, J = 8.2 Hz, 1H), 7.52 (d, J = 8.0 Hz, 2H), 7.45 (d, J = 7.7 Hz, 1H), 7.34 (s, 1H), 7.25 (t, J = 7.6 Hz, 1H), 7.13 (d, J = 6.9 Hz, 1H), 7.06 (d, J = 5.0 Hz, 1H), 6.89 (d, J = 8.3 Hz, 1H), 6.79 (dd, J = 4.9, 3.6 Hz, 1H), 6.72 (t, J = 7.6 Hz, 1H), 6.66 (d, J = 3.2 Hz, 1H), 4.22 – 4.14 (m, 1H), 3.50 (dd, J = 13.8, 7.6 Hz, 1H), 3.42 – 3.29 (m, 3H). ^{13}C NMR (150 MHz, $CDCl_3$) δ = 204.3, 162.3, 147.5, 136.3, 135.1, 133.8, 131.9, 129.6, 128.8, 127.5, 127.3, 126.5, 126.1, 125.5, 125.1, 124.2, 123.7, 123.1, 119.3, 118.7, 118.4, 44.9, 41.2, 37.2. DEPT-135 (150 MHz, $CDCl_3$) δ = 136.5, 129.9, 129.0, 127.7, 127.5, 126.8, 126.3, 125.8, 125.3, 124.4, 123.9, 123.4, 119.0, 118.6, 45.1 (neg.), 41.5 (neg.), 37.4. HRMS (ESI) m/z calcd for : $C_{24}H_{19}O_2S^-$ (M-H)$^-$ 371.1106, found 371.1107.

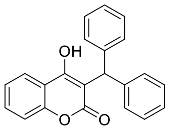

3-benzhydryl-4-hydroxy-2H-chromen-2-one (58):
Yield: 91%; 60.0 mg; white solid; m.p. 182-185 °C; TLC (nHex:EtOAc, 5:1 v/v): R_f = 0.3; ^{1}H NMR (600 MHz, $CDCl_3$) δ = 7.73 (d, J = 7.9 Hz, 1H), 7.53 (t, J = 7.8 Hz, 1H), 7.38 (t, J = 7.5

Hz, 4H), 7.32 (t, J = 7.1 Hz, 3H), 7.29 – 7.24 (m, 5H), 6.32 (s, 1H), 5.98 (s, 1H). ^{13}C NMR (150 MHz, $CDCl_3$) δ = 163.2, 160.7, 152.7, 139.9, 132.1, 129.4, 128.7, 127.8, 123.9, 123.1, 116.4, 115.9, 107.7, 47.3. DEPT-135 (150 MHz, $CDCl_3$) δ = 132.2, 129.5, 128.8, 127.8, 124.0, 123.2, 116.5, 47.4. HRMS (ESI) m/z calcd for : $C_{22}H_{15}O_3^-$ $(M-H)^-$ 327.1021, found 327.1023.

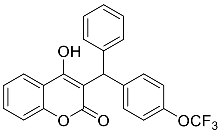

4-hydroxy-3-(phenyl(4-(trifluoromethoxy)phenyl)methyl)-2H-chromen-2-one (59):
Yield: 93%; 76.5 mg; brown solid; m.p. 146-148 °C; TLC (nHex:EtOAc, 5:1 v/v): R_f = 0.3; ^{1}H NMR (600 MHz, $CDCl_3$) δ = 7.80 (d, J = 7.8 Hz, 1H), 7.51 (d, J = 7.8 Hz, 1H), 7.36 – 7.28 (m, 5H), 7.24 (t, J = 8.3 Hz, 4H), 7.16 (d, J = 8.3 Hz, 2H), 5.98 (s, 1H). ^{13}C NMR (150 MHz, $CDCl_3$) δ = 163.5, 161.2, 152.6, 148.2, 140.0, 138.7, 132.3, 130.2, 129.3, 128.6, 127.7, 124.1, 123.2, 121.2, 119.5, 116.4, 115.8, 107.3, 46.3. DEPT-135 (150 MHz, $CDCl_3$) δ = 132.4, 130.4, 129.5, 128.7, 127.9, 124.2, 123.3, 121.3, 116.6, 46.4. ^{19}F NMR (564 MHz, $CDCl_3$) δ = -57.77. HRMS (ESI) m/z calcd for : $C_{23}H_{14}F_3O_4^-$ $(M-H)^-$ 411.0844, found 411.0853.

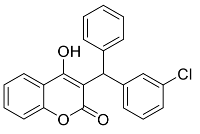

3-((3-chlorophenyl)(phenyl)methyl)-4-hydroxy-2H-chromen-2-one (60):
Yield: 88%; 63.9 mg; white solid; m.p. 171-173 °C; TLC (nHex:EtOAc, 6:1 v/v): R_f = 0.3; ^{1}H NMR (600 MHz, $CDCl_3$) δ = 7.76 (dd, J = 8.0, 1.2 Hz, 1H), 7.54 (dd, J = 11.4, 4.3 Hz, 1H), 7.38 (t, J = 7.4 Hz, 2H), 7.32 (dd, J = 17.9, 7.9 Hz, 2H), 7.28 – 7.22 (m, 6H), 7.17 (dd, J = 5.2, 2.8 Hz, 1H), 6.61 (s, 1H), 5.94 (s, 1H). ^{13}C NMR (150 MHz, $CDCl_3$) δ = 163.2, 161.0, 152.6, 141.9, 139.6, 135.0, 132.3, 130.3, 129.6, 128.8, 128.6, 128.0, 127.7, 126.9, 124.0, 123.2, 116.5, 115.7, 107.0, 46.9. DEPT-135 (150 MHz, $CDCl_3$) δ = 132.4, 130.4, 129.7, 128.9, 128.7, 128.1, 127.8, 127.1, 124.1, 123.3, 116.6, 47.0. HRMS (ESI) m/z calcd for : $C_{22}H_{14}ClO_3^-$ $(M-H)^-$ 361.0631, found 361.0632.

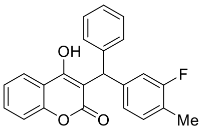

3-((3-fluoro-4-methylphenyl)(phenyl)methyl)-4-hydroxy-2H-chromen-2-one (61):
Yield: 85%; 61.0 mg; white solid; m.p. 162-164 °C; TLC (nHex:EtOAc, 5:1 v/v): R_f = 0.3; ^{1}H NMR (600 MHz, $CDCl_3$) δ = 7.74 (d, J = 7.9 Hz, 1H), 7.54 (t, J = 7.8 Hz, 1H), 7.38 (t, J = 7.5 Hz, 2H), 7.33 (t, J = 8.8 Hz, 2H), 7.26 (d, J = 7.6 Hz, 3H), 7.18 (t, J = 7.8 Hz, 1H), 6.94 (dd, J = 18.0, 9.3 Hz, 2H), 6.36 (s, 1H), 5.92 (s, 1H), 2.26 (s, 3H). ^{13}C NMR (150 MHz, $CDCl_3$) δ = 163.2, 162.6, 161.0, 160.8, 152.7, 139.8, 139.6, 132.4, 132.3, 129.6, 128.7, 128.0, 124.4, 124.1, 124.0, 123.2, 116.5, 115.9, 115.5, 107.4, 46.8, 14.3. DEPT-135 (150 MHz, $CDCl_3$) δ = 132.4, 132.3, 129.6, 128.7, 128.0, 124.1, 124.0, 123.2, 116.5, 115.5, 46.8, 14.3. ^{19}F NMR (564 MHz, $CDCl_3$) δ = -115.55. HRMS (ESI) m/z calcd for : $C_{23}H_{16}FO_3^-$ $(M-H)^-$ 359.1083, found 359.1091.

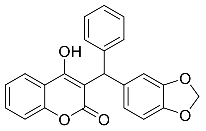

3-(benzo[d][1,3]dioxol-5-yl(phenyl)methyl)-4-hydroxy-2H-chromen-2-one (62):
Yield: 83%; 61.7 mg; yellow oil; TLC (nHex:EtOAc, 5:1 v/v): R_f = 0.3; ^{1}H NMR (600 MHz, $CDCl_3$) δ = 7.75 (s, 1H), 7.51 (s, 1H), 7.31 (d, *J* = 39.9 Hz, 7H), 6.72 (d, *J* = 56.9 Hz, 4H), 5.90 (d, *J* = 39.1 Hz, 3H). ^{13}C NMR (150 MHz, $CDCl_3$) δ = 163.5, 161.0, 152.7, 148.7, 147.3, 140.1, 133.9, 132.3, 129.4, 128.8, 127.8, 124.1, 123.2, 121.7, 116.6, 116.0, 109.4, 108.8, 107.9, 101.5, 47.0. DEPT-135 (150 MHz, $CDCl_3$) δ = 132.3, 129.4, 128.8, 127.8, 124.1, 123.2, 121.7, 116.5, 109.4, 108.8, 101.5 (neg.), 47.0. HRMS (ESI) m/z calcd for : $C_{23}H_{15}O_5^-$ $(M-H)^-$ 371.0919, found 371.0922.

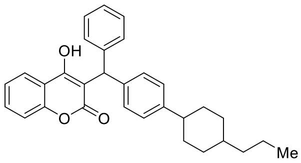

4-hydroxy-3-(phenyl(4-(4-propylcyclohexyl)phenyl)methyl)-2H-chromen-2-one (63):
Yield: 82%; 74.4 mg; white solid; m.p. 188-190 ºC; TLC (nHex:EtOAc, 8:1 v/v): R_f = 0.3; ^{1}H NMR (600 MHz, $CDCl_3$) δ = 7.73 (dd, *J* = 7.9, 1.0 Hz, 1H), 7.51 (dd, *J* = 11.4, 4.2 Hz, 1H), 7.36 (t, *J* = 7.4 Hz, 2H), 7.29 (dd, *J* = 17.3, 7.7 Hz, 4H), 7.24 (t, *J* = 5.4 Hz, 1H), 7.21 (d, *J* = 8.2 Hz, 2H), 7.17 (d, *J* = 8.2 Hz, 2H), 6.37 (s, 1H), 5.93 (s, 1H), 2.46 (t, *J* = 12.1 Hz, 1H), 1.87 (t, *J* = 14.4 Hz, 4H), 1.46 – 1.38 (m, 2H), 1.37 – 1.31 (m, 2H), 1.30 – 1.25 (m, 1H), 1.23 – 1.17 (m, 2H), 1.04 (d, *J* = 12.6 Hz, 2H), 0.89 (t, *J* = 7.3 Hz, 3H). ^{13}C NMR (150 MHz, $CDCl_3$) δ = 163.3, 160.7, 152.7, 147.7, 140.1, 137.2, 132.1, 129.3, 128.8, 128.7, 128.0, 127.7, 123.9, 123.2, 116.5, 116.0, 107.9, 47.0, 44.2, 39.7, 37.0, 34.3, 33.5, 20.1, 14.5. DEPT-135 (150 MHz, $CDCl_3$) δ = 132.1, 129.3, 128.8, 128.7, 128.0, 127.7, 123.9, 123.2, 116.5, 47.0, 44.2, 39.7 (neg.), 37.0, 34.3 (neg.), 33.5 (neg.), 20.1 (neg.), 14.5. HRMS (ESI) m/z calcd for : $C_{31}H_{31}O_3^-$ $(M-H)^-$ 451.2273, found 451.2291.

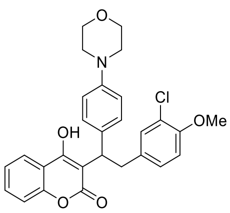

3-(2-(3-chloro-4-methoxyphenyl)-1-(4-morpholinophenyl)ethyl)-4-hydroxy-2H-chromen-2-one (64):
Yield: 74%; 72.5 mg; white solid; m.p. 189-192 ºC; TLC (nHex:EtOAc, 1:1 v/v): R_f = 0.3; ^{1}H NMR (600 MHz, $CDCl_3$) δ = 7.67 (d, *J* = 7.8 Hz, 1H), 7.50 (s, 1H), 7.39 (d, *J* = 8.4 Hz, 2H), 7.26 (d, *J* = 3.2 Hz, 2H), 7.23 (d, *J* = 7.7 Hz, 1H), 7.11 (d, *J* = 8.2 Hz, 1H), 6.89 (d, *J* = 8.5 Hz, 2H), 6.75 (d, *J* = 8.4 Hz, 1H), 4.82 (s, 1H), 3.86 – 3.83 (m, 4H), 3.81 (s, 3H), 3.35 (dd, *J* = 7.6, 5.2 Hz, 2H), 3.17 – 3.11 (m, 4H). ^{13}C NMR (150 MHz, $CDCl_3$) δ = 163.2, 160.1, 153.3, 152.5, 150.5, 132.5, 131.9, 130.5, 130.4, 128.8, 128.0, 123.8, 122.7, 121.9, 116.4, 116.2, 115.8, 111.8, 108.4, 66.8, 56.0, 48.8, 40.8, 35.7. DEPT-135 (150 MHz, $CDCl_3$) δ = 131.9, 130.5, 128.8,

128.0, 123.9, 122.8, 116.5, 116.3, 111.9, 66.8 (neg.), 56.0, 48.8 (neg.), 40.9, 35.8 (neg.). HRMS (ESI) m/z calcd for : $C_{28}H_{25}ClNO_5^-$ (M-H)$^-$ 490.1421, found 490.1422.

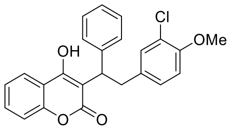

3-(2-(3-chloro-4-methoxyphenyl)-1-phenylethyl)-4-hydroxy-2H-chromen-2-one (65):
Yield: 84%; 68.4 mg; yellow solid; m.p. 206-208 ºC; TLC (nHex:EtOAc, 4:1 v/v): R_f = 0.3; ^{1}H NMR (600 MHz, $CDCl_3$) δ = 8.00 (s, 1H), 7.79 (d, *J* = 8.0 Hz, 1H), 7.53 (d, *J* = 7.7 Hz, 2H), 7.46 (t, *J* = 7.7 Hz, 1H), 7.30 (t, *J* = 7.6 Hz, 2H), 7.26 – 7.19 (m, 3H), 7.15 (d, *J* = 8.3 Hz, 1H), 7.08 – 7.04 (m, 1H), 6.64 (d, *J* = 8.4 Hz, 1H), 4.92 (t, *J* = 8.1 Hz, 1H), 3.72 (s, 3H), 3.56 (dd, *J* = 14.0, 9.0 Hz, 1H), 3.43 (dd, *J* = 14.0, 7.3 Hz, 1H). ^{13}C NMR (150 MHz, $CDCl_3$) δ = 163.8, 160.8, 153.2, 152.4, 141.0, 132.8, 131.9, 130.4, 129.0, 128.0, 127.9, 127.3, 123.9, 122.9, 121.8, 116.4, 115.8, 111.8, 108.0, 55.9, 41.8, 35.7. DEPT-135 (150 MHz, $CDCl_3$) δ = 132.0, 130.5, 129.2, 128.1, 128.0, 127.5, 124.0, 122.9, 116.5, 111.9, 56.0, 41.8, 35.7 (neg.). HRMS (ESI) m/z calcd for : $C_{24}H_{18}ClO_4^-$ (M-H)$^-$ 405.0894, found 405.0912.

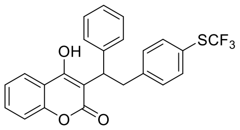

4-hydroxy-3-(1-phenyl-2-(4-((trifluoromethyl)thio)phenyl)ethyl)-2H-chromen-2-one (66):
Yield: 93%; 82.3 mg; white solid; m.p. 177-179 ºC; TLC (nHex:EtOAc, 6:1 v/v): R_f = 0.3; ^{1}H NMR (600 MHz, $CDCl_3$) δ = 7.81 (d, *J* = 7.9 Hz, 1H), 7.53 (d, *J* = 7.6 Hz, 2H), 7.48 (t, *J* = 7.7 Hz, 1H), 7.39 (d, *J* = 7.8 Hz, 2H), 7.30 (t, *J* = 7.5 Hz, 2H), 7.27 – 7.19 (m, 4H), 7.14 (d, *J* = 8.3 Hz, 1H), 4.97 (t, *J* = 8.0 Hz, 1H), 3.67 (dd, *J* = 13.7, 9.1 Hz, 1H), 3.55 (dd, *J* = 13.9, 7.2 Hz, 1H). ^{13}C NMR (150 MHz, $CDCl_3$) δ = 164.0, 161.3, 152.3, 143.2, 141.0, 136.2, 132.0, 130.5, 130.0, 129.0, 128.5, 127.9, 127.3, 124.1, 123.0, 121.7, 116.4, 115.8, 107.8, 41.5, 36.6. DEPT-135 (150 MHz, $CDCl_3$) δ = 136.3, 132.1, 130.0, 129.1, 128.0, 127.4, 124.1, 123.0, 116.5, 41.6, 36.6 (neg.). ^{19}F NMR (564 MHz, $CDCl_3$) δ = -42.97. HRMS (ESI) m/z calcd for : $C_{24}H_{16}F_3O_3S^-$ (M-H)$^-$ 441.0772, found 441.0771.

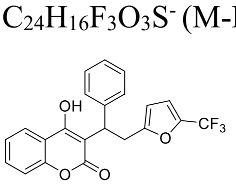

4-hydroxy-3-(1-phenyl-2-(5-(trifluoromethyl)furan-2-yl)ethyl)-2H-chromen-2-one (67):
Yield: 77%; 61.6 mg; yellow solid; m.p. 170-172 ºC; TLC (nHex:EtOAc, 6:1 v/v): R_f = 0.3; ^{1}H NMR (600 MHz, $CDCl_3$) δ = 8.73 (s, 1H), 7.91 (d, *J* = 8.0 Hz, 1H), 7.50 (dd, *J* = 14.7, 7.7 Hz, 3H), 7.30 – 7.17 (m, 5H), 6.53 (s, 1H), 6.05 (d, *J* = 2.6 Hz, 1H), 5.04 (t, *J* = 8.0 Hz, 1H), 3.81 (dd, *J* = 15.3, 9.0 Hz, 1H), 3.59 (dd, *J* = 15.3, 7.0 Hz, 1H). ^{13}C NMR (150 MHz, $CDCl_3$) δ = 164.1, 161.7, 157.1, 152.4, 140.7, 140.3, 139.8, 132.1, 128.7, 127.8, 127.1, 124.1, 123.1, 121.8, 120.0, 118.2, 116.5, 115.8, 112.4, 107.5, 107.2, 39.0, 29.5. DEPT-135 (150 MHz, $CDCl_3$) δ = 132.2, 128.8, 127.8, 127.2, 124.2, 123.2, 116.6, 112.4, 107.5, 39.1, 29.6 (neg.). ^{19}F

NMR (564 MHz, $CDCl_3$) δ = -63.95. HRMS (ESI) m/z calcd for : $C_{22}H_{14}F_3O_4^-$ $(M-H)^-$ 399.0844, found 399.0846.

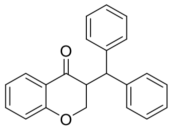

3-benzhydrylchroman-4-one (68):

Yield: 73%; 45.8 mg; yellow oil; TLC (nHex:EtOAc, 40:1 v/v): R_f = 0.3; ^1H NMR (600 MHz, $CDCl_3$) δ = 7.76 (dd, *J* = 7.7, 1.3 Hz, 1H), 7.51 – 7.46 (m, 1H), 7.31 (d, *J* = 4.3 Hz, 4H), 7.28 – 7.25 (m, 4H), 7.22 (dt, *J* = 8.7, 4.3 Hz, 1H), 7.17 (ddd, *J* = 8.5, 6.1, 2.3 Hz, 1H), 6.99 (dd, *J* = 12.1, 4.8 Hz, 2H), 4.54 (d, *J* = 10.2 Hz, 1H), 4.44 (dd, *J* = 11.8, 3.3 Hz, 1H), 4.20 (dd, *J* = 11.8, 5.6 Hz, 1H), 3.52 – 3.45 (m, 1H). ^{13}C NMR (150 MHz, $CDCl_3$) δ = 193.1, 161.0, 141.4, 141.3, 135.8, 128.9, 128.4, 128.4, 128.2, 127.7, 127.0, 126.8, 121.5, 120.7, 117.6, 69.6, 49.8, 48.6. DEPT-135 (150 MHz, $CDCl_3$) δ = 135.8, 128.9, 128.4, 128.4, 128.2, 127.7, 127.0, 126.8, 121.5, 117.6, 69.6 (neg.), 49.8, 48.6. HRMS (ESI) m/z calcd for : $C_{22}H_{18}O_2Na^+$ $(M+Na)^+$ 337.1204, found 337.1207.

Supplementary Note 5. Crystallographic data and molecular structures of 1, 8, 29, 56, 58

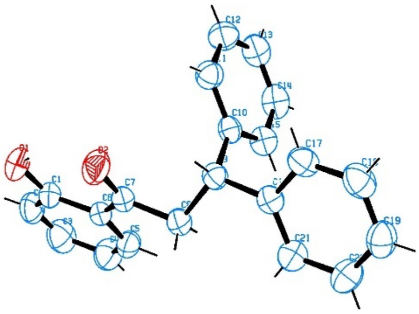

CCDC number: 2267471

Figure S1. X-ray crystal structure of **1**.

```
Bond precision:   C-C = 0.0030 A          Wavelength=0.71073

Cell:           a=6.877(3)         b=8.230(4)         c=14.364(8)
                alpha=83.598(19)   beta=82.538(18)    gamma=88.435(17)
Temperature:    283 K

                 Calculated                  Reported
Volume           801.0(7)                    801.0(7)
Space group      P -1                        P -1
Hall group       -P 1                        -P 1
Moiety formula   C21 H18 O2                  C21 H18 O2
Sum formula      C21 H18 O2                  C21 H18 O2
Mr               302.35                      302.35
Dx,g cm-3        1.254                       1.254
Z                2                           2
Mu (mm-1)        0.079                       0.079
F000             320.0                       320.0
F000'            320.14
h,k,lmax         9,10,18                     9,10,18
Nref             3853                        3819
Tmin,Tmax        0.997,0.999
Tmin'            0.996

Correction method= Not given

Data completeness= 0.991           Theta(max)= 27.998

                                                       wR2(reflections)=
R(reflections)= 0.0658( 2286)                          0.1872( 3819)

S = 1.022                  Npar= 210
```

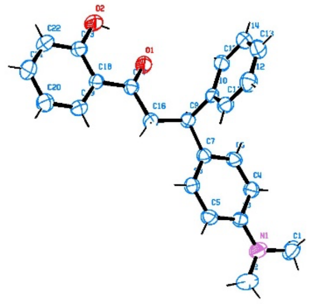

CCDC number: 2359824

Figure S2. X-ray crystal structure of **8**.

Bond precision: C-C = 0.0031 A   Wavelength=0.71073

Cell:	a=12.263(9)	b=10.773(7)	c=14.624(12)
	alpha=90	beta=96.92(3)	gamma=90
Temperature:	296 K		

	Calculated	Reported
Volume	1918(2)	1918(2)
Space group	P 21/c	P 21/c
Hall group	-P 2ybc	-P 2ybc
Moiety formula	C23 H23 N O2	C23 H23 N O2
Sum formula	C23 H23 N O2	C23 H23 N O2
Mr	345.42	345.42
Dx,g cm-3	1.196	1.196
Z	4	4
Mu (mm-1)	0.076	0.076
F000	736.0	736.0
F000’	736.31	
h,k,lmax	16,14,19	16,14,18
Nref	4459	4391
Tmin,Tmax	0.983,0.986	
Tmin’	0.983	

Correction method= Not given

Data completeness= 0.985   Theta(max)= 27.640

R(reflections)= 0.0613(2596)   wR2(reflections)= 0.1482(4391)

S = 1.020   Npar= 238

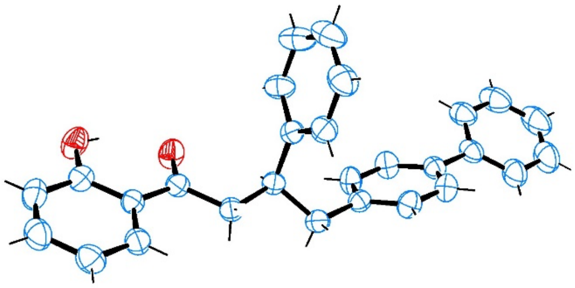

CCDC number: 2359825

Figure S3. X-ray crystal structure of **29**.

```
Bond precision:   C-C = 0.0035 A          Wavelength=0.71073

Cell:           a=6.527(2)         b=10.288(3)       c=16.793(5)
                alpha=72.929(11)   beta=82.322(11)   gamma=89.942(11)
Temperature:    296 K

                 Calculated                   Reported
Volume           1067.4(6)                    1067.4(6)
Space group      P -1                         P -1
Hall group       -P 1                         -P 1
Moiety formula   C28 H24 O2                   C28 H24 O2
Sum formula      C28 H24 O2                   C28 H24 O2
Mr               392.47                       392.47
Dx,g cm-3        1.221                        1.221
Z                2                            2
Mu (mm-1)        0.075                        0.075
F000             416.0                        416.0
F000'            416.18
h,k,lmax         8,13,22                      8,13,22
Nref             5104                         5076
Tmin,Tmax        0.985,0.988
Tmin'            0.985

Correction method= Not given

Data completeness= 0.995           Theta(max)= 27.896

                                                      wR2(reflections)=
R(reflections)= 0.0618( 2761)                          0.1876( 5076)
S = 1.018                  Npar= 272
```

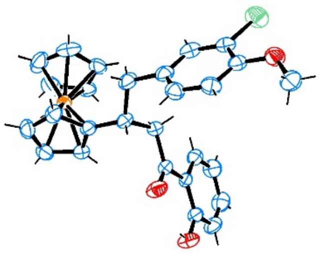

CCDC number: 2369868

Figure S4. X-ray crystal structure of **56**.

```
Bond precision:   C-C = 0.0047 A          Wavelength=0.71073

Cell:             a=7.4316(4)        b=11.6198(6)      c=14.0999(8)
                  alpha=74.061(2)    beta=81.684(2)    gamma=78.397(2)
Temperature:      298 K

                  Calculated                   Reported
Volume            1141.67(11)                  1141.67(11)
Space group       P -1                         P -1
Hall group        -P 1                         -P 1
Moiety formula    C27 H25 Cl Fe O3             C27 H25 Cl Fe O3
Sum formula       C27 H25 Cl Fe O3             C27 H25 Cl Fe O3
Mr                488.77                       488.77
Dx,g cm-3         1.422                        1.422
Z                 2                            2
Mu (mm-1)         0.804                        0.804
F000              508.0                        508.0
F000'             509.17
h,k,lmax          9,14,17                      9,14,17
Nref              4499                         4497
Tmin,Tmax         0.857,0.968
Tmin'             0.851

Correction method= Not given

Data completeness= 1.000           Theta(max)= 26.000

                                                wR2(reflections)=
R(reflections)= 0.0423( 3152)                   0.0891( 4497)

S = 1.029                  Npar= 291
```

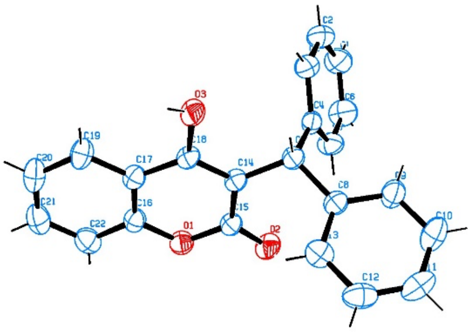

CCDC number: 2401627

Figure S5**.** X-ray crystal structure of **58**.

```
Bond precision:   C-C = 0.0039 A           Wavelength=0.71073

Cell:             a=11.3153(9)     b=13.2063(8)        c=11.3542(8)
                  alpha=90         beta=101.659(3)     gamma=90
Temperature:      273 K

                  Calculated                   Reported
Volume            1661.7(2)                    1661.7(2)
Space group       P 21/n                       P 21/n
Hall group        -P 2yn                       -P 2yn
Moiety formula    C22 H16 O3                   C22 H16 O3
Sum formula       C22 H16 O3                   C22 H16 O3
Mr                328.35                       328.35
Dx,g cm-3         1.312                        1.312
Z                 4                            4
Mu (mm-1)         0.087                        0.087
F000              688.0                        688.0
F000'             688.34
h,k,lmax          14,16,14                     14,16,14
Nref              3435                         3290
Tmin,Tmax         0.983,0.986
Tmin'             0.983

Correction method= Not given

Data completeness= 0.958            Theta(max)= 26.498

                                                          wR2(reflections)=
R(reflections)= 0.0725( 2343)                             0.1932( 3290)
S = 1.075                  Npar= 228
```

Supplementary Note 6. Materials for Biological Studies

Blank SD rat plasma (K2 EDTA as anticoagulant) was from the Drug Safety Testing Center (DSC, Hong Kong). Single-Use Rapid Equilibrium Dialysis (RED) plate with inserts (48-well, 8K MWCO, catalog number 90006) was purchased from Thermo Fisher Scientific. Phosphate buffered saline (PBS) was purchased from Gibco. Dimethyl sulfoxide (DMSO) and Cremophor EL (CrEL) were purchased from Sigma-Aldrich. Caffeine and verapamil were purchased from Sigma-Aldrich. Warfarin sodium was purchased from Macklin. Acetonitrile (ACN), methanol (MeOH), and formic acid were purchased from Fisher Chemical. Fentanyl and midazolam used for anesthesia were purchased from Hameln/Mekim (Germany), and medetomidine was purchased from Dechra (England).

Supplementary Method 2. Analytical Methods for Biological Samples

Biological samples were analyzed using a liquid chromatography-electrospray ionization triple quadrupole mass spectrometer (LC-MS/MS). Chromatographic separation was performed on a Shimadzu LC-40D system with a Kinetex 2.6 μm Polar C18 100 Å column (LC column: 50 × 2.1 mm). The mobile phases consisted of (A) Milli-Q water containing 0.1% (v/v) formic acid and (B) ACN containing 0.1% (v/v) formic acid. Separation was achieved using the following gradient elution program (**Table S3**). The flow rate was set at 0.3 mL/min, and the column temperature was set to 40°C. The autosampler temperature was maintained at 4°C. A sample volume of 2 μL was injected for analysis.

Table S3 Gradient elution program in LC-MS/MS

Time (minute)	Mobile Phase A (%)	Mobile Phase B (%)
0	90	10
1.3	90	10
6	10	90
7.3	10	90
7.4	90	10
10	90	10

The LC system was coupled online to a Sciex QTRAP 5500+ mass spectrometer equipped with an IonDrive TurboV electrospray ionization (ESI) source. Analytes were detected in both positive and negative ionization modes, depending on the chemical properties of the compounds. Compounds **58-62** were analyzed for positive ion mode. The optimized MS parameters included a capillary voltage of 5.5 kV, a curtain gas of 20 psi, a source temperature of 400°C, and gas flows of 50 psi (gas 1) and 70 psi (gas 2). Compounds **63-67** were analyzed for negative ion mode. The MS parameters included a capillary voltage of -3.5 kV, a curtain gas of 20 psi, a source temperature of 450°C, and gas flows of 40 psi (gas 1) and 70 psi (gas 2). Detection was performed in multiple reaction monitoring (MRM) mode specific to the ion pairs of each analyte. Optimal values for declustering potential (DP), entrance potential (EP), collision energy (CE), and collision cell exit potential (CXP) were determined for each

transition. The dwell time for each MRM channel was set to 100 milliseconds for precise and sensitive quantification.

Table S4. Ion pairs, MS detection parameters, and retention time for each analyte

Compound ID	MS/MS transition (m/z)	DP (V)	EP (V)	CE (V)	CXP(V)	Retention time (minute)
**58**	329.0/251.1	34.86	11.78	41	21.31	4.70
**59**	413.0/251.1	251.10	62.88	5	25.00	5.10
**60**	363.0/285.0	30.50	9.77	25	21.88	4.86
**61**	361.1/251.2	24.96	11.23	25	13.36	4.90
**62**	373.0/251.1	35.51	5.84	25	12.93	4.62
**63**	451.3/407.3	-52.79	-7.90	-50	-10.97	6.37
**64**	490.0/335.2	-85.43	-11.07	-35	-16.74	4.58
**65**	405.0/250.1	-51.98	-4.69	-30	-19.19	4.84
**66**	441.0/250.0	-53.00	-7.88	-30	-10.17	5.23
**67**	399.9/251.2	-88.97	-10.23	-25	-13.13	4.89

Supplementary Method 3. Rapid Equilibrium Dialysis Assay

Plasma protein binding (PPB) for test compounds (**58-67**) was evaluated using the RED method. The assay was performed with RED inserts (Catalog No. 90006, Thermo Fisher Scientific) following the manufacturer's protocol. Each test compound was evaluated at a concentration of 2 μg/mL at a single time point (4 hours) in triplicate. Caffeine (5 μM) and verapamil (5 μM) were included as benchmark controls. For the assay, 100 μL of plasma spiked with the test compound was added to the sample chamber of the RED device, while 350 μL of PBS (pH 7.4) was added to the buffer chamber. The RED device was incubated at 37 °C with shaking at 80 rpm for 4 hours to allow equilibrium to be achieved. Following the incubation, 5 μL of plasma was collected from the sample chamber and diluted with 195 μL of blank PBS. Simultaneously, 19 μL of buffer was collected from the buffer chamber and diluted with 1 μL of blank plasma. The diluted samples were vortexed at room temperature for 5 minutes. Subsequently, 20 μL of the diluted sample was mixed with 80 μL of MeOH, followed by centrifugation at 4,500 rpm for 25 minutes. The resulting supernatants were collected for analysis using LC-MS/MS. The data was analyzed to determine the fraction bound (f_{bound}) of each compound to plasma proteins using the following equations:

$f_{\text{bound}} = 1 - f_{\text{unbound}}$,

where the unbound fraction (f_{unbound}) was determined as:

$f_{\text{unbound}} = C_{\text{buffer}}/(C_{\text{buffer}} + C_{\text{plasma}})$,

Here, C_{buffer} represents the concentration of the test compounds in the buffer chamber, and C_{plasma} represents the concentration of the test compounds in the plasma chamber. The percentage of plasma protein binding (PPB) was subsequently calculated using the formula:

PPB (%) = $(1 - f_{\text{unbound}}) \times 100\%$

Supplementary Method 4. Plasma Stability Assay

Test compounds (**58-67**) were evaluated for stability in rat plasma by incubating them at a concentration of 2 μg/mL with 100 μL of freshly prepared rat plasma (n = 1 biologically independent sample). Samples were incubated at 37°C with shaking at 80 rpm for 4 hours. The reaction was terminated by adding MeOH at a 4:1 volume ratio (MeOH: plasma). The remaining concentrations of the test compounds in plasma were quantified using LC-MS/MS. The stability of each compound was expressed as the percentage of the compound remaining at 4 hours relative to the concentration measured in the 0-minute (initial) sample.

Supplementary Method 5. Animal and Ethics Statement

Male ICR (CD-1) mice (6-8 weeks) were used in the *in vivo* anti-coagulation assay. All the procedures related to animal welfare in this study are in compliance with the animal welfare policies and the guidelines of Drug Safety Testing Center (DSC), Hong Kong. The study has been reviewed and approved by the DSC Institutional Animal Care and Use Committee (IACUC) (Ethics code: 24-019).

Supplementary Method 6. Drug Preparation

Warfarin sodium (4 mg/mL) was dissolved in saline. The test compounds (**58-67**) were dissolved in a formulation (DMSO: CrEL: saline = 0.5: 0.5: 9) to obtain 4 mg/mL. The solvent must be added in sequence, and another solvent should only be added once the compound has been thoroughly dissolved or combined.

Supplementary Method 7. *In vivo* Anticoagulation Assay

Male ICR (CD-1) mice (6-8 weeks) were randomly divided into several groups (n = 3 mice per group). Mice were administered with test compounds (**58-67**) by intravenous injections (I.V.) once daily for two consecutive days (Day 0-1). The dose of compounds **58-67** was 20 mg/kg, while the warfarin sodium dose was 5-20 mg/kg (no difference in efficacy). On Day 2 (24 hours after the last dose), mice were anesthetized by intraperitoneal injection (I.P.) of Fentanyl/Midazolam/Medetomidine mixture (0.05, 5, and 0.5 mg/kg respectively). After confirming the depth of anesthesia, an incision was made at a distance of 15 mm from the tail tip of the mouse. The animals were then placed on the heat pad, and the snipped tails were hung vertically. Blood was collected in a 1.5 mL Eppendorf tube for no more than 60 minutes until stopped bleeding or reached 40% of total blood volume. Bleeding time was defined as the time elapsed until bleeding stopped. Bleeding volume was defined as the weight of the blood in the tube after the bleeding had stopped. All the mice were sacrificed by CO_2 inhalation followed by cervical dislocation at the end of the experiment.

Supplementary Method 8. Statistical Analysis

Data was expressed as the mean and standard deviation (SD). Statistical differences were calculated in GraphPad Prism. The one-way ANOVA (two-sided) analysis was used to test the statistical differences among the means of two or more groups. A *p*-value of 0.05 or lower was considered statistically significant.

Supplementary Note 7. Plasma Protein Binding Rates of Ten Tested Compounds

The rat plasma protein binding rates of compounds **58-67** were evaluated using the RED method at a concentration of 2 μg/mL. The results, shown in **Table S5**, revealed that the rat plasma binding rates of compounds **58**, **59**, **60**, **61**, **62**, **64**, **65**, **66**, and **67** exceeded 99.5%. However, for compound **63**, no peak was detected in the buffer samples, precluding the determination of its plasma binding rate.

Table S5. Rat plasma protein binding rates of compounds 58-67 (n = 3 biologically independent samples)

Test compound	%Unbound	Average	SD	% Binding	Average
**58**	0.378%	0.463%	0.127%	99.62%	99.54%
	0.403%			99.60%	
	0.609%			99.39%	
**59**	0.120%	0.107%	0.012%	99.88%	99.89%
	0.098%			99.90%	
	0.103%			99.90%	
**60**	0.120%	0.108%	0.010%	99.88%	99.89%
	0.103%			99.90%	
	0.101%			99.90%	
**61**	0.078%	0.0934%	0.018%	99.92%	99.91%
	0.112%			99.89%	
	0.090%			99.91%	
**62**	0.194%	0.197%	0.003%	99.81%	99.80%
	0.197%			99.80%	
	0.199%			99.80%	
**63**#	ND	NA	NA	ND	NA
	ND			ND	
	ND			ND	
**64**	0.013%	0.0139%	0.001%	99.99%	99.99%
	0.014%			99.99%	
	0.015%			99.99%	
**65**	0.061%	0.0578%	0.010%	99.94%	99.94%
	0.066%			99.93%	
	0.047%			99.95%	
**66**	0.030%	0.0342%	0.005%	99.97%	99.97%
	0.033%			99.97%	
	0.039%			99.96%	
**67**	0.200%	0.203%	0.022%	99.80%	99.80%
	0.183%			99.82%	
	0.226%			99.77%	
verapamil	9.26%	8.90%	0.97%	90.7%	91.10%
	7.81%			92.2%	
	9.65%			90.4%	
caffeine	72.4%	76.8%	5.30%	27.60%	23.17%
	82.7%			17.31%	
	75.4%			24.62%	

#: **63** was not detectable in the buffer side, with no peak observed.

Supplementary Note 8. Plasma Stability of Ten Tested Compounds

The rat plasma stability of compounds **58**-**67** was assessed to ensure accurate plasma protein binding measurements. The results, shown in **Table S6**, indicated that all the compounds, except for compound **58**, remained stable following a 4-hour incubation in rat plasma at 37°C. Compound **58** exhibited an average remaining percentage of 54.2% post-incubation.

Table S6. Stability of test compound after 4-hour incubation in rat plasma at 37 °C (n = 1 biologically independent sample)

Test compound	Percentage remaining
**58**	54.2%
**59**	118.7%
**60**	98.1%
**61**	98.8%
**62**	89.8%
**63**	106.9%
**64**	105.4%
**65**	113.1%
**66**	100.8%
**67**	101.0%
verapamil	115.8%
caffeine	97.9%

Supplementary Note 9. NMR spectra of compounds

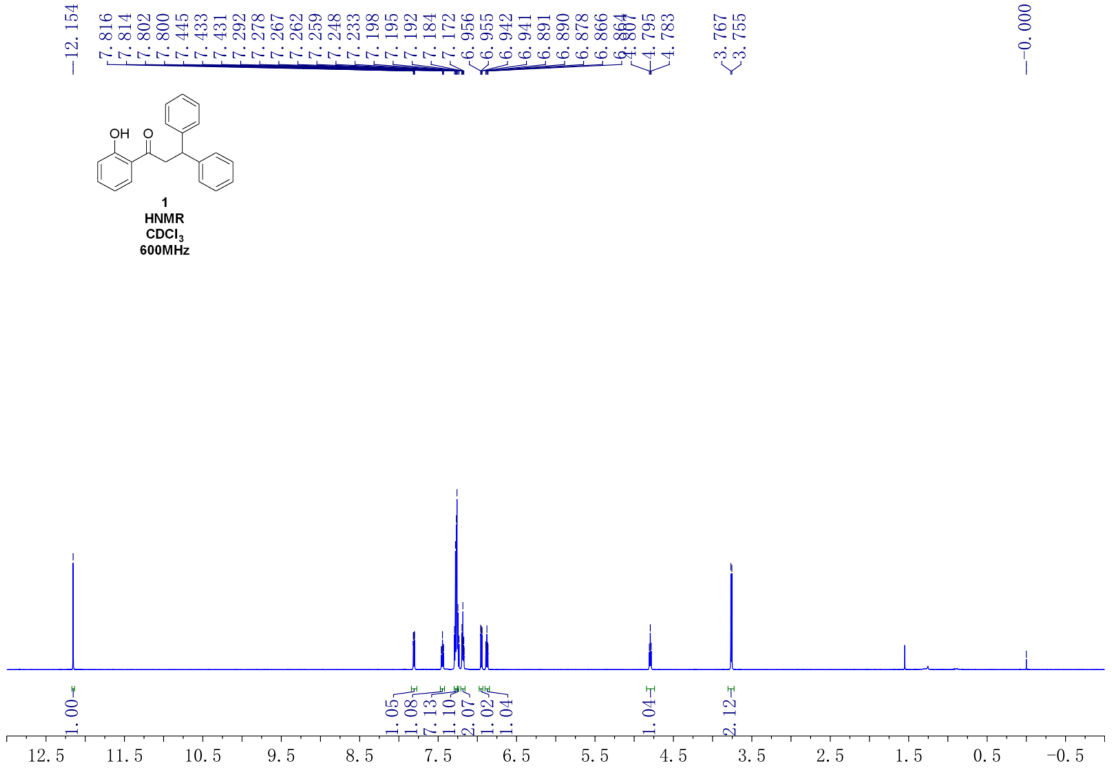

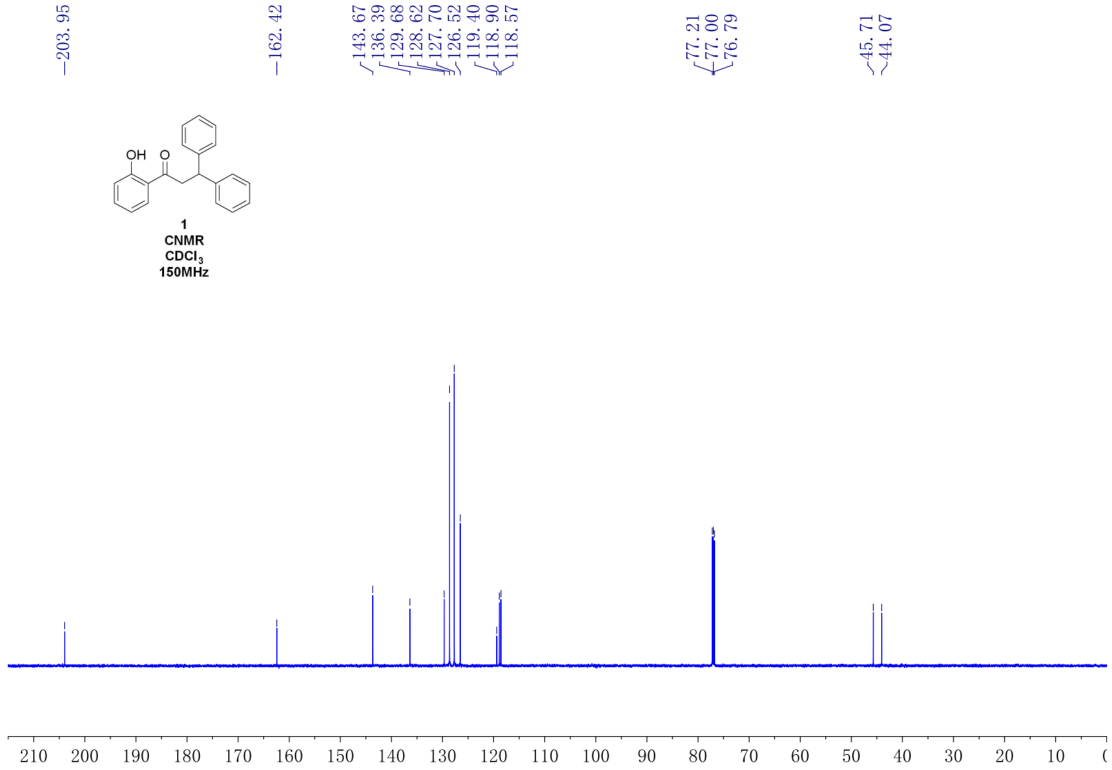

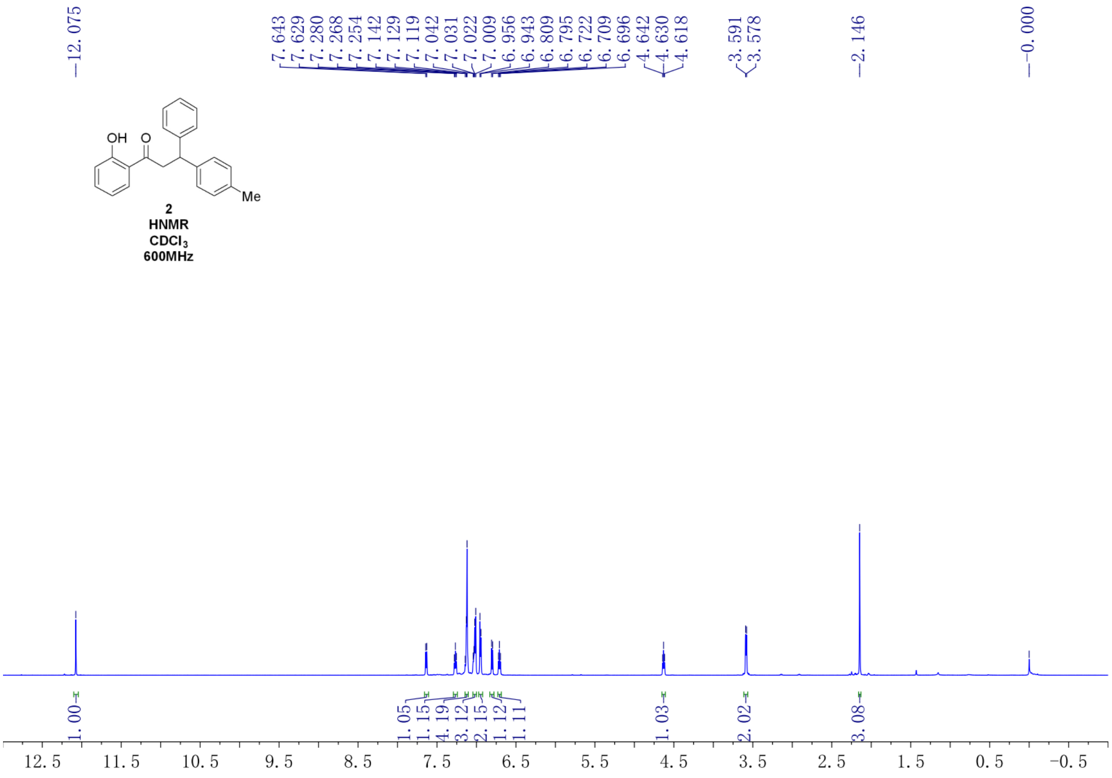
12.075
7.643
7.629
7.280
7.268
7.254
7.142
7.129
7.119
7.042
7.031
7.022
7.009
6.956
6.943
6.809
6.795
6.722
6.709
6.696
4.642
4.630
4.618
3.591
3.578
2.146
-0.000
OH
O
Me
2
HNMR
CDCl3
600MHz
1.00
1.05
1.15
4.19
3.12
2.15
1.12
1.11
1.03
2.02
3.08
12.5
11.5
10.5
9.5
8.5
7.5
6.5
5.5
4.5
3.5
2.5
1.5
0.5
-0.5

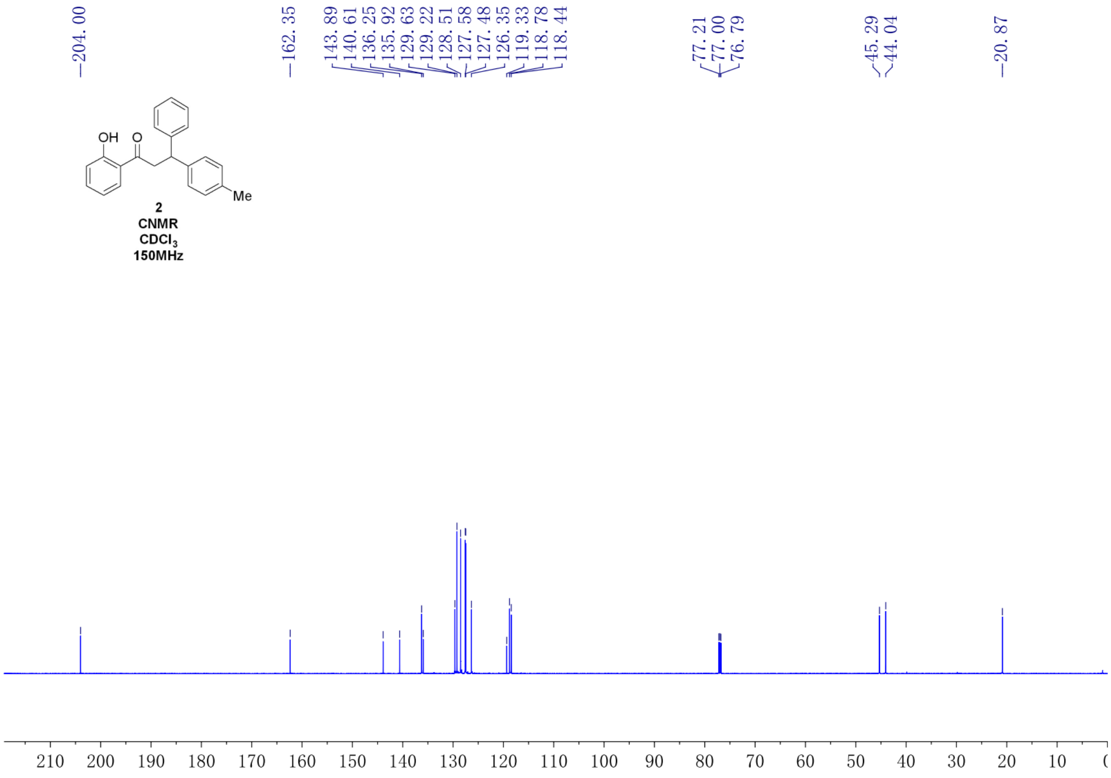
204.00
162.35
143.89
140.61
136.25
135.92
129.63
129.22
128.51
127.58
127.48
126.35
119.33
118.78
118.44
77.21
77.00
76.79
45.29
44.04
20.87
OH
O
Me
2
CNMR
CDCl3
150MHz
210
200
190
180
170
160
150
140
130
120
110
100
90
80
70
60
50
40
30
20
10

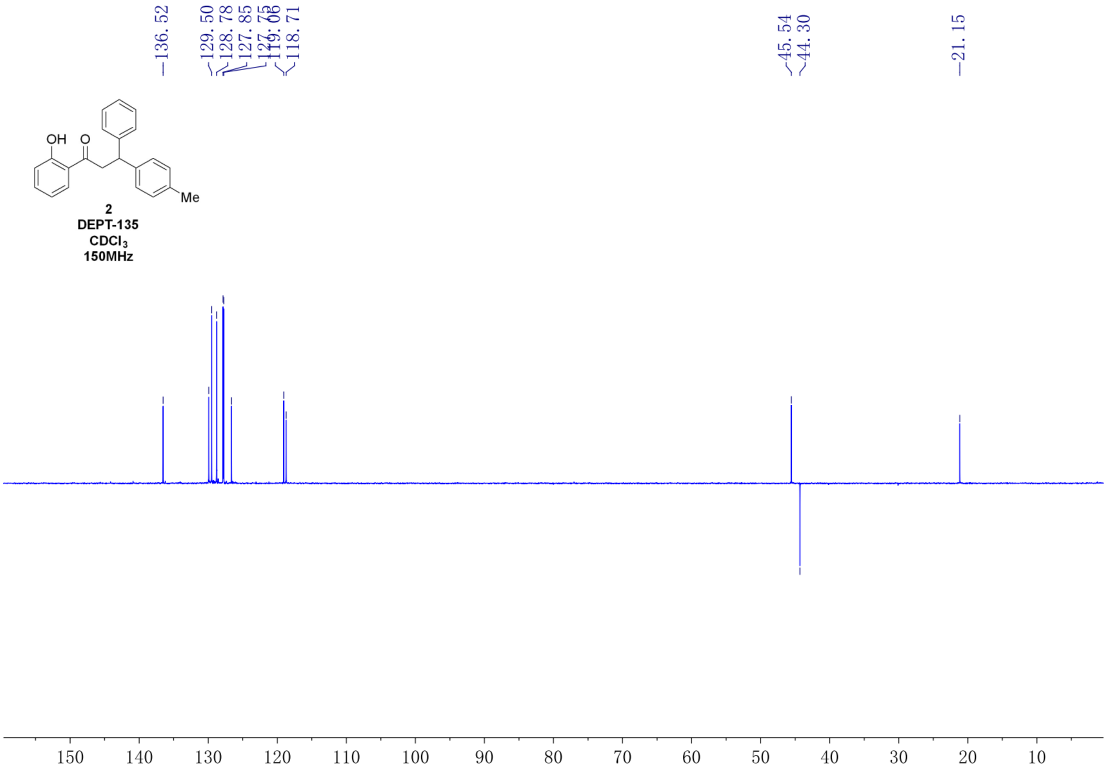
136.52
129.50
128.78
127.85
127.75
119.06
118.71
45.54
44.30
21.15
OH
O
Me
2
DEPT-135
CDCl3
150MHz
150
140
130
120
110
100
90
80
70
60
50
40
30
20
10

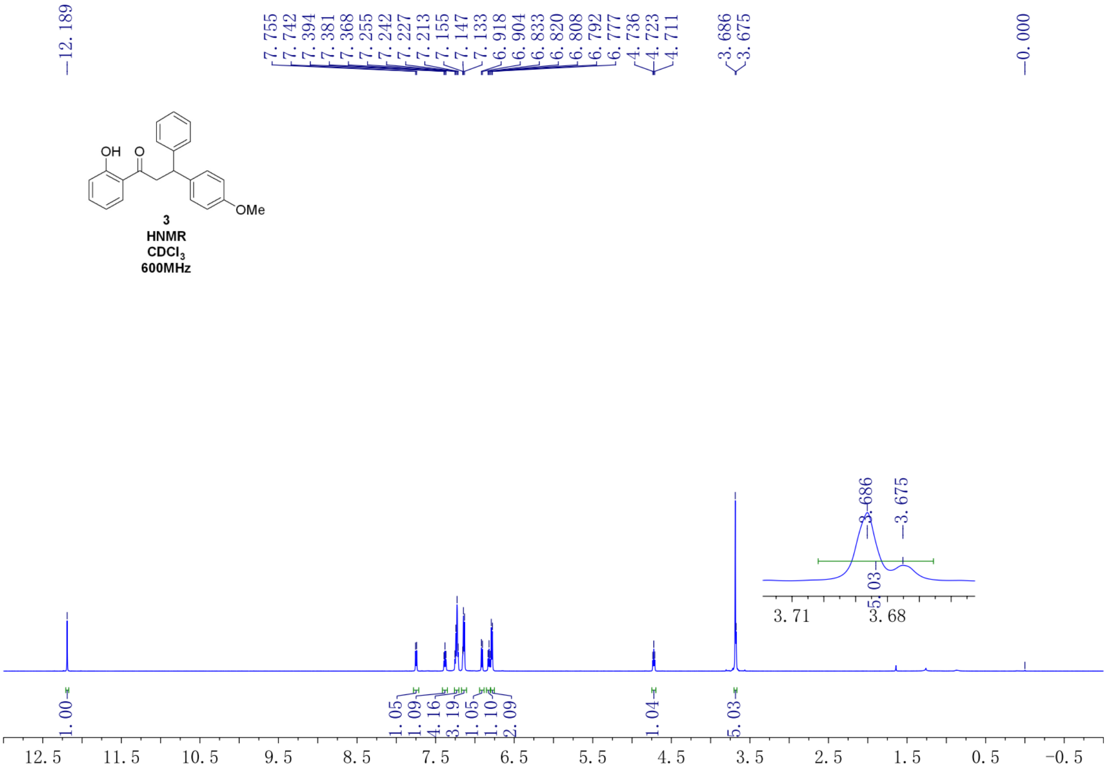
12.189
7.755
7.742
7.394
7.381
7.368
7.255
7.242
7.227
7.213
7.155
7.147
7.133
6.918
6.904
6.833
6.820
6.808
6.792
6.777
4.736
4.723
4.711
3.686
3.675
0.000
OH
O
OMe
3
HNMR
CDCl3
600MHz
3.71
3.68
5.03
1.00
1.05
1.09
4.16
3.19
1.05
1.10
2.09
1.04
5.03
12.5
11.5
10.5
9.5
8.5
7.5
6.5
5.5
4.5
3.5
2.5
1.5
0.5
-0.5

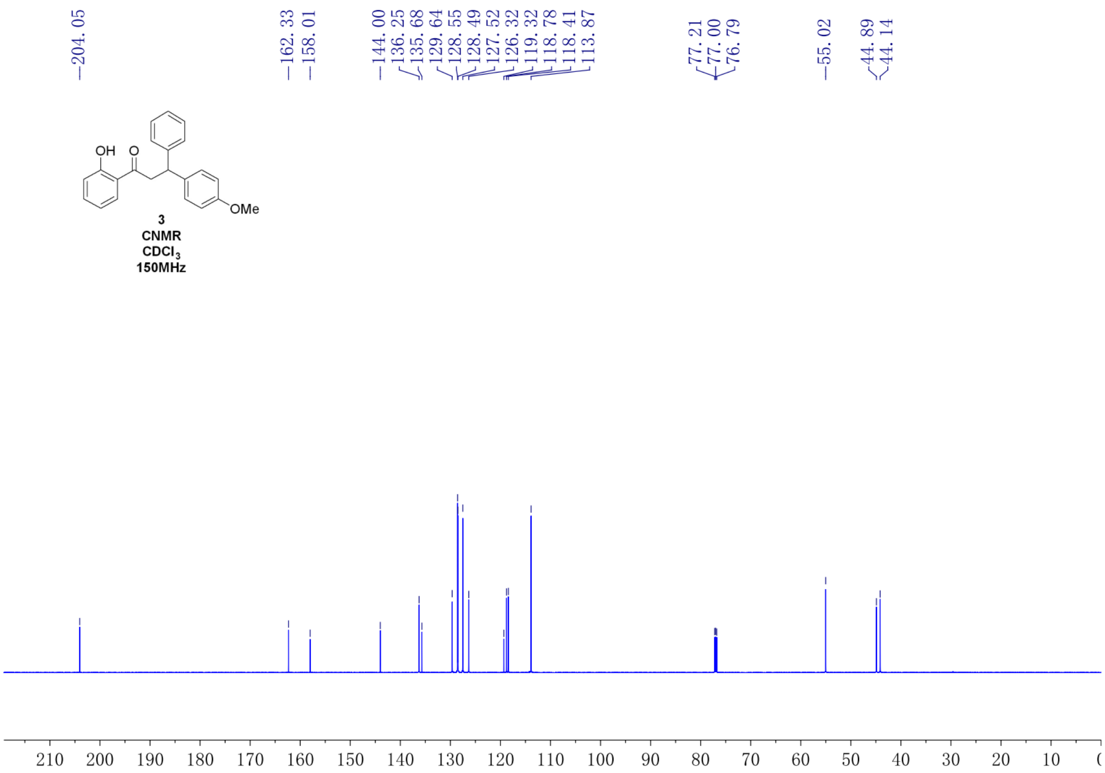
204.05
162.33
158.01
144.00
136.25
135.68
129.64
128.55
128.49
127.52
126.32
119.32
118.78
118.41
113.87
77.21
77.00
76.79
55.02
44.89
44.14
OH
O
OMe
3
CNMR
$CDCl_3$
150MHz
210 200 190 180 170 160 150 140 130 120 110 100 90 80 70 60 50 40 30 20 10

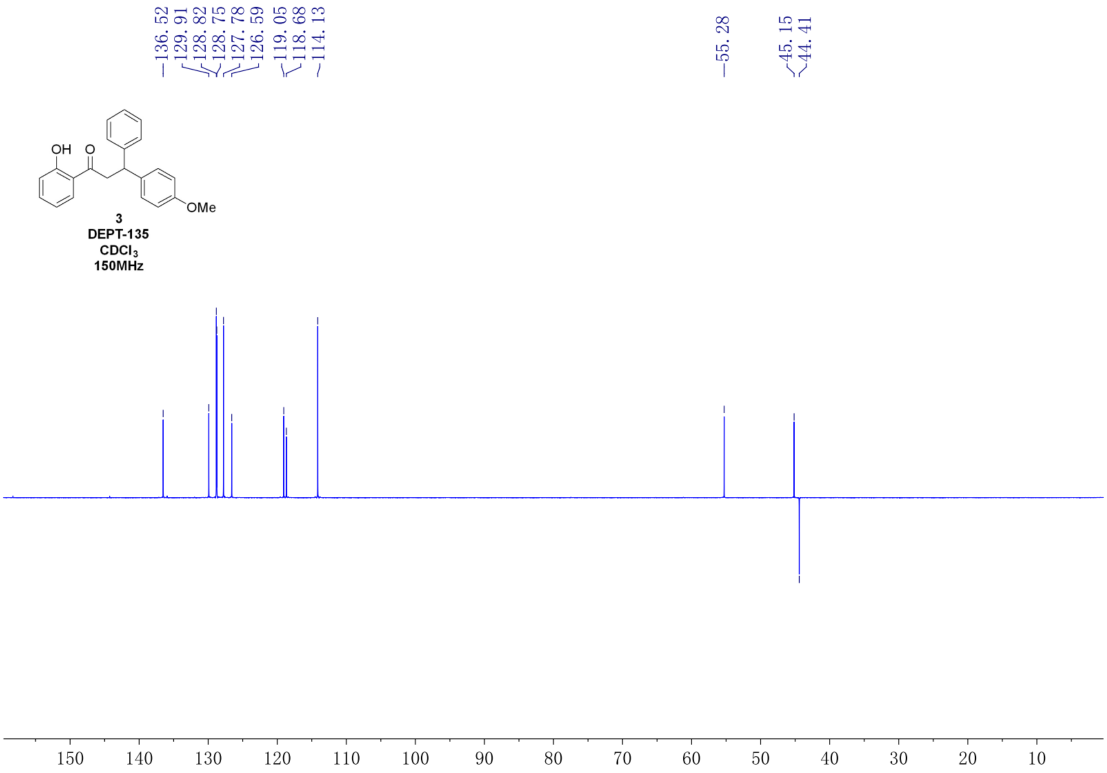
136.52
129.91
128.82
128.75
127.78
126.59
119.05
118.68
114.13
55.28
45.15
44.41
OH
O
OMe
3
DEPT-135
$CDCl_3$
150MHz
150 140 130 120 110 100 90 80 70 60 50 40 30 20 10

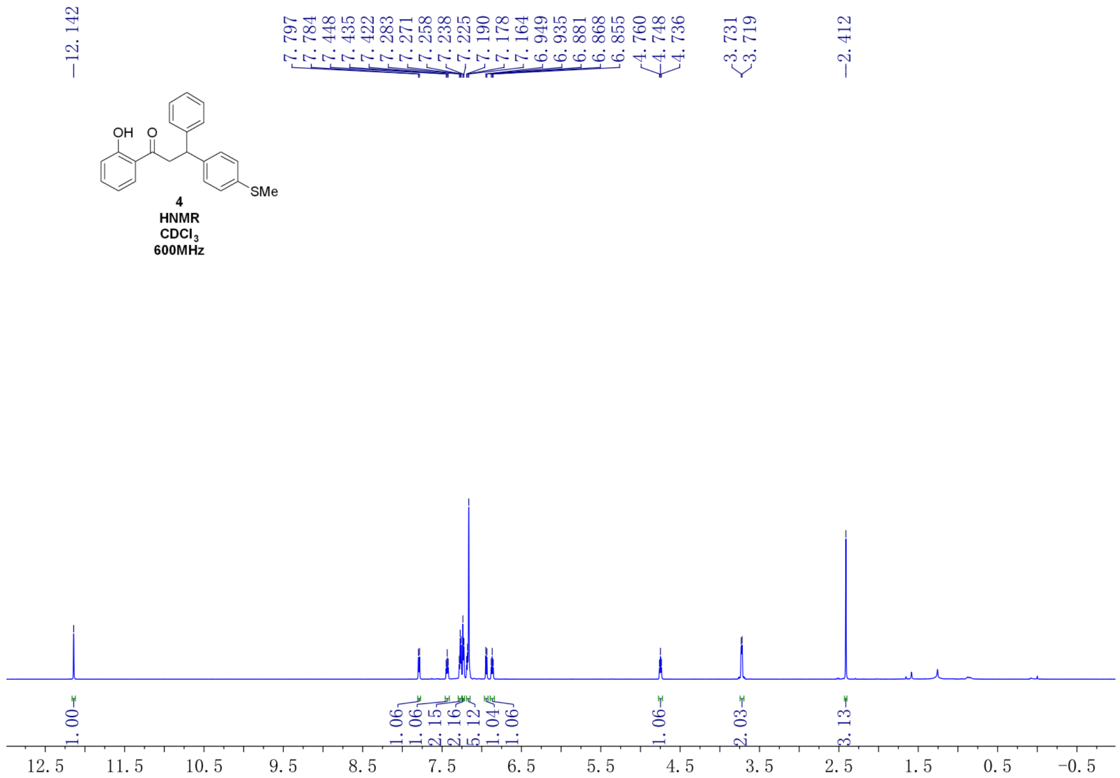
12.142
7.797
7.784
7.448
7.435
7.422
7.283
7.271
7.258
7.238
7.225
7.190
7.178
7.164
6.949
6.935
6.881
6.868
6.855
4.760
4.748
4.736
3.731
3.719
2.412
OH
O
SMe
4
HNMR
CDCl3
600MHz
1.00
1.06
1.06
2.15
2.16
5.12
1.04
1.06
1.06
2.03
3.13
12.5
11.5
10.5
9.5
8.5
7.5
6.5
5.5
4.5
3.5
2.5
1.5
0.5
-0.5

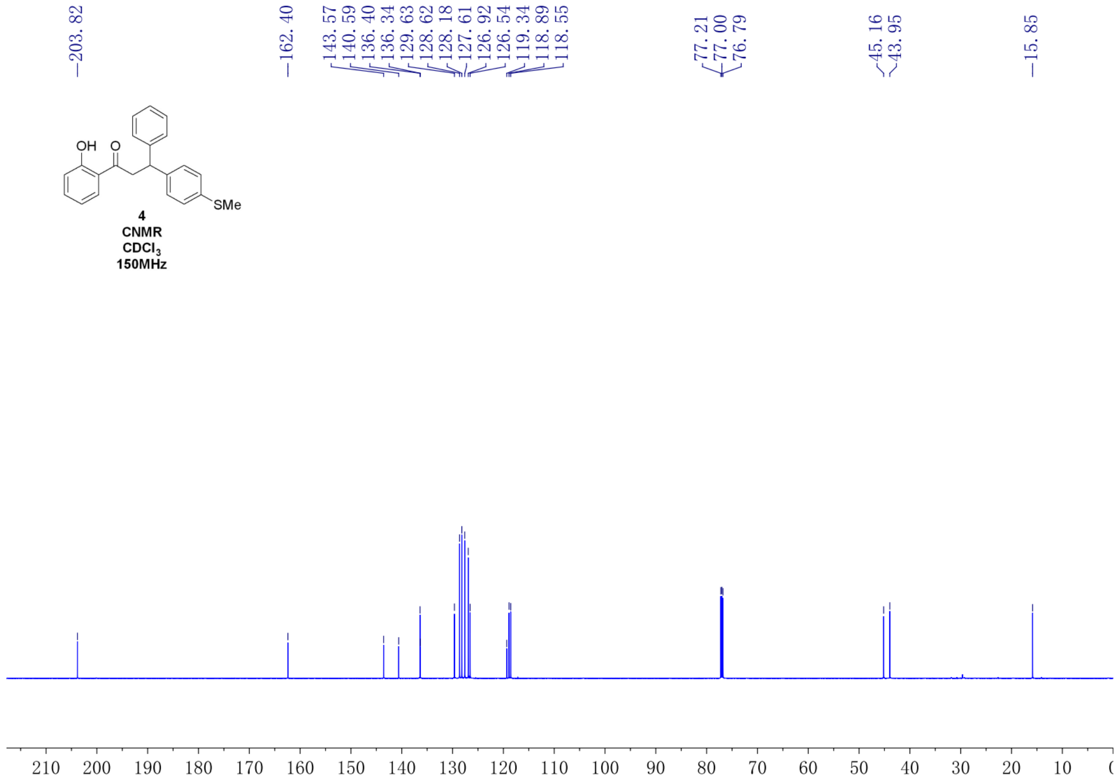
203.82
162.40
143.57
140.59
136.40
136.34
129.63
128.62
128.18
127.61
126.92
126.54
119.34
118.89
118.55
77.21
77.00
76.79
45.16
43.95
15.85
OH
O
SMe
4
CNMR
CDCl3
150MHz
210
200
190
180
170
160
150
140
130
120
110
100
90
80
70
60
50
40
30
20
10

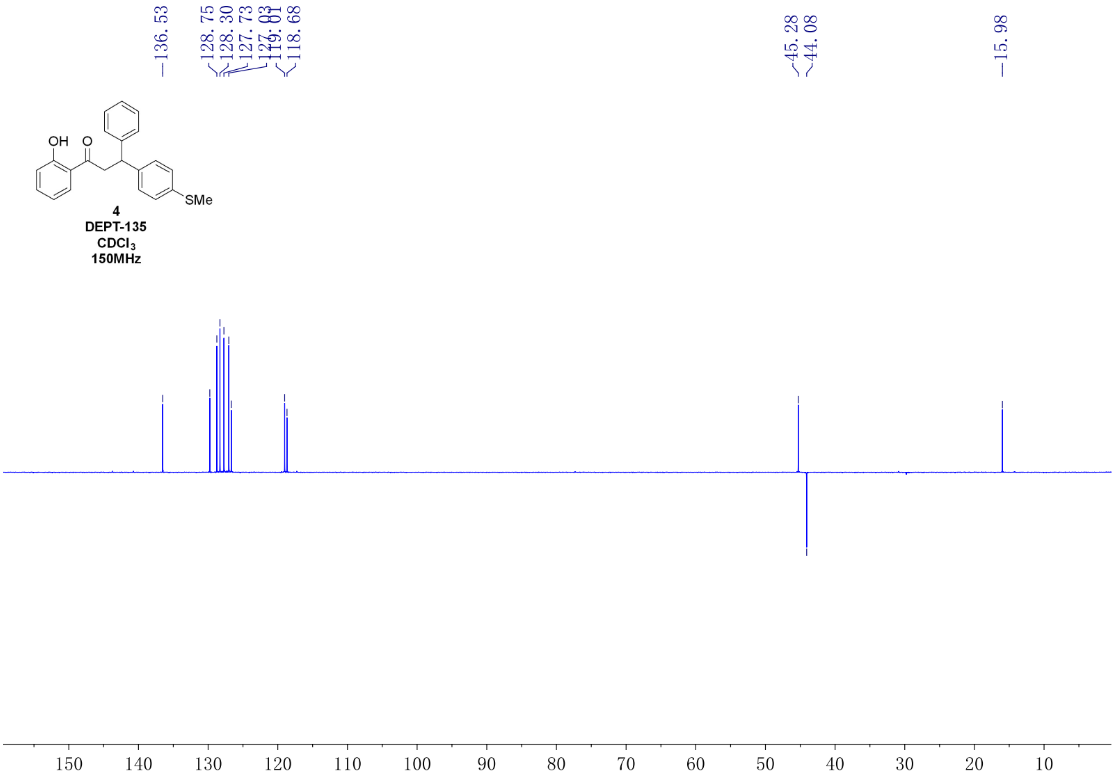
136.53
128.75
128.30
127.73
127.03
119.01
118.68
45.28
44.08
15.98
OH
O
SMe
4
DEPT-135
$CDCl_3$
150MHz
150
140
130
120
110
100
90
80
70
60
50
40
30
20
10

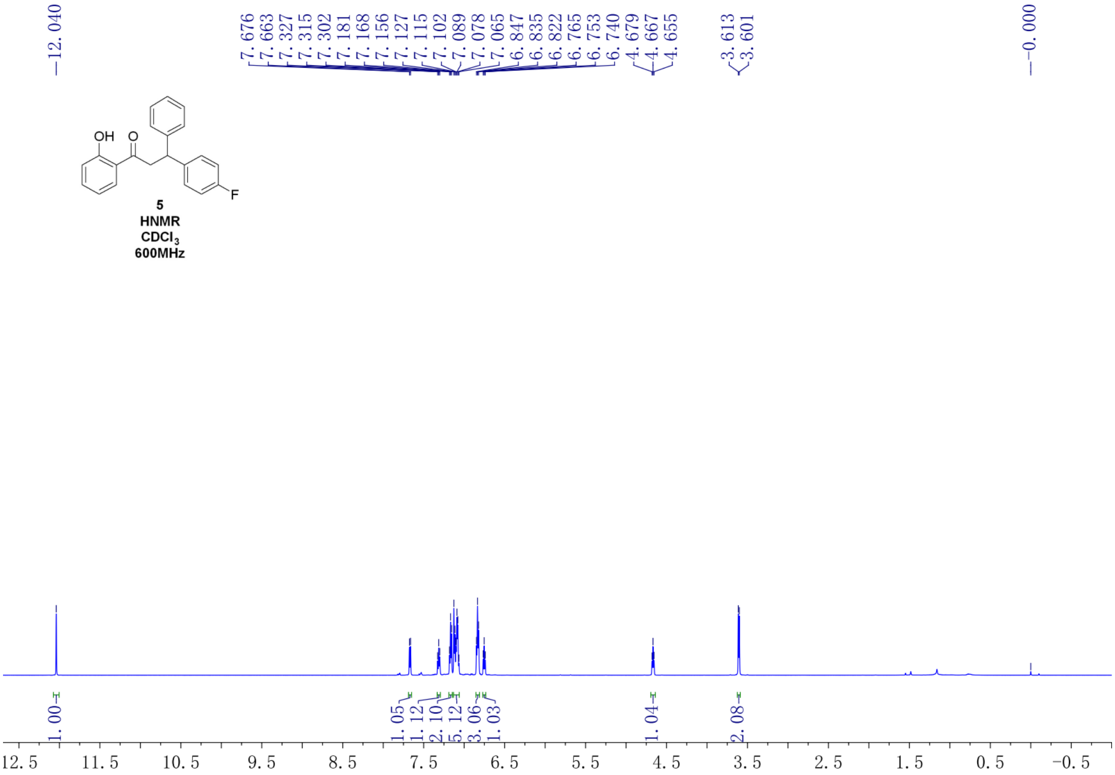
12.040
7.676
7.663
7.327
7.315
7.302
7.181
7.168
7.156
7.127
7.115
7.102
7.089
7.078
7.065
6.847
6.835
6.822
6.765
6.753
6.740
4.679
4.667
4.655
3.613
3.601
-0.000
OH
O
F
5
HNMR
$CDCl_3$
600MHz
1.00
1.05
1.12
2.10
5.12
3.06
1.03
1.04
2.08
12.5
11.5
10.5
9.5
8.5
7.5
6.5
5.5
4.5
3.5
2.5
1.5
0.5
-0.5

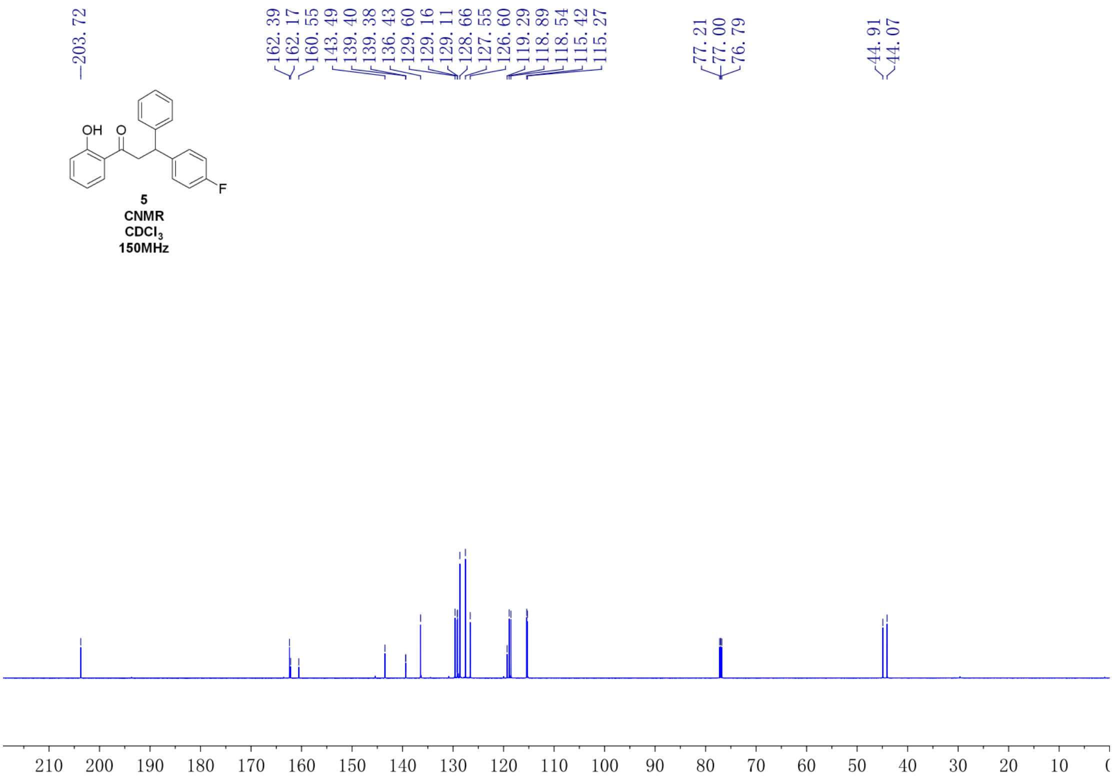
203.72
162.39
162.17
160.55
143.49
139.40
139.38
136.43
129.60
129.16
129.11
128.66
127.55
126.60
119.29
118.89
118.54
115.42
115.27
77.21
77.00
76.79
44.91
44.07
OH
O
F
5
CNMR
CDCl3
150MHz
210 200 190 180 170 160 150 140 130 120 110 100 90 80 70 60 50 40 30 20 10

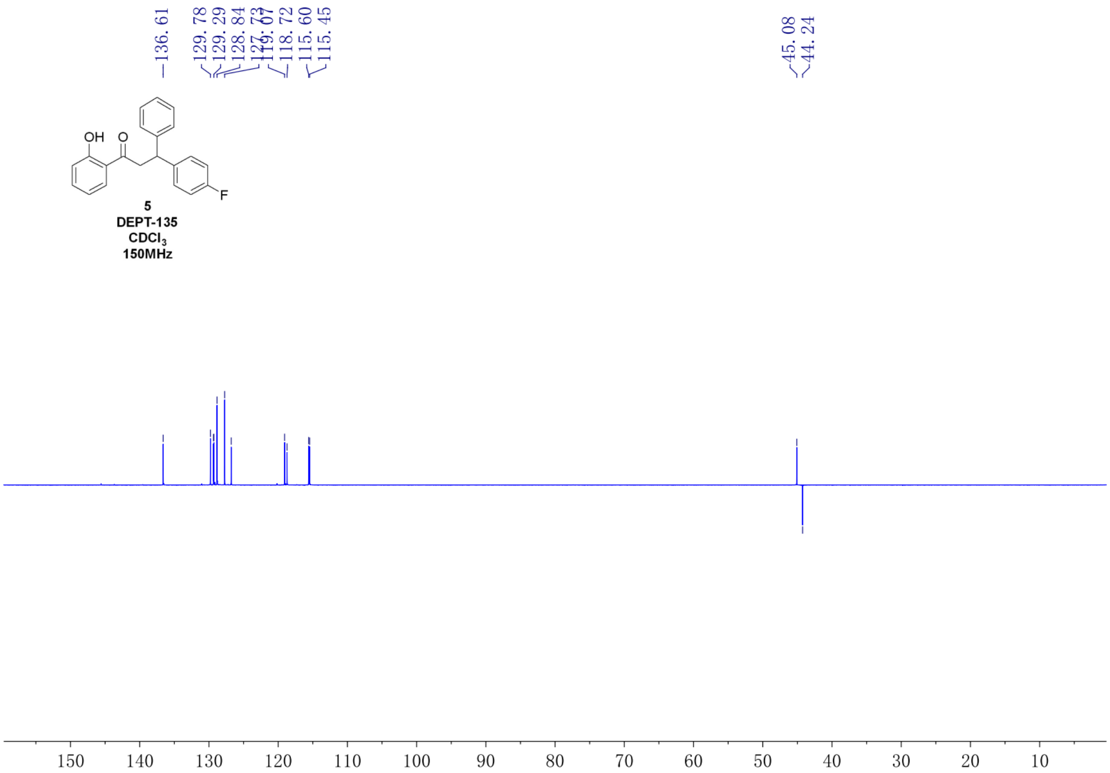
136.61
129.78
129.29
128.84
127.73
127.07
118.72
115.60
115.45
45.08
44.24
OH
O
F
5
DEPT-135
CDCl3
150MHz
150 140 130 120 110 100 90 80 70 60 50 40 30 20 10

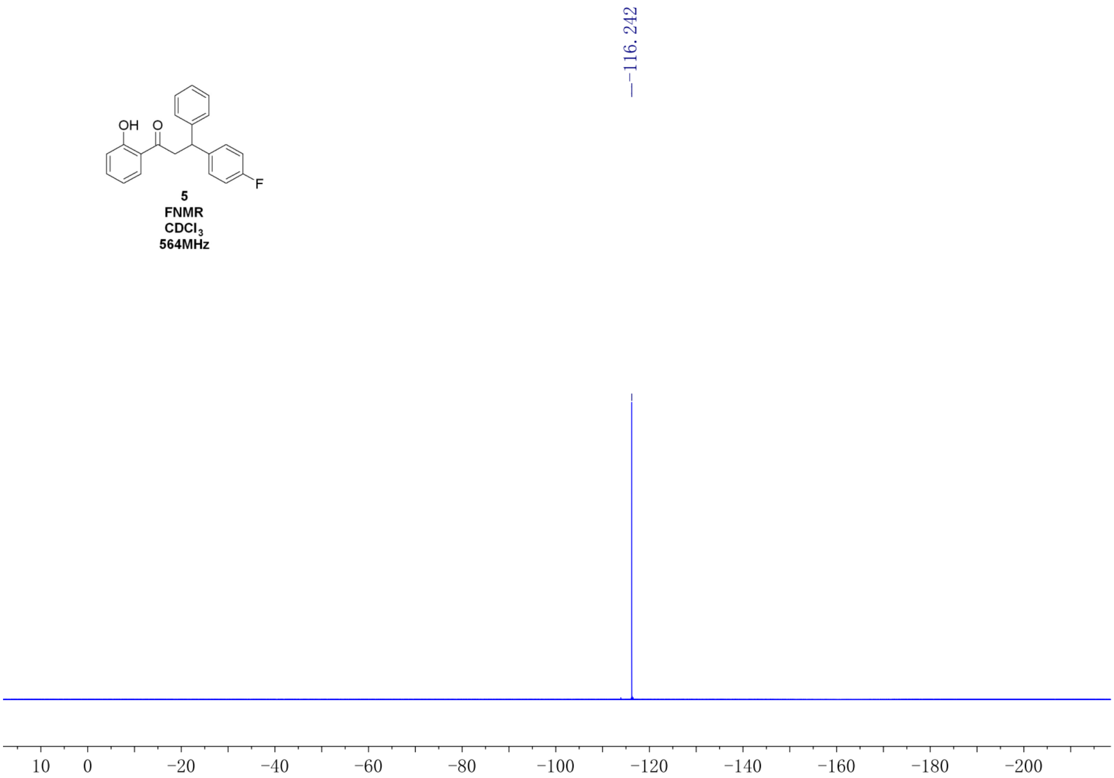
-116.242
5
FNMR
CDCl3
564MHz
10 0 -20 -40 -60 -80 -100 -120 -140 -160 -180 -200

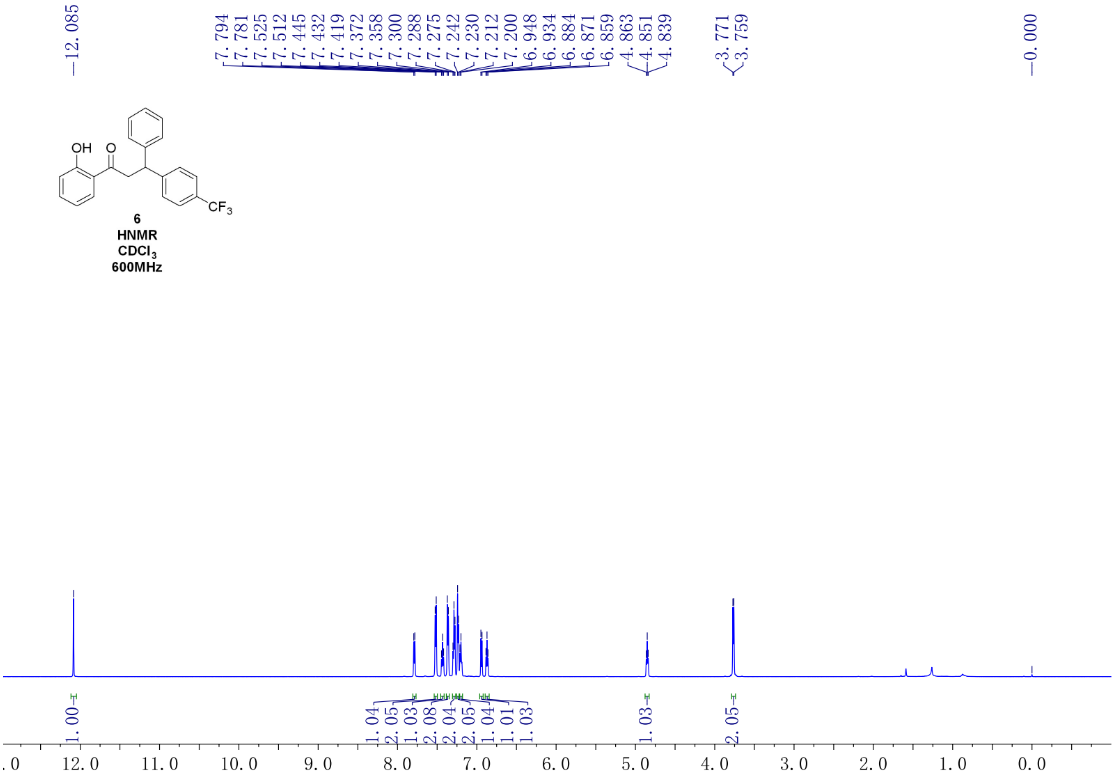
12.085
7.794 7.781 7.525 7.512 7.445 7.432 7.419 7.372 7.358 7.300 7.288 7.275 7.242 7.230 7.212 7.200 6.948 6.934 6.884 6.871 6.859
4.863 4.851 4.839
3.771 3.759
0.000
6
HNMR
CDCl3
600MHz
1.00 1.04 2.05 1.03 2.08 2.04 2.05 1.04 1.01 1.03 1.03 2.05
12.0 11.0 10.0 9.0 8.0 7.0 6.0 5.0 4.0 3.0 2.0 1.0 0.0

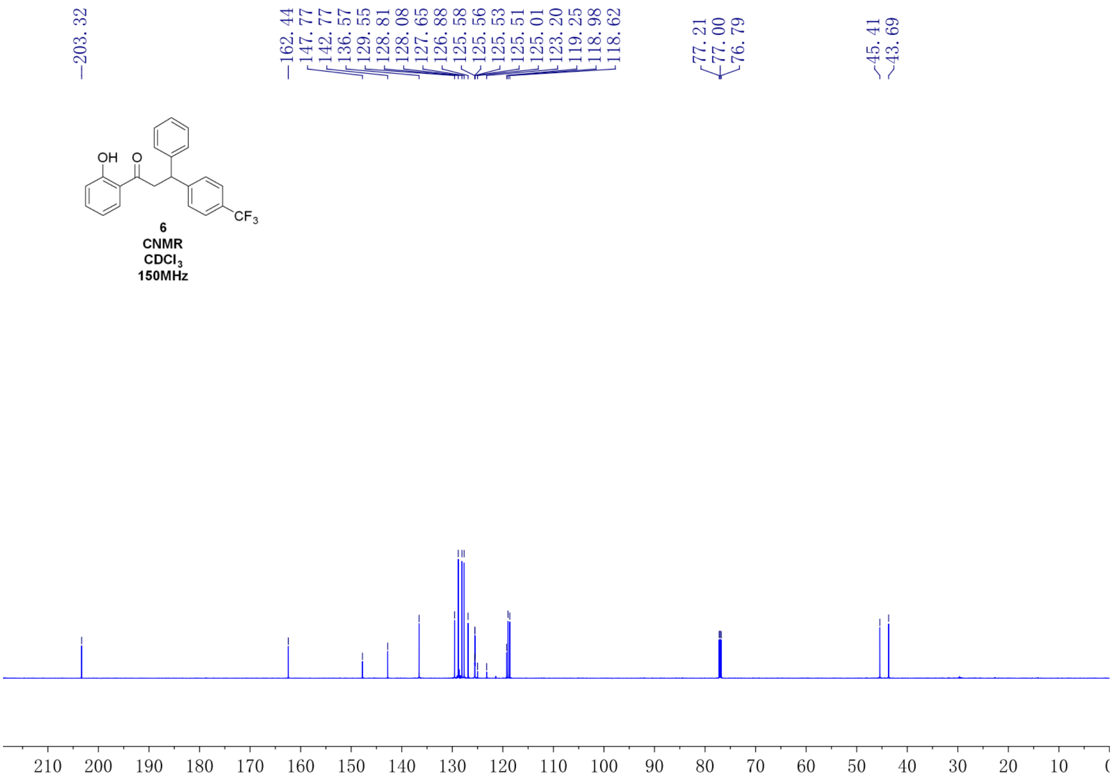
203.32
162.44
147.77
142.77
136.57
129.55
128.81
128.08
127.65
126.88
125.58
125.56
125.53
125.51
125.01
123.20
119.25
118.98
118.62
77.21
77.00
76.79
45.41
43.69
OH
O
CF3
6
CNMR
CDCl3
150MHz
210
200
190
180
170
160
150
140
130
120
110
100
90
80
70
60
50
40
30
20
10

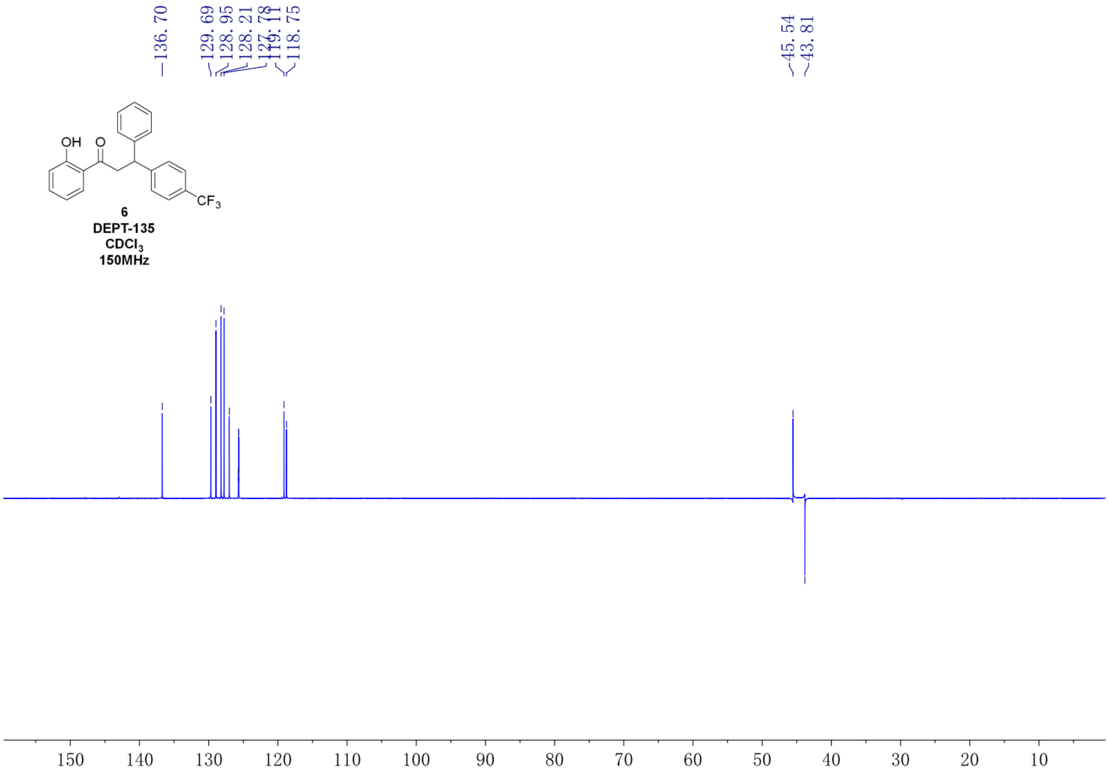
136.70
129.69
128.95
128.21
119.11
118.75
45.54
43.81
OH
O
CF3
6
DEPT-135
CDCl3
150MHz
150
140
130
120
110
100
90
80
70
60
50
40
30
20
10

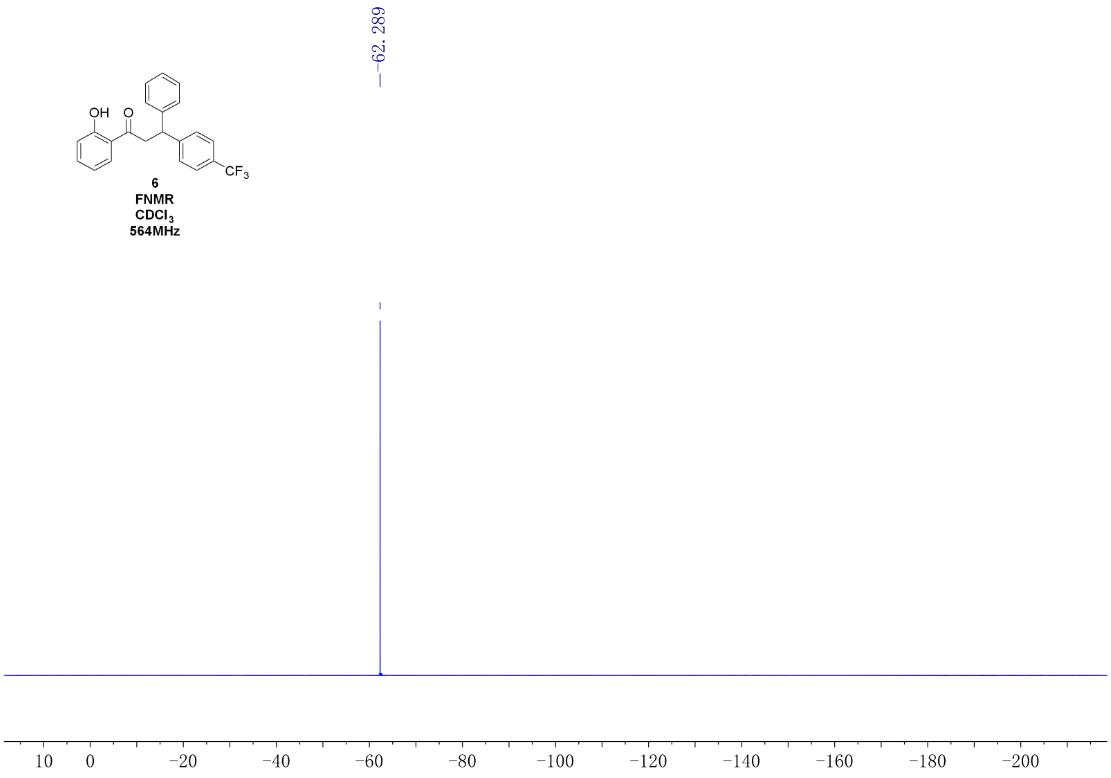
—62.289
OH
O
CF3
6
FNMR
CDCl3
564MHz
10
0
−20
−40
−60
−80
−100
−120
−140
−160
−180
−200

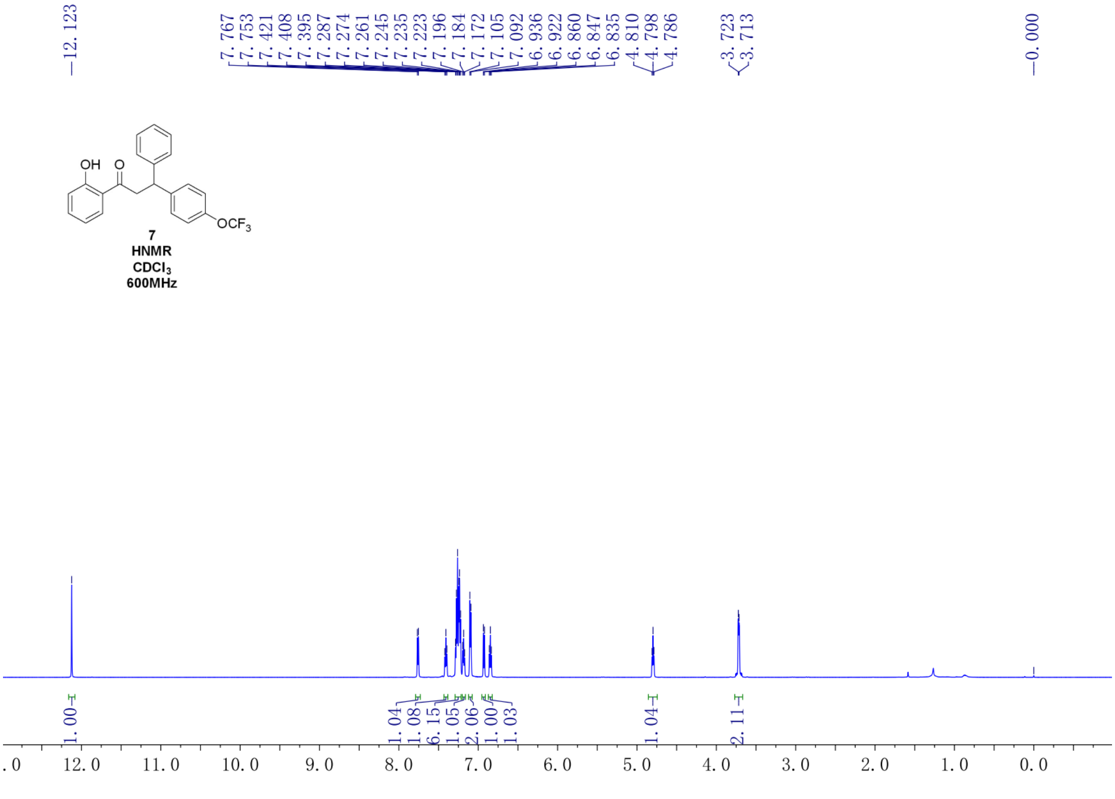
—12.123
7.767
7.753
7.421
7.408
7.395
7.287
7.274
7.261
7.245
7.235
7.223
7.196
7.184
7.172
7.105
7.092
6.936
6.922
6.860
6.847
6.835
4.810
4.798
4.786
3.723
3.713
—0.000
OH
O
OCF3
7
HNMR
CDCl3
600MHz
1.00
1.04
1.08
6.15
1.05
2.06
1.00
1.03
1.04
2.11
.0
12.0
11.0
10.0
9.0
8.0
7.0
6.0
5.0
4.0
3.0
2.0
1.0
0.0

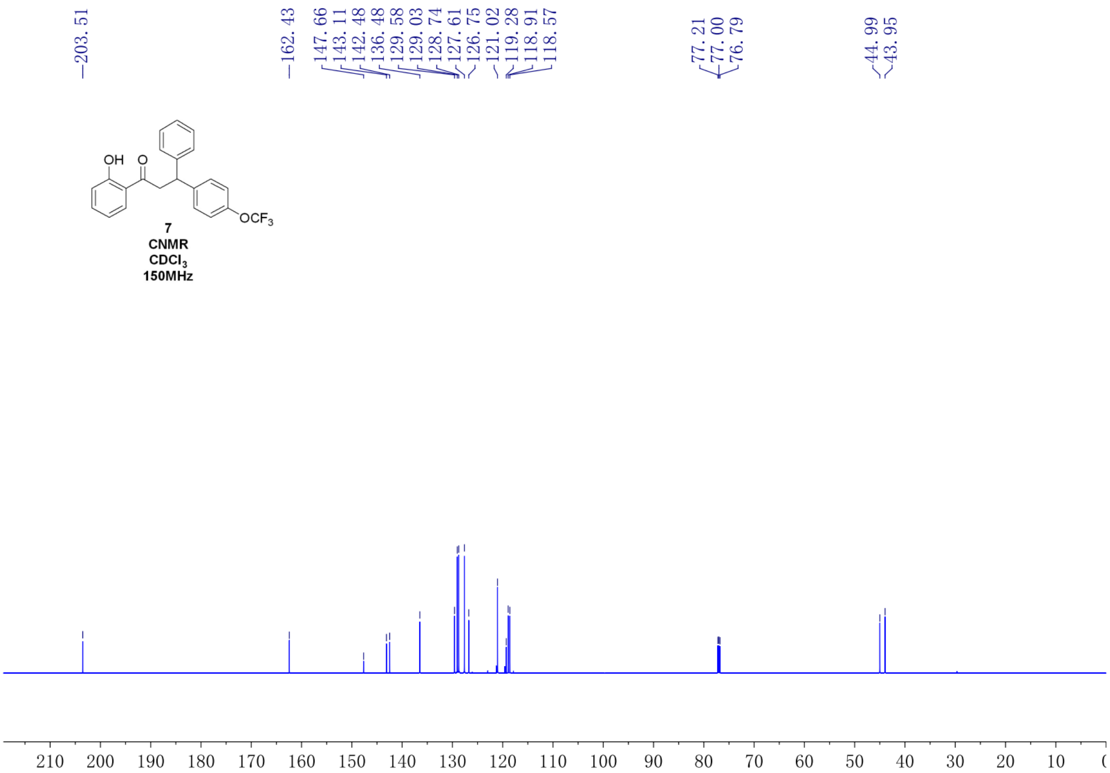
203.51
162.43
147.66
143.11
142.48
136.48
129.58
129.03
128.74
127.61
126.75
121.02
119.28
118.91
118.57
77.21
77.00
76.79
44.99
43.95
OH
O
OCF_3
7
CNMR
$CDCl_3$
150MHz
210
200
190
180
170
160
150
140
130
120
110
100
90
80
70
60
50
40
30
20
10

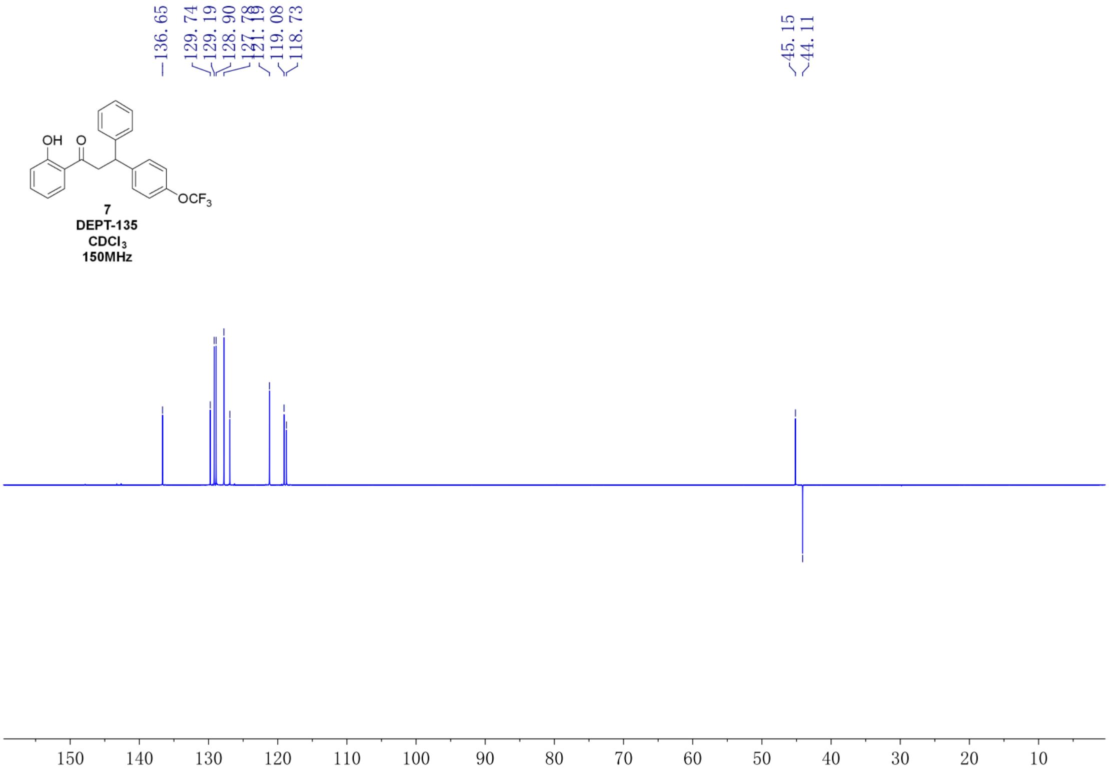
136.65
129.74
129.19
128.90
127.78
121.19
119.08
118.73
45.15
44.11
OH
O
OCF_3
7
DEPT-135
$CDCl_3$
150MHz
150
140
130
120
110
100
90
80
70
60
50
40
30
20
10

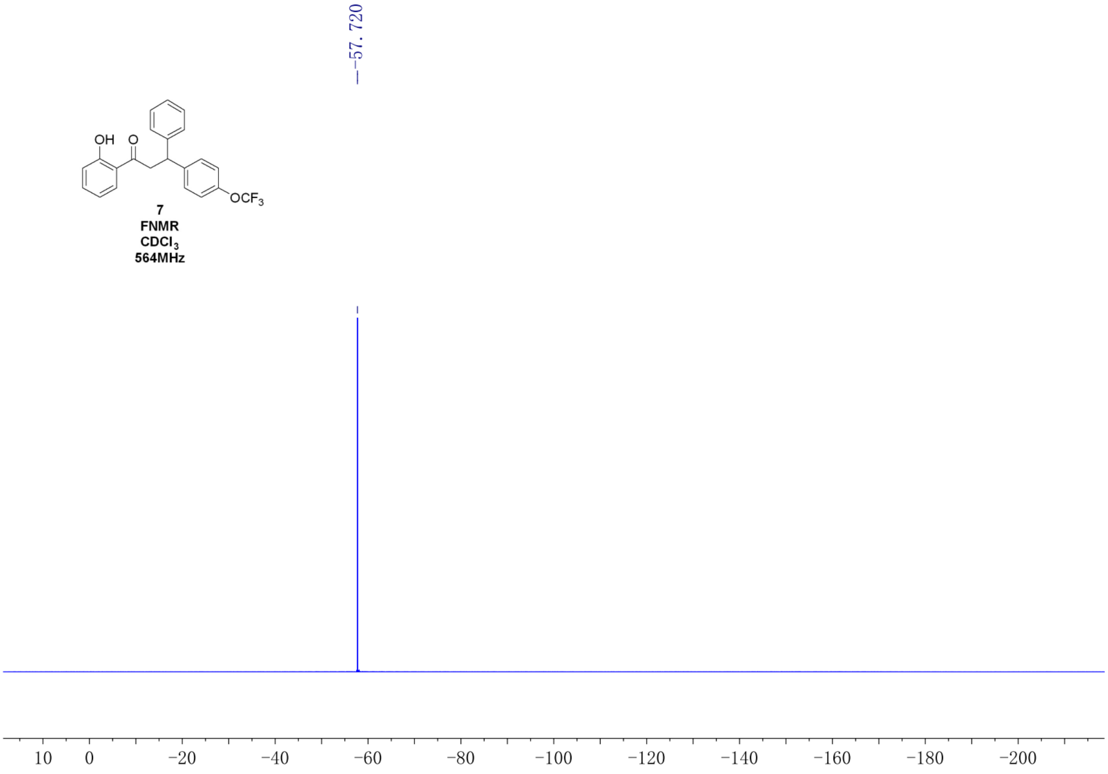
-57.720
OH O
OCF3
7
FNMR
CDCl3
564MHz
10 0 -20 -40 -60 -80 -100 -120 -140 -160 -180 -200

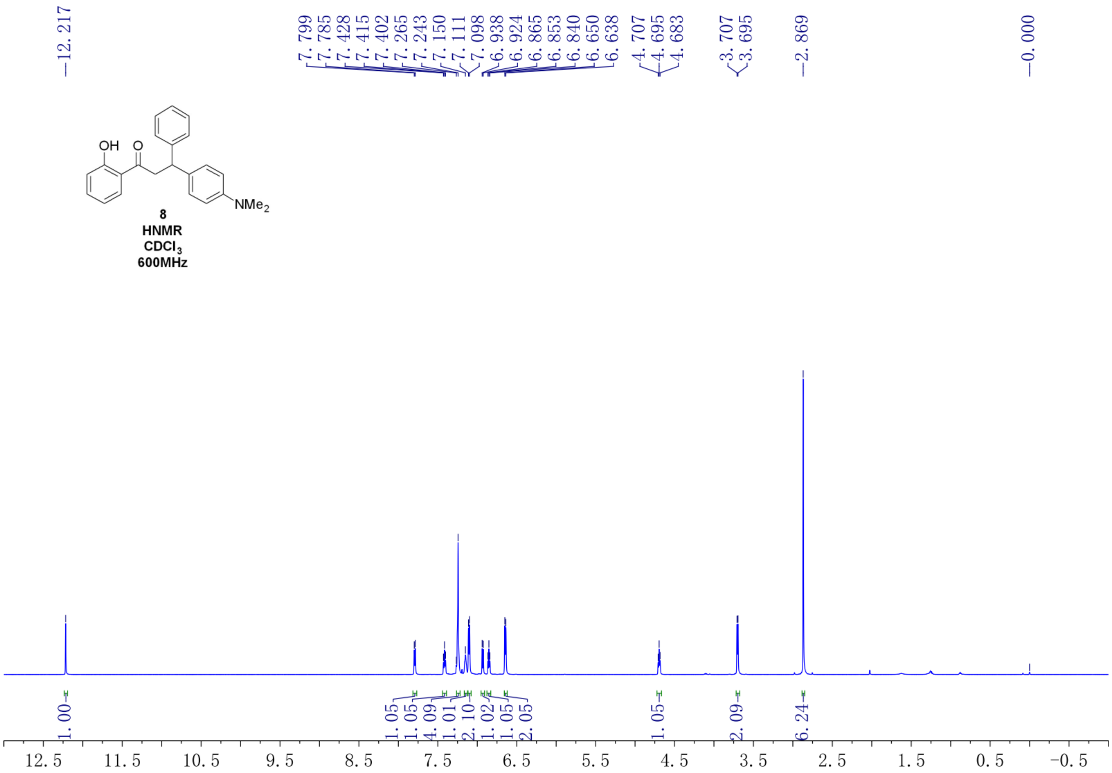
12.217
7.799
7.785
7.428
7.415
7.402
7.265
7.243
7.150
7.111
7.098
6.938
6.924
6.865
6.853
6.840
6.650
6.638
4.707
4.695
4.683
3.707
3.695
2.869
0.000
OH O
NMe2
8
HNMR
CDCl3
600MHz
1.00
1.05
1.05
4.09
1.01
2.10
1.02
1.05
2.05
1.05
2.09
6.24
12.5 11.5 10.5 9.5 8.5 7.5 6.5 5.5 4.5 3.5 2.5 1.5 0.5 -0.5

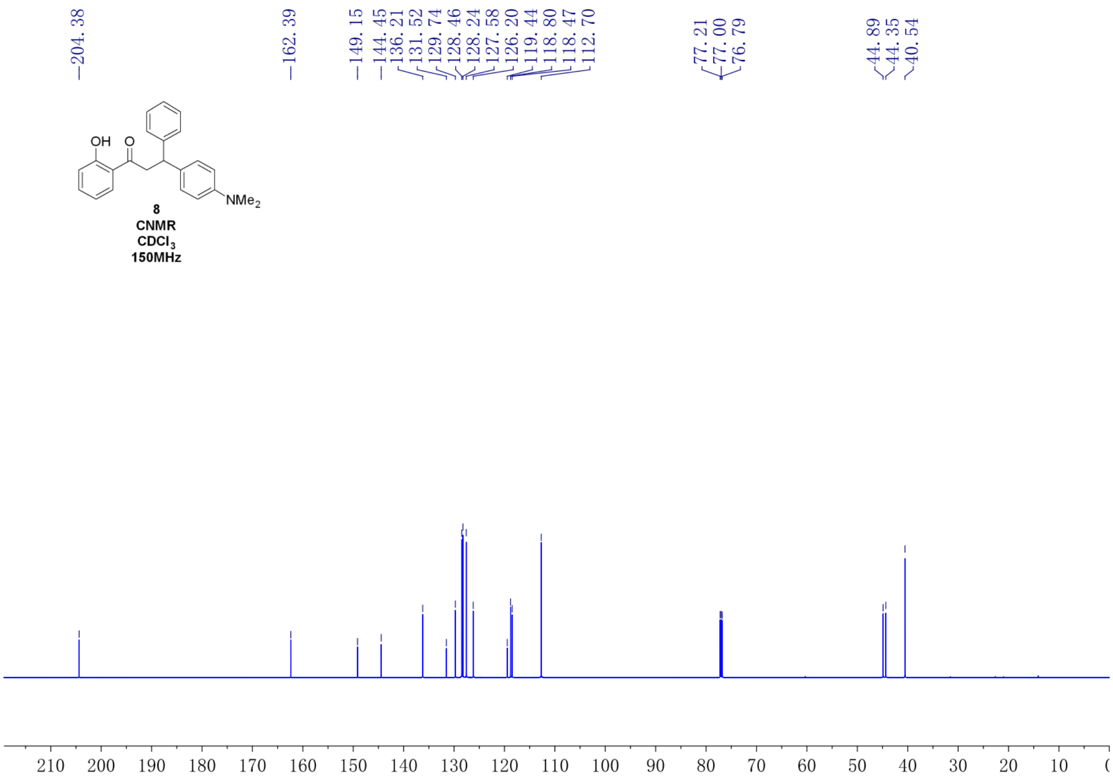
204.38
162.39
149.15
144.45
136.21
131.52
129.74
128.46
128.24
127.58
126.20
119.44
118.80
118.47
112.70
77.21
77.00
76.79
44.89
44.35
40.54
OH
O
NMe2
8
CNMR
CDCl3
150MHz
210 200 190 180 170 160 150 140 130 120 110 100 90 80 70 60 50 40 30 20 10

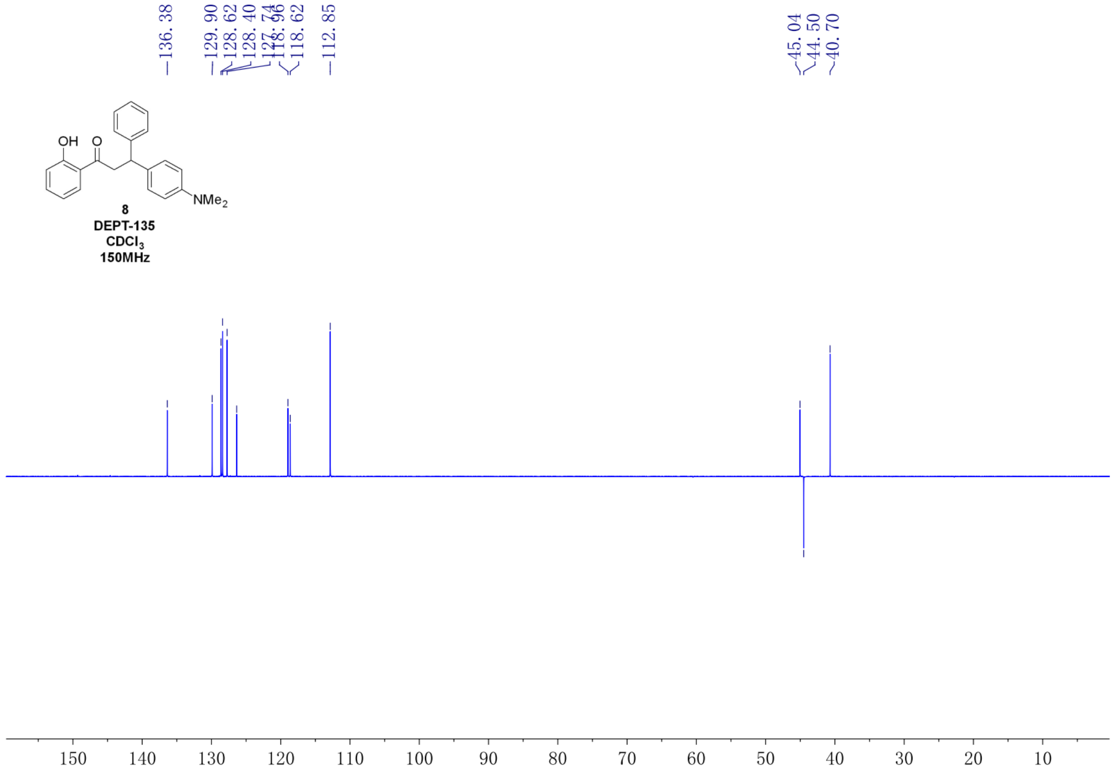
136.38
129.90
128.62
128.40
127.74
118.96
118.62
112.85
45.04
44.50
40.70
OH
O
NMe2
8
DEPT-135
CDCl3
150MHz
150 140 130 120 110 100 90 80 70 60 50 40 30 20 10

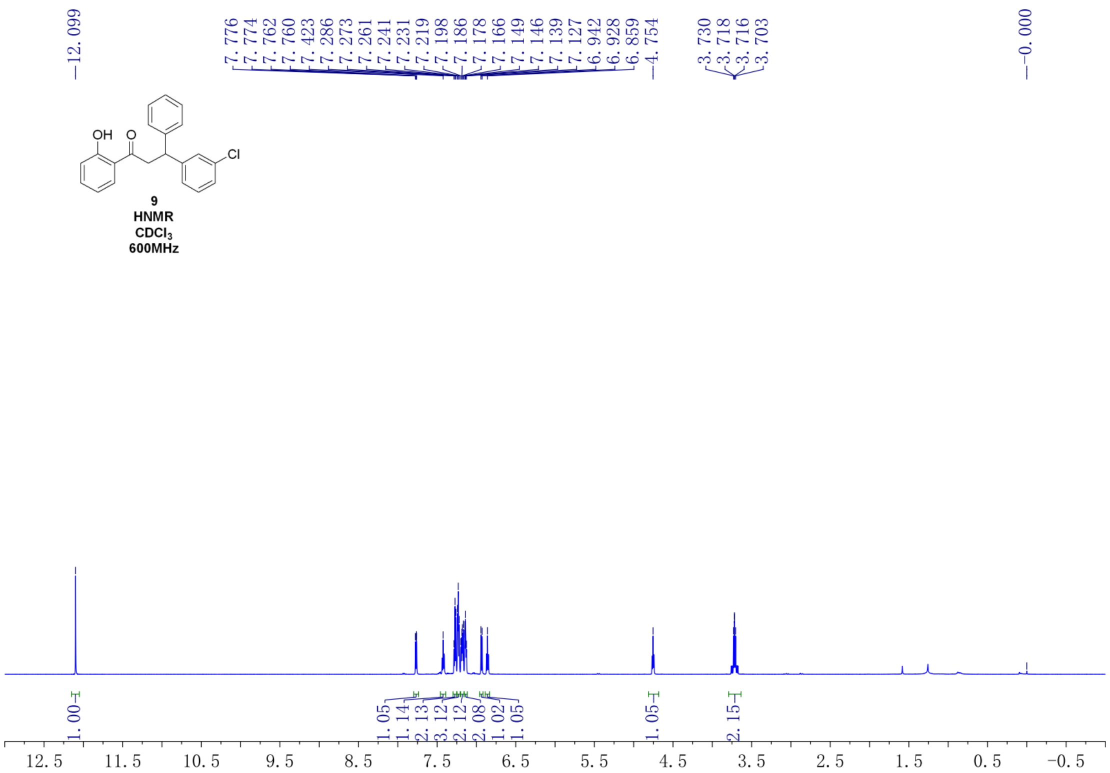
12.099
7.776 7.774 7.762 7.760 7.423 7.286 7.273 7.261 7.241 7.231 7.219 7.198 7.186 7.178 7.166 7.149 7.146 7.139 7.127 6.942 6.928 6.859 4.754
3.730 3.718 3.716 3.703
0.000
OH
O
Cl
9
HNMR
$CDCl_3$
600MHz
1.00
1.05 1.14 2.13 3.12 2.12 2.08 1.02 1.05
1.05
2.15
12.5 11.5 10.5 9.5 8.5 7.5 6.5 5.5 4.5 3.5 2.5 1.5 0.5 -0.5

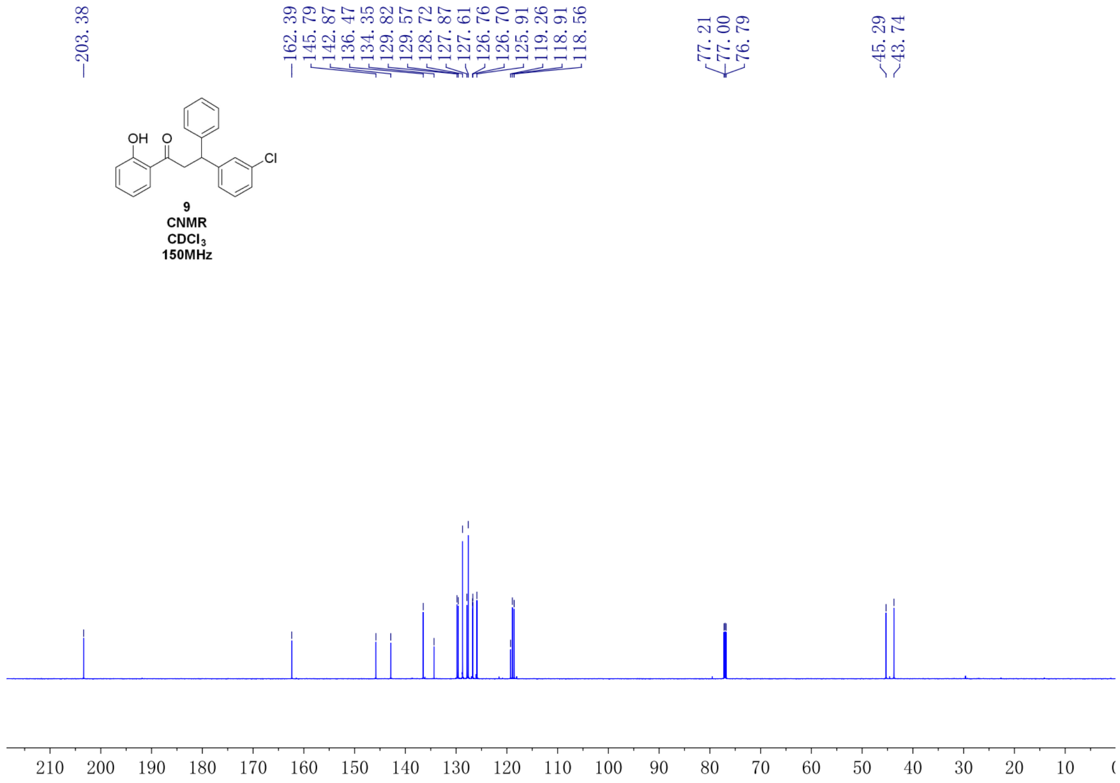
203.38
162.39 145.79 142.87 136.47 134.35 129.82 129.57 128.72 127.87 127.61 126.76 126.70 125.91 119.26 118.91 118.56
77.21 77.00 76.79
45.29 43.74
OH
O
Cl
9
CNMR
$CDCl_3$
150MHz
210 200 190 180 170 160 150 140 130 120 110 100 90 80 70 60 50 40 30 20 10

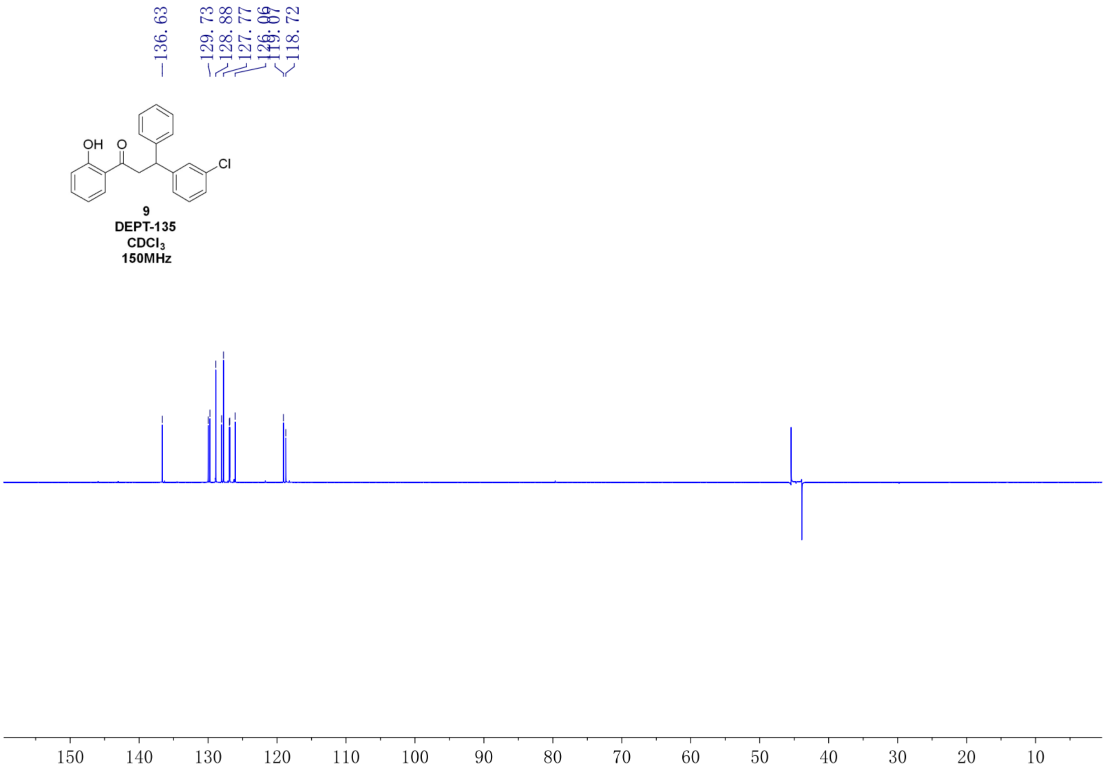
136.63
129.73
128.88
127.77
118.72
OH
O
Cl
9
DEPT-135
CDCl3
150MHz
150
140
130
120
110
100
90
80
70
60
50
40
30
20
10

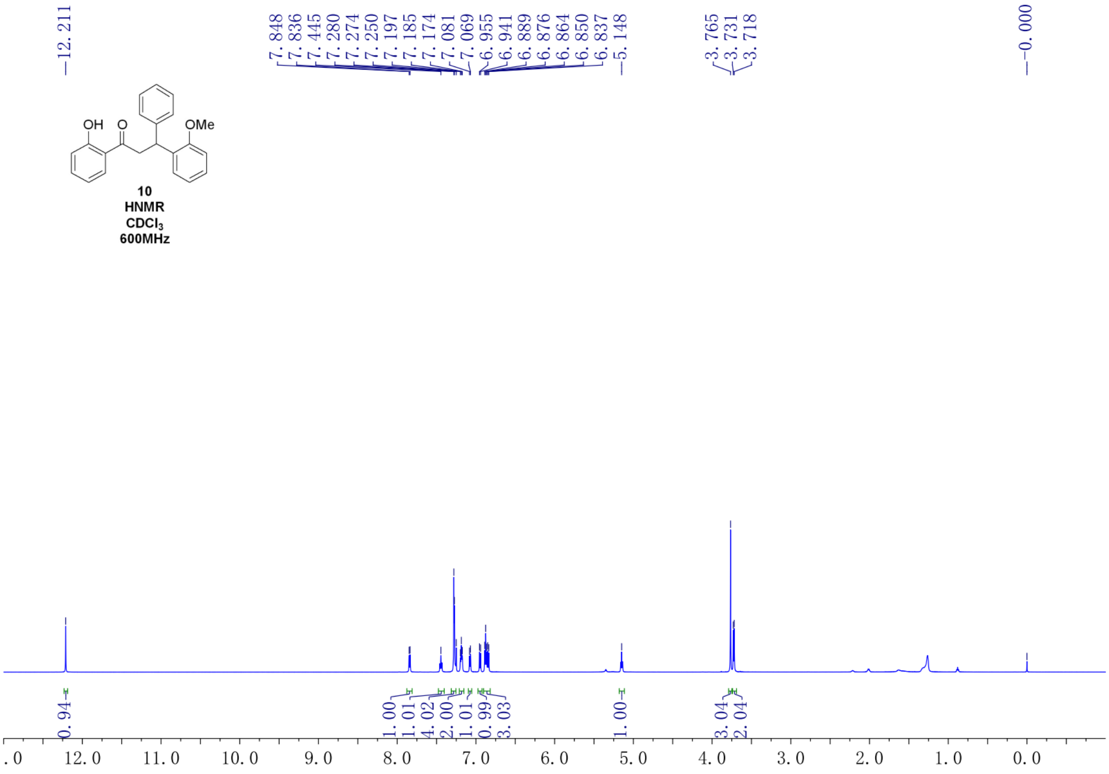
12.211
7.848
7.836
7.445
7.280
7.274
7.250
7.197
7.185
7.174
7.081
7.069
6.955
6.941
6.889
6.876
6.864
6.850
6.837
5.148
3.765
3.731
3.718
0.000
OH
O
OMe
10
HNMR
CDCl3
600MHz
0.94
1.00
1.01
4.02
2.00
1.01
0.99
3.03
1.00
3.04
2.04
12.0
11.0
10.0
9.0
8.0
7.0
6.0
5.0
4.0
3.0
2.0
1.0
0.0

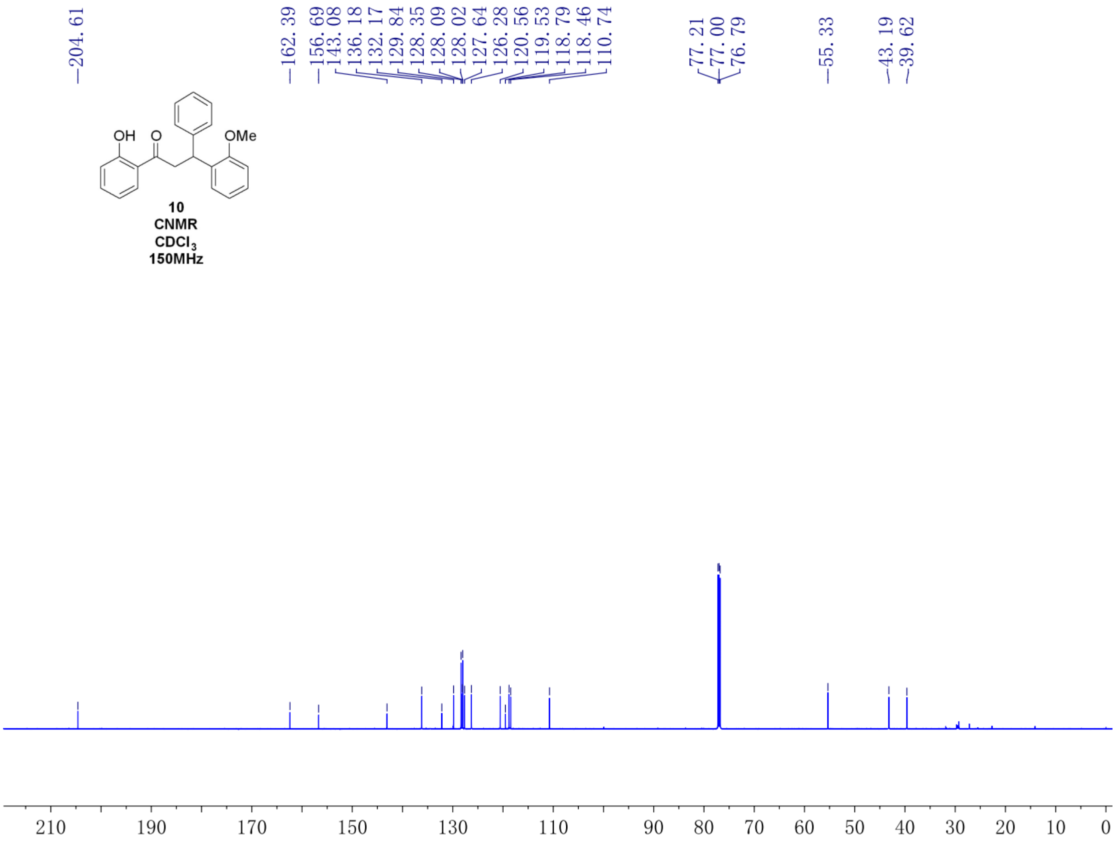
204.61
162.39
156.69
143.08
136.18
132.17
129.84
128.35
128.09
128.02
127.64
126.28
120.56
119.53
118.79
118.46
110.74
77.21
77.00
76.79
55.33
43.19
39.62
OH
O
OMe
10
CNMR
CDCl3
150MHz
210
190
170
150
130
110
90
80
70
60
50
40
30
20
10
0

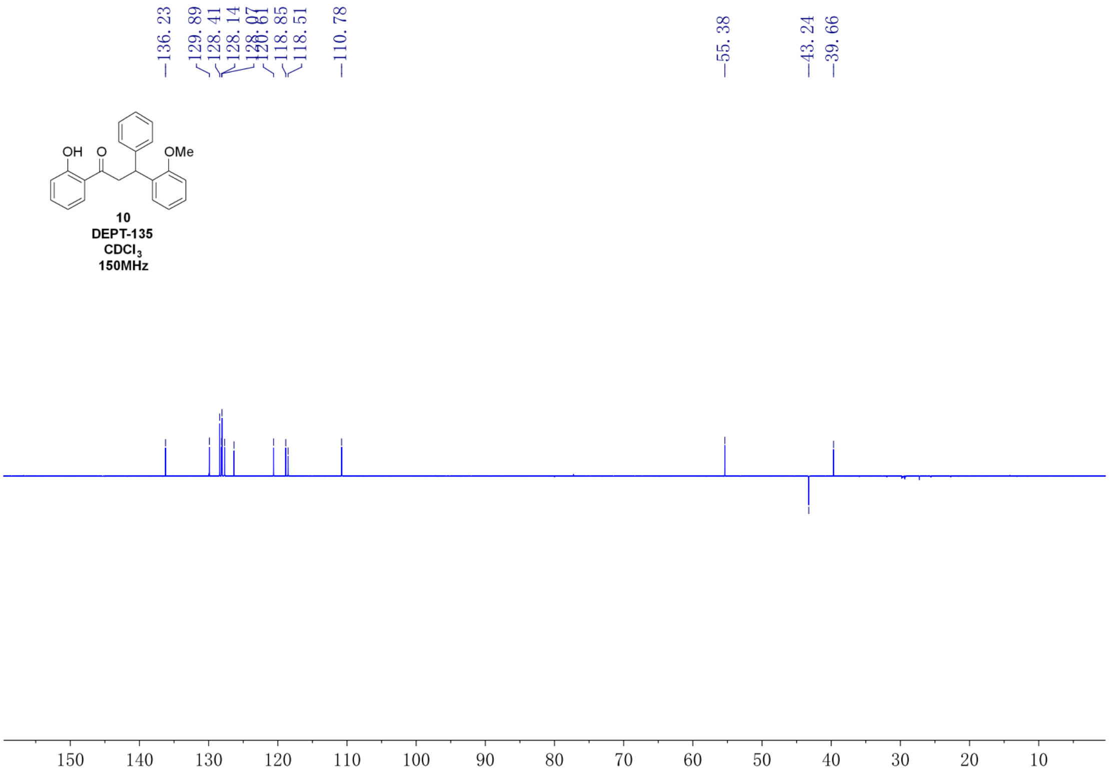
136.23
129.89
128.41
128.14
128.07
120.61
118.85
118.51
110.78
55.38
43.24
39.66
OH
O
OMe
10
DEPT-135
CDCl3
150MHz
150
140
130
120
110
100
90
80
70
60
50
40
30
20
10

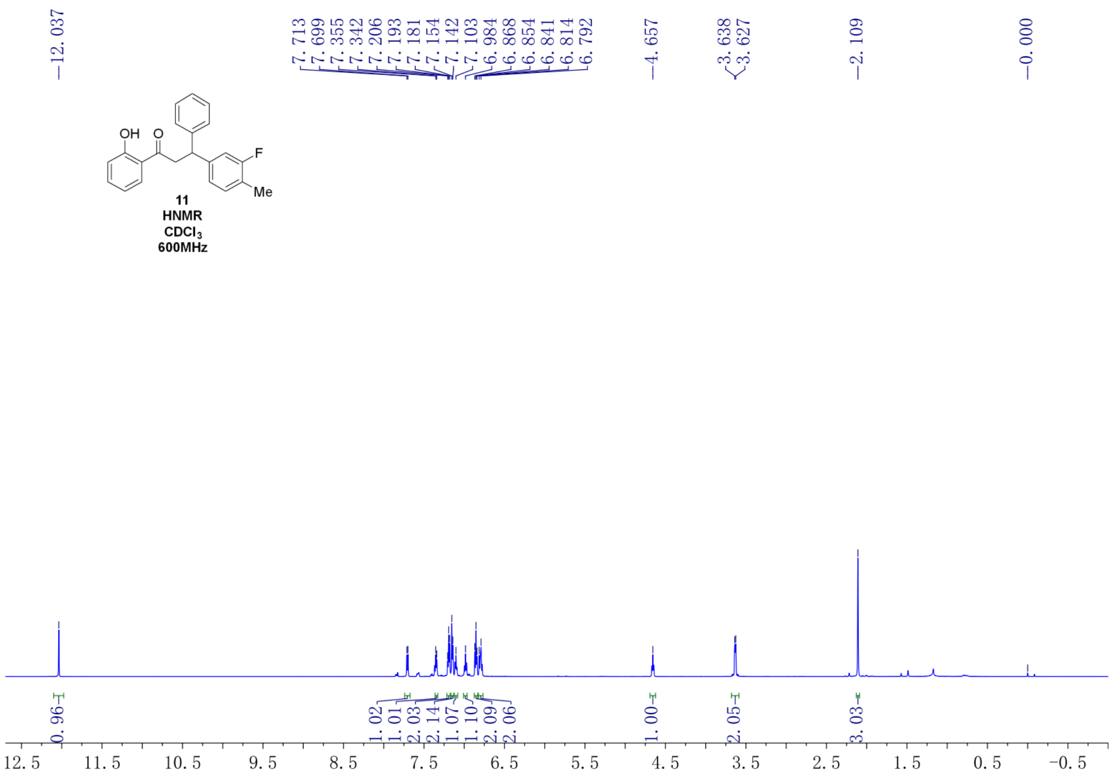
12.037
7.713
7.699
7.355
7.342
7.206
7.193
7.181
7.154
7.142
7.103
6.984
6.868
6.854
6.841
6.814
6.792
4.657
3.638
3.627
2.109
0.000
OH
O
F
Me
11
HNMR
$CDCl_3$
600MHz
0.96
1.02
1.01
2.03
2.14
1.07
1.10
2.09
2.06
1.00
2.05
3.03
12.5
11.5
10.5
9.5
8.5
7.5
6.5
5.5
4.5
3.5
2.5
1.5
0.5
-0.5

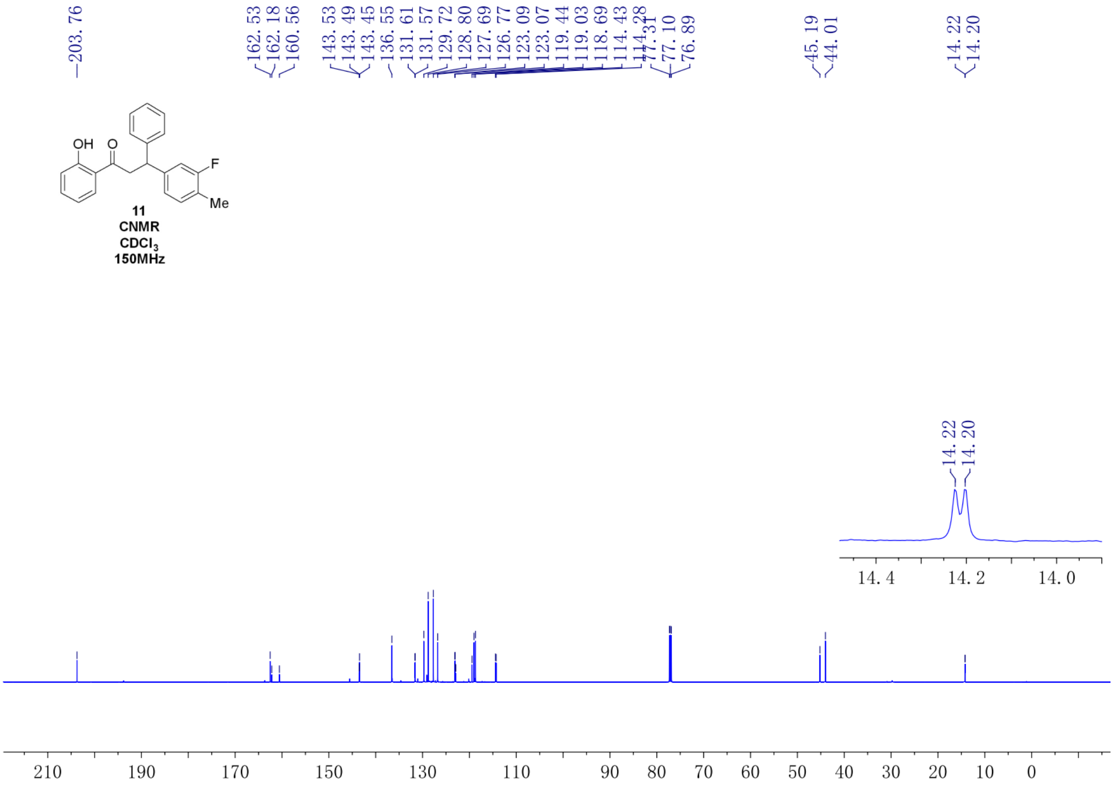
203.76
162.53
162.18
160.56
143.53
143.49
143.45
136.55
131.61
131.57
129.72
128.80
127.69
126.77
123.09
123.07
119.44
119.03
118.69
114.43
114.28
77.31
77.10
76.89
45.19
44.01
14.22
14.20
OH
O
F
Me
11
CNMR
$CDCl_3$
150MHz
14.22
14.20
14.4
14.2
14.0
210
190
170
150
130
110
90
80
70
60
50
40
30
20
10
0

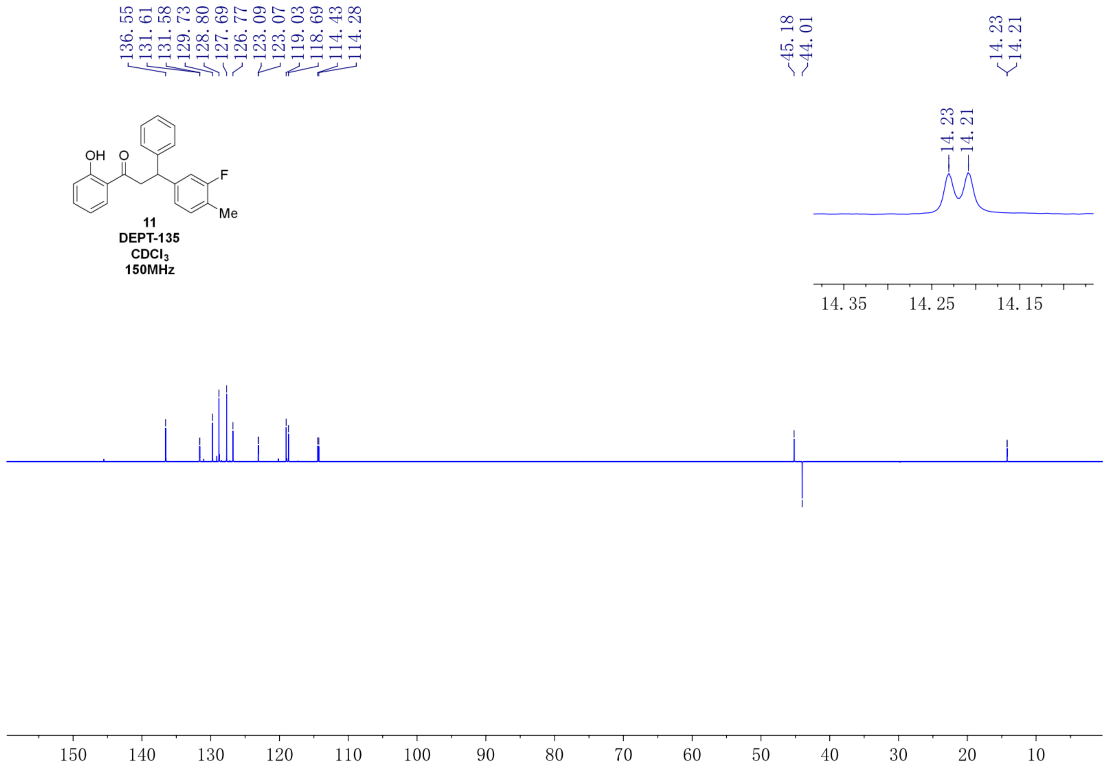
136.55
131.61
131.58
129.73
128.80
127.69
126.77
123.09
123.07
119.03
118.69
114.43
114.28
45.18
44.01
14.23
14.21
OH
O
F
Me
11
DEPT-135
$CDCl_3$
150MHz
14.23
14.21
14.35
14.25
14.15
150
140
130
120
110
100
90
80
70
60
50
40
30
20
10

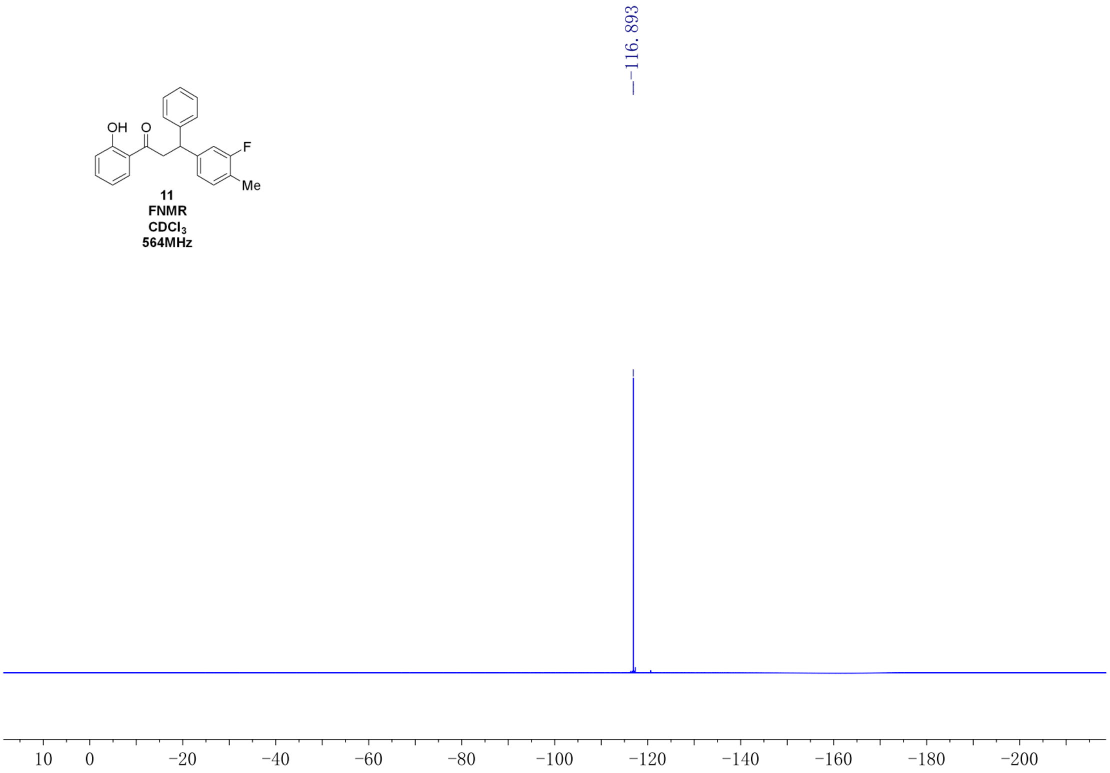
−116.893
OH
O
F
Me
11
FNMR
$CDCl_3$
564MHz
10
0
−20
−40
−60
−80
−100
−120
−140
−160
−180
−200

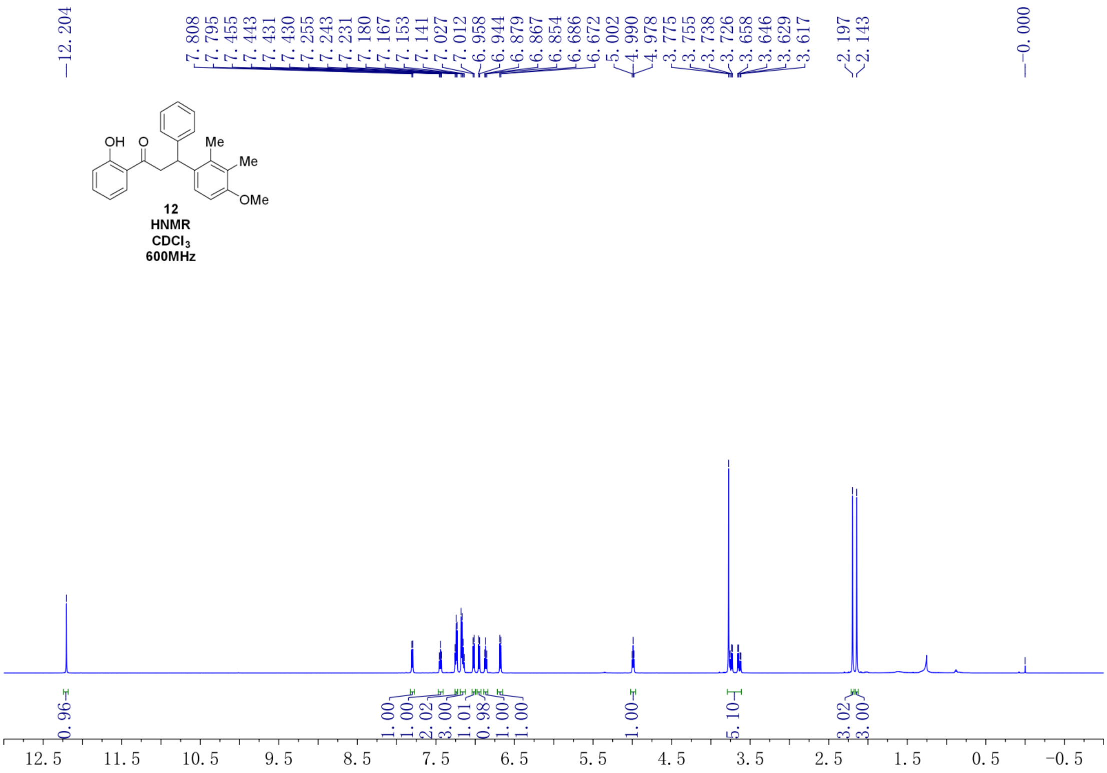
12.204
7.808
7.795
7.455
7.443
7.431
7.430
7.255
7.243
7.231
7.180
7.167
7.153
7.141
7.027
7.012
6.958
6.944
6.879
6.867
6.854
6.686
6.672
5.002
4.990
4.978
3.775
3.755
3.738
3.726
3.658
3.646
3.629
3.617
2.197
2.143
0.000
OH
O
Me
Me
OMe
12
HNMR
$CDCl_3$
600MHz
0.96
1.00
1.00
2.02
3.00
1.01
0.98
1.00
1.00
1.00
5.10
3.02
3.00
12.5
11.5
10.5
9.5
8.5
7.5
6.5
5.5
4.5
3.5
2.5
1.5
0.5
-0.5

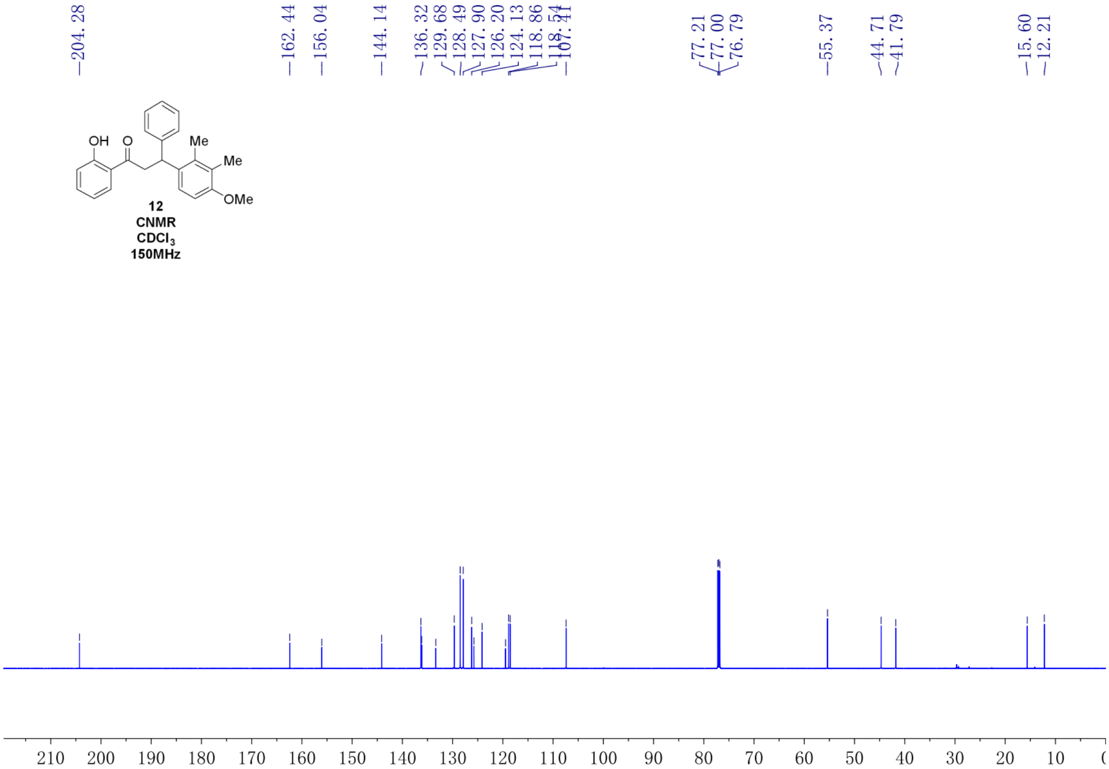
204.28
162.44
156.04
144.14
136.32
129.68
128.49
127.90
126.20
124.13
118.86
118.54
107.41
77.21
77.00
76.79
55.37
44.71
41.79
15.60
12.21
OH
O
Me
Me
OMe
12
CNMR
$CDCl_3$
150MHz
210
200
190
180
170
160
150
140
130
120
110
100
90
80
70
60
50
40
30
20
10

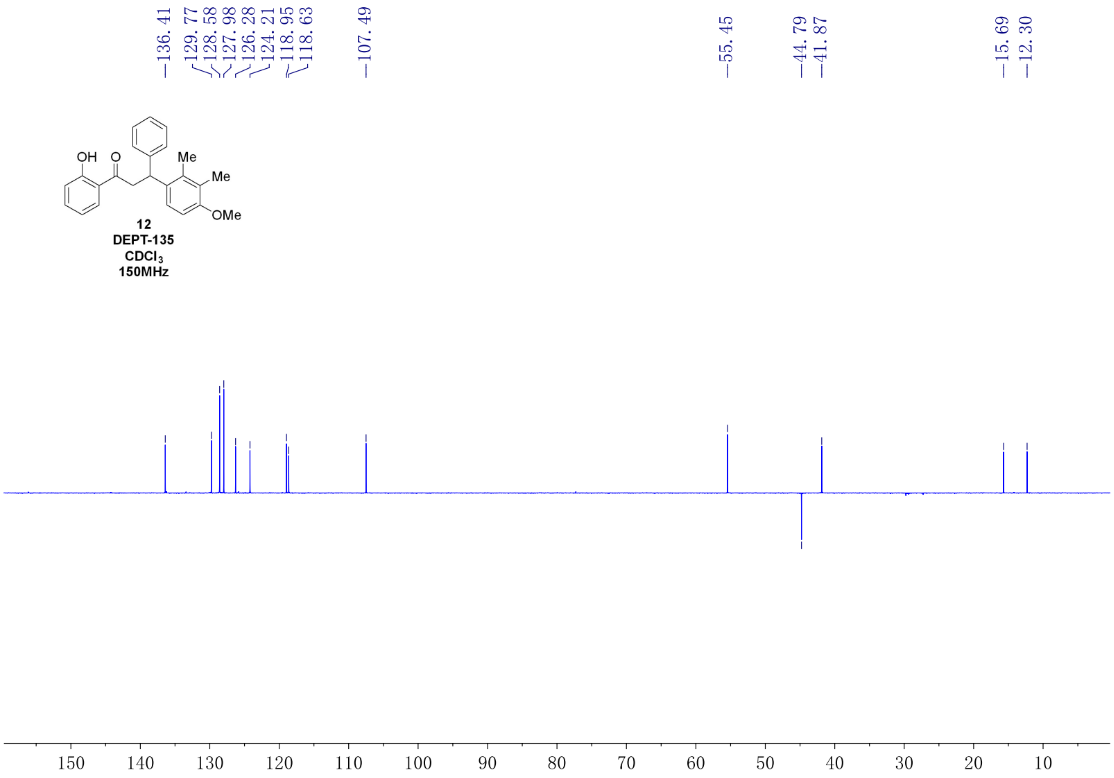
136.41
129.77
128.58
127.98
126.28
124.21
118.95
118.63
107.49
55.45
44.79
41.87
15.69
12.30
OH
O
Me
Me
OMe
12
DEPT-135
CDCl3
150MHz
150 140 130 120 110 100 90 80 70 60 50 40 30 20 10

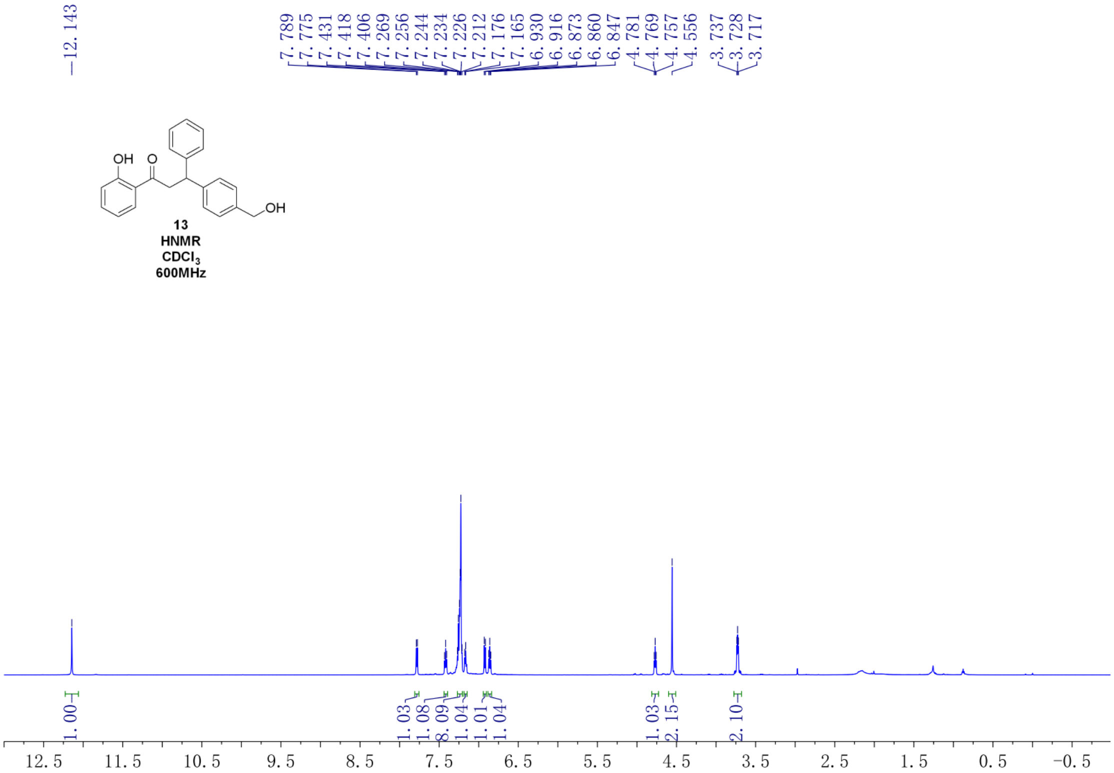
12.143
7.789
7.775
7.431
7.418
7.406
7.269
7.256
7.244
7.234
7.226
7.212
7.176
7.165
6.930
6.916
6.873
6.860
6.847
4.781
4.769
4.757
4.556
3.737
3.728
3.717
OH
O
OH
13
HNMR
CDCl3
600MHz
1.00
1.03
1.08
8.09
1.04
1.01
1.04
1.03
2.15
2.10
12.5 11.5 10.5 9.5 8.5 7.5 6.5 5.5 4.5 3.5 2.5 1.5 0.5 -0.5

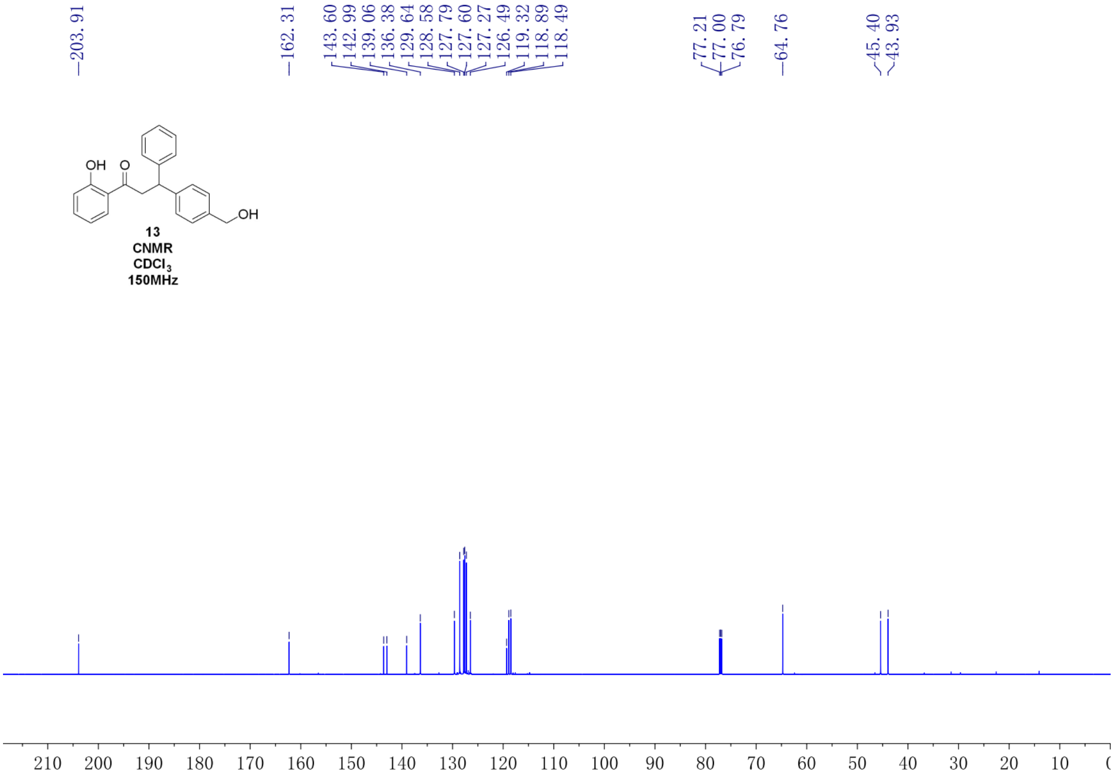
203.91
162.31
143.60
142.99
139.06
136.38
129.64
128.58
127.79
127.60
127.27
126.49
119.32
118.89
118.49
77.21
77.00
76.79
64.76
45.40
43.93
OH
O
OH
13
CNMR
CDCl3
150MHz
210 200 190 180 170 160 150 140 130 120 110 100 90 80 70 60 50 40 30 20 10

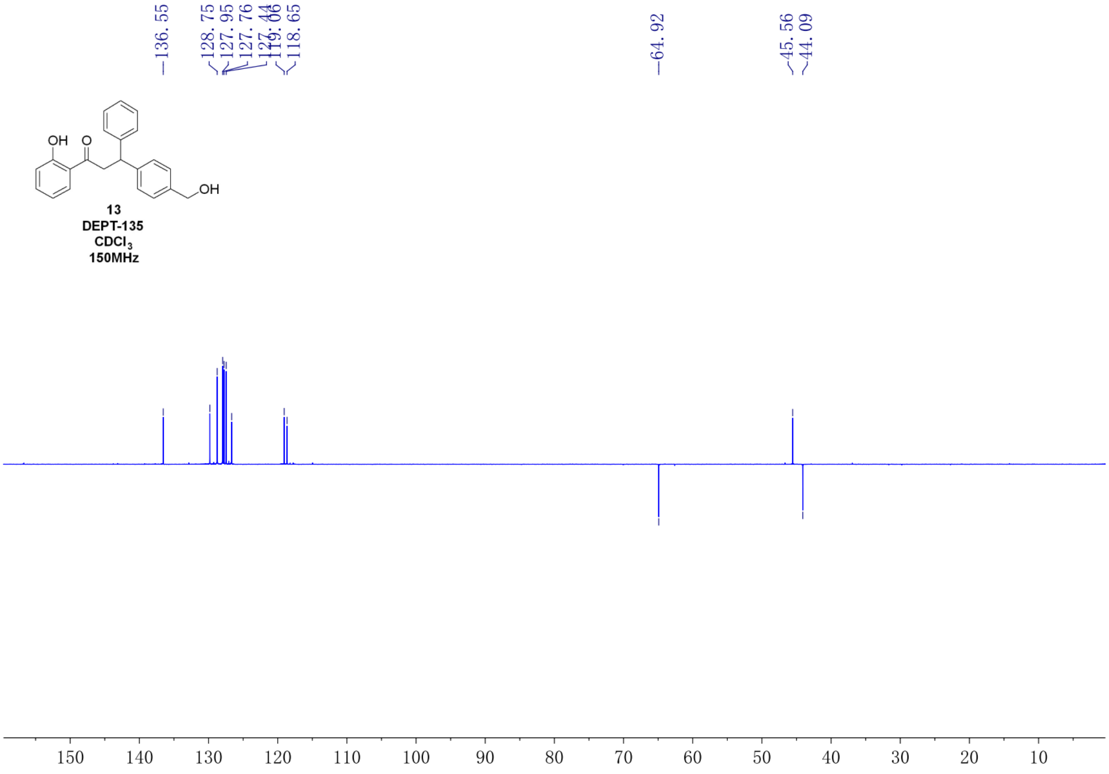
136.55
128.75
127.95
127.76
127.44
119.06
118.65
64.92
45.56
44.09
OH
O
OH
13
DEPT-135
CDCl3
150MHz
150 140 130 120 110 100 90 80 70 60 50 40 30 20 10

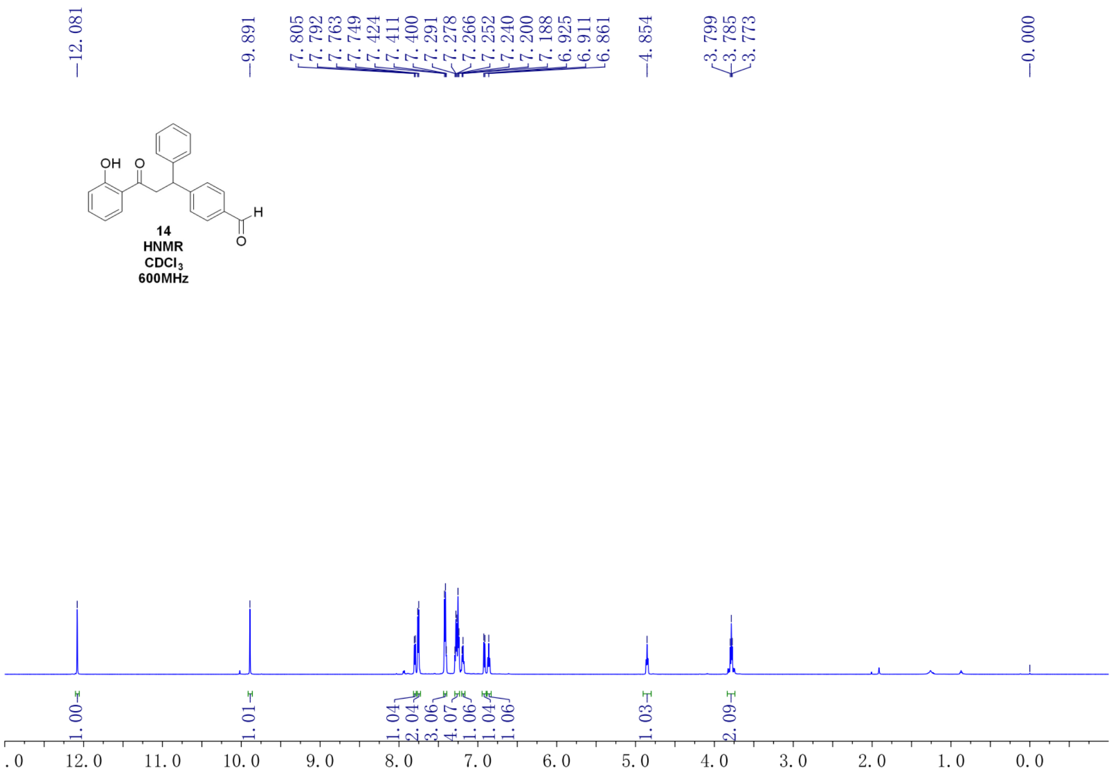
12.081
9.891
7.805
7.792
7.763
7.749
7.424
7.411
7.400
7.291
7.278
7.266
7.252
7.240
7.200
7.188
6.925
6.911
6.861
4.854
3.799
3.785
3.773
0.000
OH
O
H
O
14
HNMR
CDCl3
600MHz
1.00
1.01
1.04
2.04
3.06
4.07
1.06
1.04
1.06
1.03
2.09
12.0
11.0
10.0
9.0
8.0
7.0
6.0
5.0
4.0
3.0
2.0
1.0
0.0

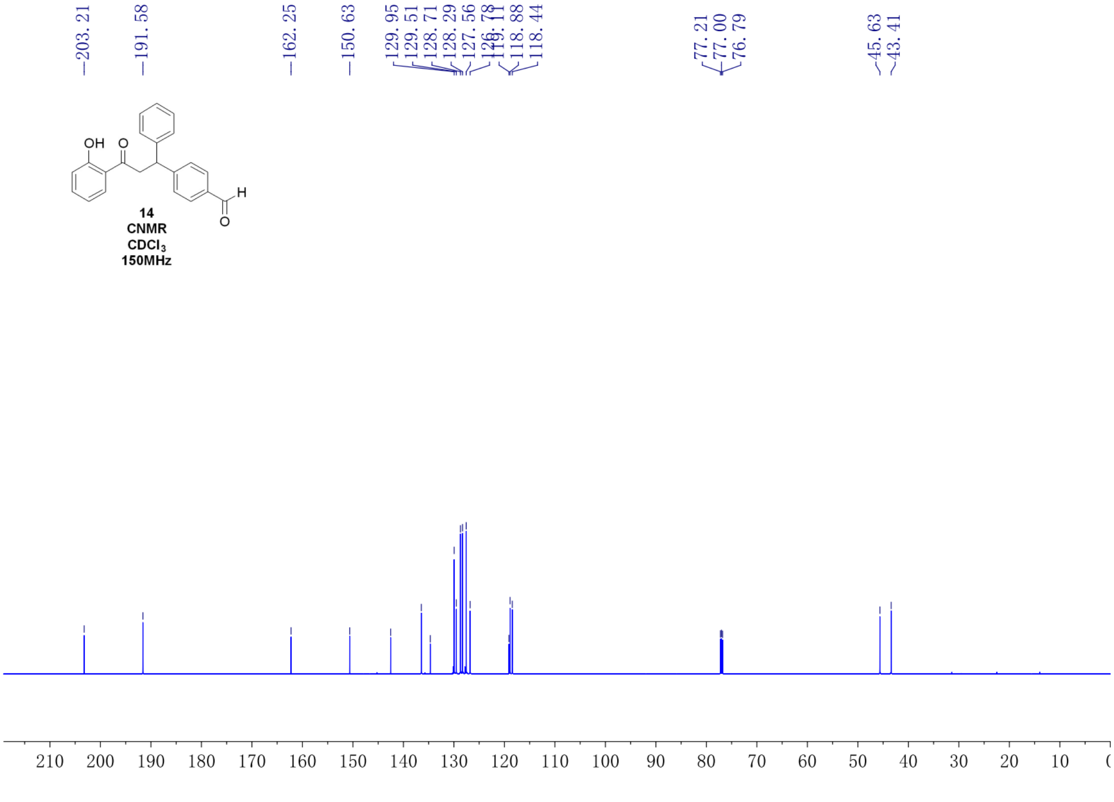
203.21
191.58
162.25
150.63
129.95
129.51
128.71
128.29
127.56
126.78
119.11
118.88
118.44
77.21
77.00
76.79
45.63
43.41
OH
O
H
O
14
CNMR
CDCl3
150MHz
210
200
190
180
170
160
150
140
130
120
110
100
90
80
70
60
50
40
30
20
10

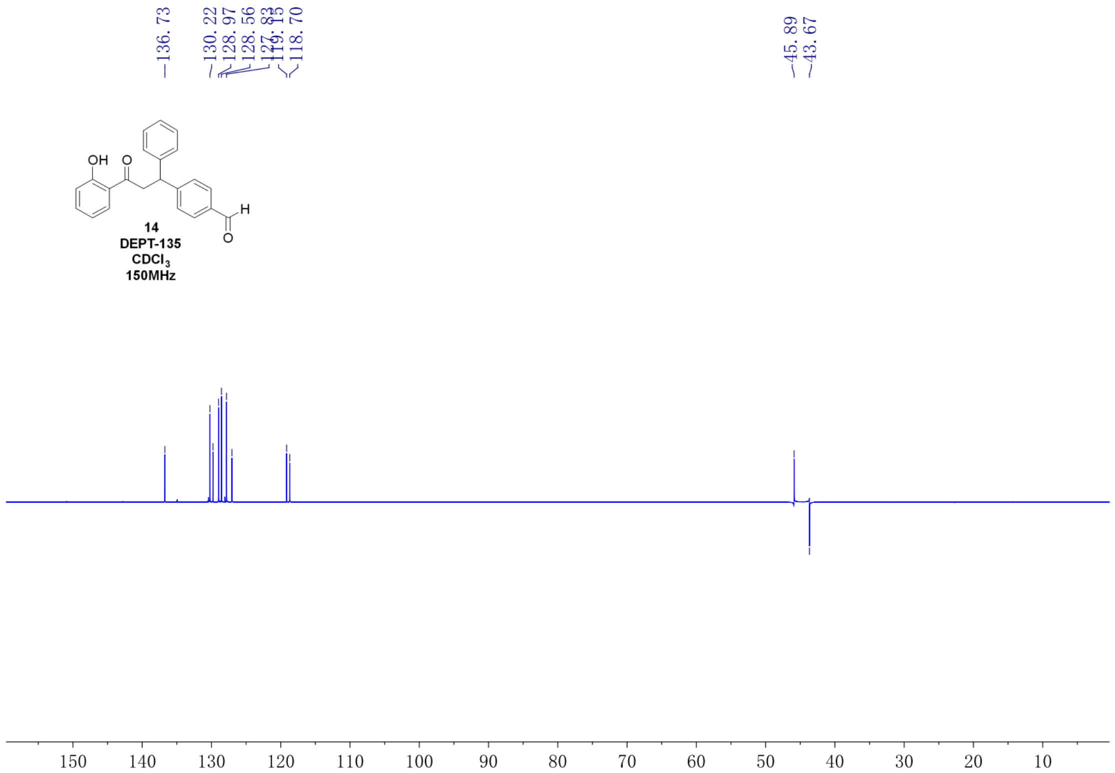
136.73
130.22
128.97
128.56
127.83
119.15
118.70
45.89
43.67
OH
O
H
O
14
DEPT-135
CDCl3
150MHz
150
140
130
120
110
100
90
80
70
60
50
40
30
20
10

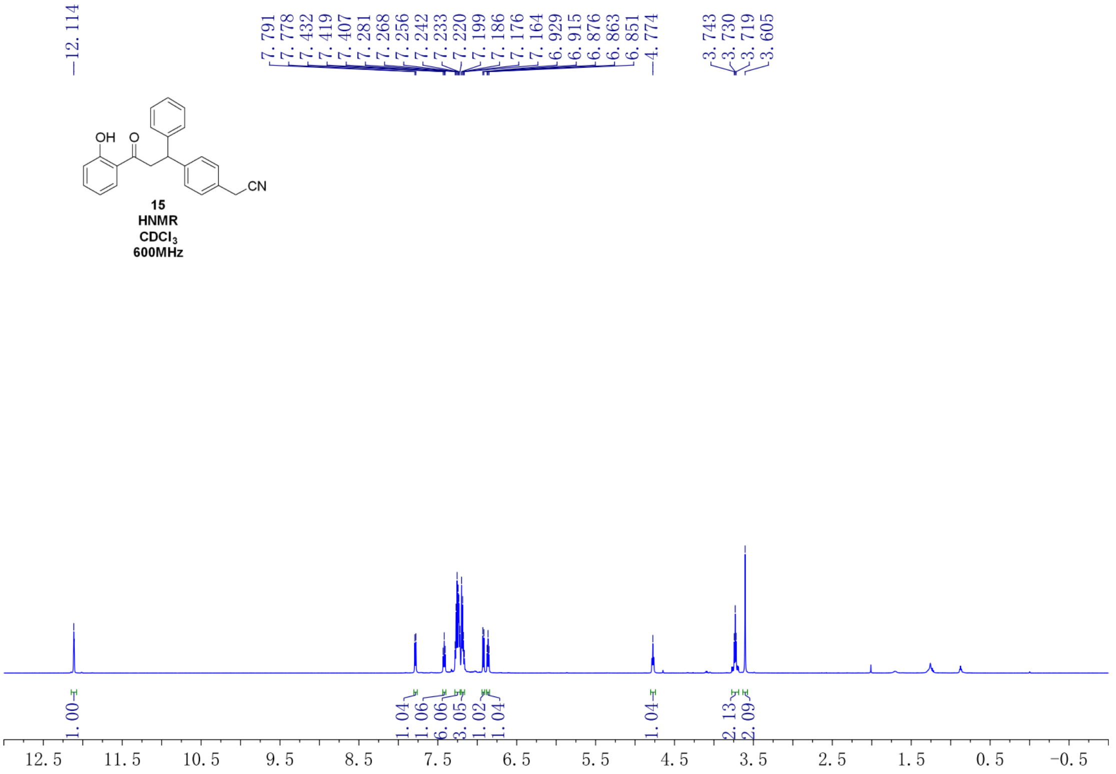
12.114
7.791
7.778
7.432
7.419
7.407
7.281
7.268
7.256
7.242
7.233
7.220
7.199
7.186
7.176
7.164
6.929
6.915
6.876
6.863
6.851
4.774
3.743
3.730
3.719
3.605
OH
O
CN
15
HNMR
CDCl3
600MHz
1.00
1.04
1.06
6.06
3.05
1.02
1.04
1.04
2.13
2.09
12.5
11.5
10.5
9.5
8.5
7.5
6.5
5.5
4.5
3.5
2.5
1.5
0.5
-0.5

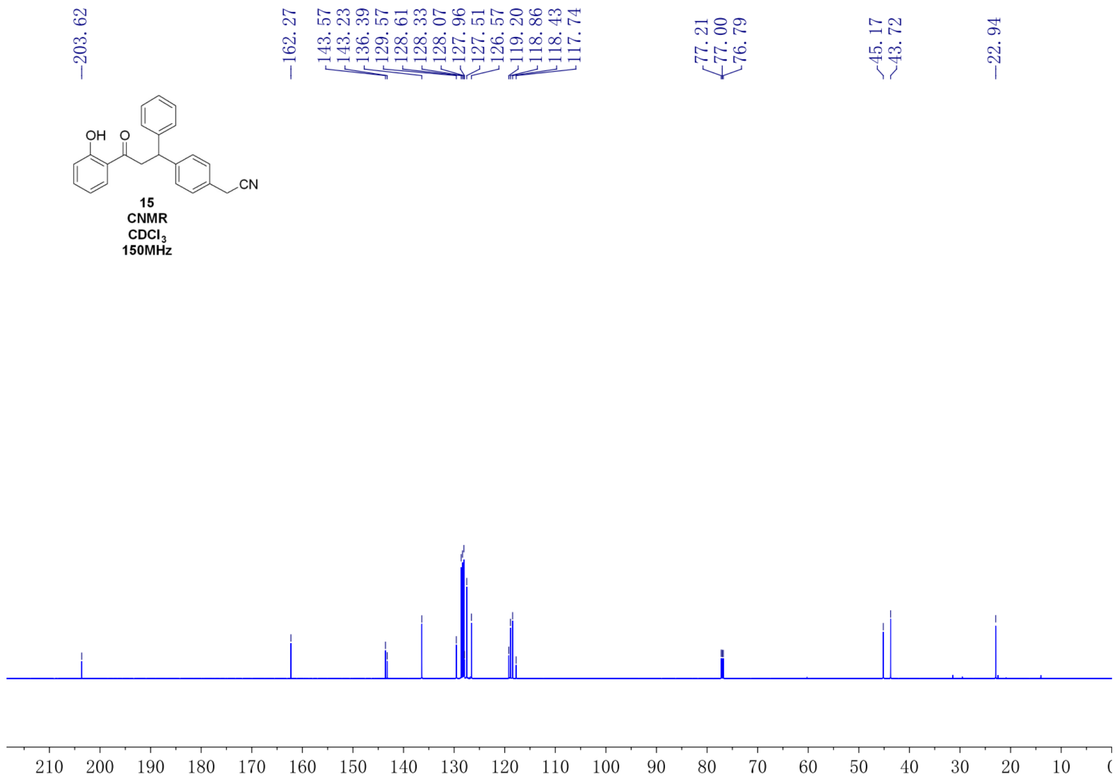
203.62
162.27
143.57
143.23
136.39
129.57
128.61
128.33
128.07
127.96
127.51
126.57
119.20
118.86
118.43
117.74
77.21
77.00
76.79
45.17
43.72
22.94
OH
O
CN
15
CNMR
CDCl3
150MHz
210 200 190 180 170 160 150 140 130 120 110 100 90 80 70 60 50 40 30 20 10

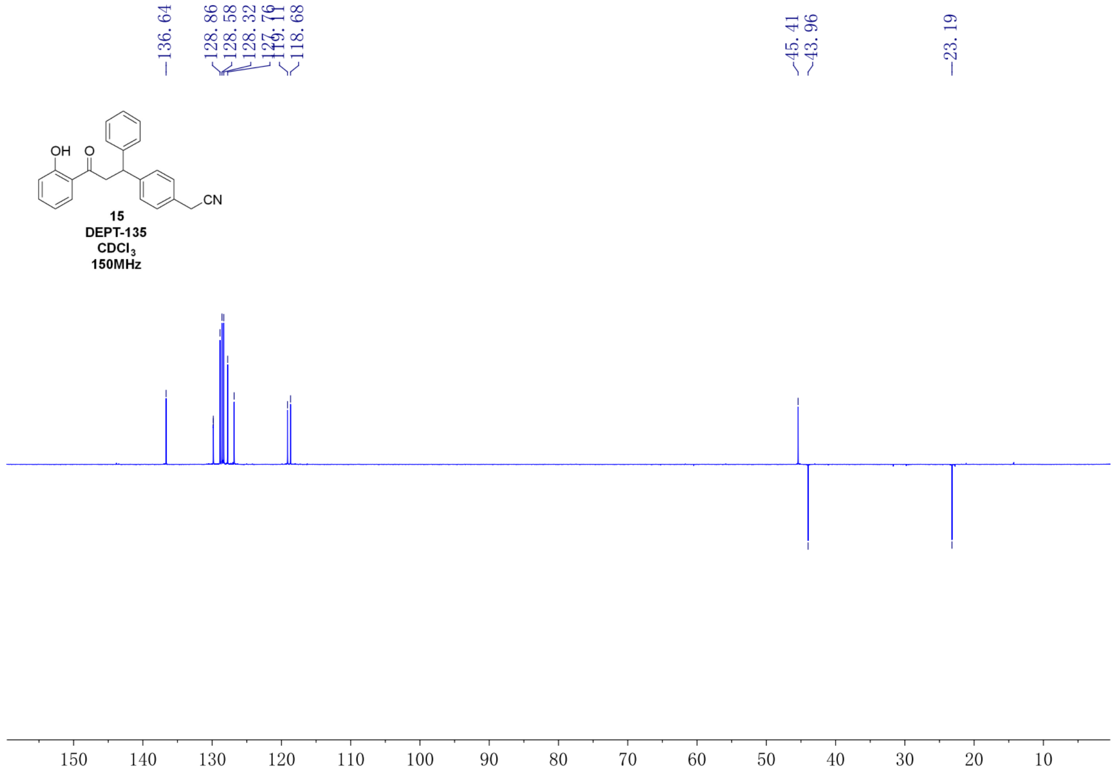
136.64
128.86
128.58
128.32
127.76
119.11
118.68
45.41
43.96
23.19
OH
O
CN
15
DEPT-135
CDCl3
150MHz
150 140 130 120 110 100 90 80 70 60 50 40 30 20 10

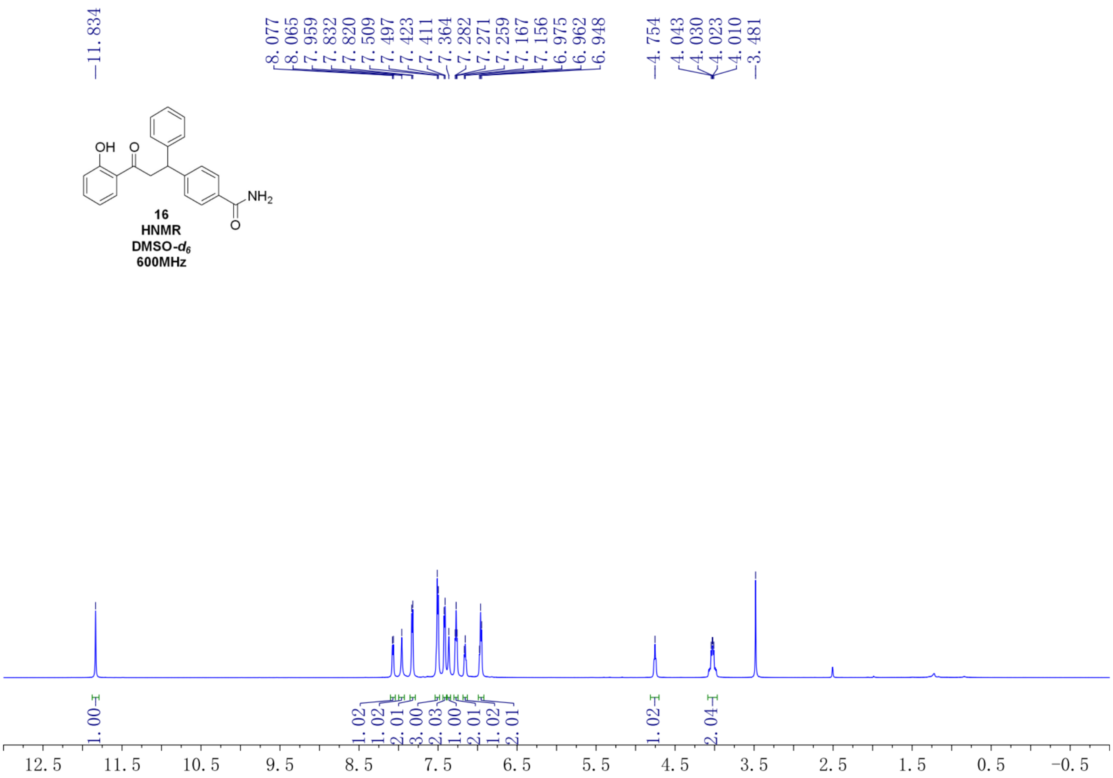

11.834
8.077
8.065
7.959
7.832
7.820
7.509
7.497
7.423
7.411
7.364
7.282
7.271
7.259
7.167
7.156
6.975
6.962
6.948
4.754
4.043
4.030
4.023
4.010
3.481
OH
O
NH2
O
16
HNMR
DMSO-d6
600MHz
1.00
1.02
1.02
2.01
3.00
2.03
1.00
2.01
1.02
2.01
1.02
2.04
12.5 11.5 10.5 9.5 8.5 7.5 6.5 5.5 4.5 3.5 2.5 1.5 0.5 -0.5

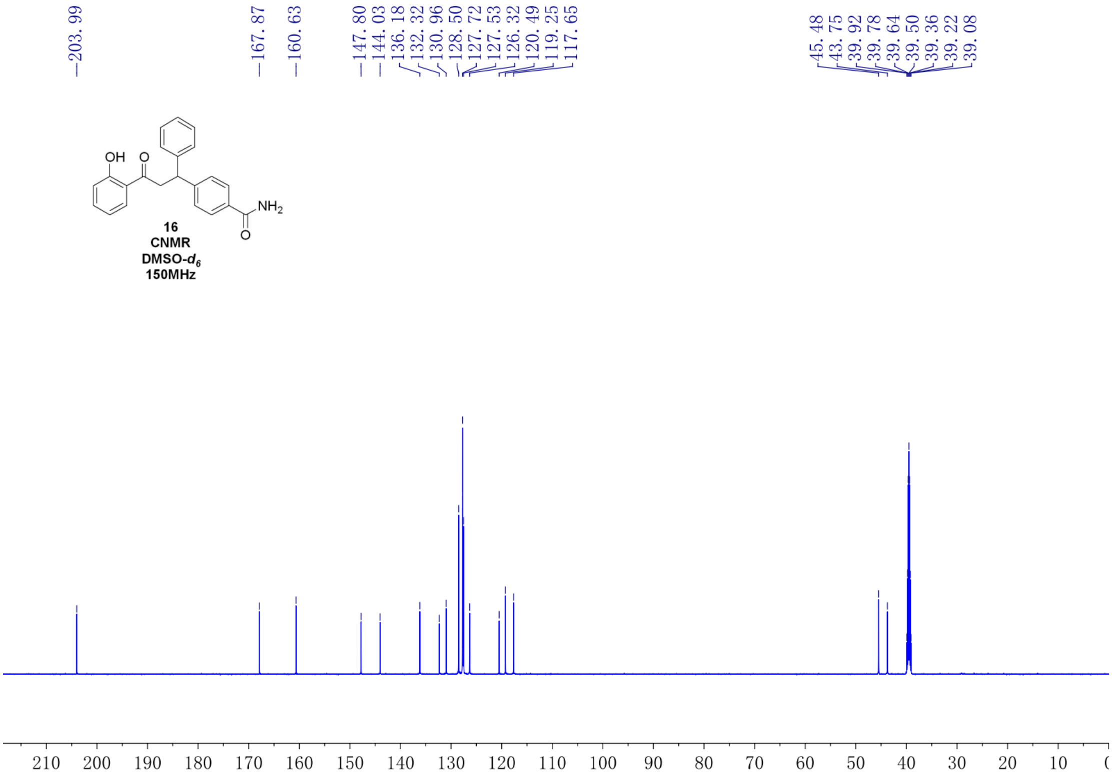

203.99
167.87
160.63
147.80
144.03
136.18
132.32
130.96
128.50
127.72
127.53
126.32
120.49
119.25
117.65
45.48
43.75
39.92
39.78
39.64
39.50
39.36
39.22
39.08
OH
O
NH2
O
16
CNMR
DMSO-d6
150MHz
210 200 190 180 170 160 150 140 130 120 110 100 90 80 70 60 50 40 30 20 10

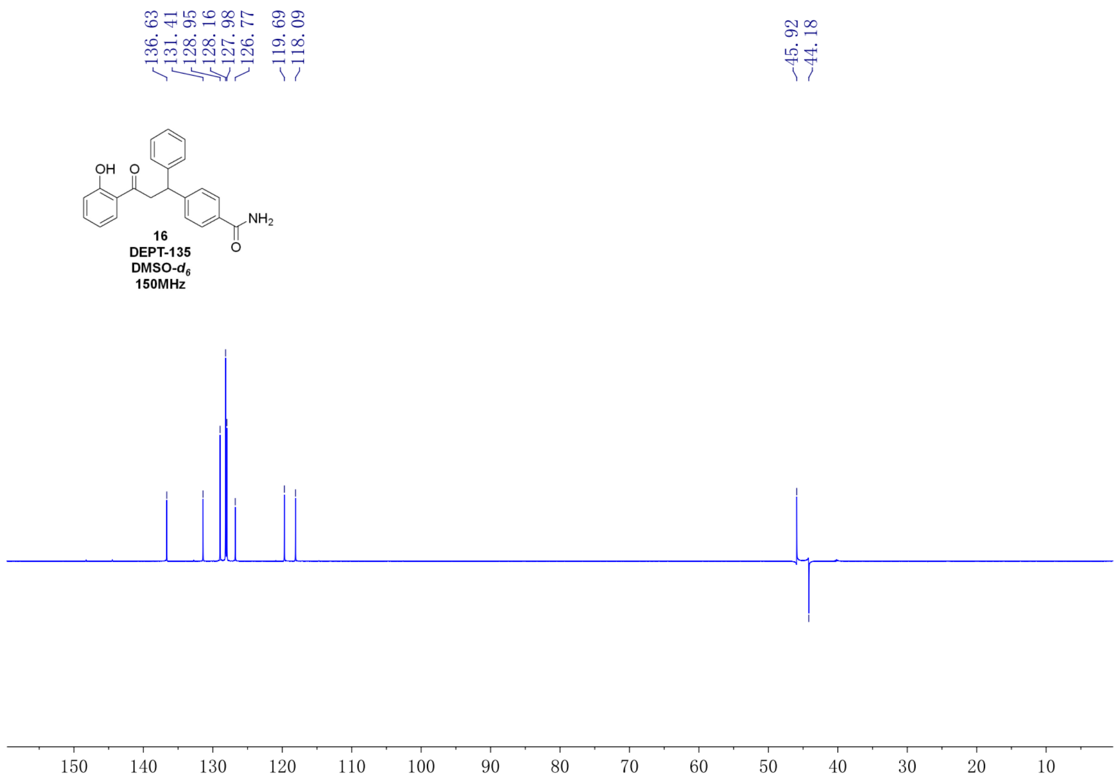
136.63
131.41
128.95
128.16
127.98
126.77
119.69
118.09
45.92
44.18
OH
O
NH2
16
DEPT-135
DMSO-d6
150MHz
150 140 130 120 110 100 90 80 70 60 50 40 30 20 10

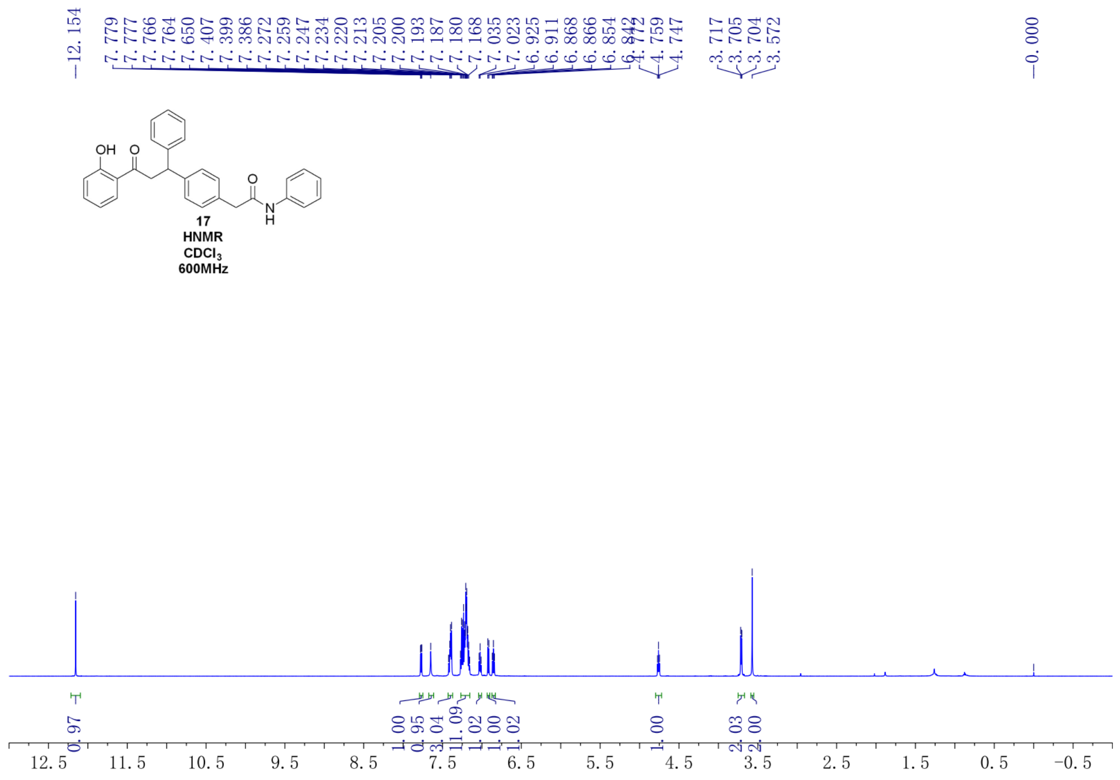
12.154
7.779
7.777
7.766
7.764
7.650
7.407
7.399
7.386
7.272
7.259
7.247
7.234
7.220
7.213
7.205
7.200
7.193
7.187
7.180
7.168
7.035
7.023
6.925
6.911
6.868
6.866
6.854
6.842
4.772
4.759
4.747
3.717
3.705
3.704
3.572
0.000
OH
O
O
N
H
17
HNMR
CDCl3
600MHz
0.97
1.00
0.95
3.04
11.09
1.02
1.00
1.02
1.00
2.03
2.00
12.5 11.5 10.5 9.5 8.5 7.5 6.5 5.5 4.5 3.5 2.5 1.5 0.5 -0.5

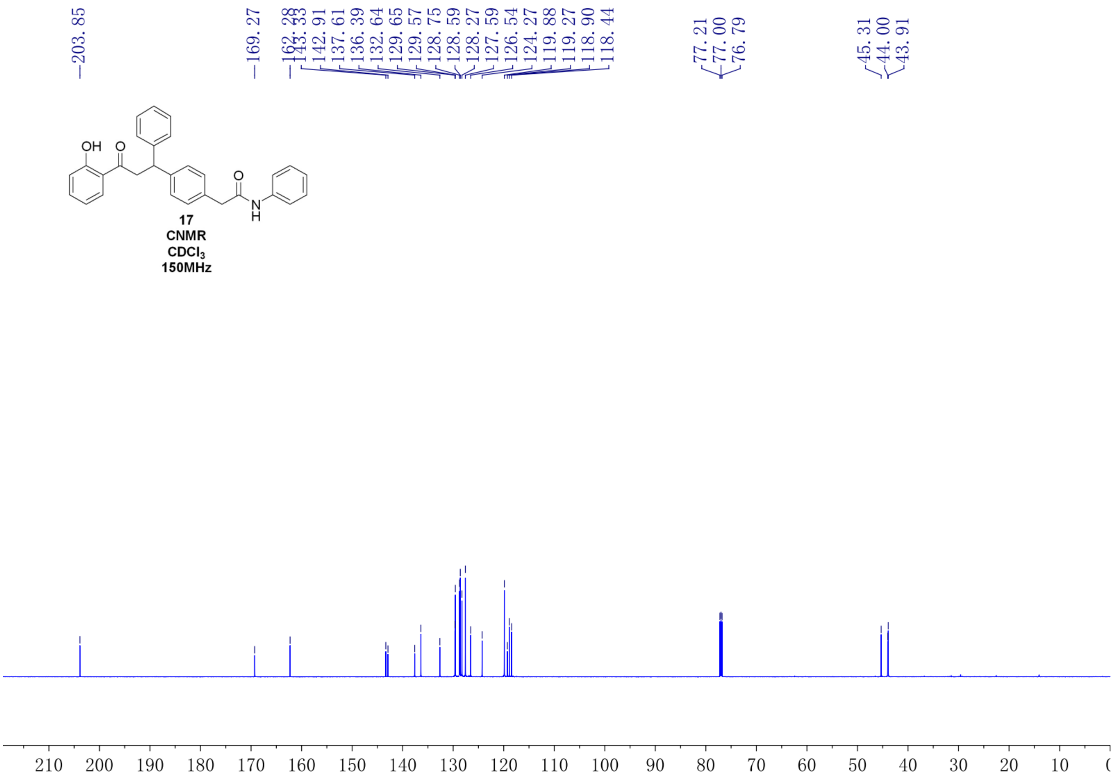
203.85
169.27
162.28
143.33
142.91
137.61
136.39
132.64
129.65
129.57
128.75
128.59
128.27
127.59
126.54
124.27
119.88
119.27
118.90
118.44
77.21
77.00
76.79
45.31
44.00
43.91
OH
O
O
N
H
17
CNMR
$CDCl_3$
150MHz
210 200 190 180 170 160 150 140 130 120 110 100 90 80 70 60 50 40 30 20 10

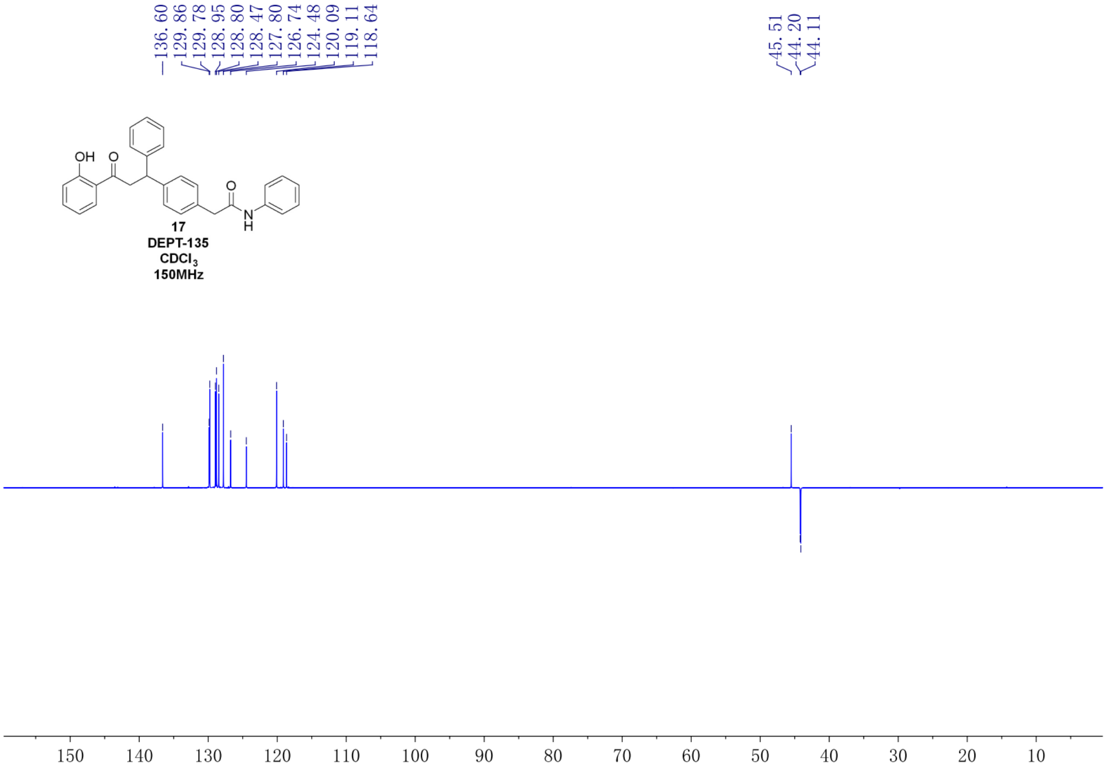
136.60
129.86
129.78
128.95
128.80
128.47
127.80
126.74
124.48
120.09
119.11
118.64
45.51
44.20
44.11
OH
O
O
N
H
17
DEPT-135
$CDCl_3$
150MHz
150 140 130 120 110 100 90 80 70 60 50 40 30 20 10

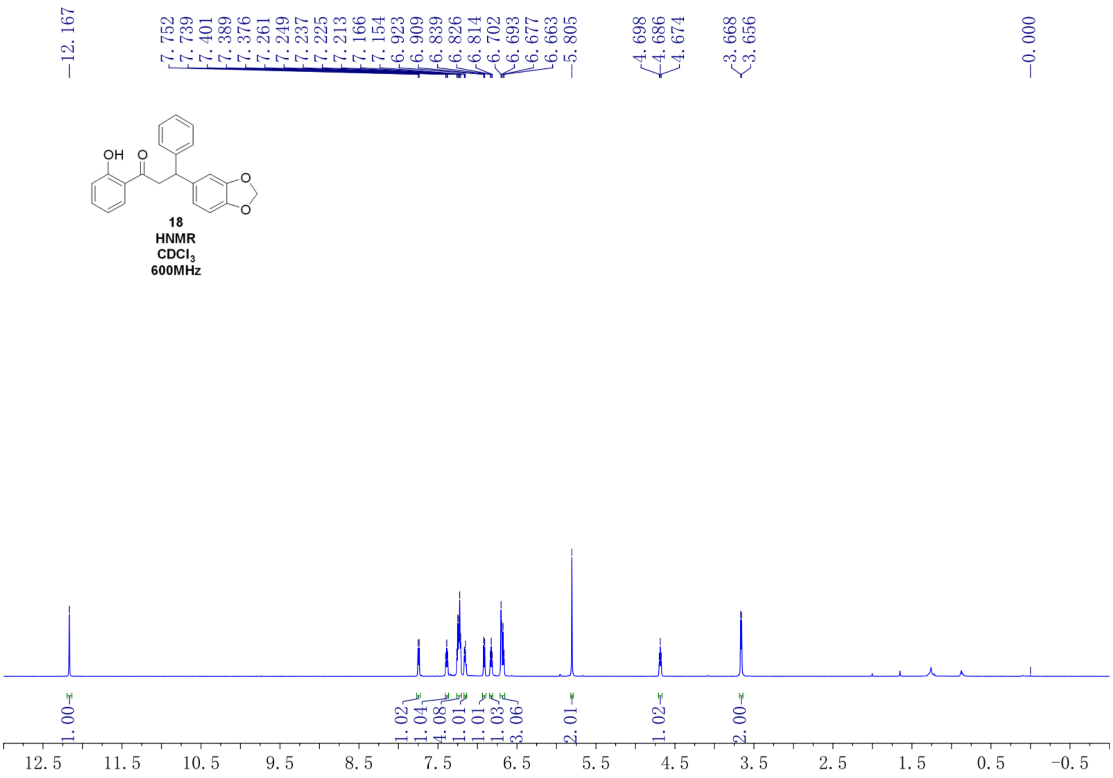
12.167
7.752
7.739
7.401
7.389
7.376
7.261
7.249
7.237
7.225
7.213
7.166
7.154
6.923
6.909
6.839
6.826
6.814
6.702
6.693
6.677
6.663
5.805
4.698
4.686
4.674
3.668
3.656
0.000
OH
O
O
O
18
HNMR
CDCl3
600MHz
1.00
1.02
1.04
4.08
1.01
1.01
1.03
3.06
2.01
1.02
2.00
12.5
11.5
10.5
9.5
8.5
7.5
6.5
5.5
4.5
3.5
2.5
1.5
0.5
-0.5

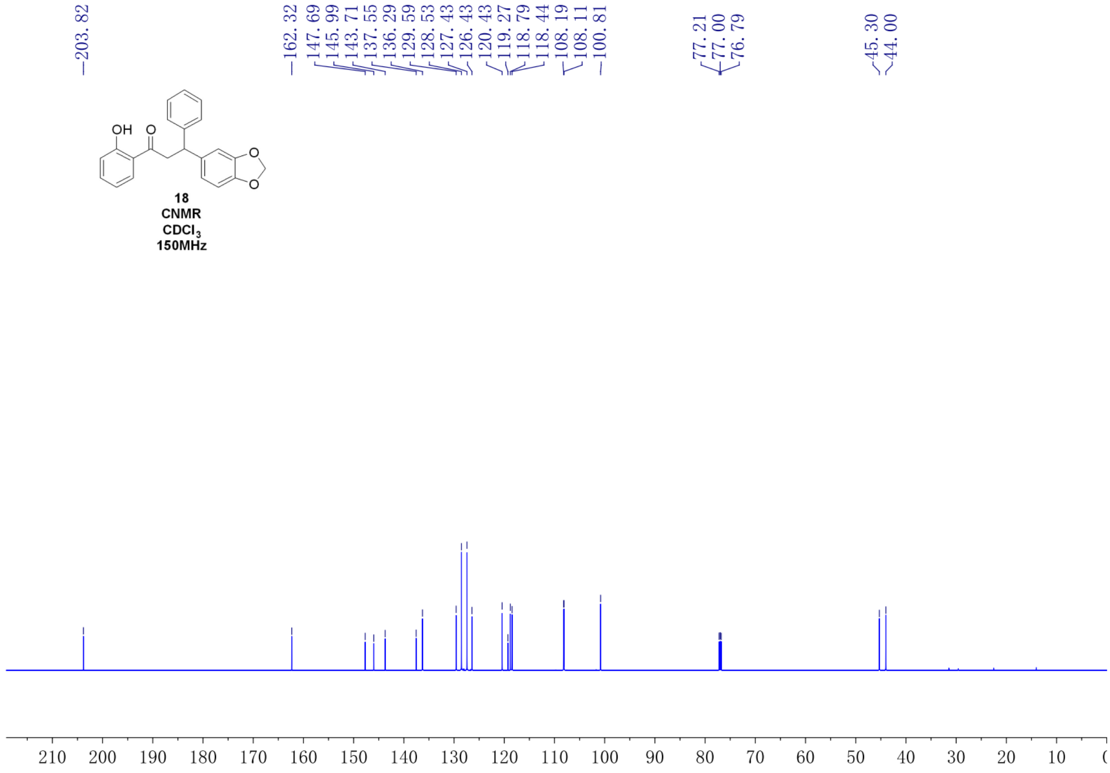
203.82
162.32
147.69
145.99
143.71
137.55
136.29
129.59
128.53
127.43
126.43
120.43
119.27
118.79
118.44
108.19
108.11
100.81
77.21
77.00
76.79
45.30
44.00
OH
O
O
O
18
CNMR
CDCl3
150MHz
210
200
190
180
170
160
150
140
130
120
110
100
90
80
70
60
50
40
30
20
10

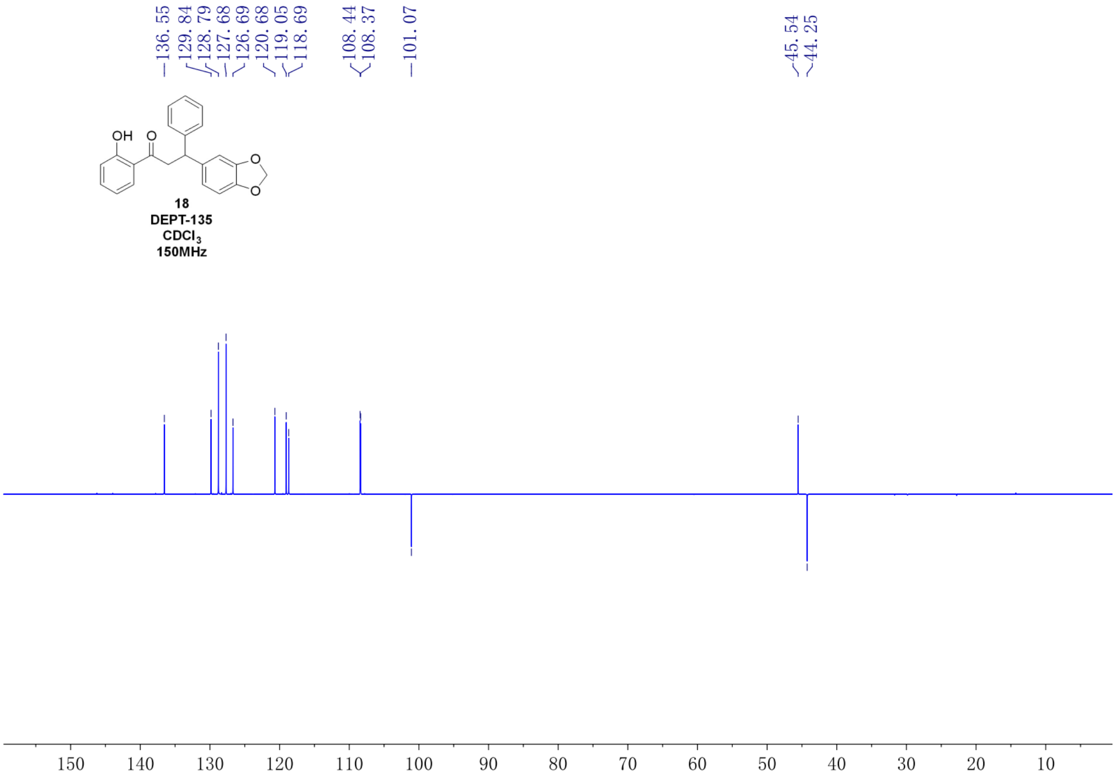
136.55
129.84
128.79
127.68
126.69
120.68
119.05
118.69
108.44
108.37
101.07
45.54
44.25
OH
O
O
O
18
DEPT-135
$CDCl_3$
150MHz
150 140 130 120 110 100 90 80 70 60 50 40 30 20 10

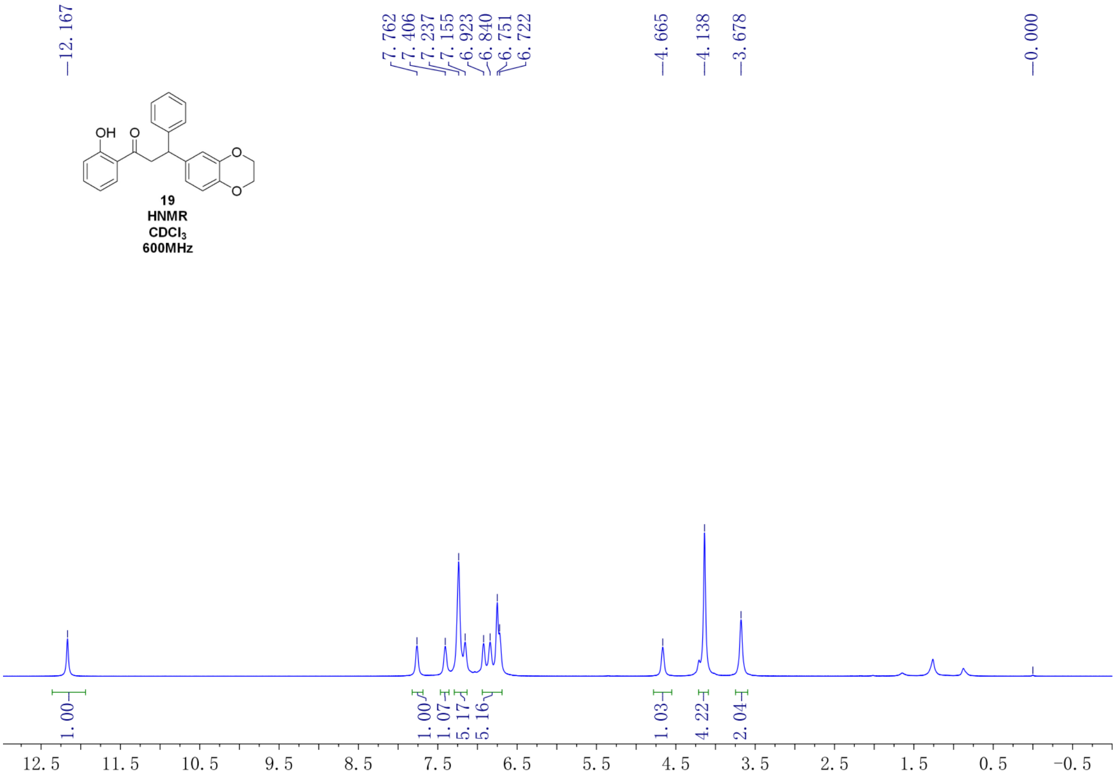
12.167
7.762
7.406
7.237
7.155
6.923
6.840
6.751
6.722
4.665
4.138
3.678
0.000
OH
O
O
O
19
HNMR
$CDCl_3$
600MHz
1.00
1.00
1.07
5.17
5.16
1.03
4.22
2.04
12.5 11.5 10.5 9.5 8.5 7.5 6.5 5.5 4.5 3.5 2.5 1.5 0.5 -0.5

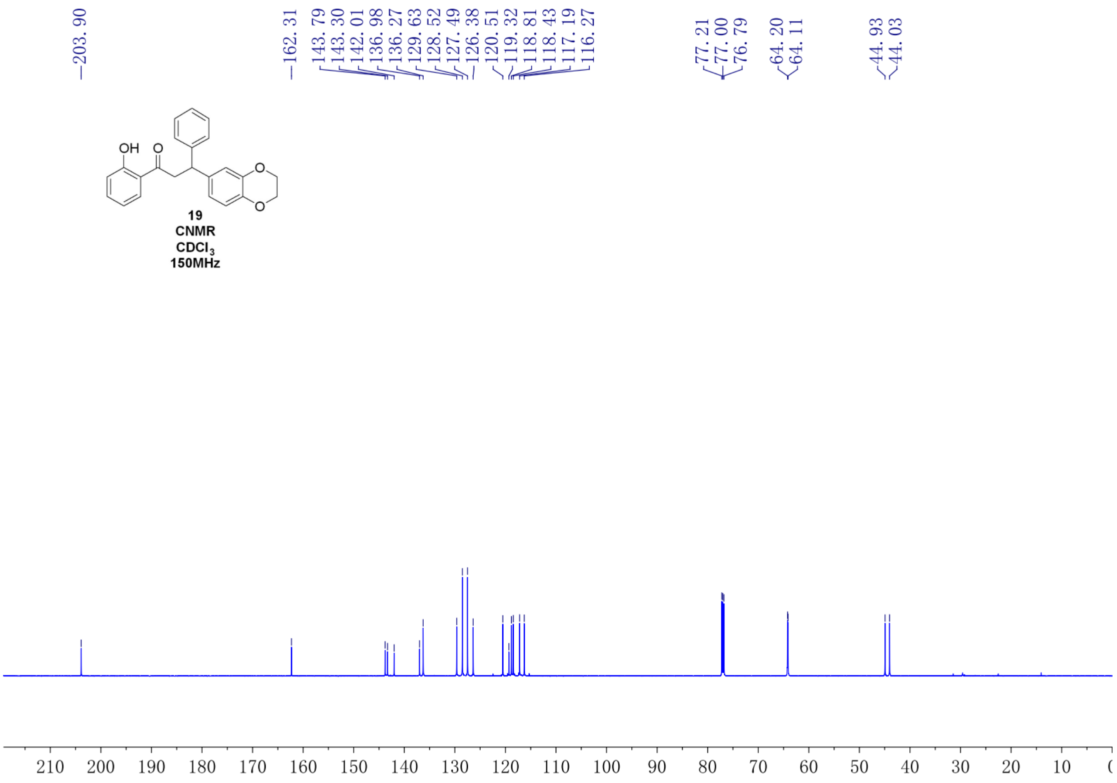

203.90
162.31
143.79
143.30
142.01
136.98
136.27
129.63
128.52
127.49
126.38
120.51
119.32
118.81
118.43
117.19
116.27
77.21
77.00
76.79
64.20
64.11
44.93
44.03
OH
O
O
O
19
CNMR
CDCl3
150MHz
210 200 190 180 170 160 150 140 130 120 110 100 90 80 70 60 50 40 30 20 10

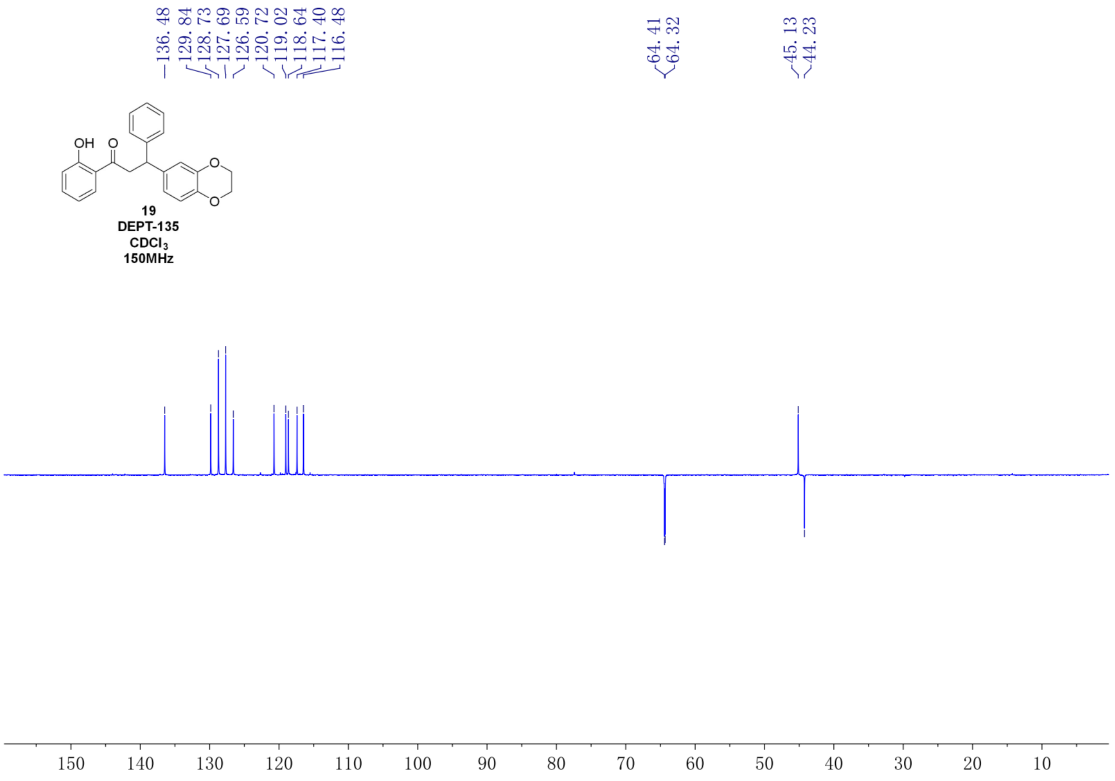

136.48
129.84
128.73
127.69
126.59
120.72
119.02
118.64
117.40
116.48
64.41
64.32
45.13
44.23
OH
O
O
O
19
DEPT-135
CDCl3
150MHz
150 140 130 120 110 100 90 80 70 60 50 40 30 20 10

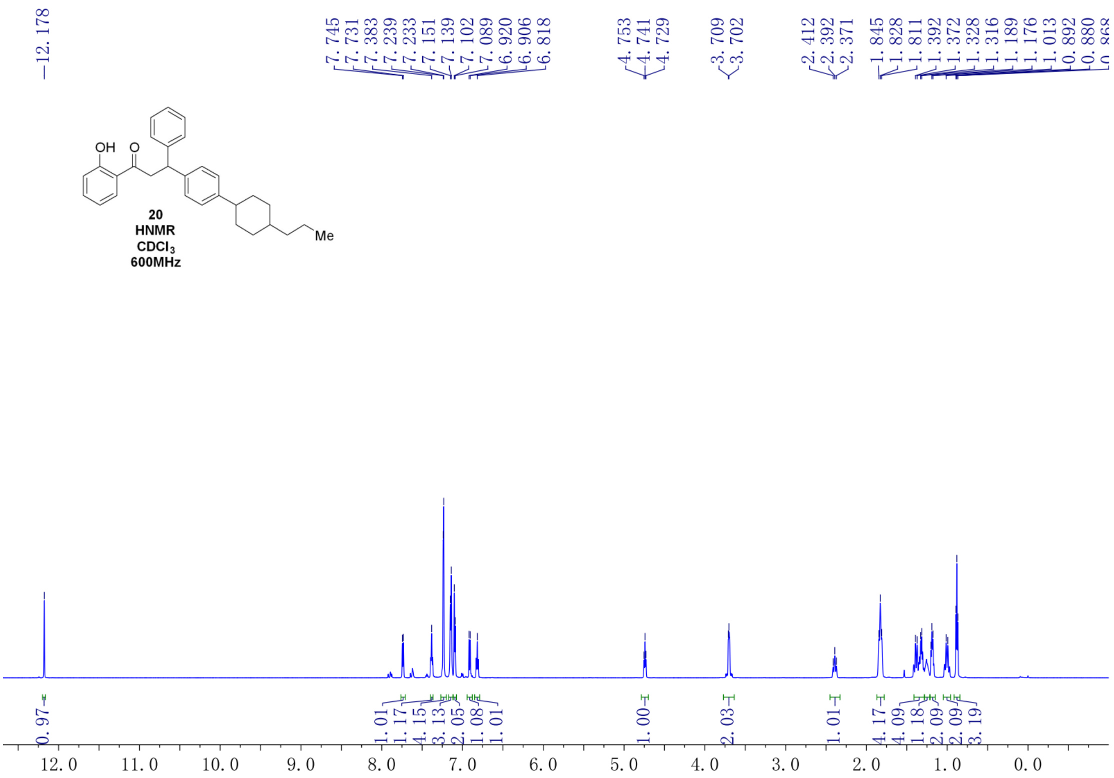
12.178
7.745 7.731 7.383 7.239 7.233 7.151 7.139 7.102 7.089 6.920 6.906 6.818
4.753 4.741 4.729
3.709 3.702
2.412 2.392 2.371
1.845 1.828 1.811 1.392 1.372 1.328 1.316 1.189 1.176 1.013 0.892 0.880
OH
O
Me
20
HNMR
CDCl3
600MHz
0.97 1.01 1.17 4.15 3.13 2.05 1.08 1.01 1.00 2.03 1.01 4.17 4.09 1.18 2.09 2.09 3.19
12.0 11.0 10.0 9.0 8.0 7.0 6.0 5.0 4.0 3.0 2.0 1.0 0.0

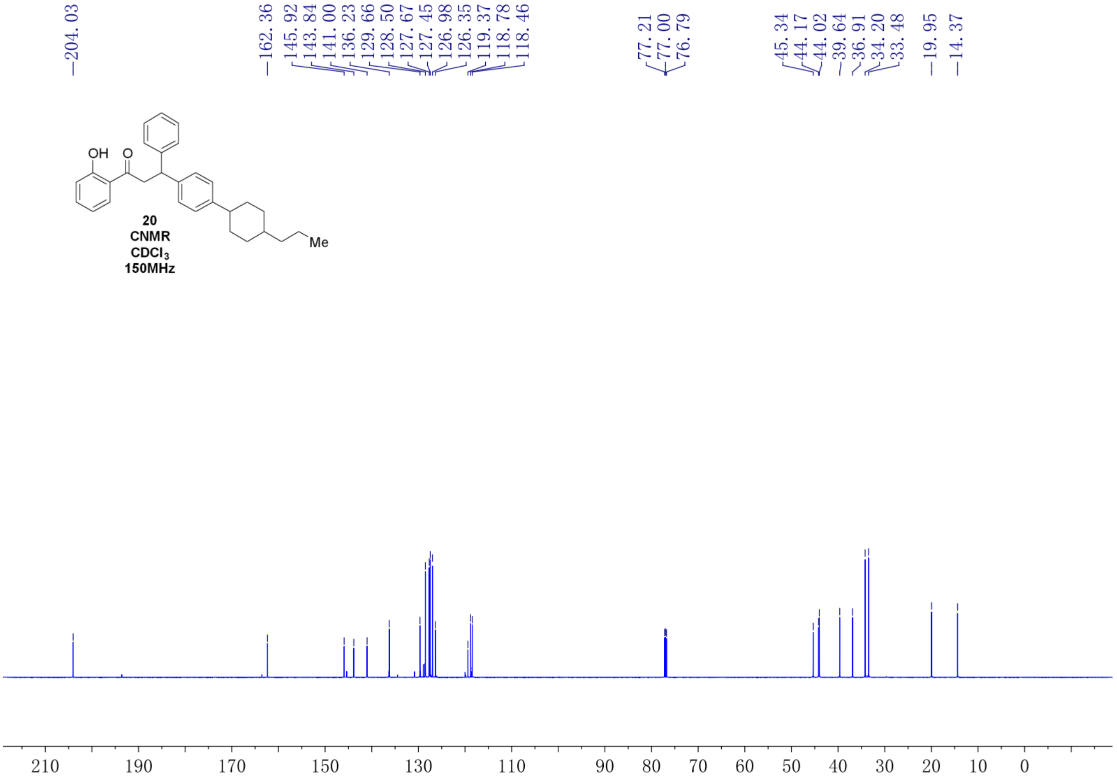
204.03
162.36 145.92 143.84 141.00 136.23 129.66 128.50 127.67 127.45 126.98 126.35 119.37 118.78 118.46
77.21 77.00 76.79
45.34 44.17 44.02 39.64 36.91 34.20 33.48 19.95 14.37
OH
O
Me
20
CNMR
CDCl3
150MHz
210 190 170 150 130 110 90 80 70 60 50 40 30 20 10 0

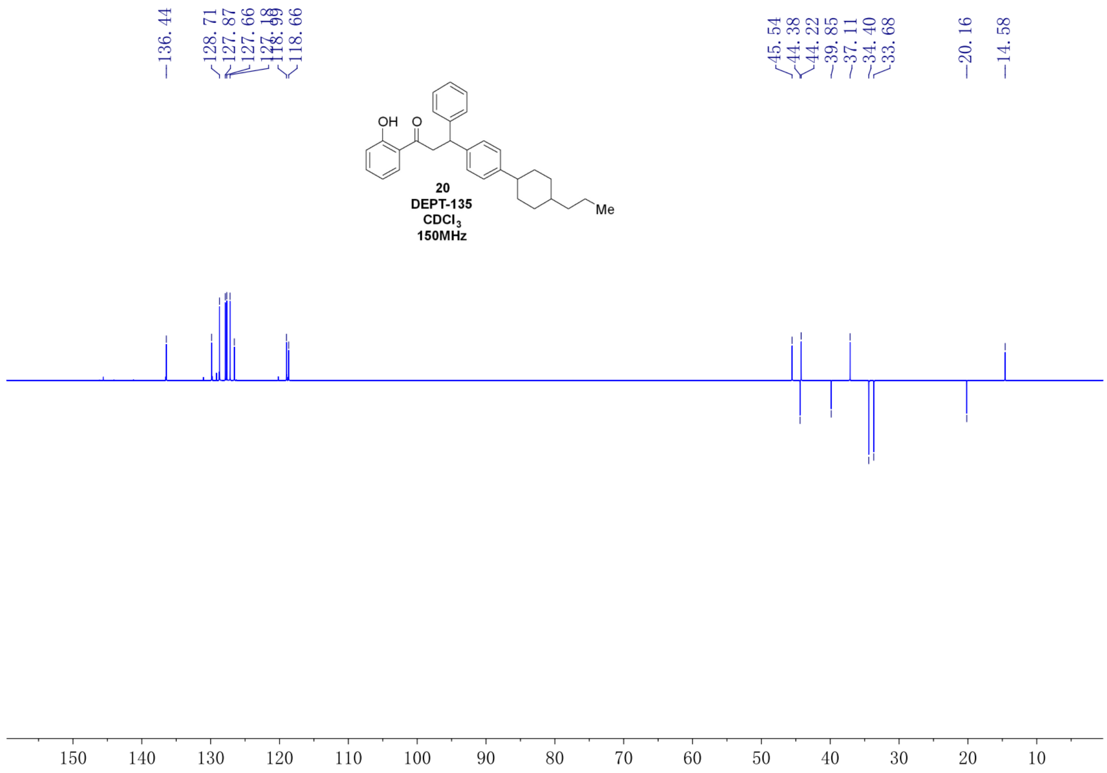
136.44
128.71
127.87
127.66
127.18
118.66
45.54
44.38
44.22
39.85
37.11
34.40
33.68
20.16
14.58
OH
O
Me
20
DEPT-135
CDCl3
150MHz
150
140
130
120
110
100
90
80
70
60
50
40
30
20
10

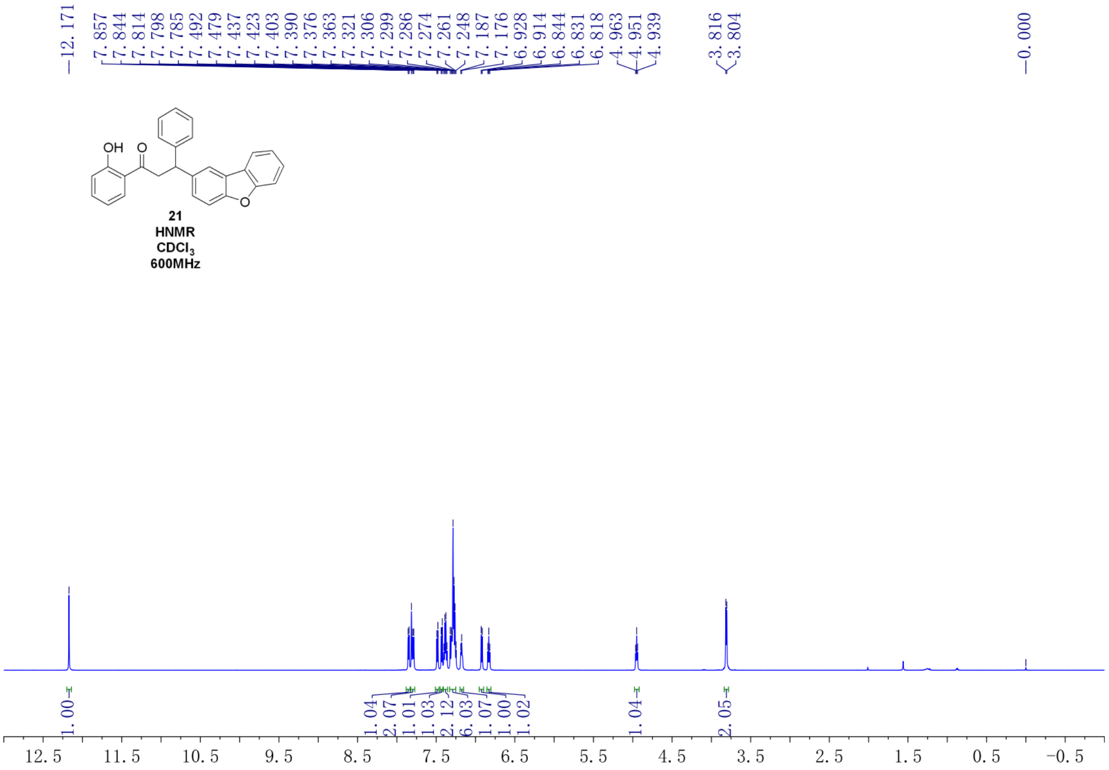
12.171
7.857
7.844
7.814
7.798
7.785
7.492
7.479
7.437
7.423
7.403
7.390
7.376
7.363
7.321
7.306
7.299
7.286
7.274
7.261
7.248
7.187
7.176
6.928
6.914
6.844
6.831
6.818
4.963
4.951
4.939
3.816
3.804
0.000
OH
O
O
21
HNMR
CDCl3
600MHz
1.00
1.04
2.07
1.01
1.03
2.12
6.03
1.07
1.00
1.02
1.04
2.05
12.5
11.5
10.5
9.5
8.5
7.5
6.5
5.5
4.5
3.5
2.5
1.5
0.5
-0.5

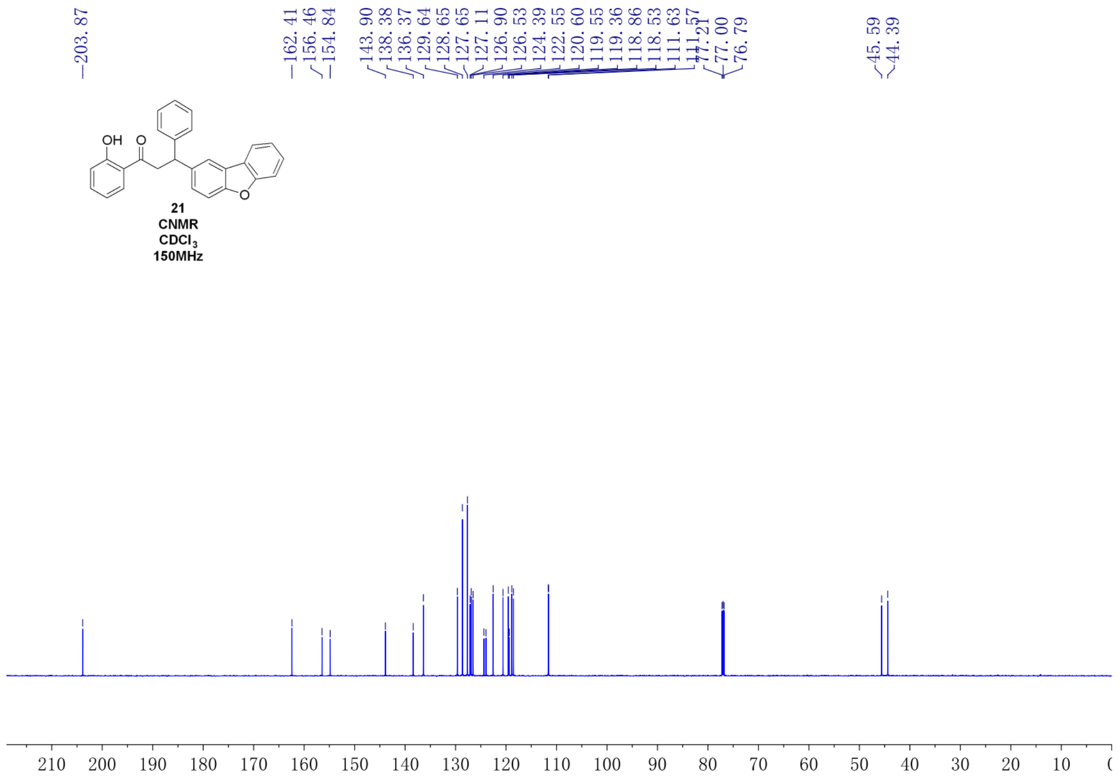
203.87
162.41
156.46
154.84
143.90
138.38
136.37
129.64
128.65
127.65
127.11
126.90
126.53
124.39
122.55
120.60
119.55
119.36
118.86
118.53
111.63
111.57
77.21
77.00
76.79
45.59
44.39
OH
O
O
21
CNMR
CDCl3
150MHz

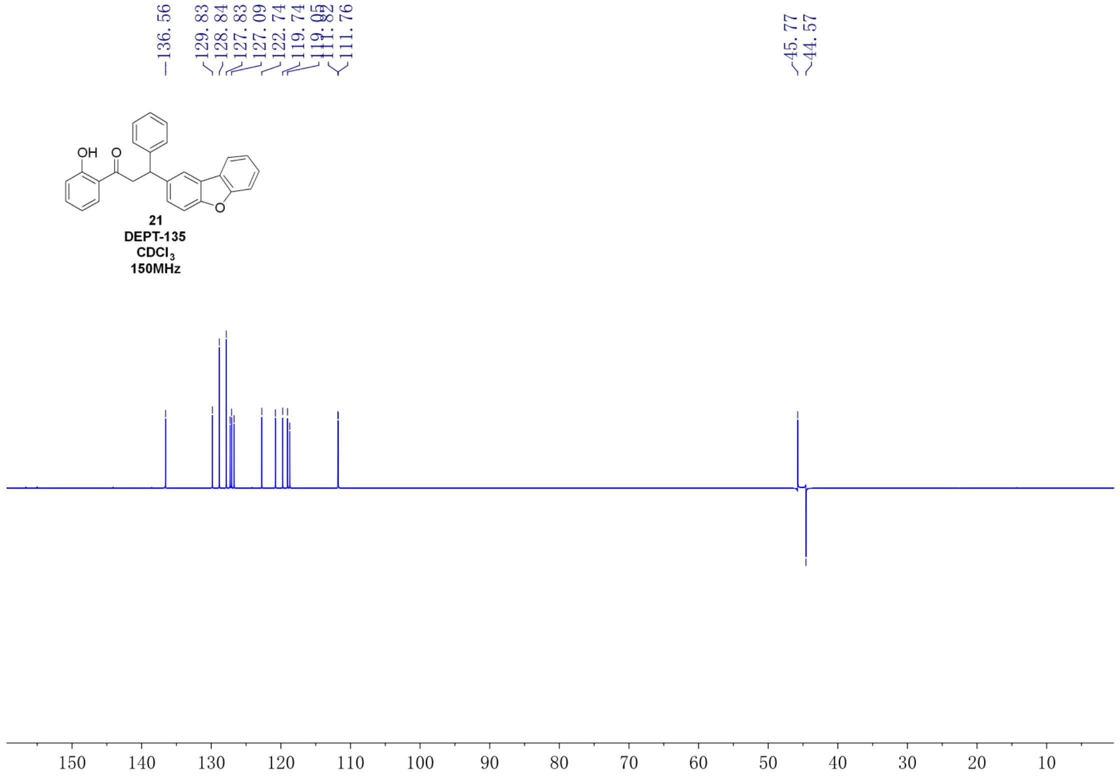
136.56
129.83
128.84
127.83
127.09
122.74
119.74
111.76
45.77
44.57
OH
O
O
21
DEPT-135
CDCl3
150MHz

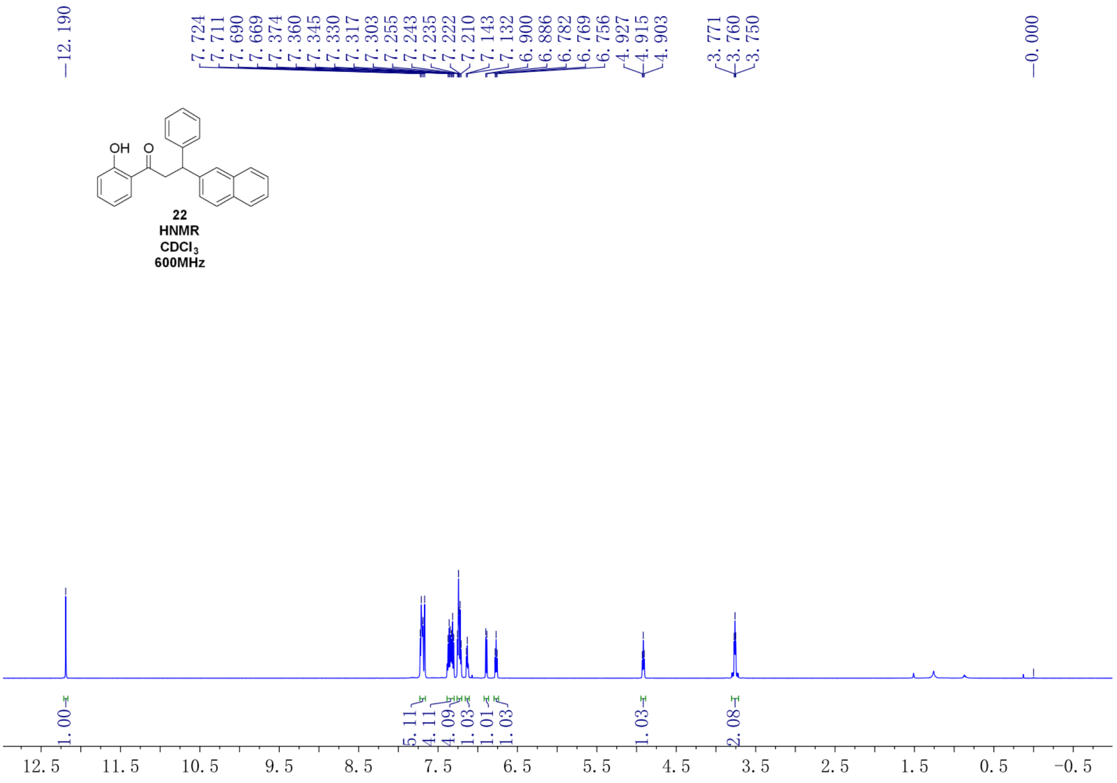
12.190
7.724
7.711
7.690
7.669
7.374
7.360
7.345
7.330
7.317
7.303
7.255
7.243
7.235
7.222
7.210
7.143
7.132
6.900
6.886
6.782
6.769
6.756
4.927
4.915
4.903
3.771
3.760
3.750
0.000
OH
O
22
HNMR
$CDCl_3$
600MHz
1.00
5.11
4.11
4.09
1.03
1.01
1.03
1.03
2.08
12.5
11.5
10.5
9.5
8.5
7.5
6.5
5.5
4.5
3.5
2.5
1.5
0.5
-0.5

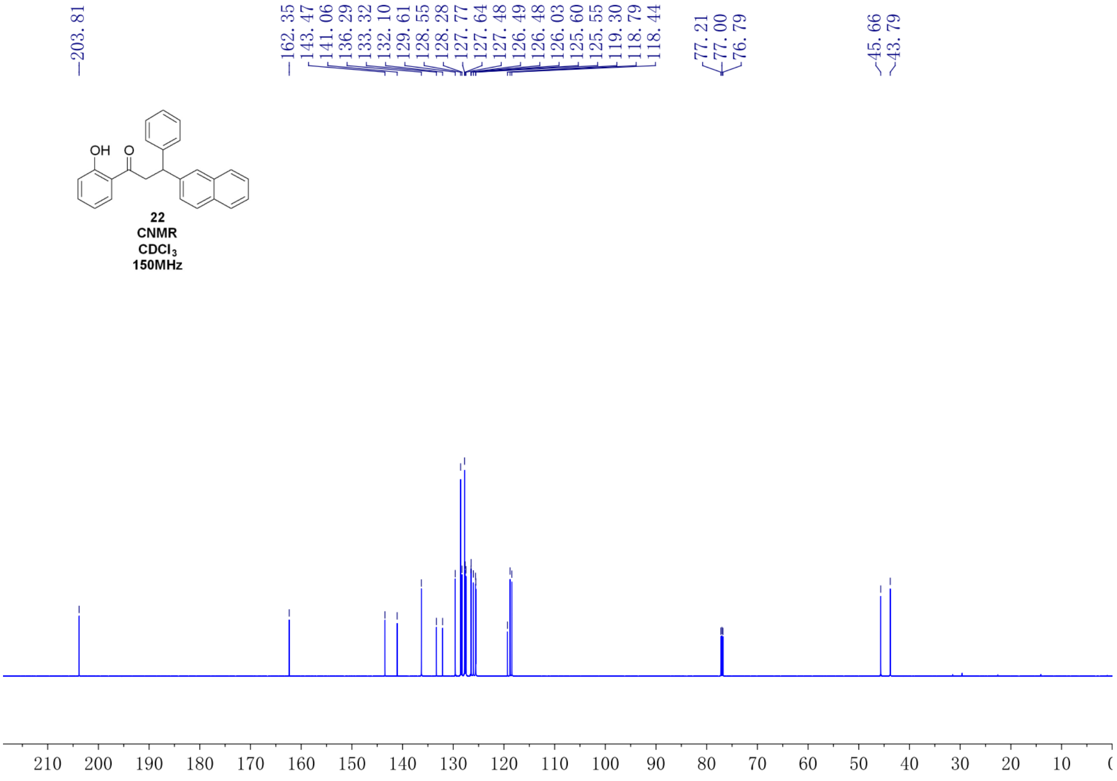
203.81
162.35
143.47
141.06
136.29
133.32
132.10
129.61
128.55
128.28
127.77
127.64
127.48
126.49
126.48
126.03
125.60
125.55
119.30
118.79
118.44
77.21
77.00
76.79
45.66
43.79
OH
O
22
CNMR
$CDCl_3$
150MHz
210
200
190
180
170
160
150
140
130
120
110
100
90
80
70
60
50
40
30
20
10

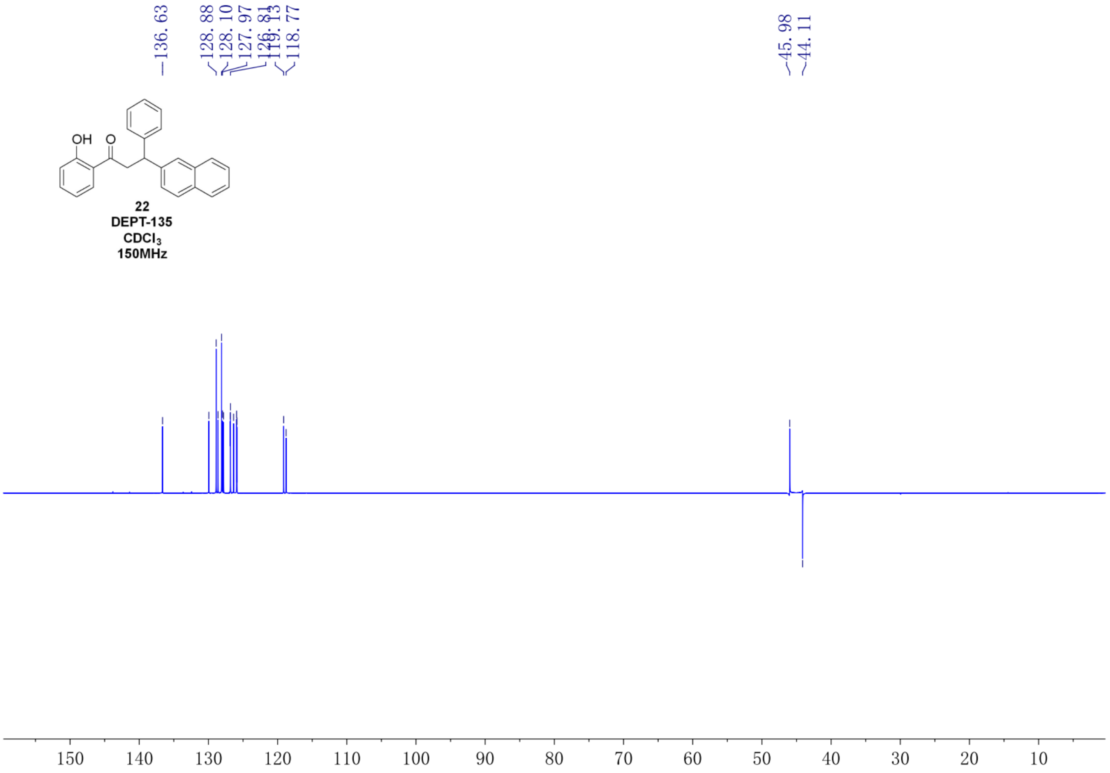
136.63
128.88
128.10
127.97
126.81
119.13
118.77
45.98
44.11
OH
O
22
DEPT-135
$CDCl_3$
150MHz
150
140
130
120
110
100
90
80
70
60
50
40
30
20
10

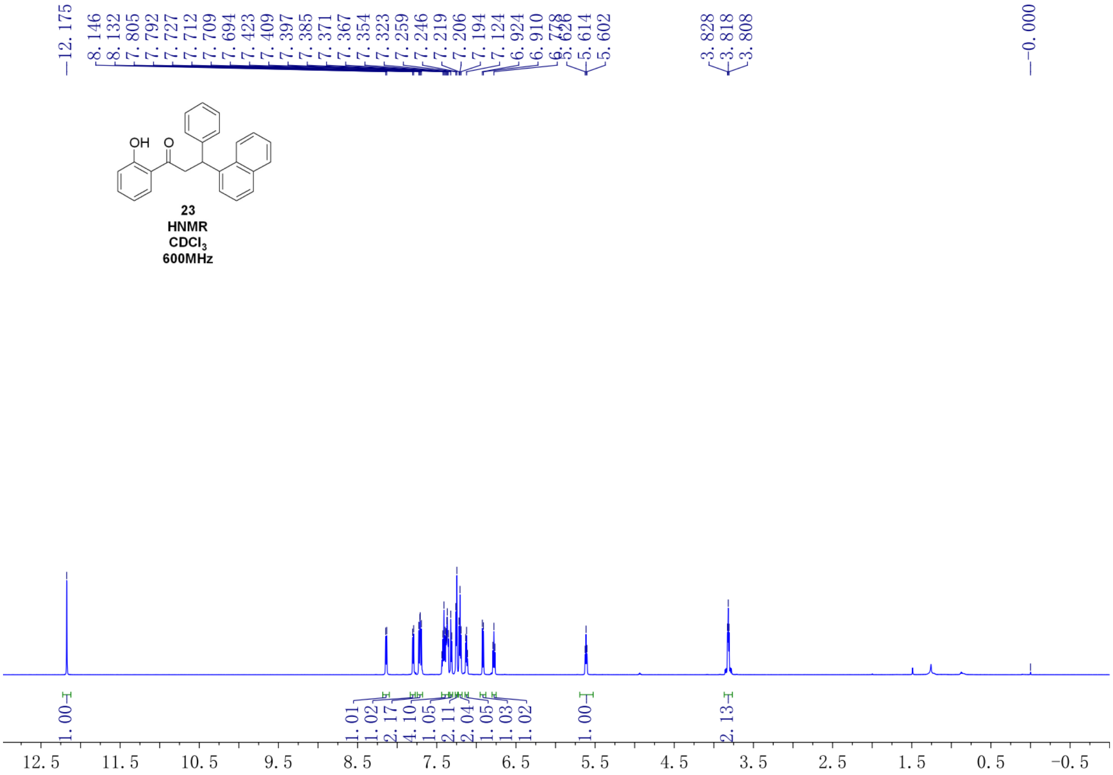
12.175
8.146
8.132
7.805
7.792
7.727
7.712
7.709
7.694
7.423
7.409
7.397
7.385
7.371
7.367
7.354
7.323
7.259
7.246
7.219
7.206
7.194
7.124
6.924
6.910
6.778
5.626
5.614
5.602
3.828
3.818
3.808
-0.000
OH
O
23
HNMR
$CDCl_3$
600MHz
1.00
1.01
1.02
2.17
4.10
1.05
2.11
2.04
1.05
1.03
1.02
1.00
2.13
12.5
11.5
10.5
9.5
8.5
7.5
6.5
5.5
4.5
3.5
2.5
1.5
0.5
-0.5

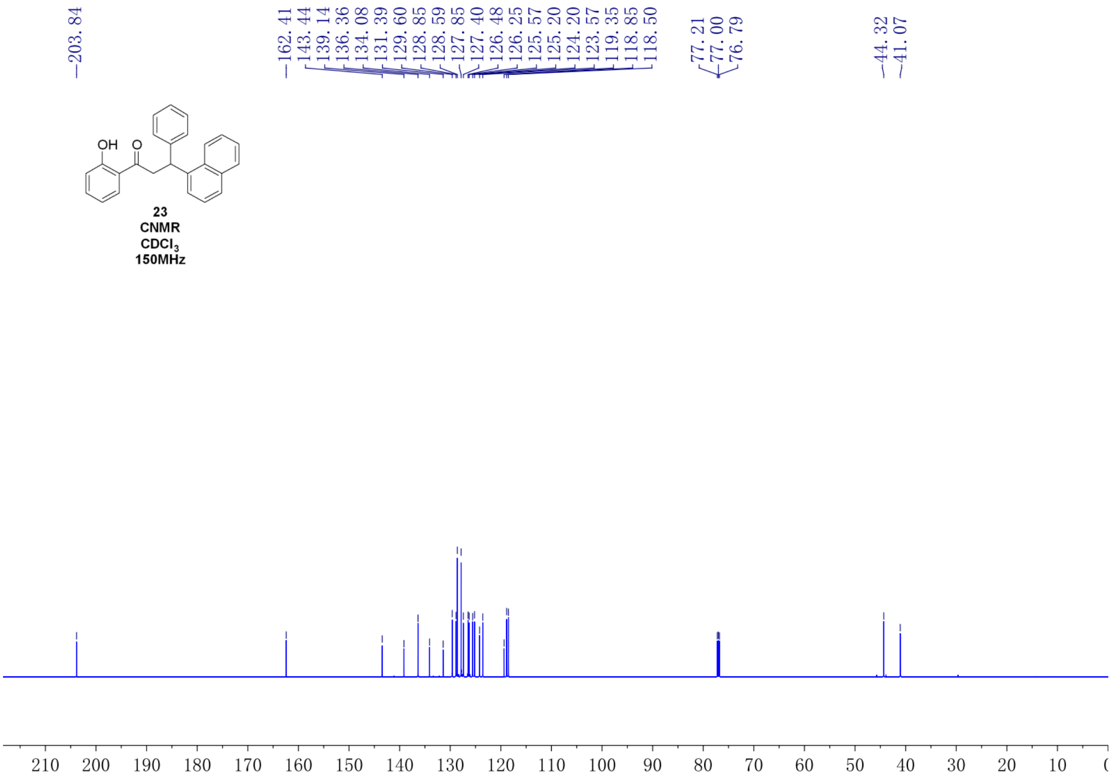
203.84
162.41
143.44
139.14
136.36
134.08
131.39
129.60
128.85
128.59
127.85
127.40
126.48
126.25
125.57
125.20
124.20
123.57
119.35
118.85
118.50
77.21
77.00
76.79
44.32
41.07
OH
O
23
CNMR
$CDCl_3$
150MHz
210 200 190 180 170 160 150 140 130 120 110 100 90 80 70 60 50 40 30 20 10

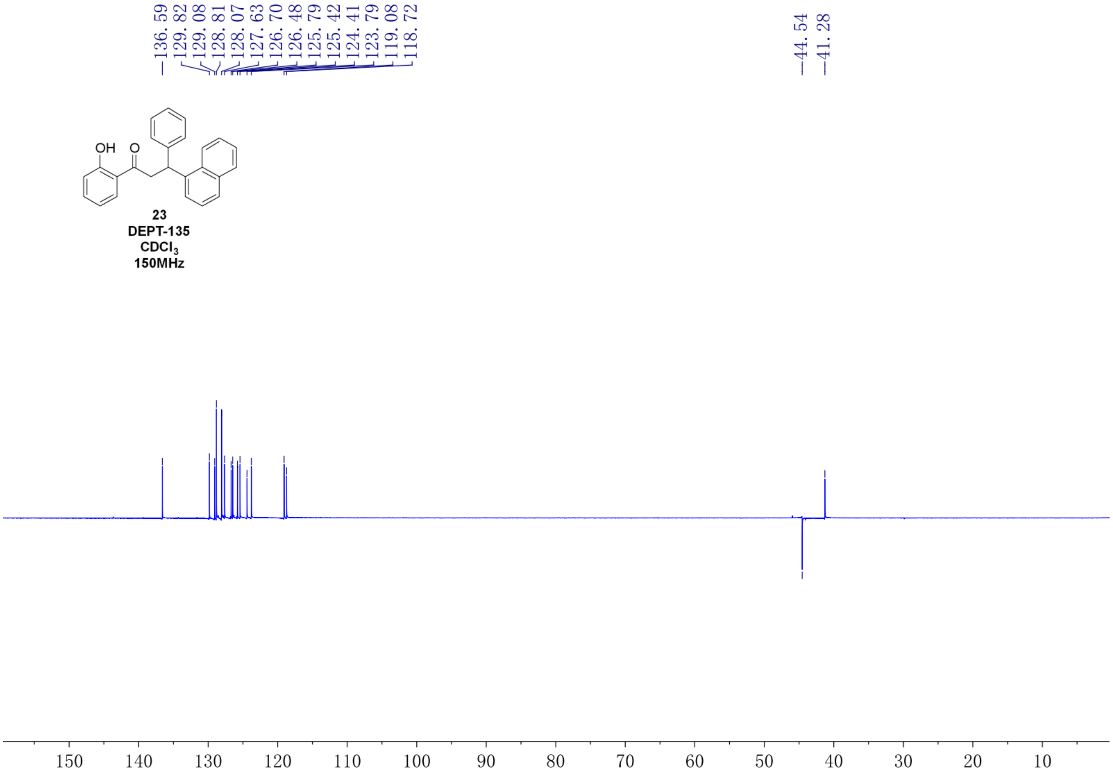
136.59
129.82
129.08
128.81
128.07
127.63
126.70
126.48
125.79
125.42
124.41
123.79
119.08
118.72
44.54
41.28
OH
O
23
DEPT-135
$CDCl_3$
150MHz
150 140 130 120 110 100 90 80 70 60 50 40 30 20 10

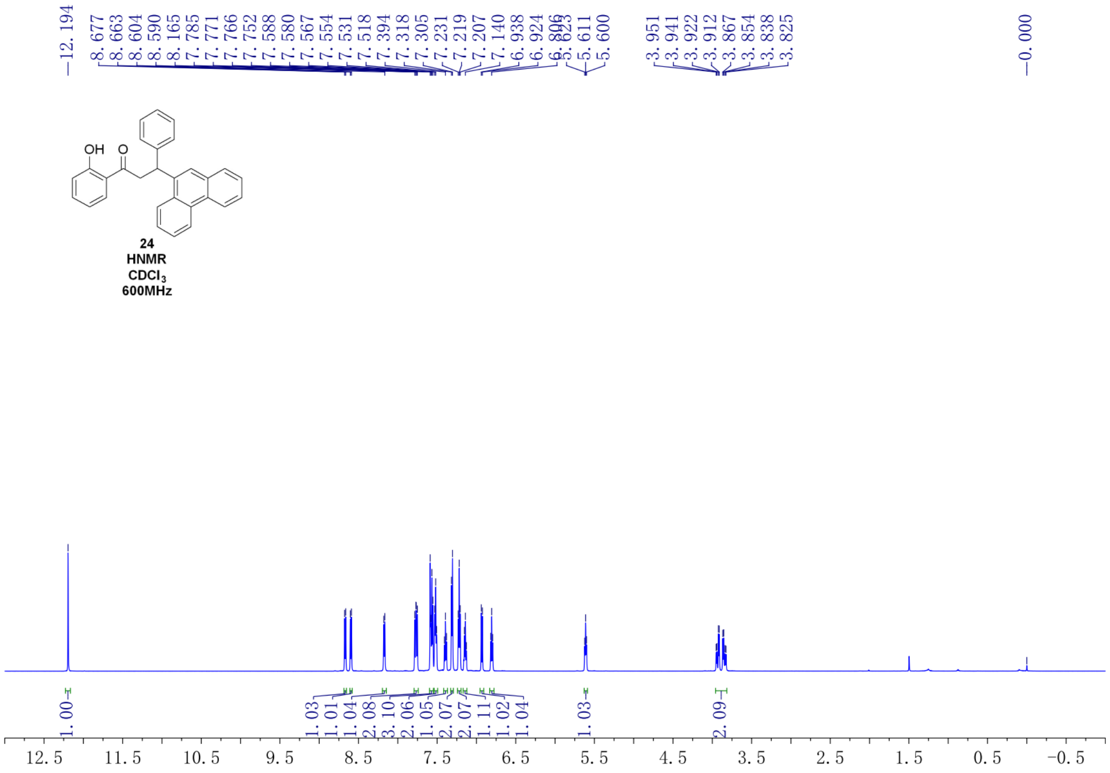
12.194
8.677
8.663
8.604
8.590
8.165
7.785
7.771
7.766
7.752
7.588
7.580
7.567
7.554
7.531
7.518
7.394
7.318
7.305
7.231
7.219
7.207
7.140
6.938
6.924
[illegible]
5.623
5.611
5.600
3.951
3.941
3.922
3.912
3.867
3.854
3.838
3.825
0.000
OH
O
24
HNMR
CDCl3
600MHz
1.00
1.03
1.01
1.04
2.08
3.10
2.06
1.05
2.07
2.07
1.11
1.02
1.04
1.03
2.09
12.5 11.5 10.5 9.5 8.5 7.5 6.5 5.5 4.5 3.5 2.5 1.5 0.5 -0.5

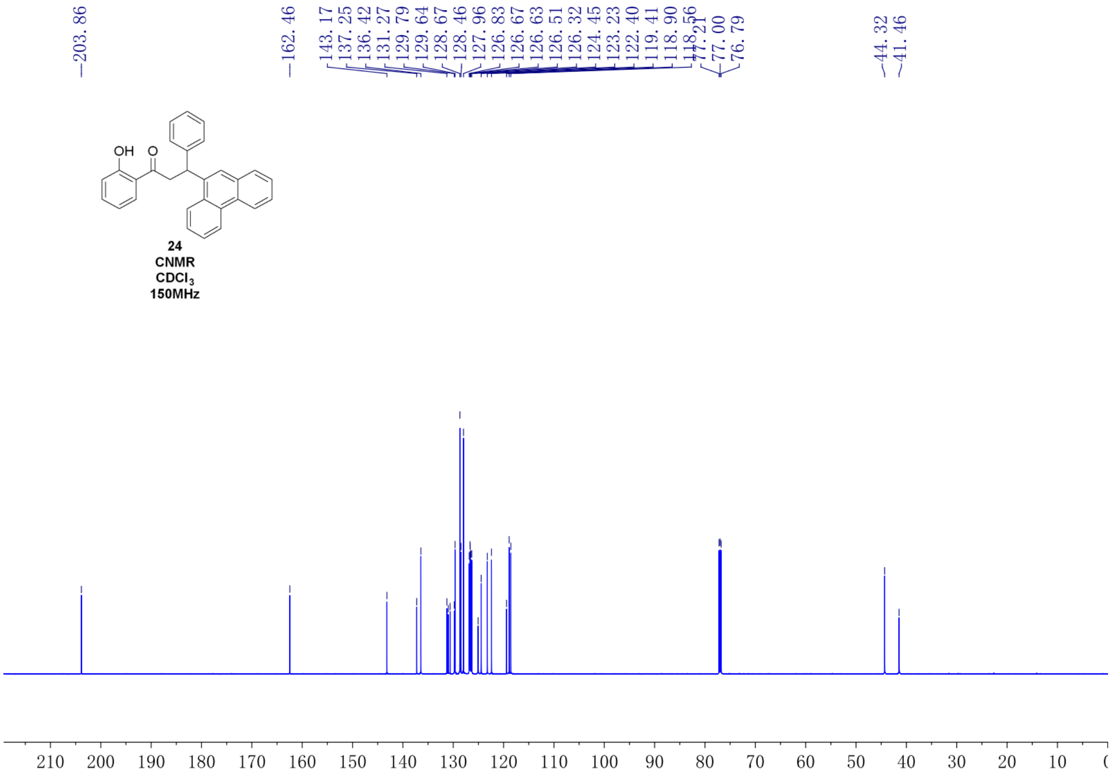
203.86
162.46
143.17
137.25
136.42
131.27
129.79
129.64
128.67
128.46
127.96
126.83
126.67
126.63
126.51
126.32
124.45
123.23
122.40
119.41
118.90
118.56
77.21
77.00
76.79
44.32
41.46
OH
O
24
CNMR
CDCl3
150MHz
210 200 190 180 170 160 150 140 130 120 110 100 90 80 70 60 50 40 30 20 10

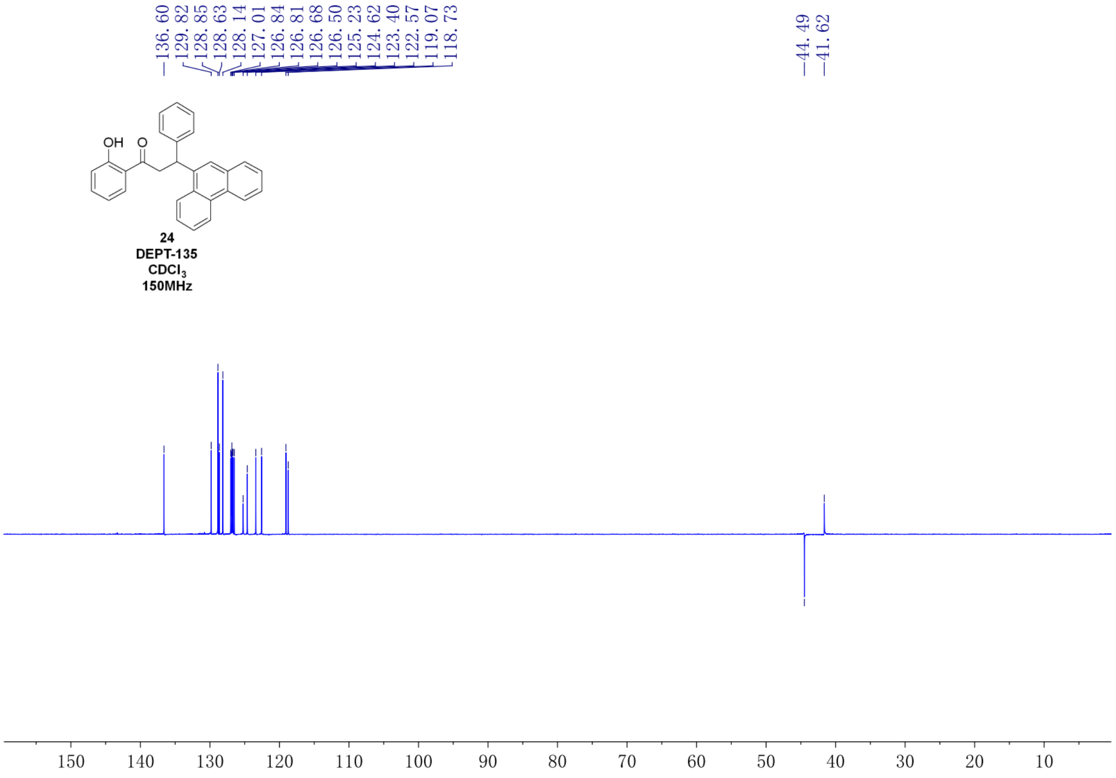
136.60
129.82
128.85
128.63
128.14
127.01
126.84
126.81
126.68
126.50
125.23
124.62
123.40
122.57
119.07
118.73
44.49
41.62
OH
O
24
DEPT-135
CDCl3
150MHz
150
140
130
120
110
100
90
80
70
60
50
40
30
20
10

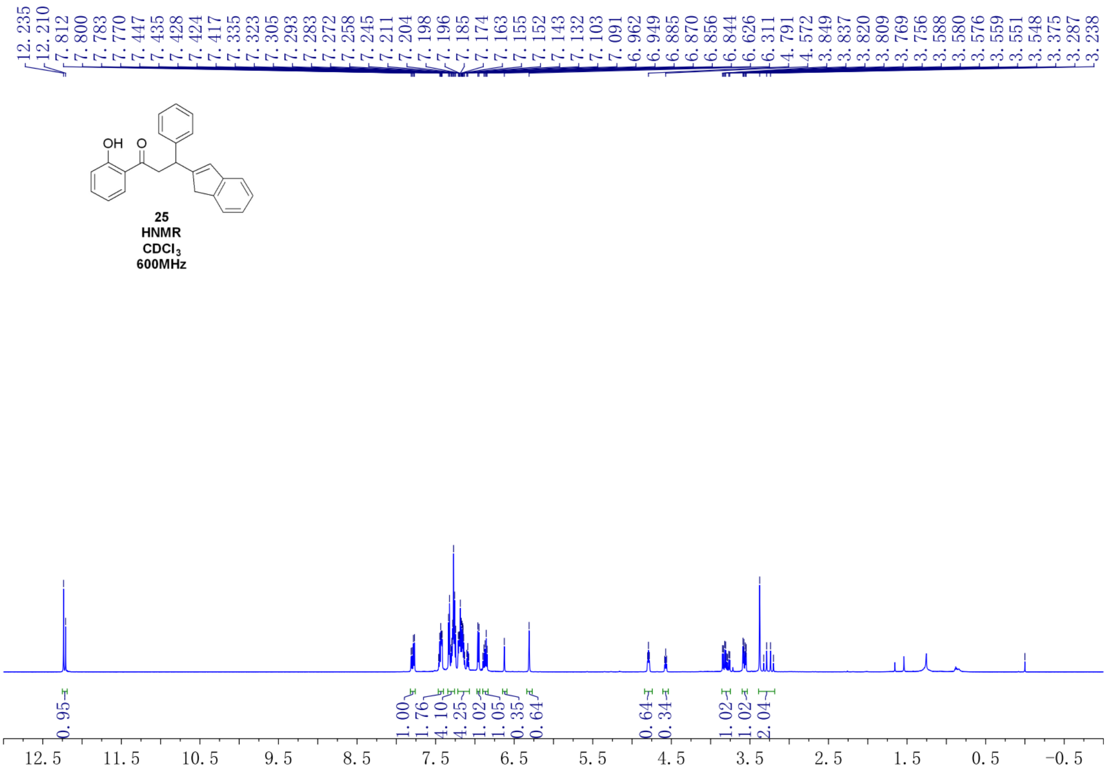
12.235
12.210
7.812
7.800
7.783
7.770
7.447
7.435
7.428
7.424
7.417
7.335
7.323
7.305
7.293
7.283
7.272
7.258
7.245
7.211
7.204
7.198
7.196
7.185
7.174
7.163
7.155
7.152
7.143
7.132
7.103
7.091
6.962
6.949
6.885
6.870
6.856
6.844
6.626
6.311
4.791
4.572
3.849
3.837
3.820
3.809
3.769
3.756
3.588
3.580
3.576
3.559
3.551
3.548
3.375
3.287
3.238
OH
O
25
HNMR
CDCl3
600MHz
0.95
1.00
1.76
4.10
4.25
1.02
1.05
0.35
0.64
0.64
0.34
1.02
1.02
2.04
12.5
11.5
10.5
9.5
8.5
7.5
6.5
5.5
4.5
3.5
2.5
1.5
0.5
-0.5

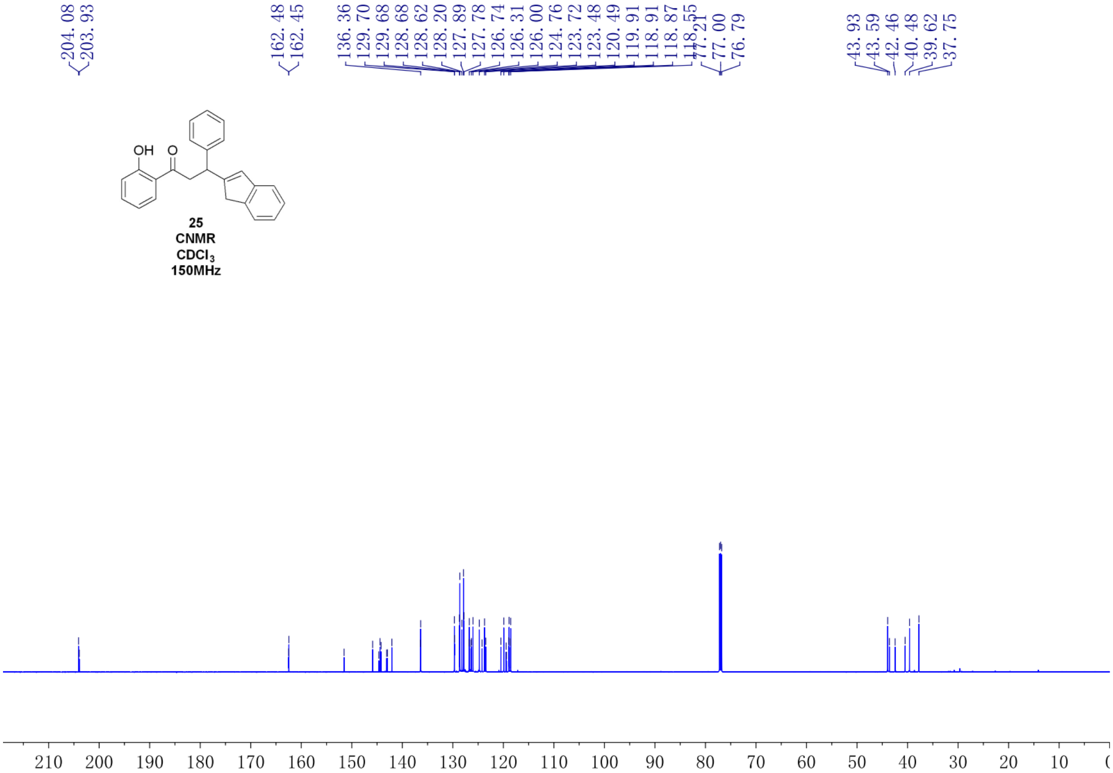
204.08
203.93
162.48
162.45
136.36
129.70
129.68
128.68
128.62
128.20
127.89
127.78
126.74
126.31
126.00
124.76
123.72
123.48
120.49
119.91
118.91
118.87
118.55
77.21
77.00
76.79
43.93
43.59
42.46
40.48
39.62
37.75
OH
O
25
CNMR
CDCl3
150MHz

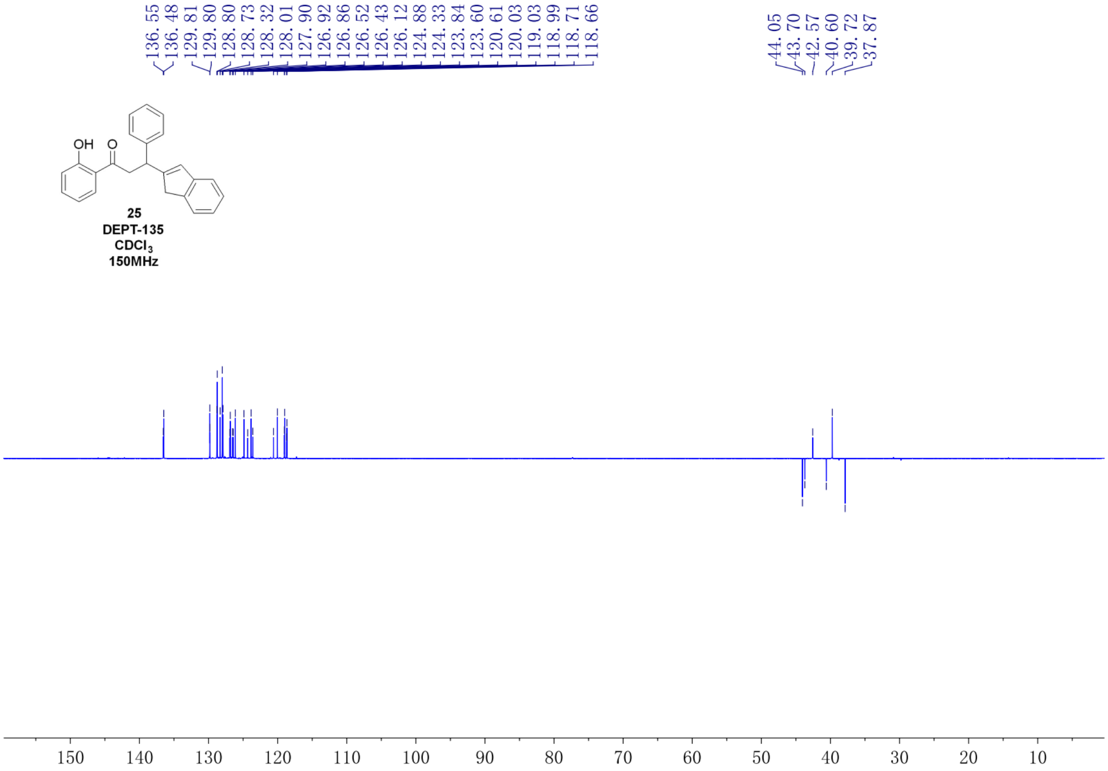
136.55
136.48
129.81
129.80
128.80
128.73
128.32
128.01
127.90
126.92
126.86
126.52
126.43
126.12
124.88
124.33
123.84
123.60
120.61
120.03
119.03
118.99
118.71
118.66
44.05
43.70
42.57
40.60
39.72
37.87
OH
O
25
DEPT-135
CDCl3
150MHz

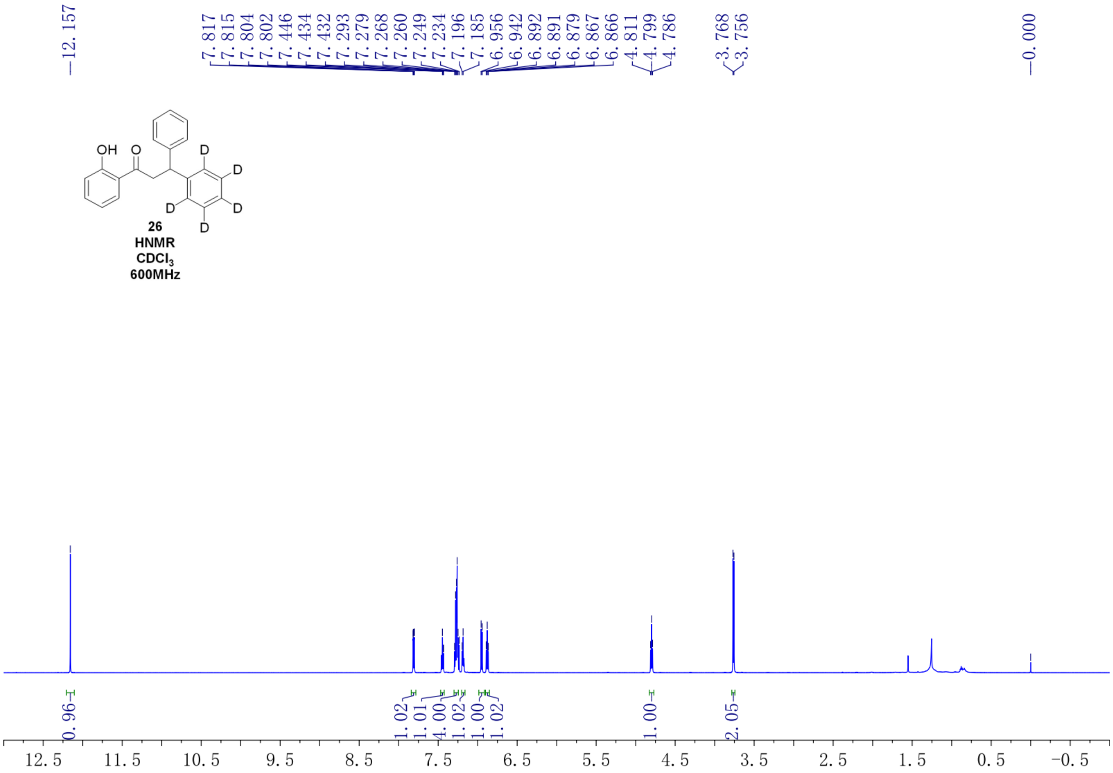
12.157
7.817 7.815 7.804 7.802 7.446 7.434 7.432 7.293 7.279 7.268 7.260 7.249 7.234 7.196 7.185 6.956 6.942 6.892 6.891 6.879 6.867 6.866 4.811 4.799 4.786
3.768 3.756
0.000
OH O D D D D D
26
HNMR
$CDCl_3$
600MHz
0.96 1.02 1.01 4.00 1.02 1.00 1.02 1.00 2.05
12.5 11.5 10.5 9.5 8.5 7.5 6.5 5.5 4.5 3.5 2.5 1.5 0.5 -0.5

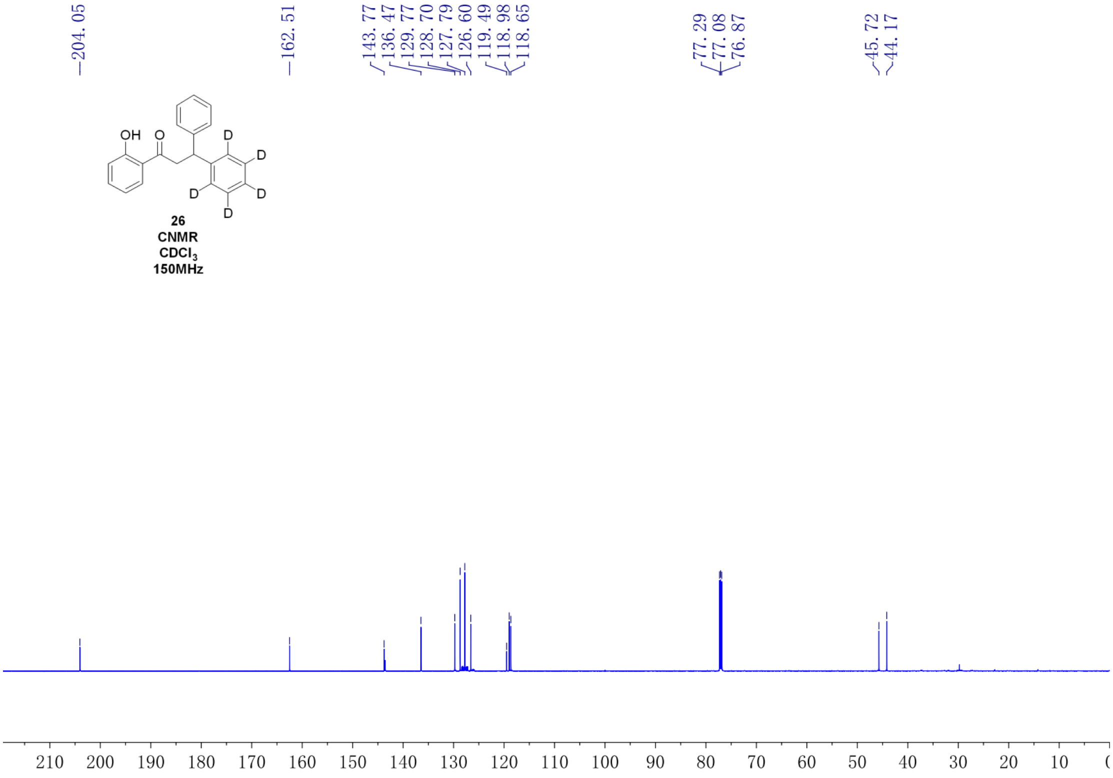
204.05
162.51
143.77 136.47 129.77 128.70 127.79 126.60 119.49 118.98 118.65
77.29 77.08 76.87
45.72 44.17
OH O D D D D D
26
CNMR
$CDCl_3$
150MHz
210 200 190 180 170 160 150 140 130 120 110 100 90 80 70 60 50 40 30 20 10

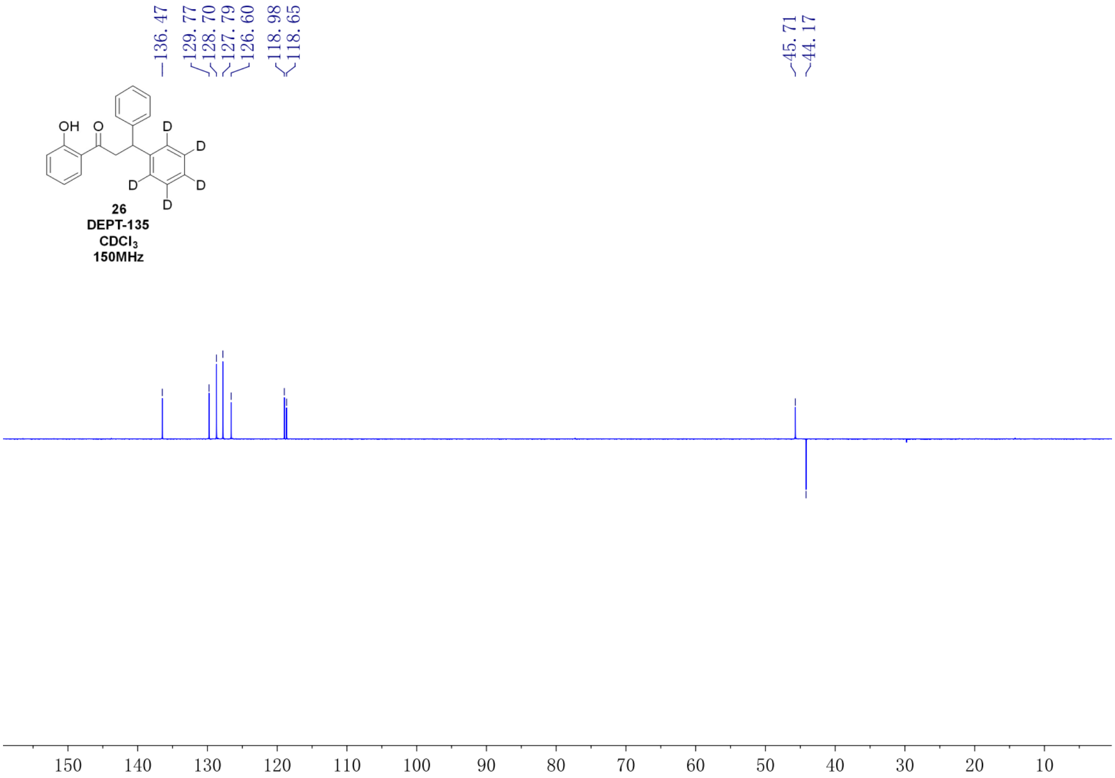
136.47
129.77
128.70
127.79
126.60
118.98
118.65
45.71
44.17
OH
O
D
26
DEPT-135
CDCl3
150MHz
150
140
130
120
110
100
90
80
70
60
50
40
30
20
10

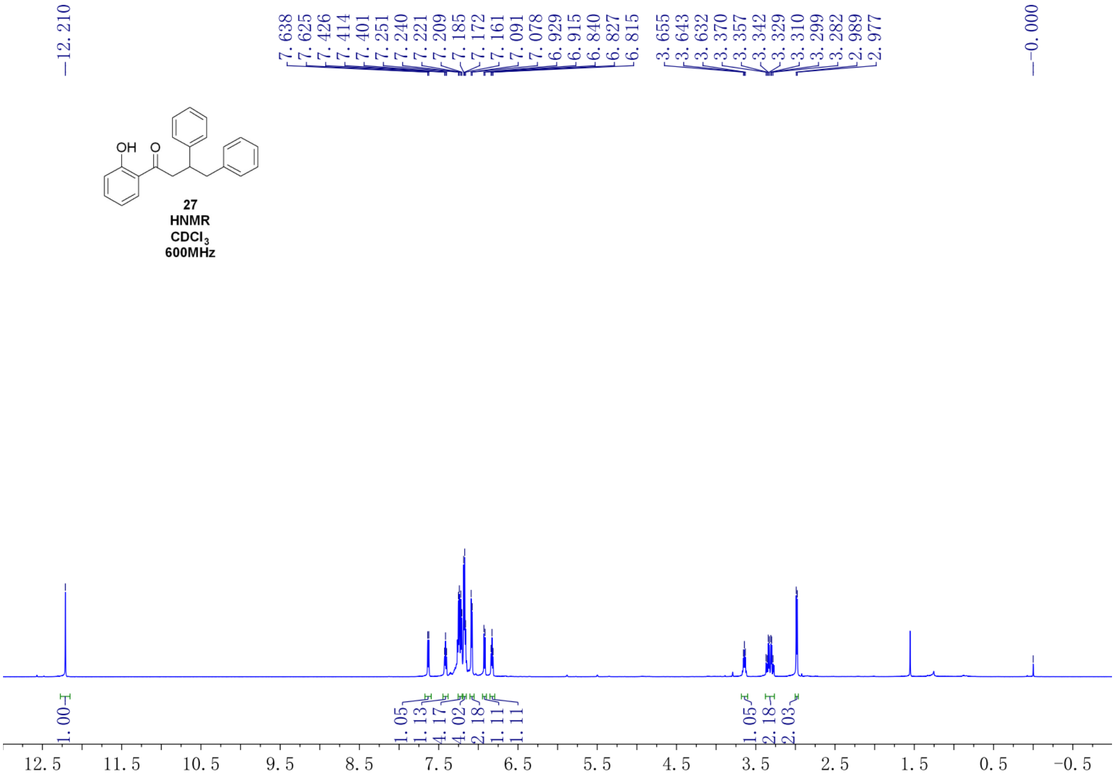
12.210
7.638
7.625
7.426
7.414
7.401
7.251
7.240
7.221
7.209
7.185
7.172
7.161
7.091
7.078
6.929
6.915
6.840
6.827
6.815
3.655
3.643
3.632
3.370
3.357
3.342
3.329
3.310
3.299
3.282
2.989
2.977
-0.000
OH
O
27
HNMR
CDCl3
600MHz
1.00
1.05
1.13
4.17
4.02
2.18
1.11
1.11
1.05
2.18
2.03
12.5
11.5
10.5
9.5
8.5
7.5
6.5
5.5
4.5
3.5
2.5
1.5
0.5
-0.5

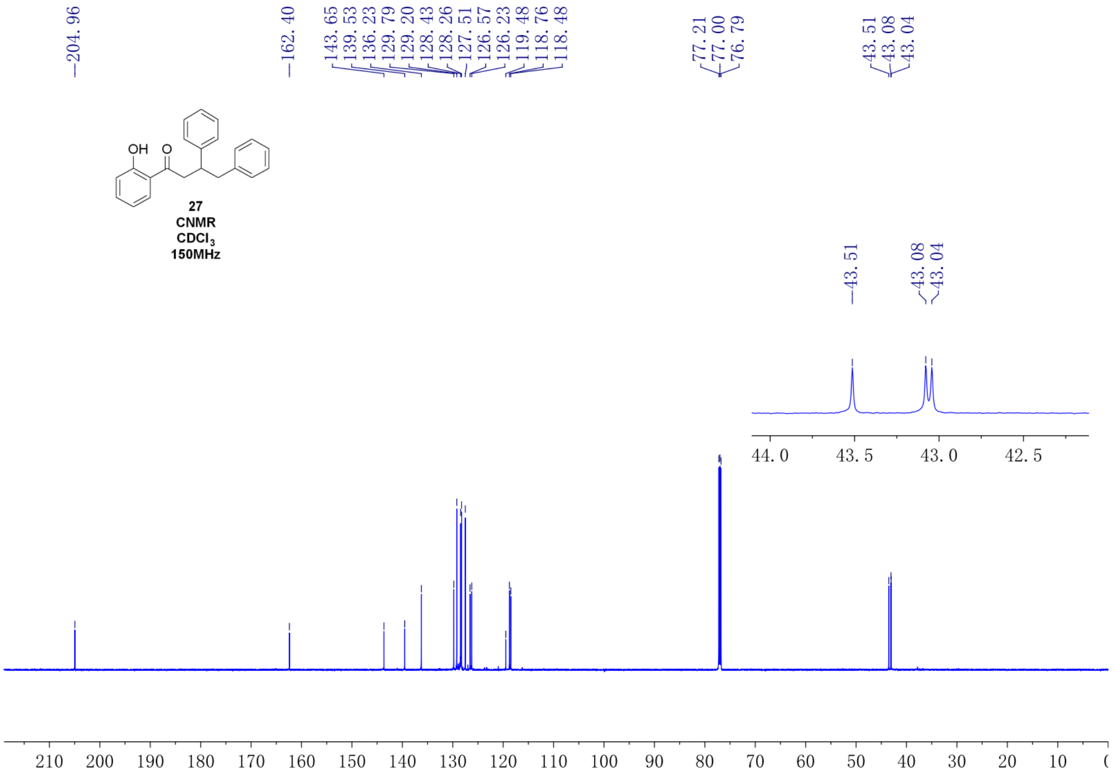
204.96
162.40
143.65
139.53
136.23
129.79
129.20
128.43
128.26
127.51
126.57
126.23
119.48
118.76
118.48
77.21
77.00
76.79
43.51
43.08
43.04
OH
O
27
CNMR
CDCl3
150MHz
43.51
43.08
43.04
44.0
43.5
43.0
42.5
210
200
190
180
170
160
150
140
130
120
110
100
90
80
70
60
50
40
30
20
10

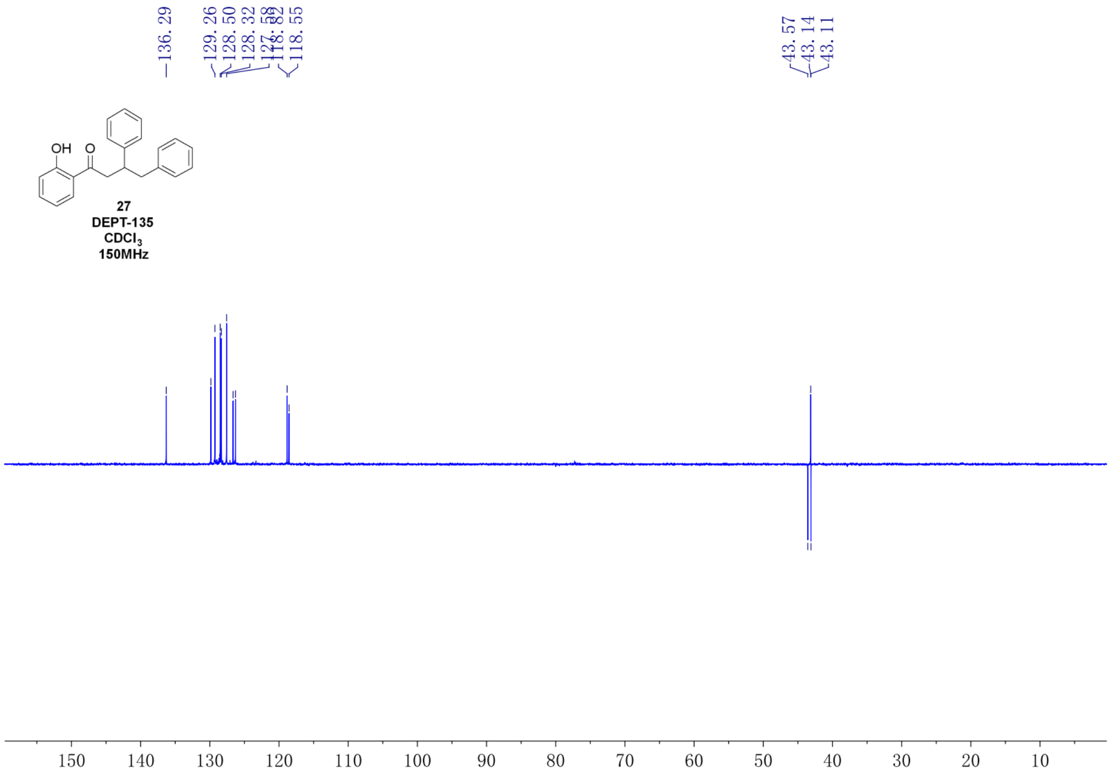
136.29
129.26
128.50
128.32
118.55
43.57
43.14
43.11
OH
O
27
DEPT-135
CDCl3
150MHz
150
140
130
120
110
100
90
80
70
60
50
40
30
20
10

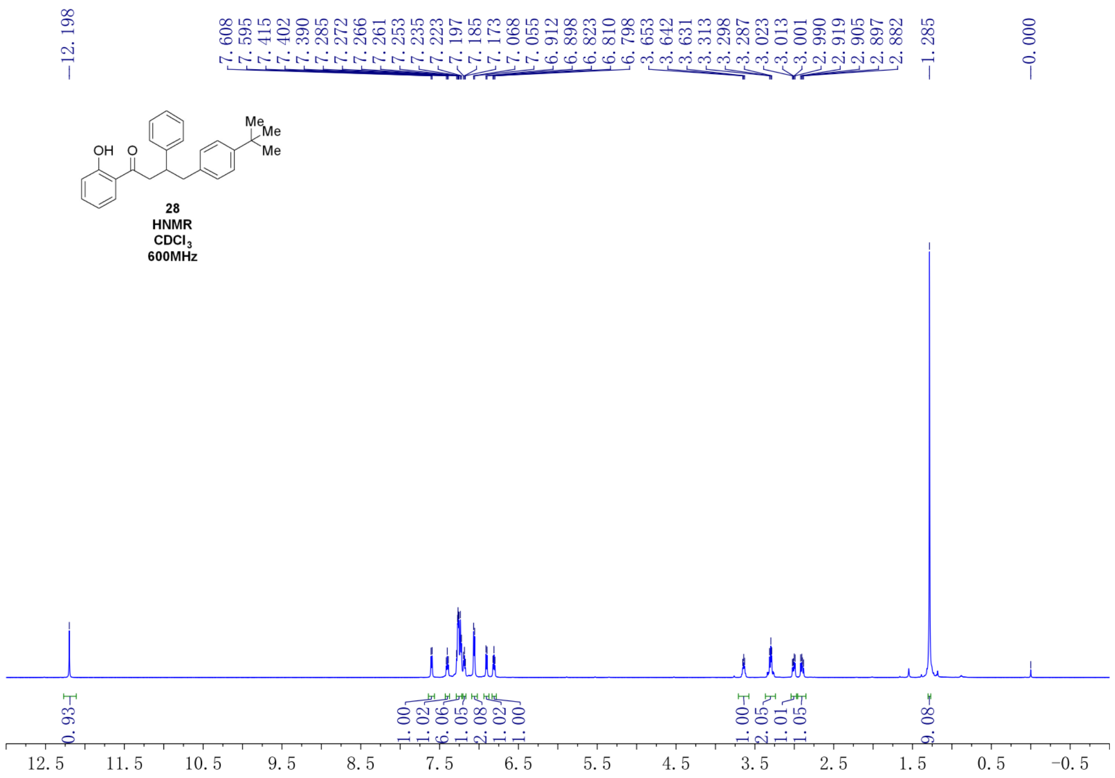
12.198
7.608
7.595
7.415
7.402
7.390
7.285
7.272
7.266
7.261
7.253
7.235
7.223
7.197
7.185
7.173
7.068
7.055
6.912
6.898
6.823
6.810
6.798
3.653
3.642
3.631
3.313
3.298
3.287
3.023
3.013
3.001
2.990
2.919
2.905
2.897
2.882
1.285
0.000
OH
O
Me
Me
Me
28
HNMR
$CDCl_3$
600MHz
0.93
1.00
1.02
6.06
1.05
2.08
1.02
1.00
1.00
2.05
1.01
1.05
9.08
12.5
11.5
10.5
9.5
8.5
7.5
6.5
5.5
4.5
3.5
2.5
1.5
0.5
-0.5

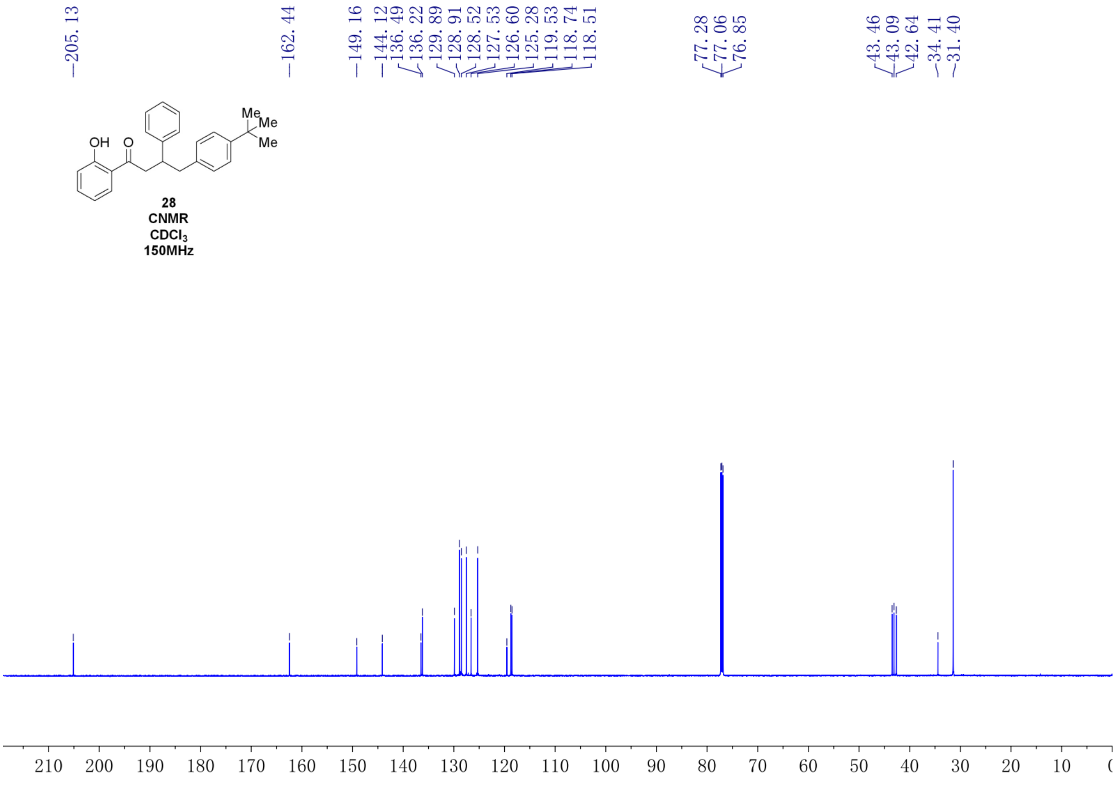
205.13
162.44
149.16
144.12
136.49
136.22
129.89
128.91
128.52
127.53
126.60
125.28
119.53
118.74
118.51
77.28
77.06
76.85
43.46
43.09
42.64
34.41
31.40
OH
O
Me
Me
Me
28
CNMR
$CDCl_3$
150MHz
210
200
190
180
170
160
150
140
130
120
110
100
90
80
70
60
50
40
30
20
10

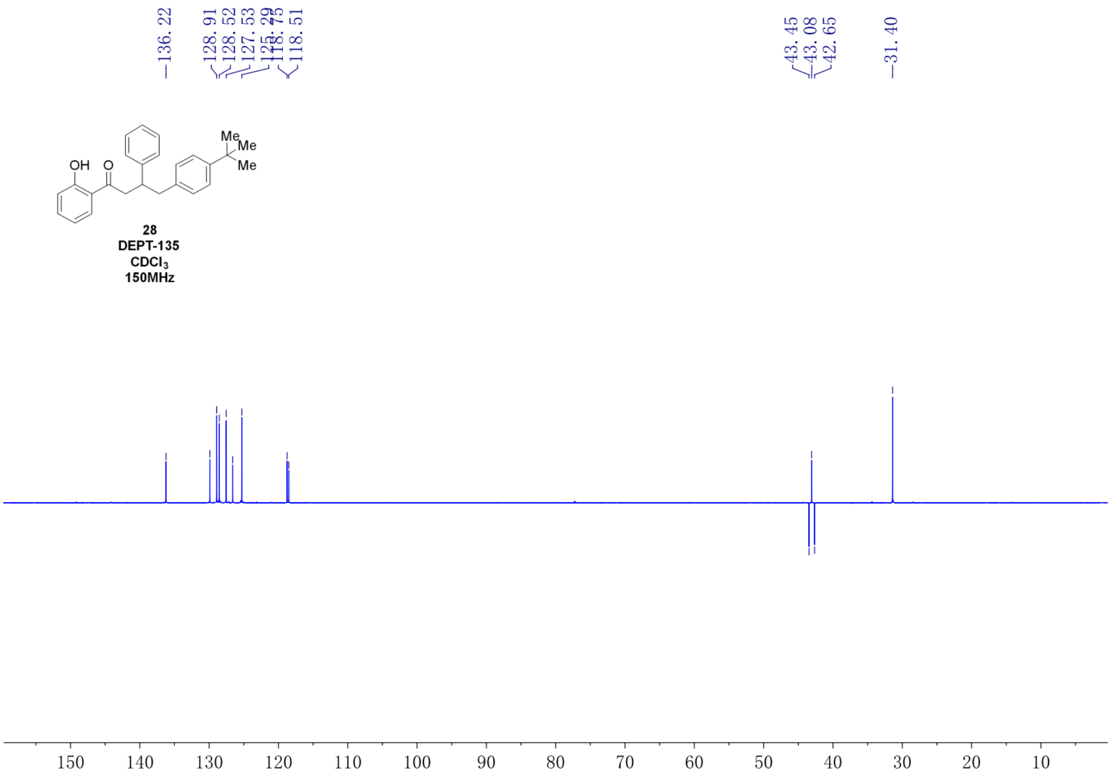
136.22
128.91
128.52
127.53
125.29
118.75
118.51
43.45
43.08
42.65
31.40
OH
O
Me
Me
Me
28
DEPT-135
$CDCl_3$
150MHz
150
140
130
120
110
100
90
80
70
60
50
40
30
20
10

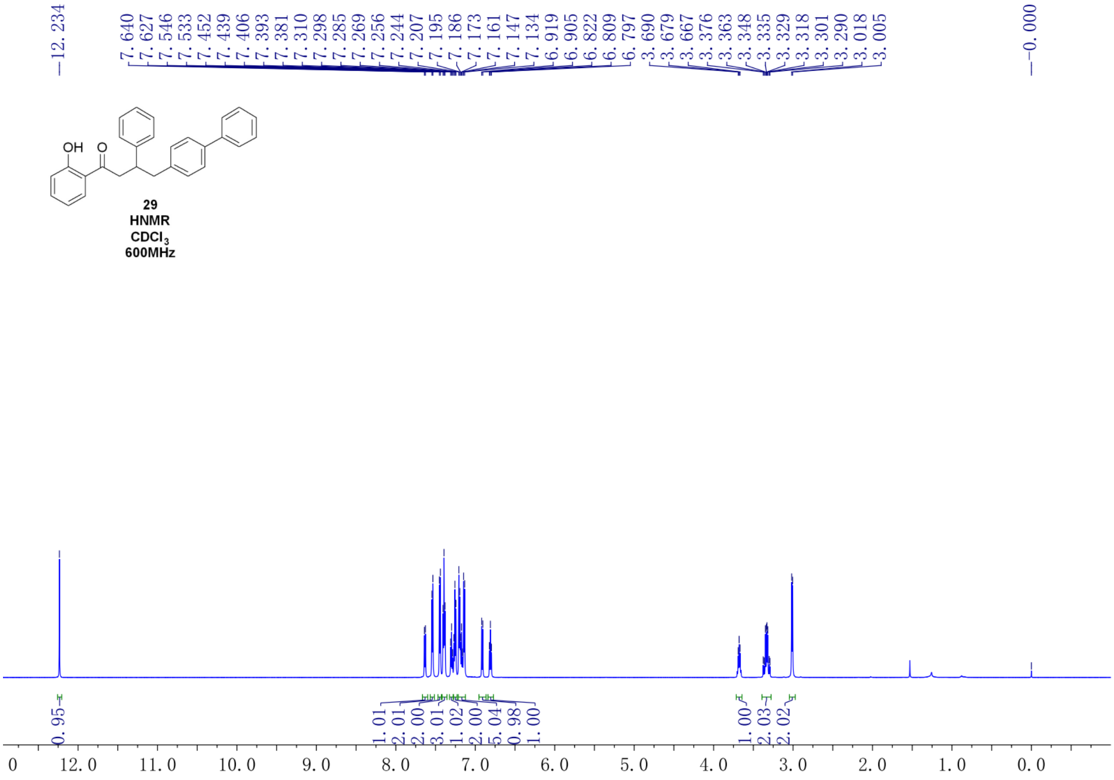
12.234
7.640
7.627
7.546
7.533
7.452
7.439
7.406
7.393
7.381
7.310
7.298
7.285
7.269
7.256
7.244
7.207
7.195
7.186
7.173
7.161
7.147
7.134
6.919
6.905
6.822
6.809
6.797
3.690
3.679
3.667
3.376
3.363
3.348
3.335
3.329
3.318
3.301
3.290
3.018
3.005
0.000
OH
O
29
HNMR
$CDCl_3$
600MHz
0.95
1.01
2.01
2.00
3.01
1.02
2.00
5.04
0.98
1.00
1.00
2.03
2.02
12.0
11.0
10.0
9.0
8.0
7.0
6.0
5.0
4.0
3.0
2.0
1.0
0.0

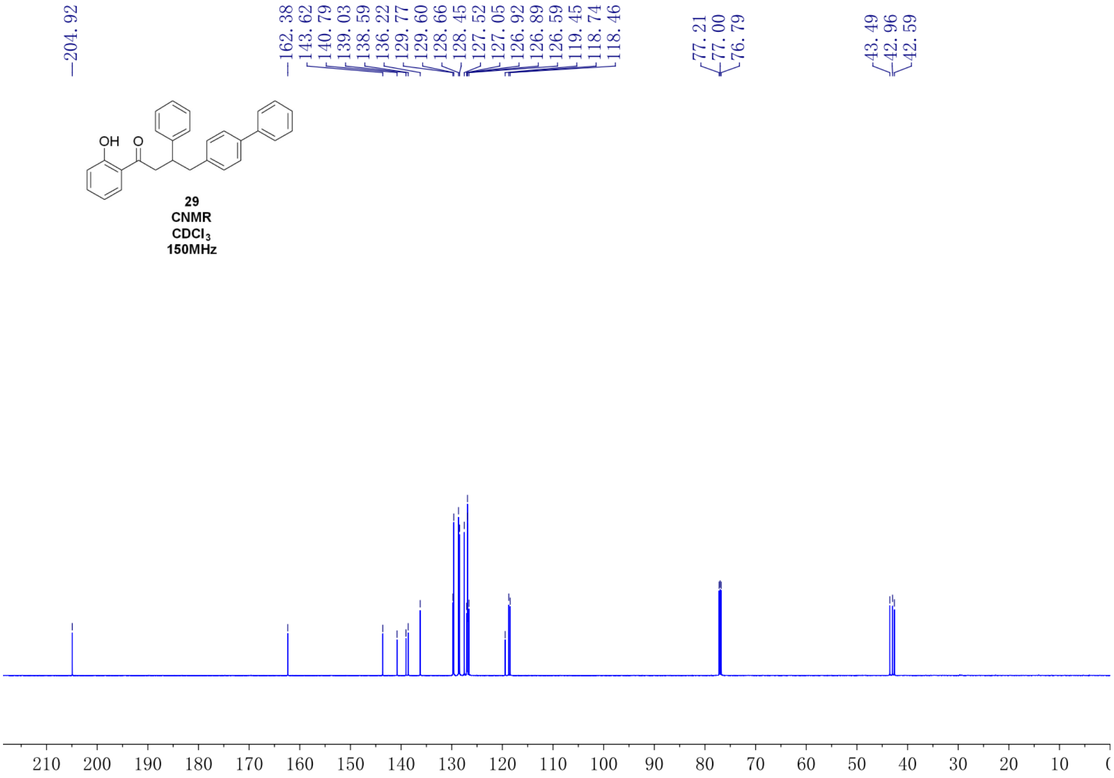
204.92
162.38
143.62
140.79
139.03
138.59
136.22
129.77
129.60
128.66
128.45
127.52
127.05
126.92
126.89
126.59
119.45
118.74
118.46
77.21
77.00
76.79
43.49
42.96
42.59
OH
O
29
CNMR
CDCl3
150MHz
210 200 190 180 170 160 150 140 130 120 110 100 90 80 70 60 50 40 30 20 10

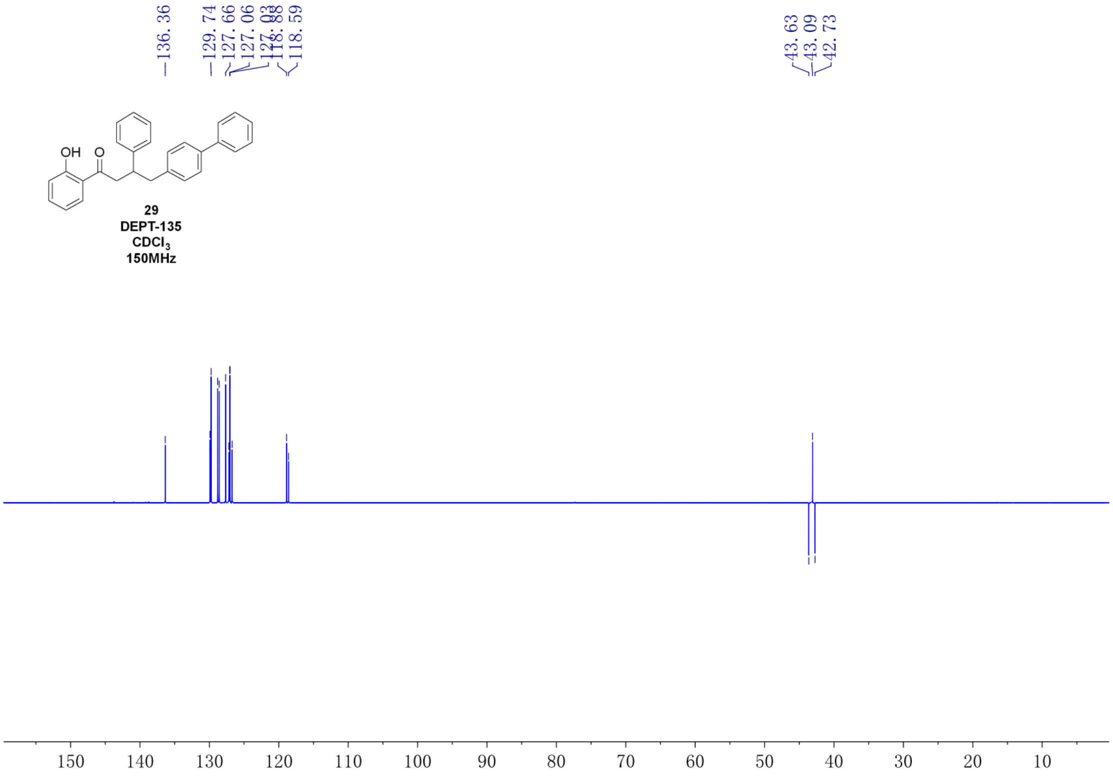
136.36
129.74
127.66
127.06
118.59
43.63
43.09
42.73
OH
O
29
DEPT-135
CDCl3
150MHz
150 140 130 120 110 100 90 80 70 60 50 40 30 20 10

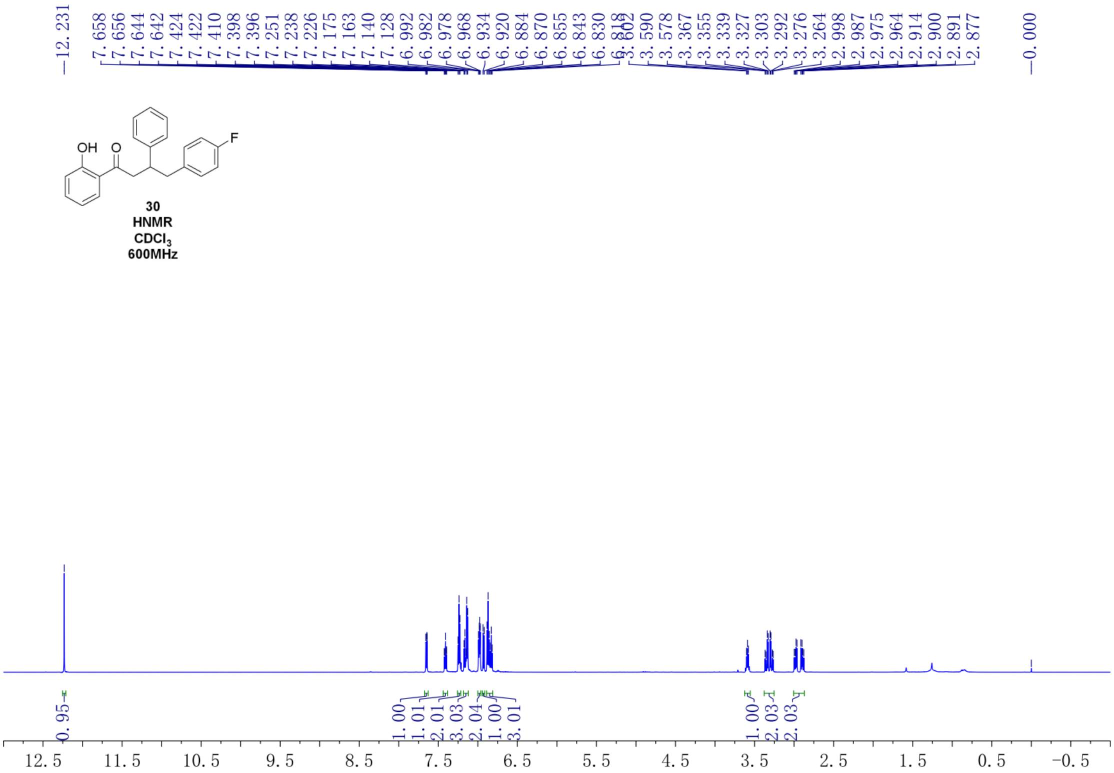
12.231
7.658
7.656
7.644
7.642
7.424
7.422
7.410
7.398
7.396
7.251
7.238
7.226
7.175
7.163
7.140
7.128
6.992
6.982
6.978
6.968
6.934
6.920
6.884
6.870
6.855
6.843
6.830
3.590
3.578
3.367
3.355
3.339
3.327
3.303
3.292
3.276
3.264
2.998
2.987
2.975
2.964
2.914
2.900
2.891
2.877
0.000
OH
O
F
30
HNMR
CDCl3
600MHz
0.95
1.00
1.01
2.01
3.03
2.04
1.00
3.01
1.00
2.03
2.03
12.5
11.5
10.5
9.5
8.5
7.5
6.5
5.5
4.5
3.5
2.5
1.5
0.5
-0.5

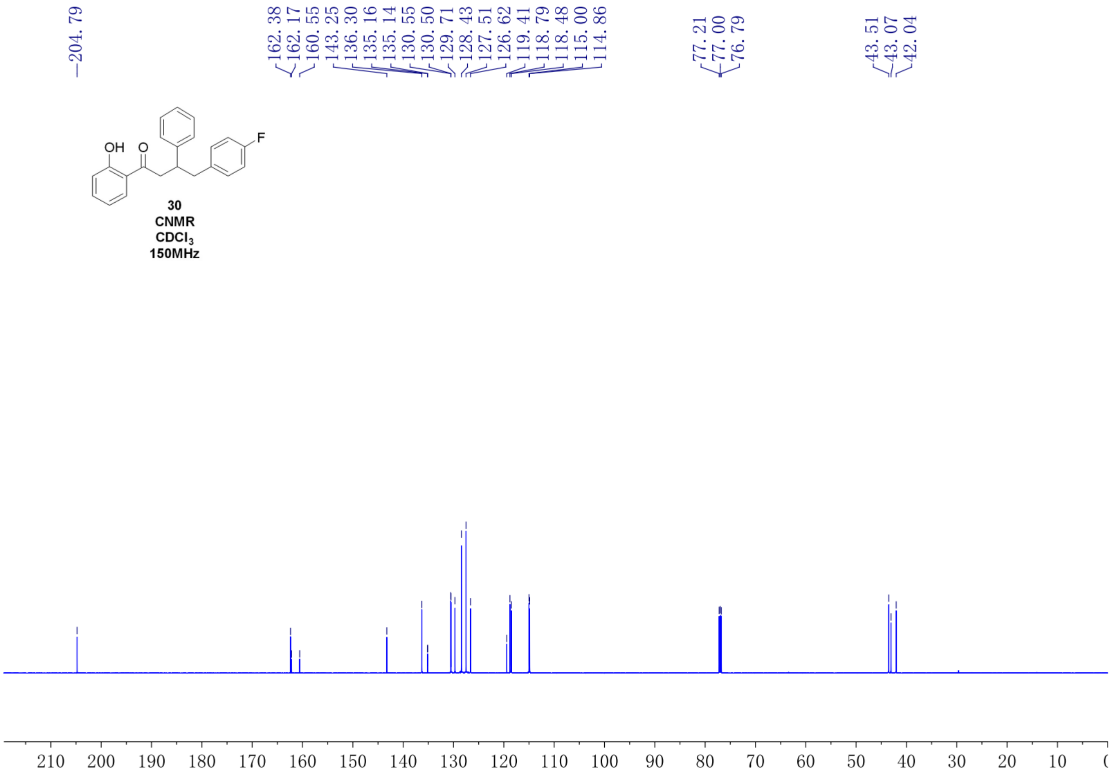
204.79
162.38
162.17
160.55
143.25
136.30
135.16
135.14
130.55
130.50
129.71
128.43
127.51
126.62
119.41
118.79
118.48
115.00
114.86
77.21
77.00
76.79
43.51
43.07
42.04
OH
O
F
30
CNMR
CDCl3
150MHz
210
200
190
180
170
160
150
140
130
120
110
100
90
80
70
60
50
40
30
20
10

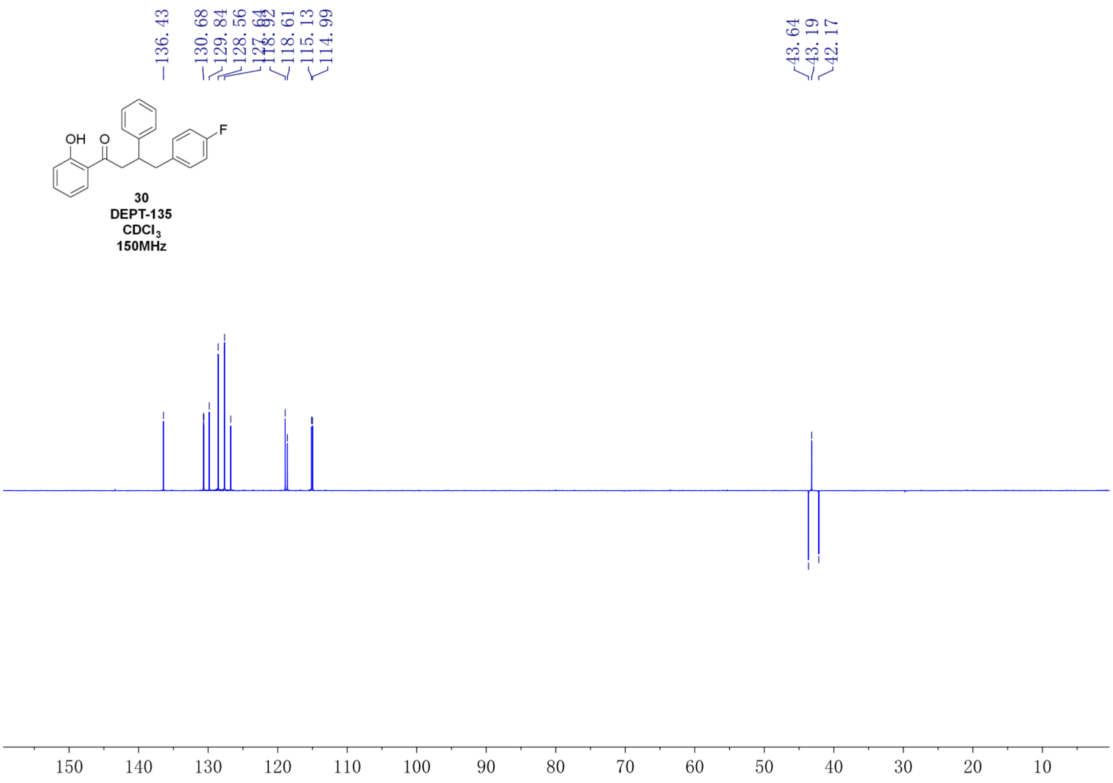

136.43
130.68
129.84
128.56
127.64
126.92
118.61
115.13
114.99
43.64
43.19
42.17
OH
O
F
30
DEPT-135
CDCl3
150MHz
150
140
130
120
110
100
90
80
70
60
50
40
30
20
10

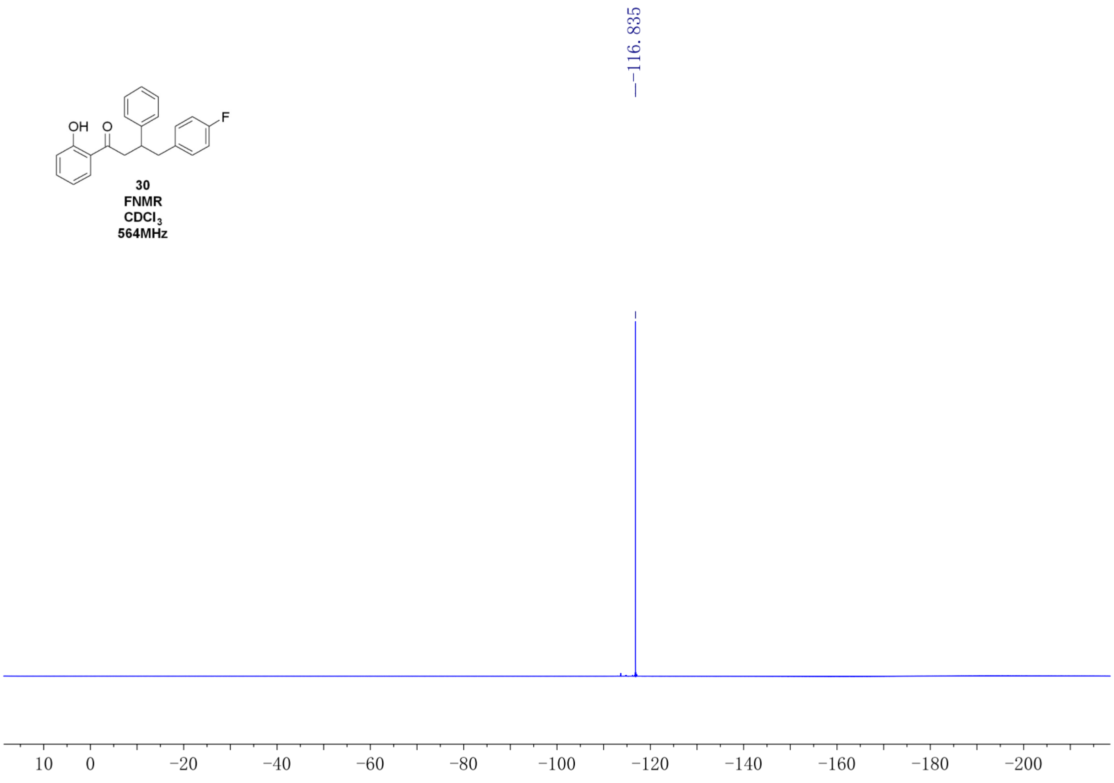

−116.835
OH
O
F
30
FNMR
CDCl3
564MHz
10
0
−20
−40
−60
−80
−100
−120
−140
−160
−180
−200

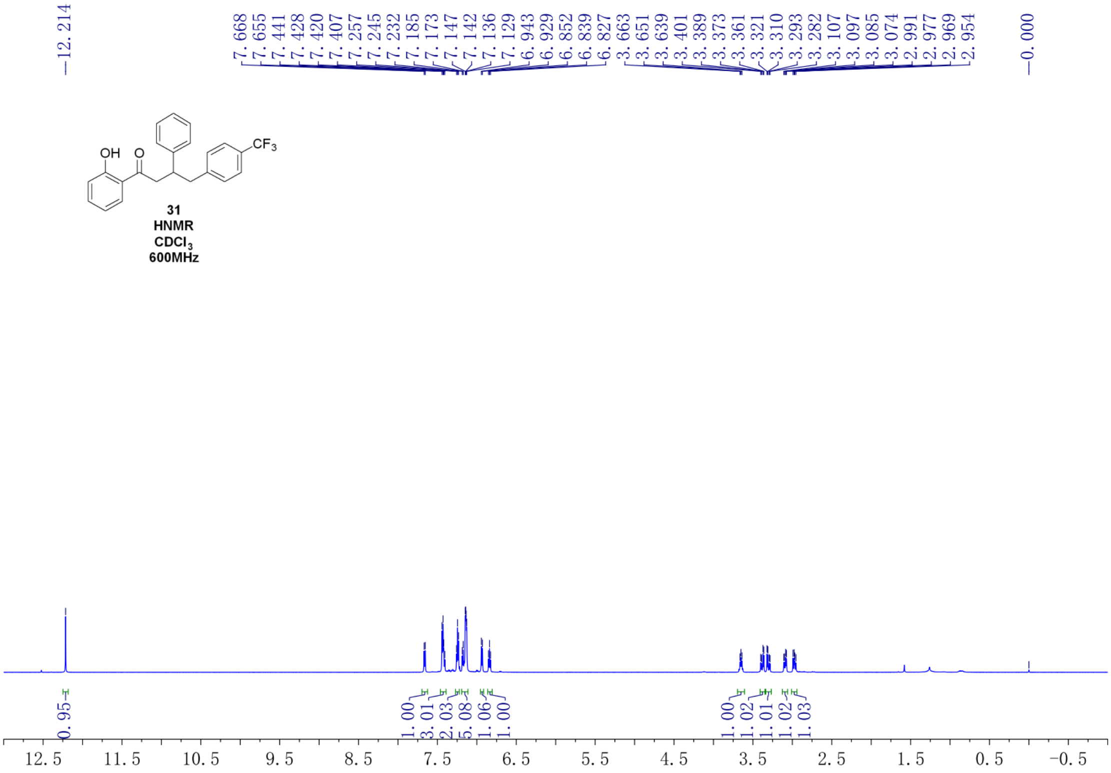
12.214
7.668 7.655 7.441 7.428 7.420 7.407 7.257 7.245 7.232 7.185 7.173 7.147 7.142 7.136 7.129 6.943 6.929 6.852 6.839 6.827 3.663 3.651 3.639 3.401 3.389 3.373 3.361 3.321 3.310 3.293 3.282 3.107 3.097 3.085 3.074 2.991 2.977 2.969 2.954
0.000
OH
O
CF3
31
HNMR
CDCl3
600MHz
0.95
1.00 3.01 2.03 5.08 1.06 1.00
1.00 1.02 1.01 1.02 1.03
12.5 11.5 10.5 9.5 8.5 7.5 6.5 5.5 4.5 3.5 2.5 1.5 0.5 -0.5

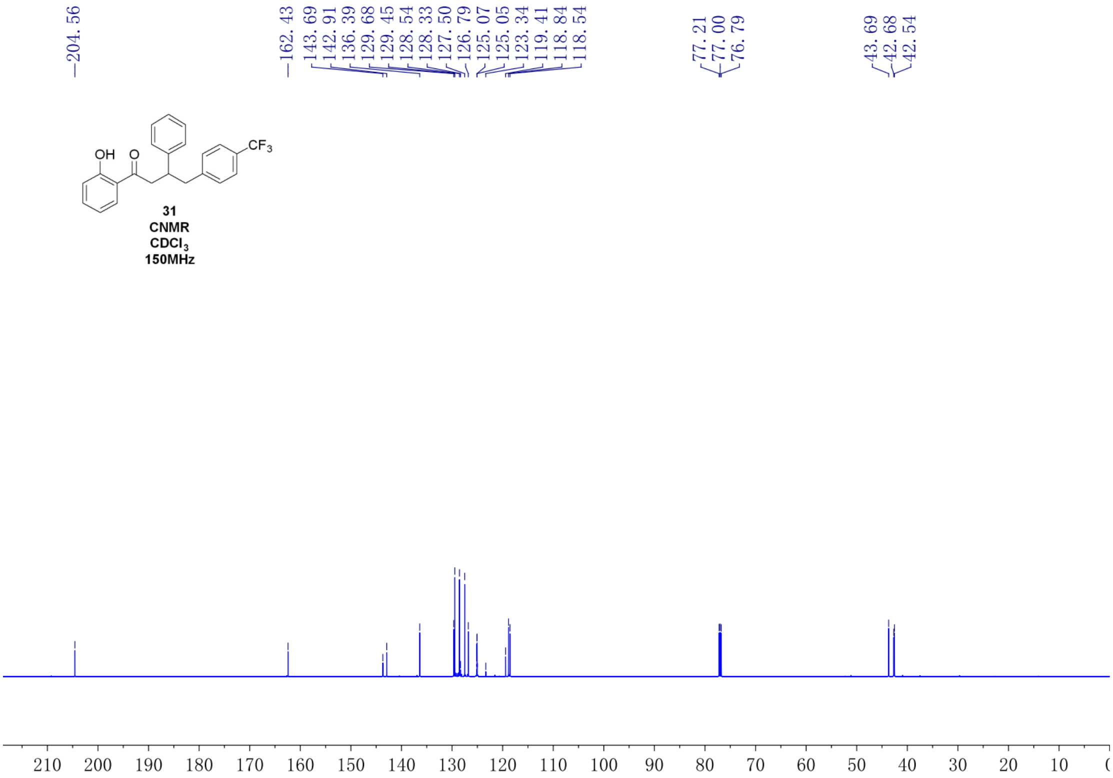
204.56
162.43
143.69 142.91 136.39 129.68 129.45 128.54 128.33 127.50 126.79 125.07 125.05 123.34 119.41 118.84 118.54
77.21 77.00 76.79
43.69 42.68 42.54
OH
O
CF3
31
CNMR
CDCl3
150MHz
210 200 190 180 170 160 150 140 130 120 110 100 90 80 70 60 50 40 30 20 10

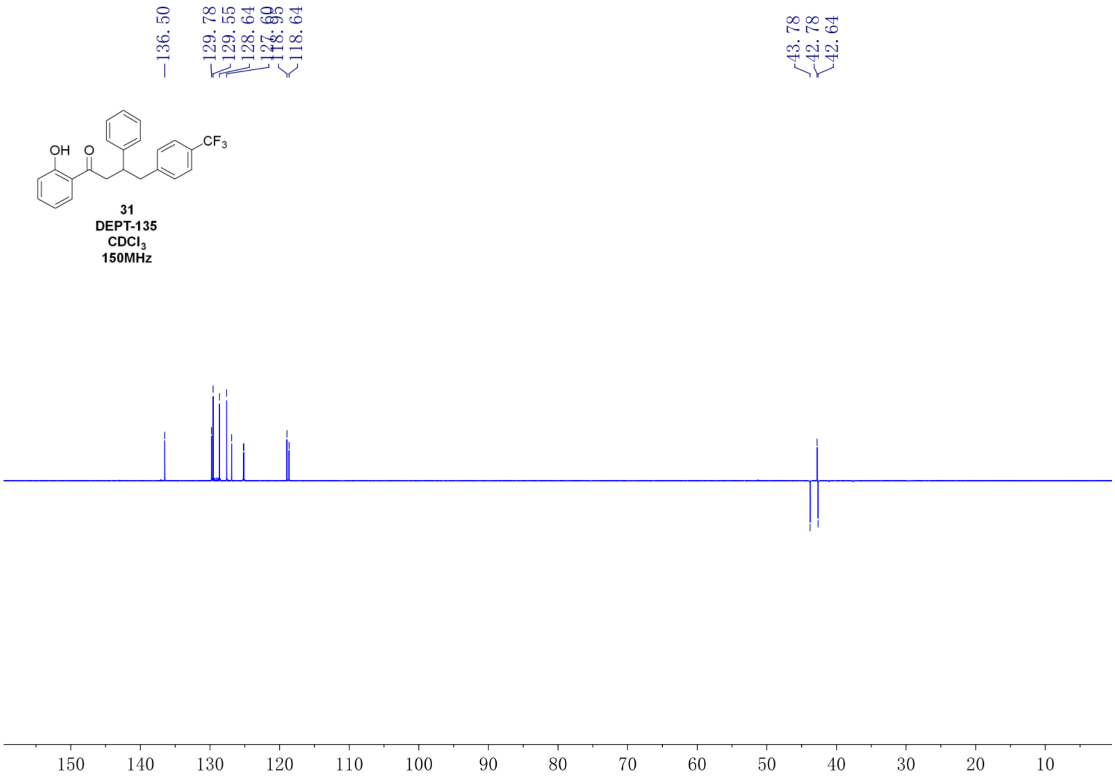
136.50
129.78
129.55
128.64
127.60
118.95
118.64
43.78
42.78
42.64
OH
O
CF3
31
DEPT-135
CDCl3
150MHz
150
140
130
120
110
100
90
80
70
60
50
40
30
20
10

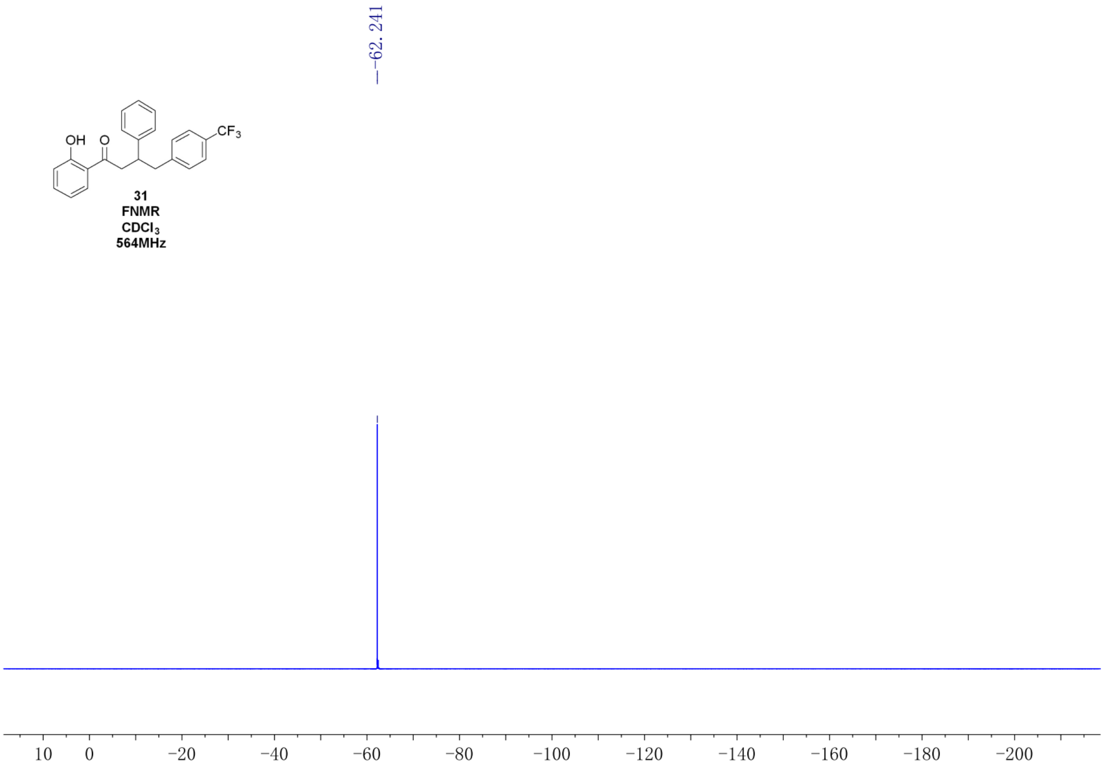
−62.241
OH
O
CF3
31
FNMR
CDCl3
564MHz
10
0
−20
−40
−60
−80
−100
−120
−140
−160
−180
−200

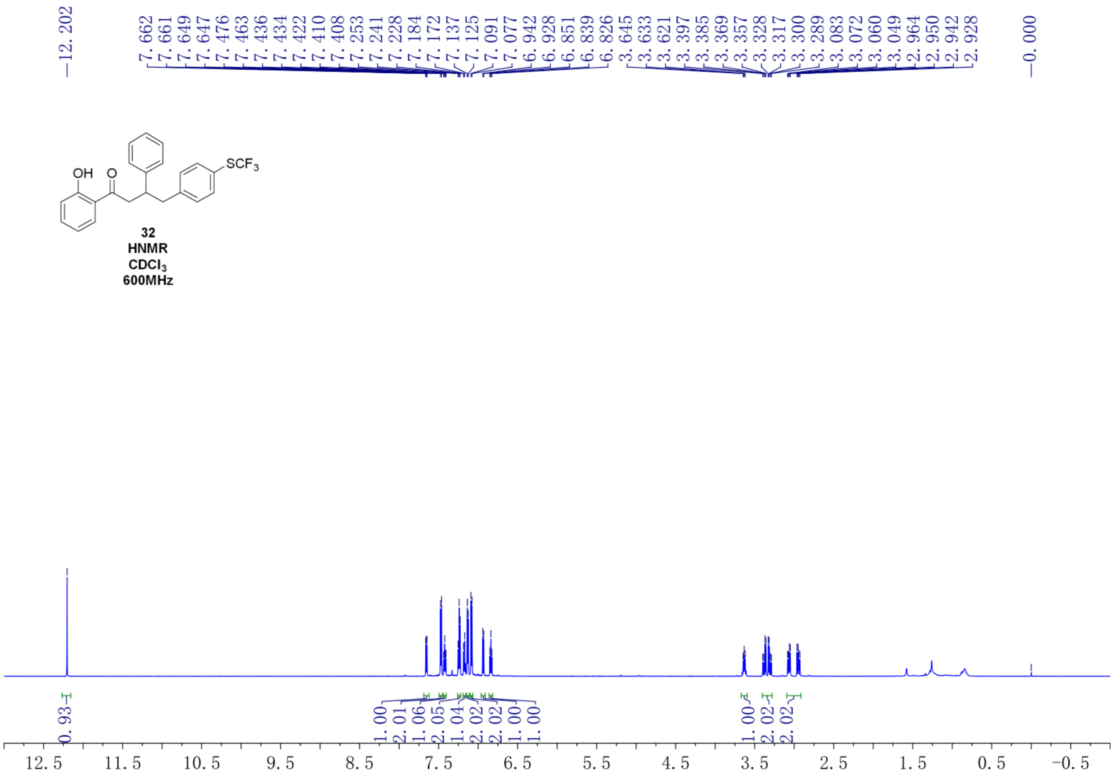

12.202
7.662
7.661
7.649
7.647
7.476
7.463
7.436
7.434
7.422
7.410
7.408
7.253
7.241
7.228
7.184
7.172
7.137
7.125
7.091
7.077
6.942
6.928
6.851
6.839
6.826
3.645
3.633
3.621
3.397
3.385
3.369
3.357
3.328
3.317
3.300
3.289
3.083
3.072
3.060
3.049
2.964
2.950
2.942
2.928
0.000
OH
O
SCF_3
32
HNMR
$CDCl_3$
600MHz
0.93
1.00
2.01
1.06
2.05
1.04
2.02
2.02
1.00
1.00
1.00
2.02
2.02
12.5
11.5
10.5
9.5
8.5
7.5
6.5
5.5
4.5
3.5
2.5
1.5
0.5
-0.5

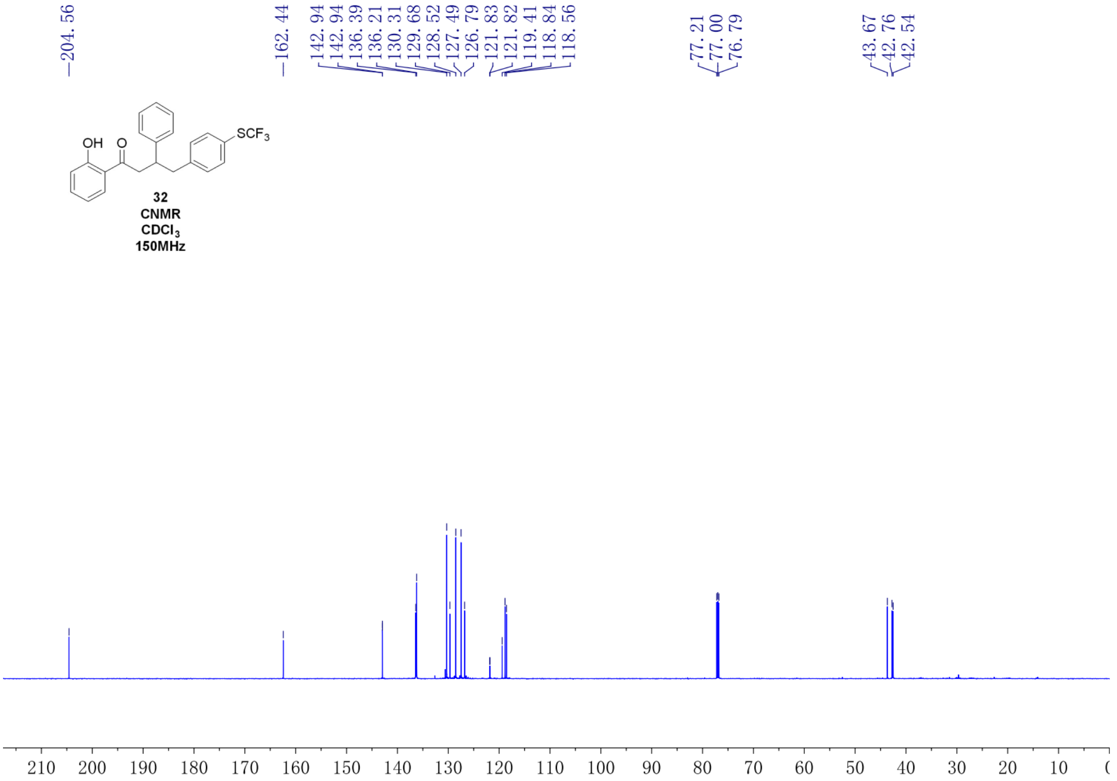

204.56
162.44
142.94
142.94
136.39
136.21
130.31
129.68
128.52
127.49
126.79
121.83
121.82
119.41
118.84
118.56
77.21
77.00
76.79
43.67
42.76
42.54
OH
O
SCF_3
32
CNMR
$CDCl_3$
150MHz
210
200
190
180
170
160
150
140
130
120
110
100
90
80
70
60
50
40
30
20
10

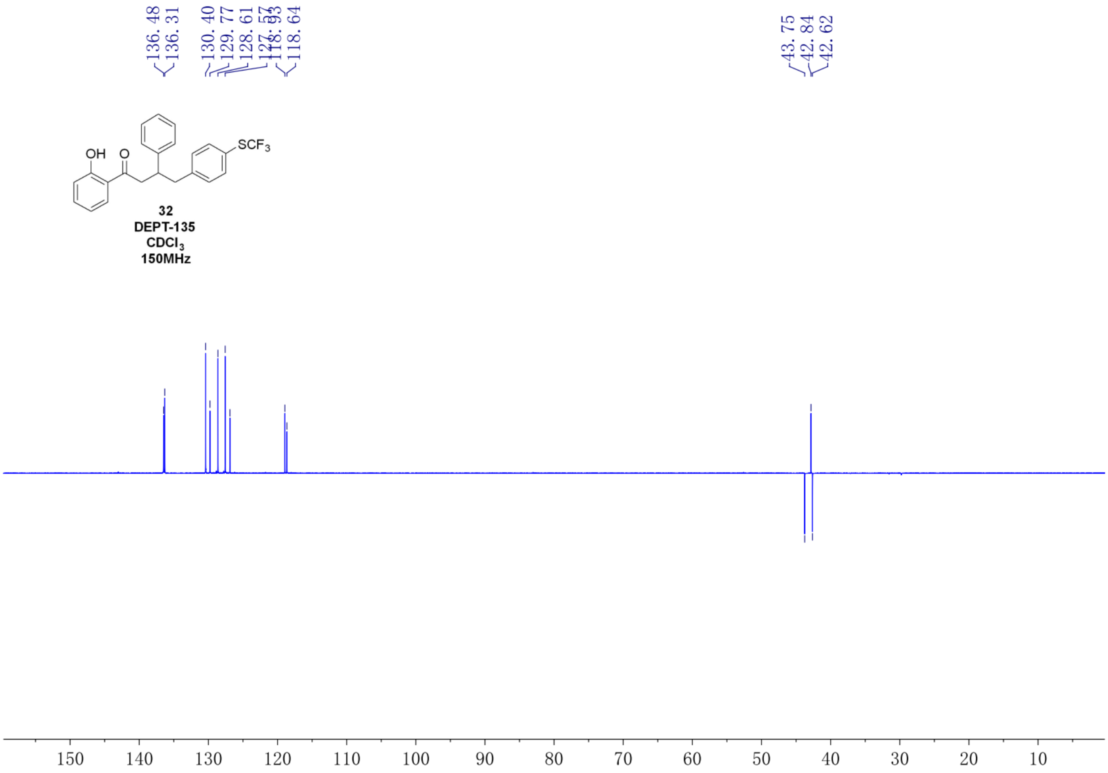

32
DEPT-135
$CDCl_3$
150MHz

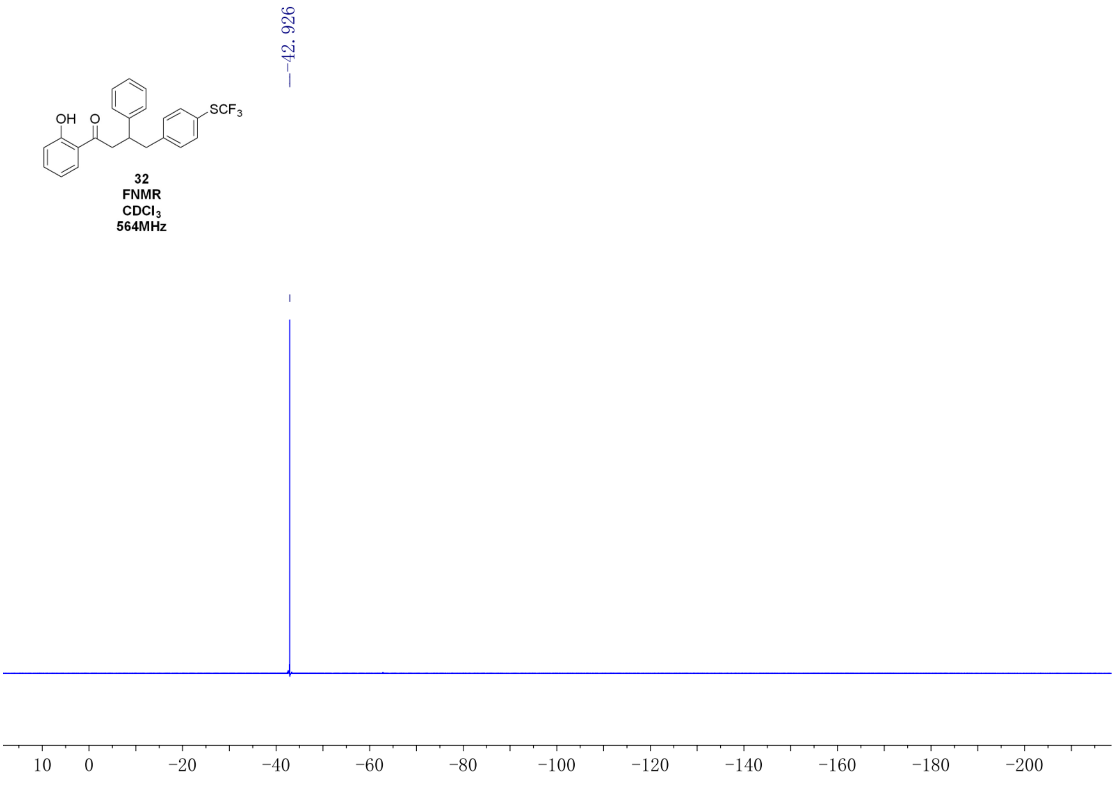

32
FNMR
$CDCl_3$
564MHz

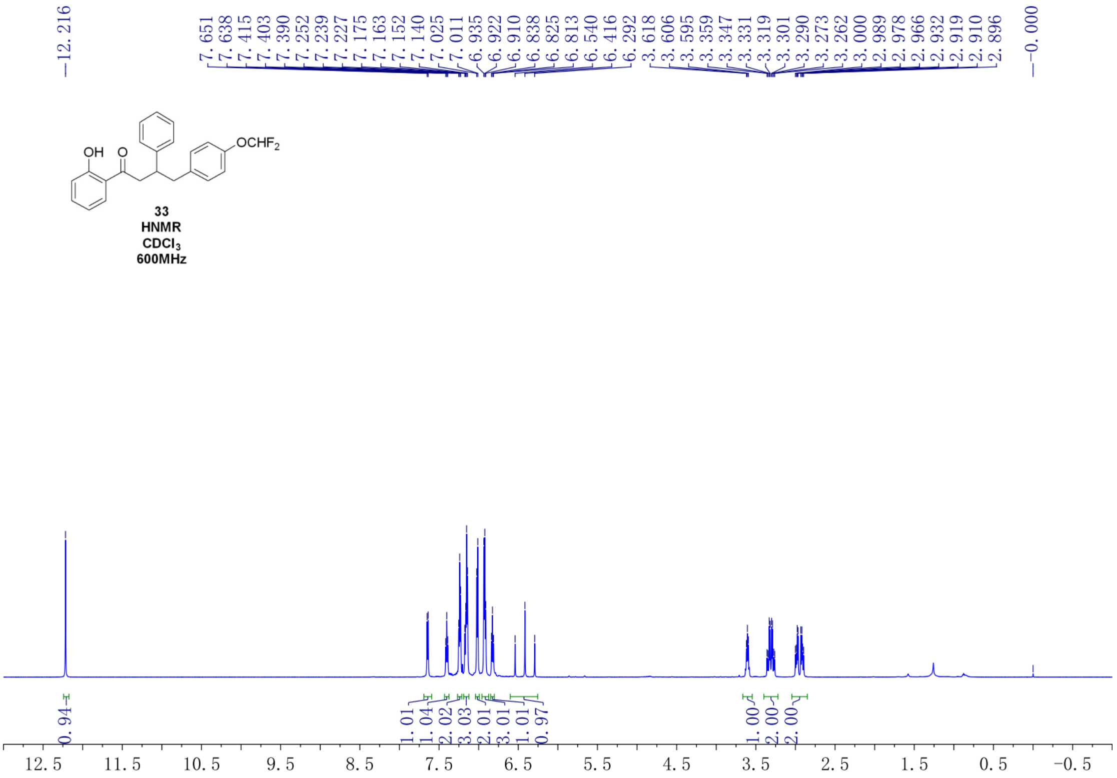
12.216
7.651
7.638
7.415
7.403
7.390
7.252
7.239
7.227
7.175
7.163
7.152
7.140
7.025
7.011
6.935
6.922
6.910
6.838
6.825
6.813
6.540
6.416
6.292
3.618
3.606
3.595
3.359
3.347
3.331
3.319
3.301
3.290
3.273
3.262
3.000
2.989
2.978
2.966
2.932
2.919
2.910
2.896
-0.000
OH
O
OCHF2
33
HNMR
CDCl3
600MHz
0.94
1.01
1.04
2.02
3.03
2.01
3.01
1.01
0.97
1.00
2.00
2.00
12.5
11.5
10.5
9.5
8.5
7.5
6.5
5.5
4.5
3.5
2.5
1.5
0.5
-0.5

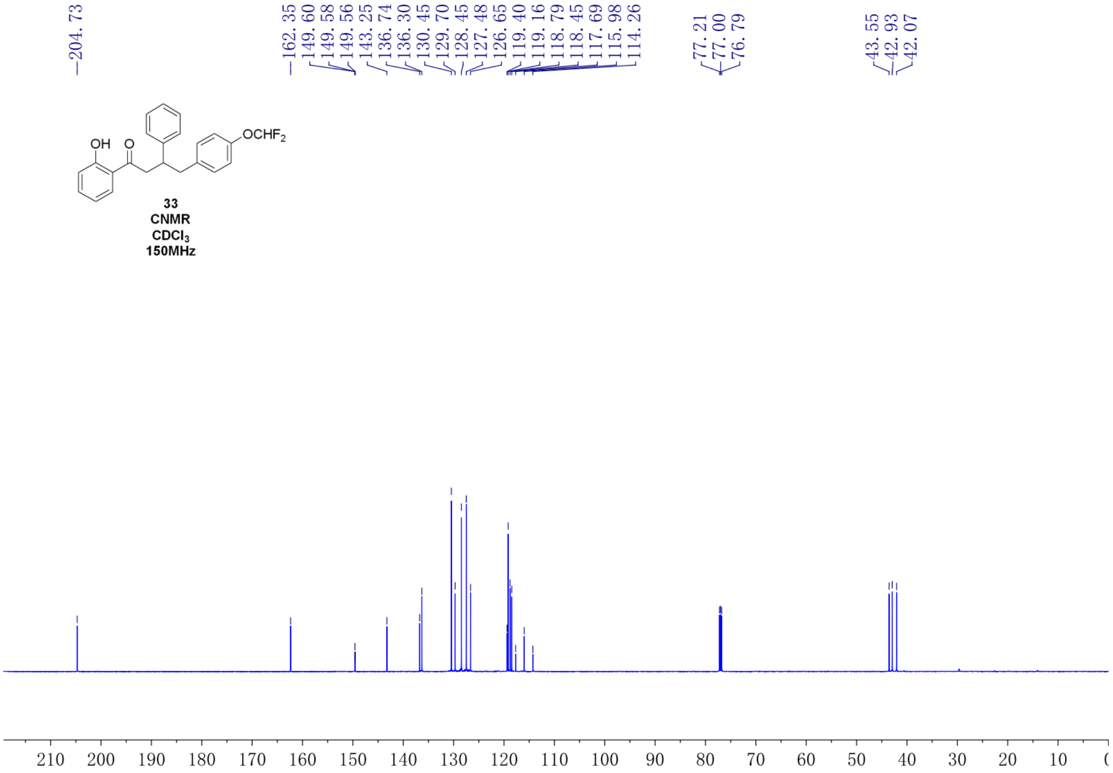
204.73
162.35
149.60
149.58
149.56
143.25
136.74
136.30
130.45
129.70
128.45
127.48
126.65
119.40
119.16
118.79
118.45
117.69
115.98
114.26
77.21
77.00
76.79
43.55
42.93
42.07
OH
O
OCHF2
33
CNMR
CDCl3
150MHz
210
200
190
180
170
160
150
140
130
120
110
100
90
80
70
60
50
40
30
20
10

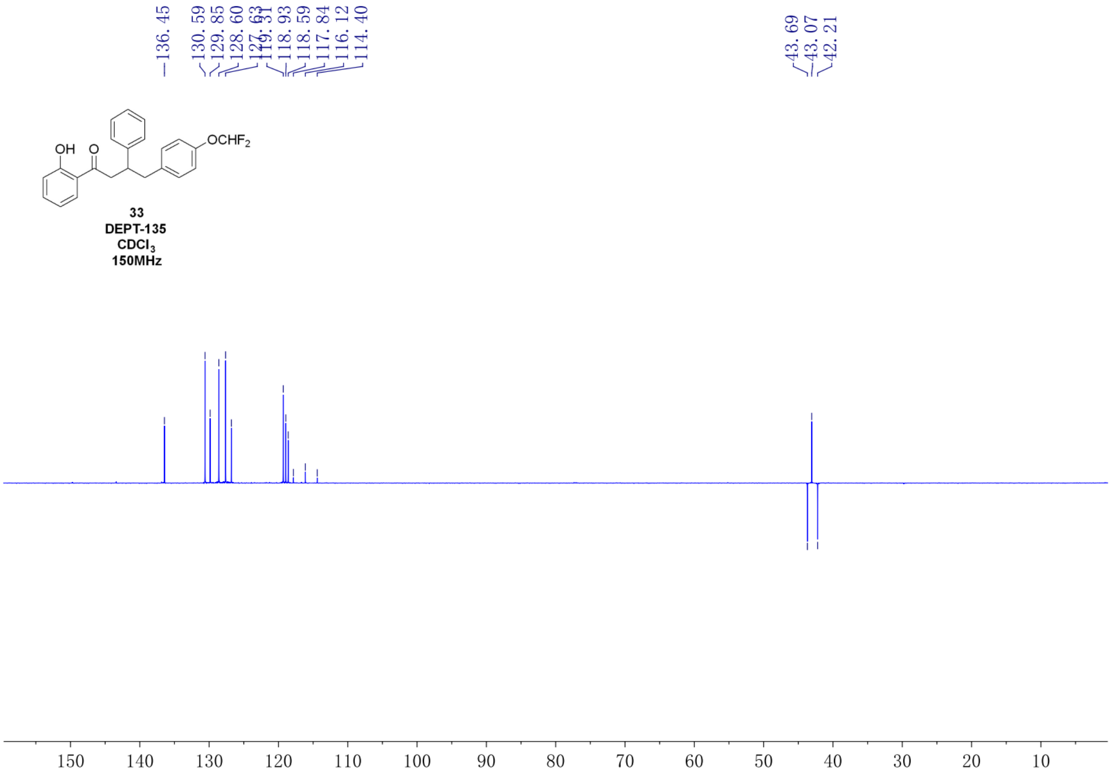
136.45
130.59
129.85
128.60
127.63
119.31
118.93
118.59
117.84
116.12
114.40
43.69
43.07
42.21
OH
O
OCHF2
33
DEPT-135
CDCl3
150MHz
150
140
130
120
110
100
90
80
70
60
50
40
30
20
10

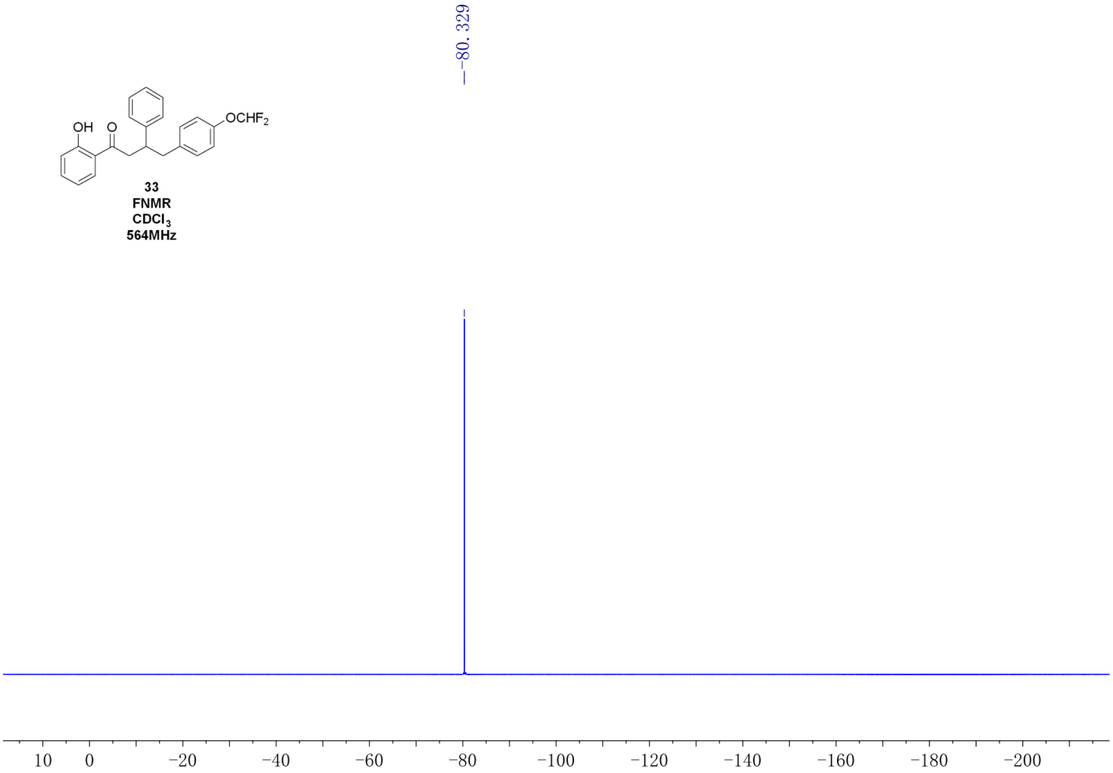
-80.329
OH
O
OCHF2
33
FNMR
CDCl3
564MHz
10
0
-20
-40
-60
-80
-100
-120
-140
-160
-180
-200

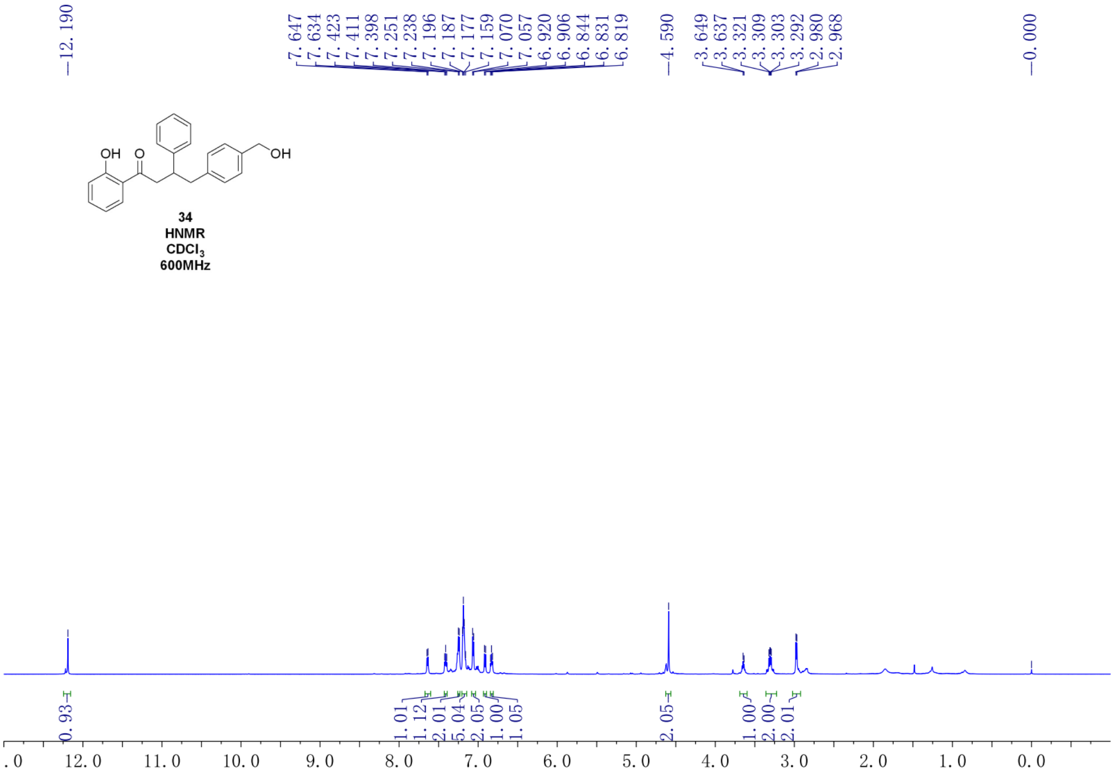
12.190
7.647
7.634
7.423
7.411
7.398
7.251
7.238
7.196
7.187
7.177
7.159
7.070
7.057
6.920
6.906
6.844
6.831
6.819
4.590
3.649
3.637
3.321
3.309
3.303
3.292
2.980
2.968
0.000
OH
O
OH
34
HNMR
CDCl3
600MHz
0.93
1.01
1.12
2.01
5.04
2.05
1.00
1.05
2.05
1.00
2.00
2.01
12.0
11.0
10.0
9.0
8.0
7.0
6.0
5.0
4.0
3.0
2.0
1.0
0.0

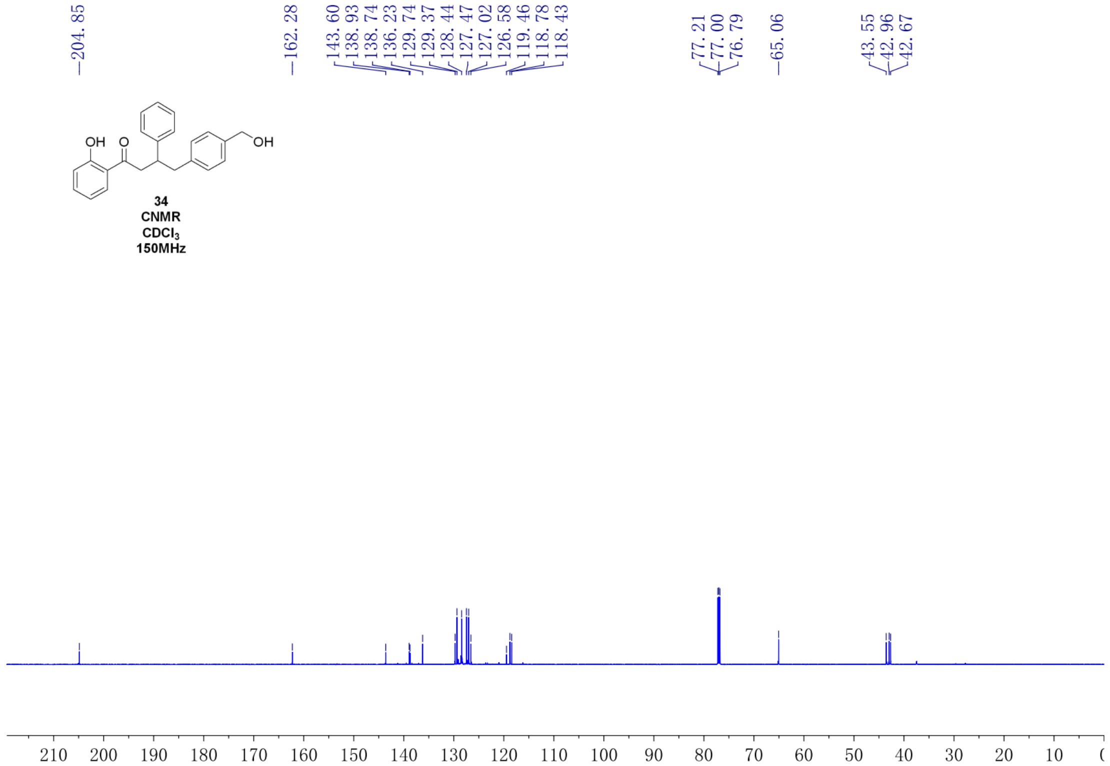
204.85
162.28
143.60
138.93
138.74
136.23
129.74
129.37
128.44
127.47
127.02
126.58
119.46
118.78
118.43
77.21
77.00
76.79
65.06
43.55
42.96
42.67
OH
O
OH
34
CNMR
CDCl3
150MHz
210
200
190
180
170
160
150
140
130
120
110
100
90
80
70
60
50
40
30
20
10

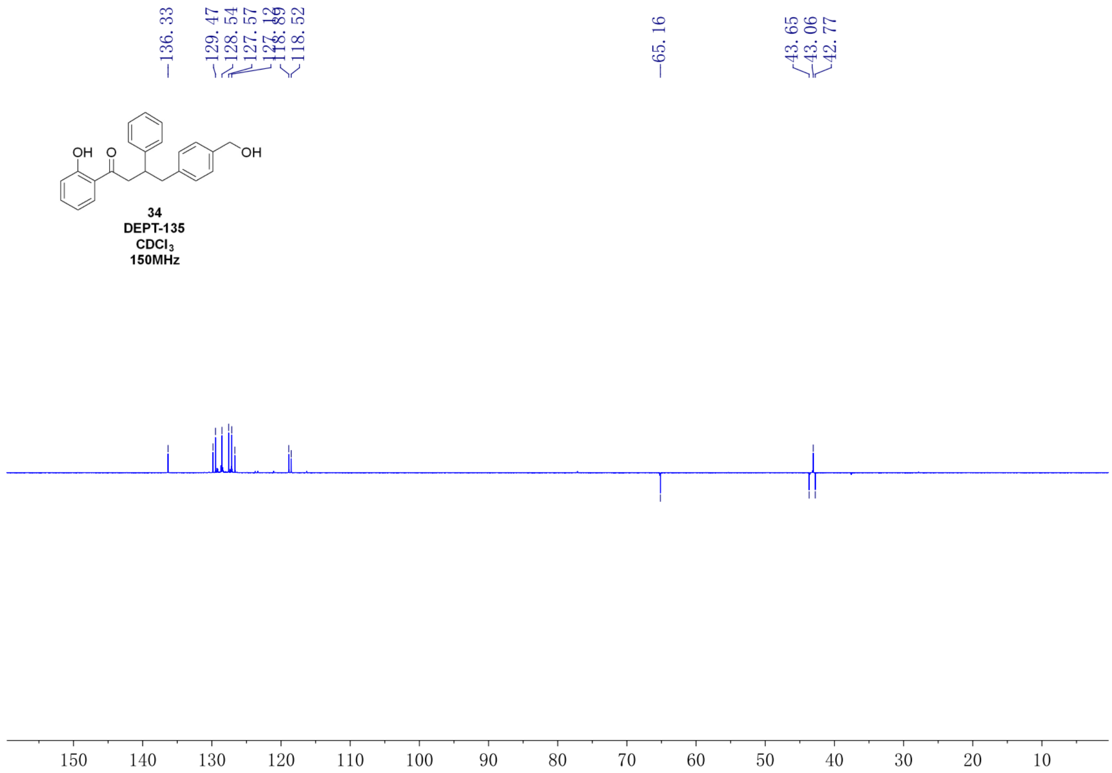
136.33
129.47
128.54
127.57
127.12
118.89
118.52
65.16
43.65
43.06
42.77
OH
O
OH
34
DEPT-135
$CDCl_3$
150MHz
150 140 130 120 110 100 90 80 70 60 50 40 30 20 10

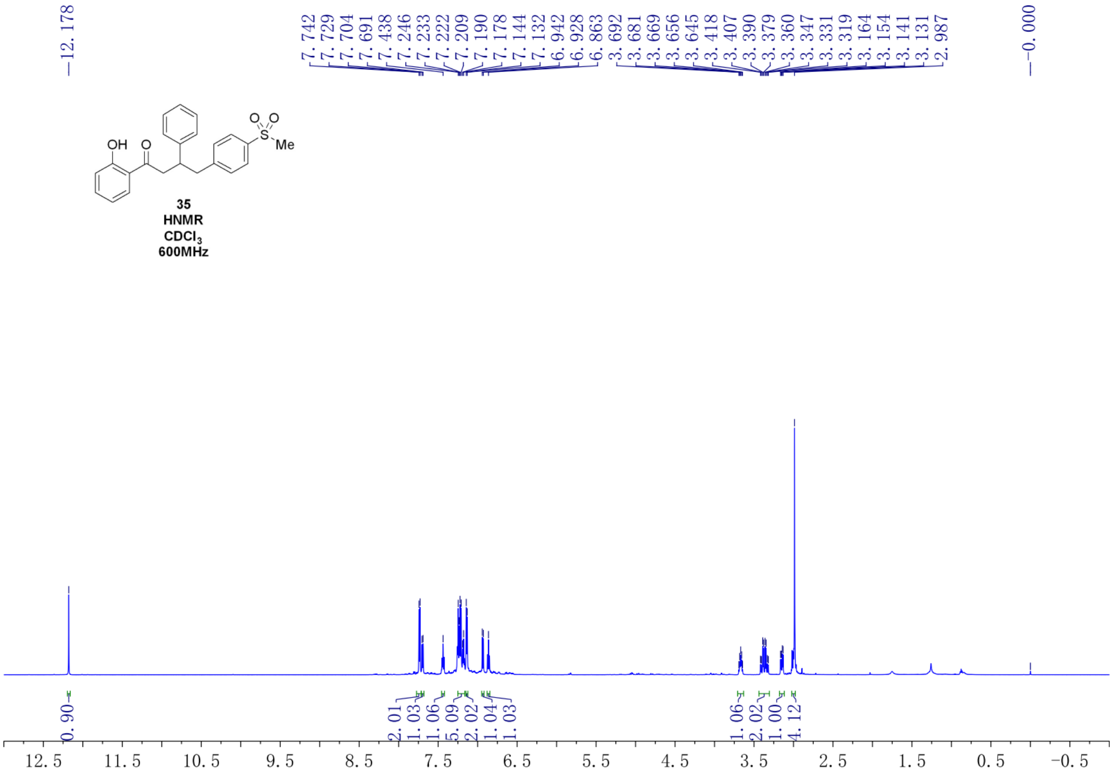
12.178
7.742
7.729
7.704
7.691
7.438
7.246
7.233
7.222
7.209
7.190
7.178
7.144
7.132
6.942
6.928
6.863
3.692
3.681
3.669
3.656
3.645
3.418
3.407
3.390
3.379
3.360
3.347
3.331
3.319
3.164
3.154
3.141
3.131
2.987
-0.000
OH
O
S
Me
35
HNMR
$CDCl_3$
600MHz
0.90
2.01
1.03
1.06
5.09
2.02
1.04
1.03
1.06
2.02
1.00
4.12
12.5 11.5 10.5 9.5 8.5 7.5 6.5 5.5 4.5 3.5 2.5 1.5 0.5 -0.5

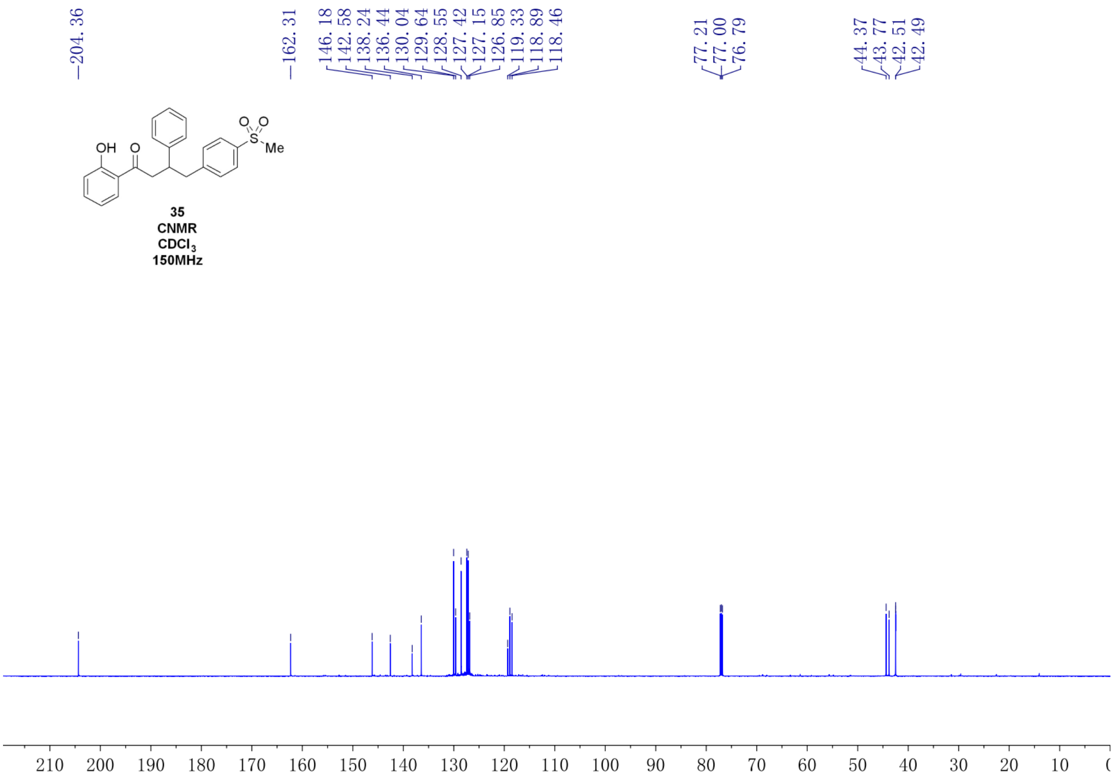
204.36
162.31
146.18
142.58
138.24
136.44
130.04
129.64
128.55
127.42
127.15
126.85
119.33
118.89
118.46
77.21
77.00
76.79
44.37
43.77
42.51
42.49
OH
O
O
O
S
Me
35
CNMR
CDCl3
150MHz
210 200 190 180 170 160 150 140 130 120 110 100 90 80 70 60 50 40 30 20 10

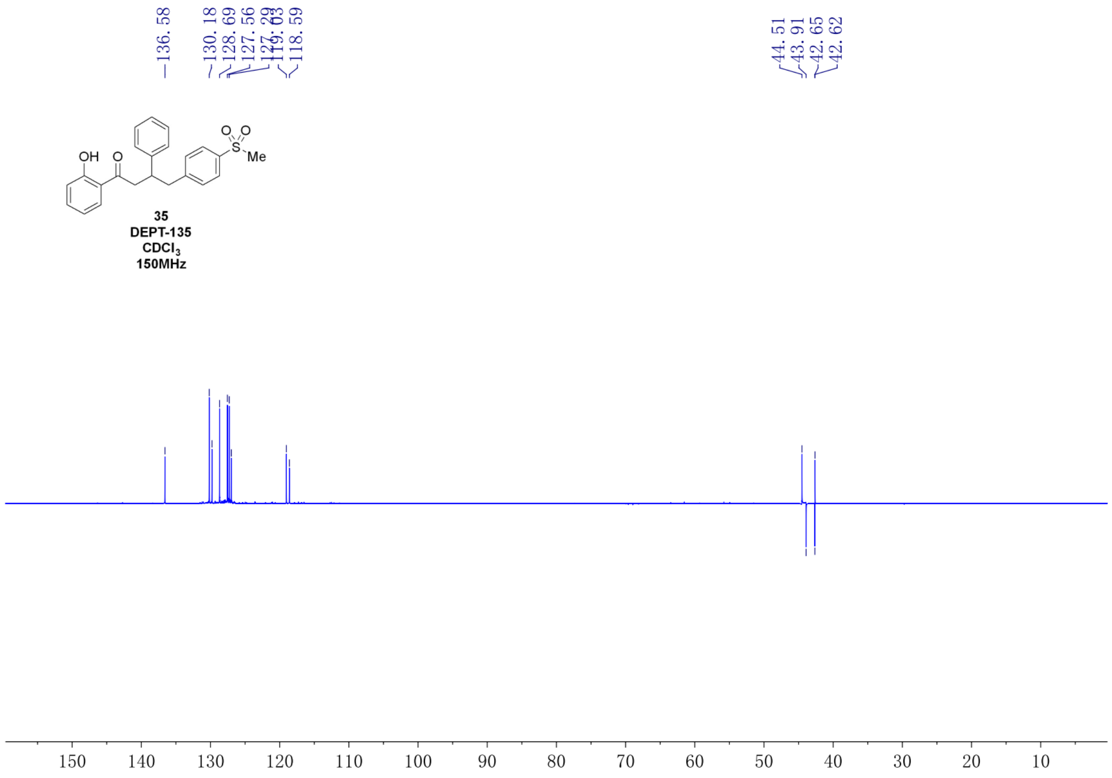
136.58
130.18
128.69
127.56
118.59
44.51
43.91
42.65
42.62
OH
O
O
O
S
Me
35
DEPT-135
CDCl3
150MHz
150 140 130 120 110 100 90 80 70 60 50 40 30 20 10

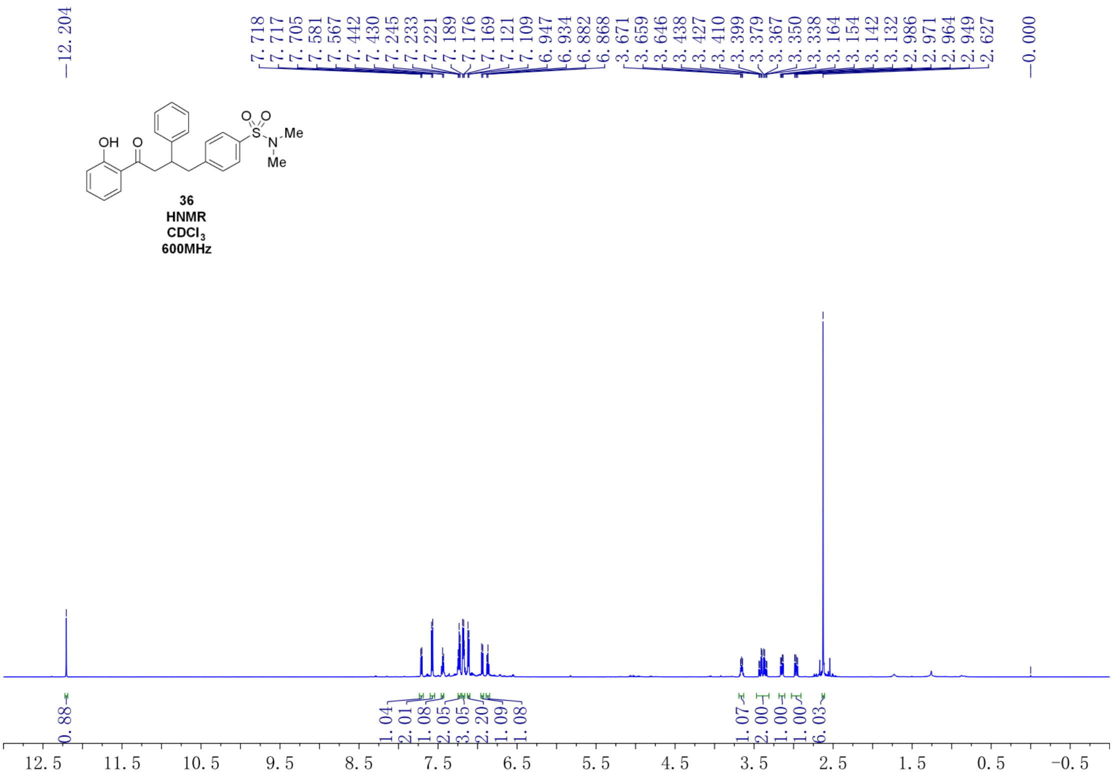
12.204
7.718
7.717
7.705
7.581
7.567
7.442
7.430
7.245
7.233
7.221
7.189
7.176
7.169
7.121
7.109
6.947
6.934
6.882
6.868
3.671
3.659
3.646
3.438
3.427
3.410
3.399
3.379
3.367
3.350
3.338
3.164
3.154
3.142
3.132
2.986
2.971
2.964
2.949
2.627
0.000
OH
O
O
O
S
N
Me
Me
36
HNMR
CDCl3
600MHz
0.88
1.04
2.01
1.08
2.05
3.05
2.20
1.09
1.08
1.07
2.00
1.00
1.00
6.03
12.5
11.5
10.5
9.5
8.5
7.5
6.5
5.5
4.5
3.5
2.5
1.5
0.5
-0.5

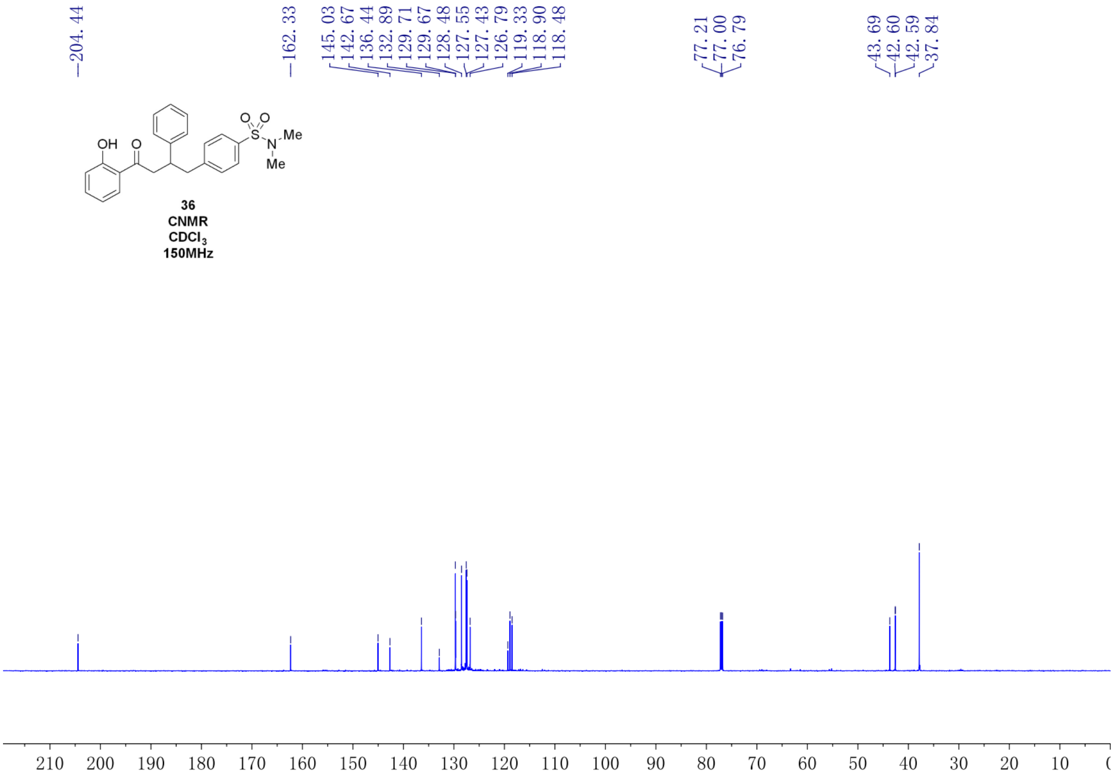
204.44
162.33
145.03
142.67
136.44
132.89
129.71
129.67
128.48
127.55
127.43
126.79
119.33
118.90
118.48
77.21
77.00
76.79
43.69
42.60
42.59
37.84
OH
O
O
O
S
N
Me
Me
36
CNMR
CDCl3
150MHz
210
200
190
180
170
160
150
140
130
120
110
100
90
80
70
60
50
40
30
20
10

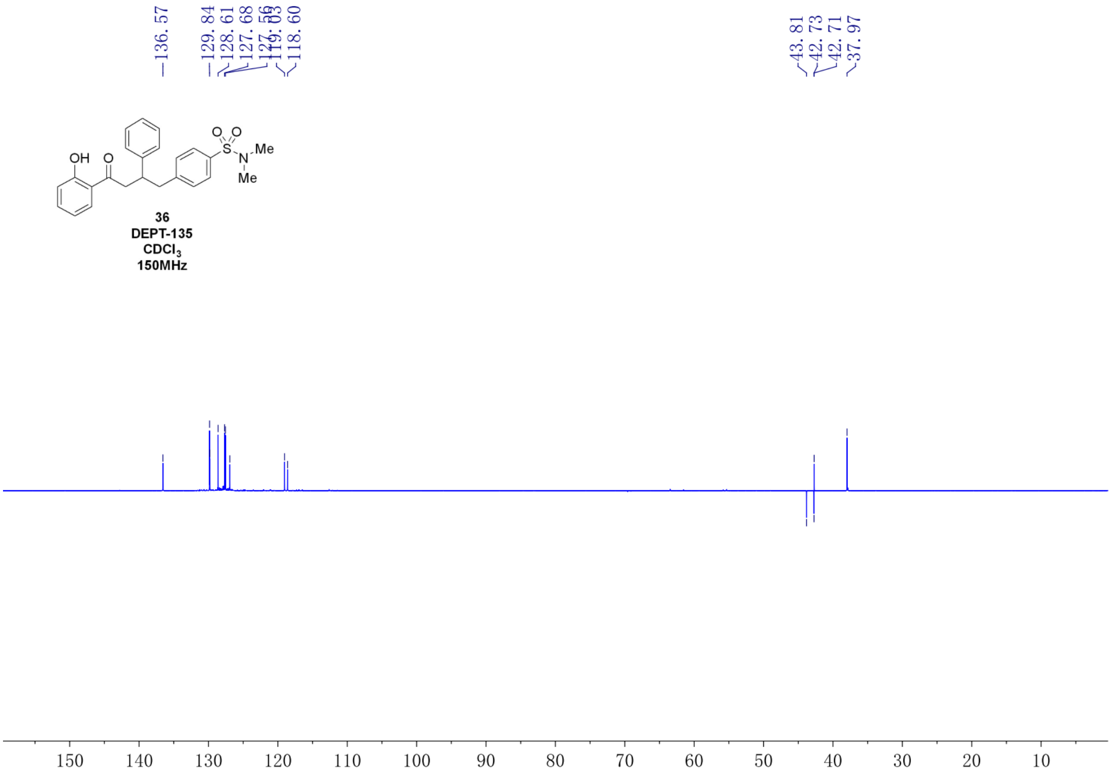

36
DEPT-135
$CDCl_3$
150MHz

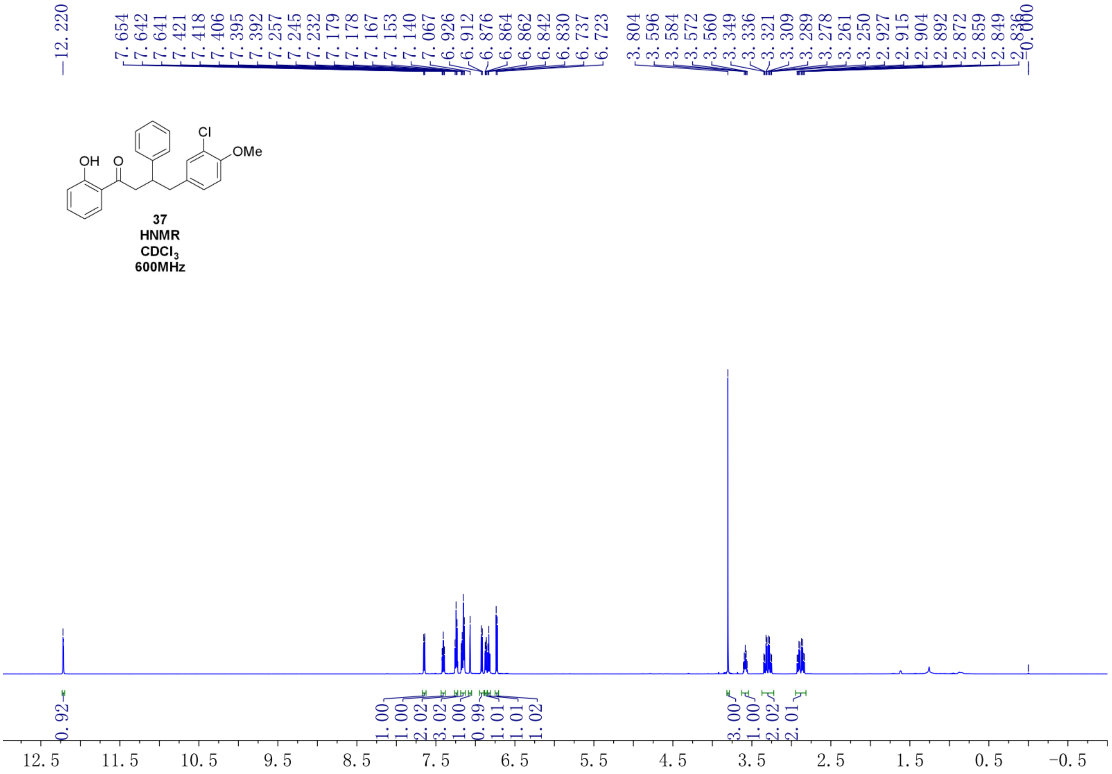

37
HNMR
$CDCl_3$
600MHz

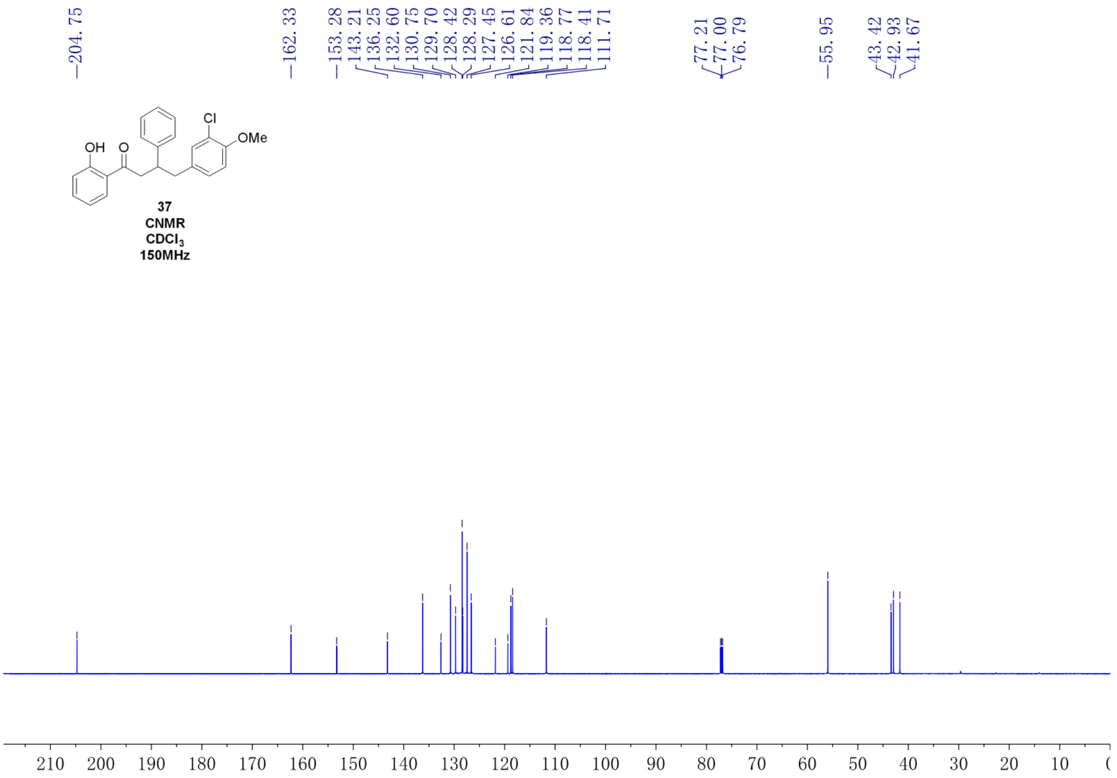
204.75
162.33
153.28
143.21
136.25
132.60
130.75
129.70
128.42
128.29
127.45
126.61
121.84
119.36
118.77
118.41
111.71
77.21
77.00
76.79
55.95
43.42
42.93
41.67
OH
O
Cl
OMe
37
CNMR
CDCl3
150MHz
210 200 190 180 170 160 150 140 130 120 110 100 90 80 70 60 50 40 30 20 10

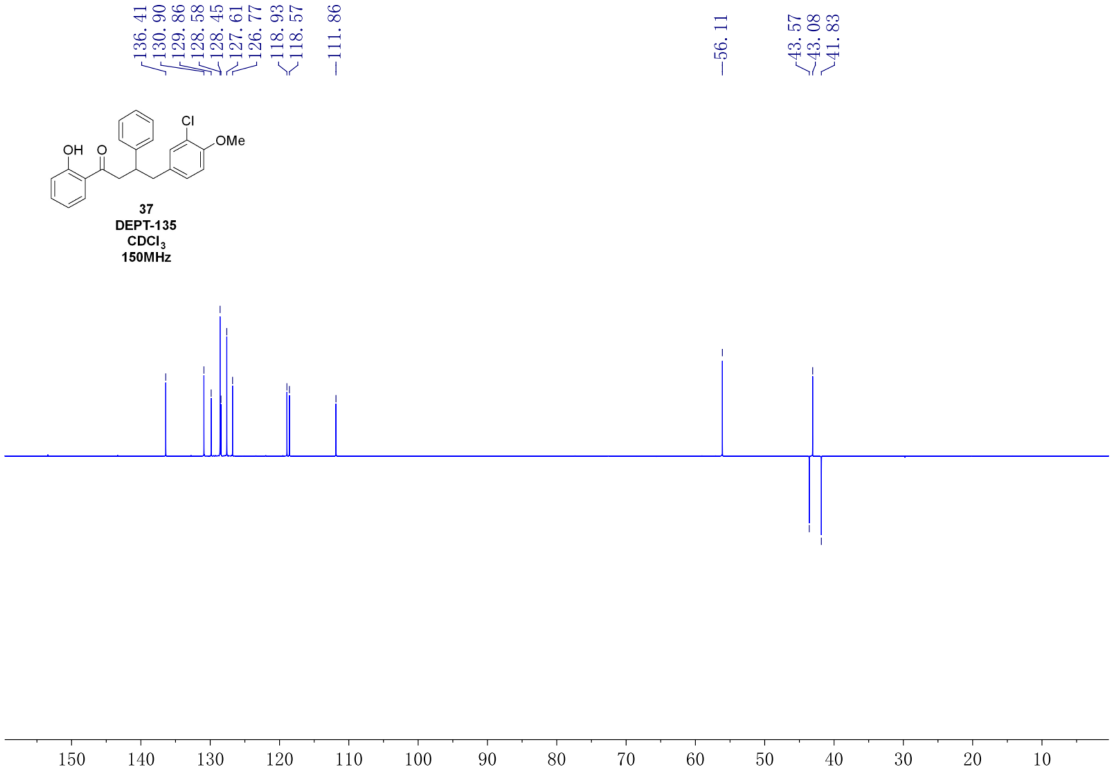
136.41
130.90
129.86
128.58
128.45
127.61
126.77
118.93
118.57
111.86
56.11
43.57
43.08
41.83
OH
O
Cl
OMe
37
DEPT-135
CDCl3
150MHz
150 140 130 120 110 100 90 80 70 60 50 40 30 20 10

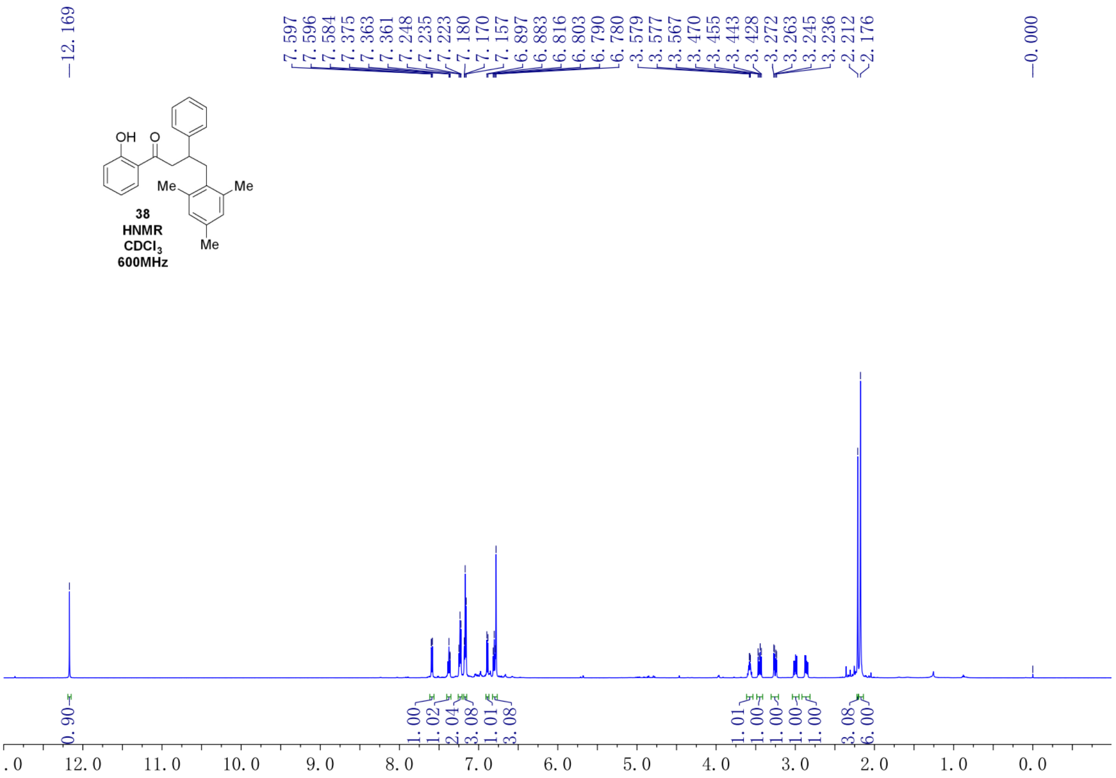
12.169
7.597 7.596 7.584 7.375 7.363 7.361 7.248 7.235 7.223 7.180 7.170 7.157 6.897 6.883 6.816 6.803 6.790 6.780
3.579 3.577 3.567 3.470 3.455 3.443 3.428 3.272 3.263 3.245 3.236 2.212 2.176
0.000
OH O
Me Me
Me
38
HNMR
CDCl3
600MHz
0.90 1.00 1.02 2.04 3.08 1.01 3.08 1.01 1.00 1.00 1.00 1.00 3.08 6.00
12.0 11.0 10.0 9.0 8.0 7.0 6.0 5.0 4.0 3.0 2.0 1.0 0.0

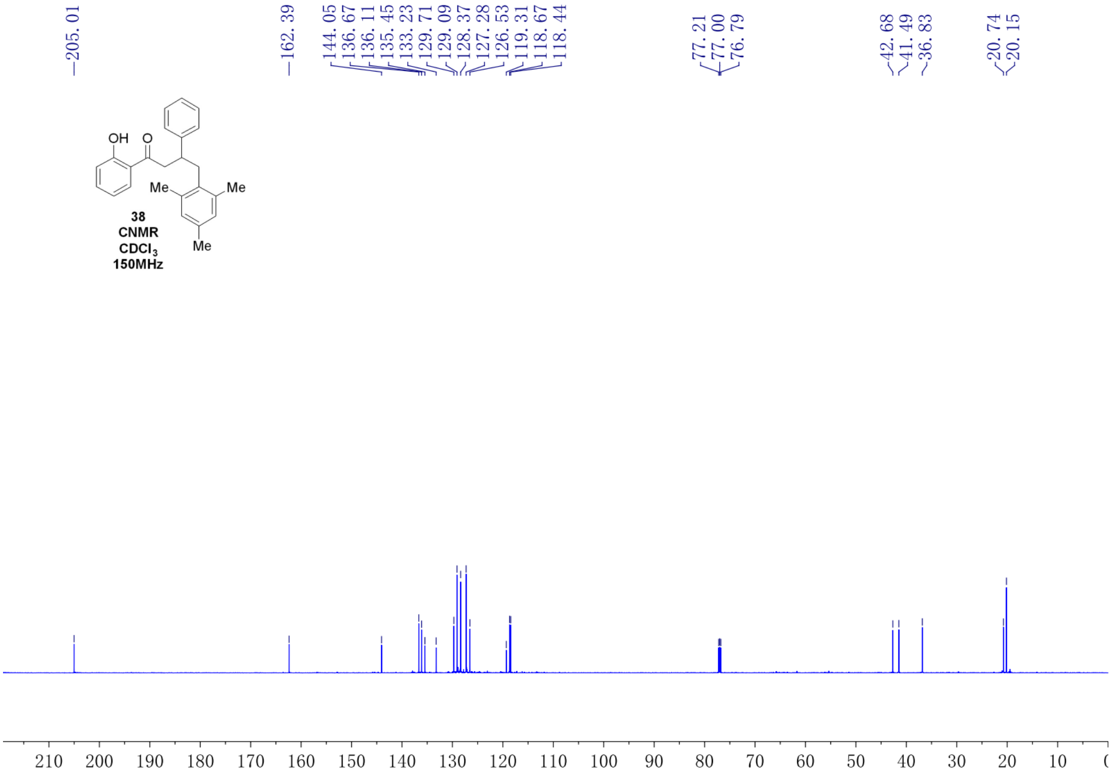
205.01
162.39
144.05 136.67 136.11 135.45 133.23 129.71 129.09 128.37 127.28 126.53 119.31 118.67 118.44
77.21 77.00 76.79
42.68 41.49 36.83
20.74 20.15
OH O
Me Me
Me
38
CNMR
CDCl3
150MHz
210 200 190 180 170 160 150 140 130 120 110 100 90 80 70 60 50 40 30 20 10

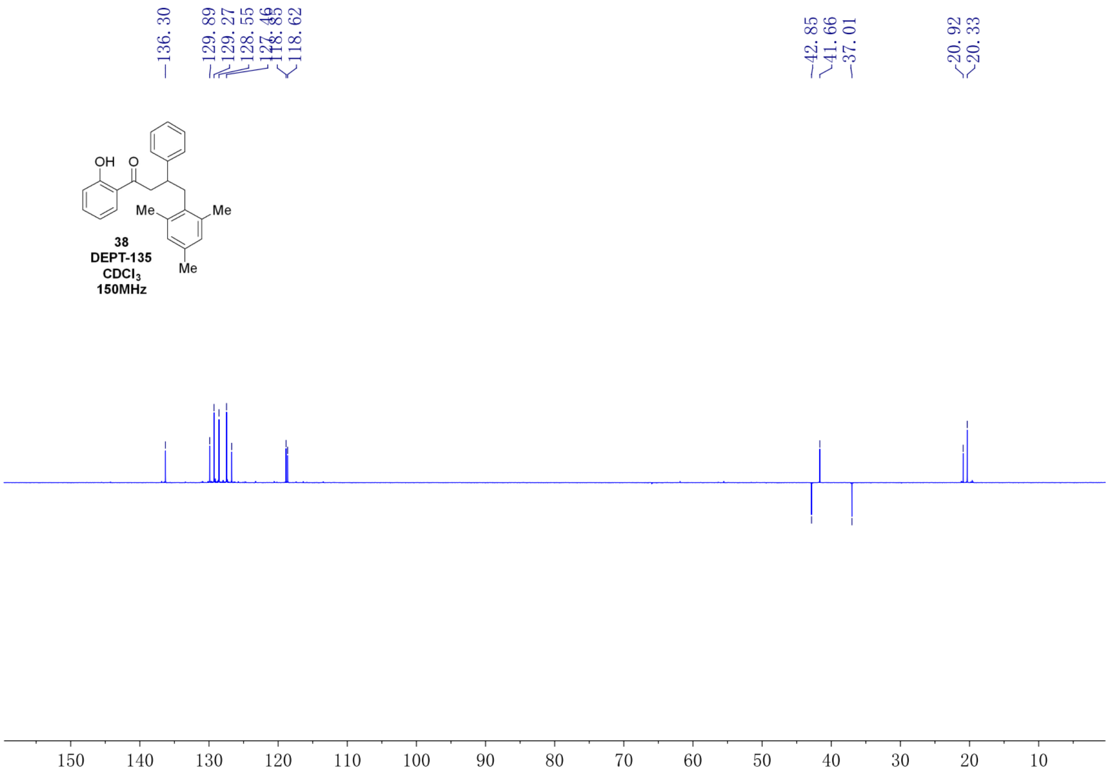

38
DEPT-135
$CDCl_3$
150MHz

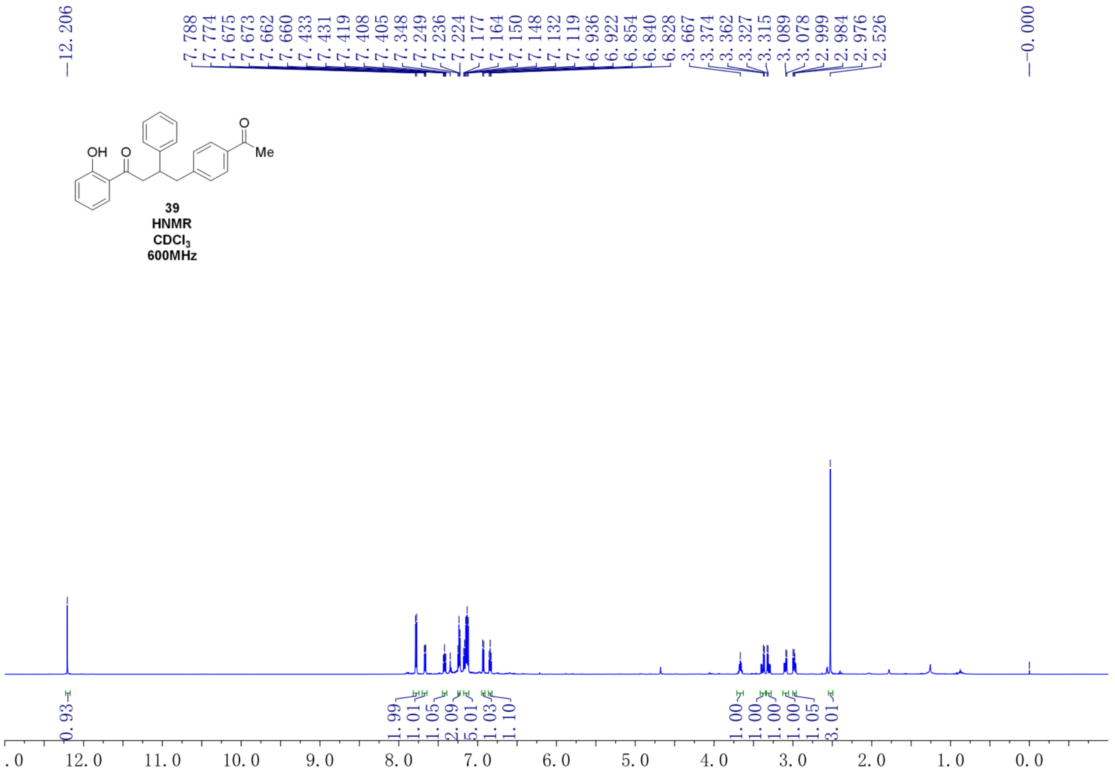

39
HNMR
$CDCl_3$
600MHz

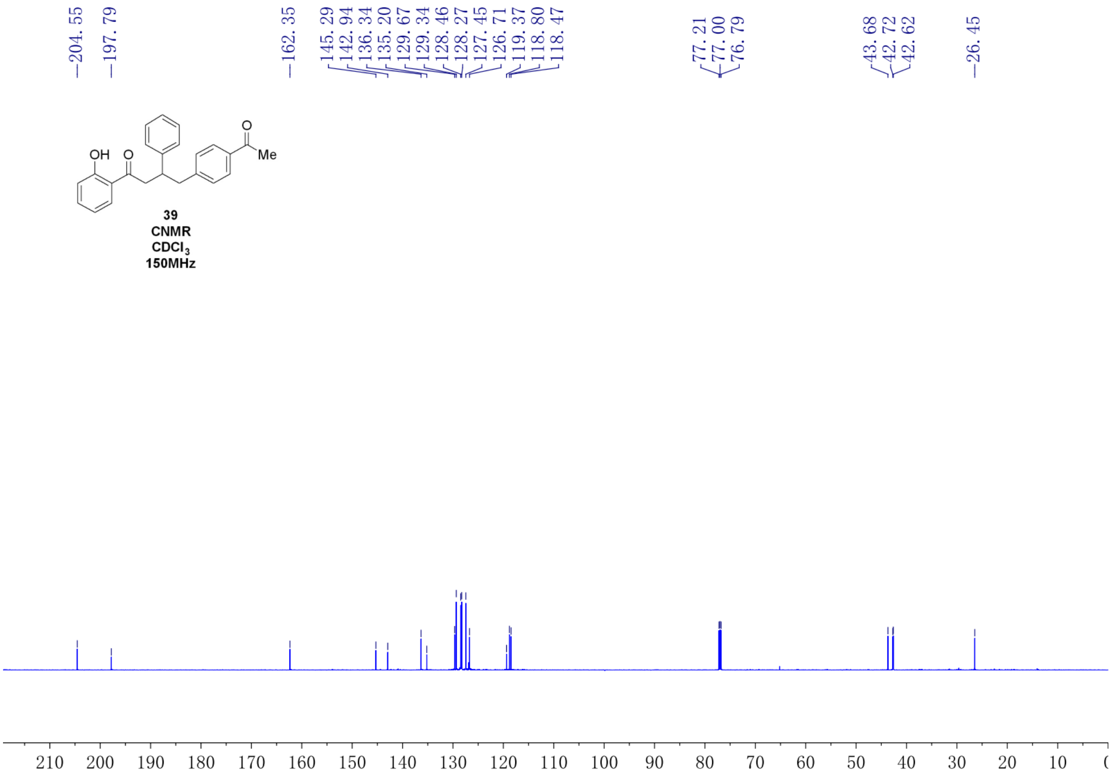
204.55
197.79
162.35
145.29
142.94
136.34
135.20
129.67
129.34
128.46
128.27
127.45
126.71
119.37
118.80
118.47
77.21
77.00
76.79
43.68
42.72
42.62
26.45
OH
O
O
Me
39
CNMR
$CDCl_3$
150MHz
210 200 190 180 170 160 150 140 130 120 110 100 90 80 70 60 50 40 30 20 10

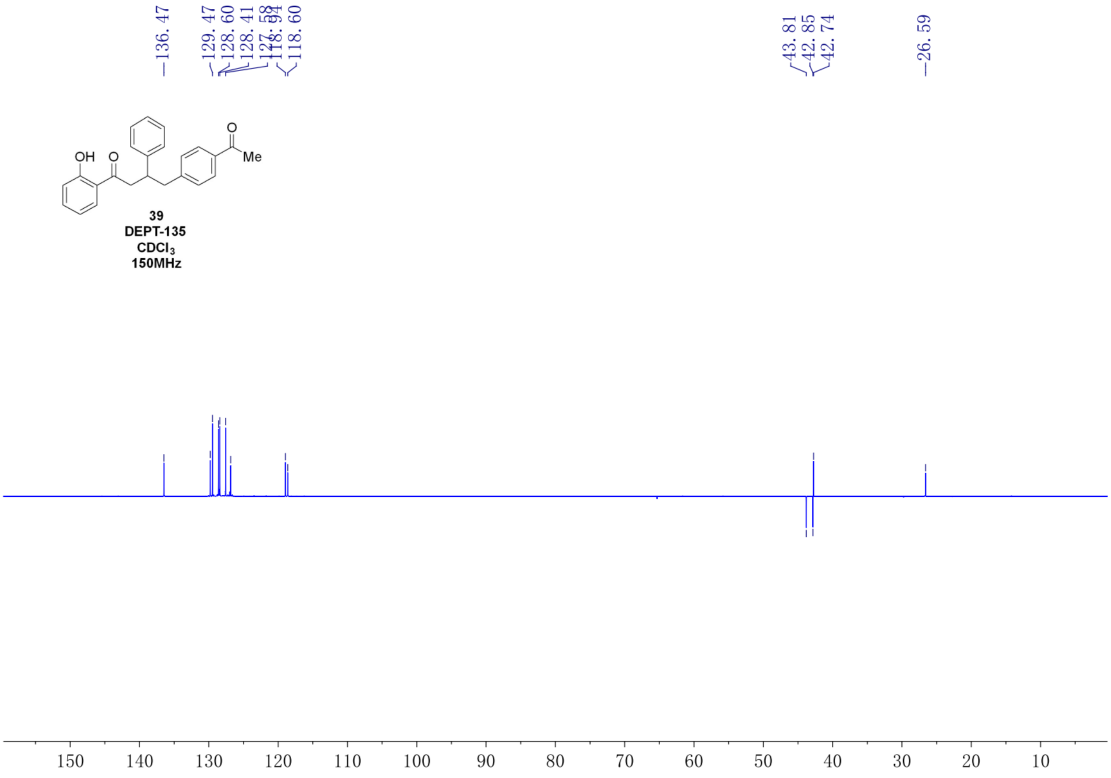
136.47
129.47
128.60
128.41
127.58
118.94
118.60
43.81
42.85
42.74
26.59
OH
O
O
Me
39
DEPT-135
$CDCl_3$
150MHz
150 140 130 120 110 100 90 80 70 60 50 40 30 20 10

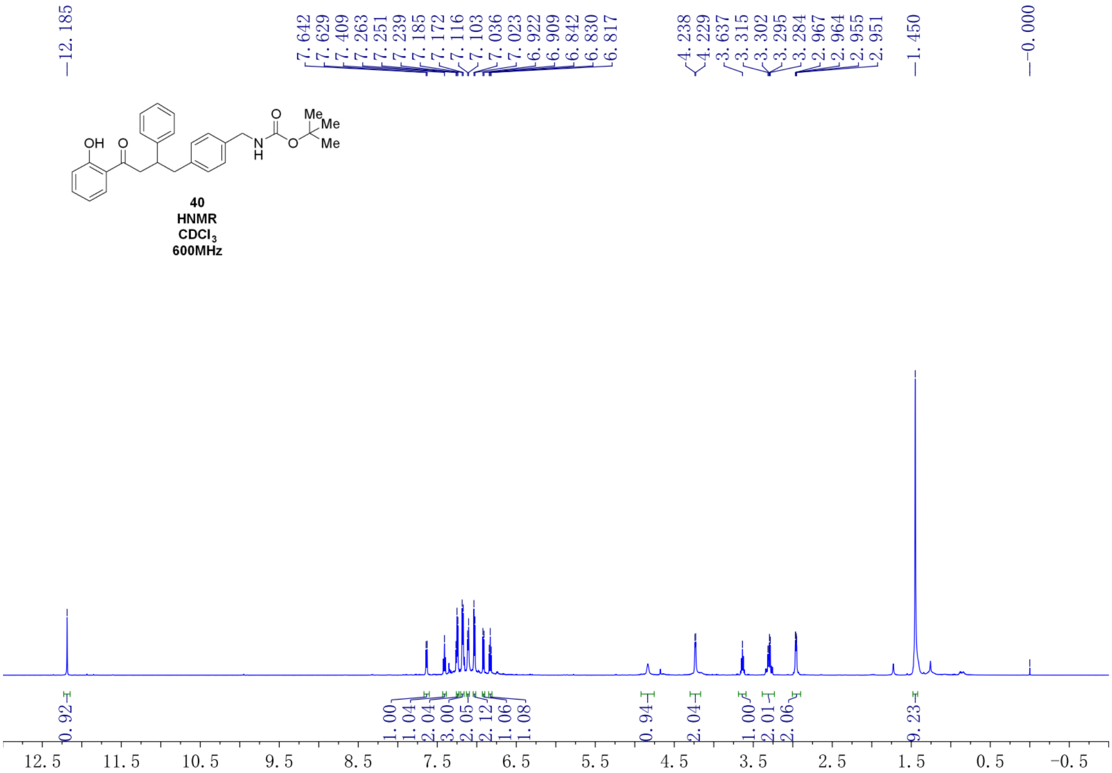
12.185
7.642
7.629
7.409
7.263
7.251
7.239
7.185
7.172
7.116
7.103
7.036
7.023
6.922
6.909
6.842
6.830
6.817
4.238
4.229
3.637
3.315
3.302
3.295
3.284
2.967
2.964
2.955
2.951
1.450
0.000
OH
O
O
Me
Me
Me
N
H
O
40
HNMR
$CDCl_3$
600MHz
0.92
1.00
1.04
2.04
3.00
2.05
2.12
1.06
1.08
0.94
2.04
1.00
2.01
2.06
9.23
12.5
11.5
10.5
9.5
8.5
7.5
6.5
5.5
4.5
3.5
2.5
1.5
0.5
-0.5

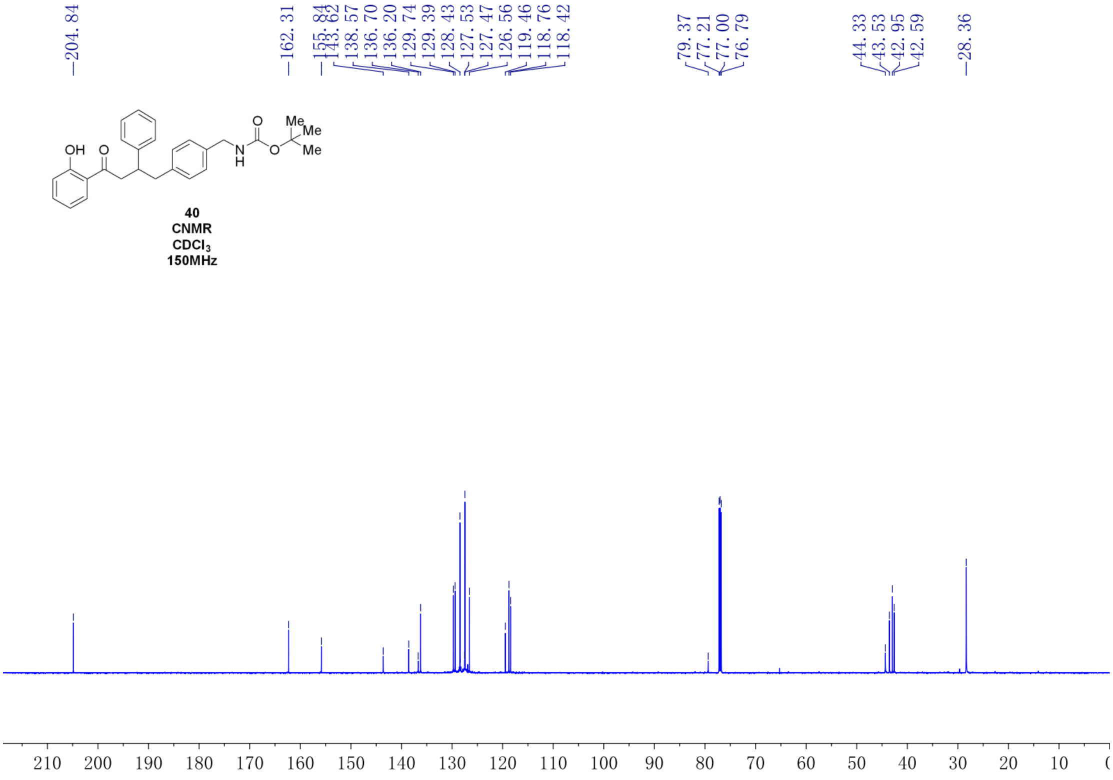
204.84
162.31
155.84
143.62
138.57
136.70
136.20
129.74
129.39
128.43
127.53
127.47
126.56
119.46
118.76
118.42
79.37
77.21
77.00
76.79
44.33
43.53
42.95
42.59
28.36
OH
O
O
Me
Me
Me
N
H
O
40
CNMR
$CDCl_3$
150MHz
210
200
190
180
170
160
150
140
130
120
110
100
90
80
70
60
50
40
30
20
10

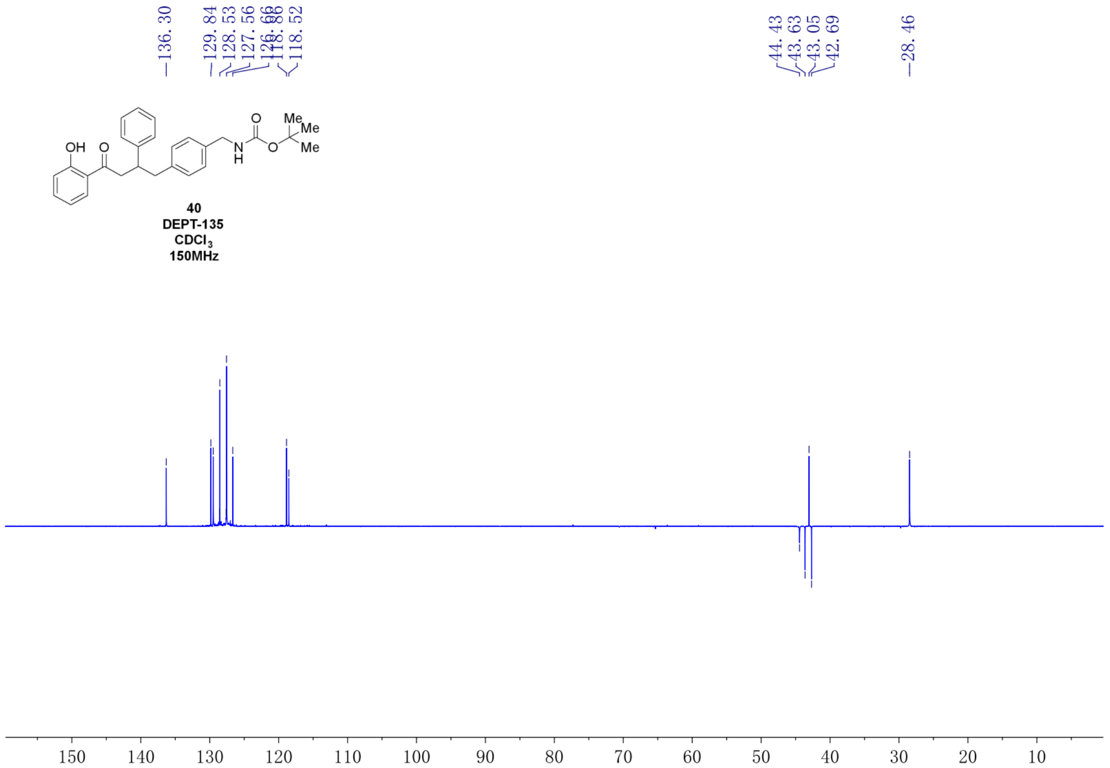
136.30
129.84
128.53
127.56
126.66
118.86
118.52
44.43
43.63
43.05
42.69
28.46
OH
O
Me
Me
Me
O
N
H
O
40
DEPT-135
CDCl3
150MHz
150
140
130
120
110
100
90
80
70
60
50
40
30
20
10

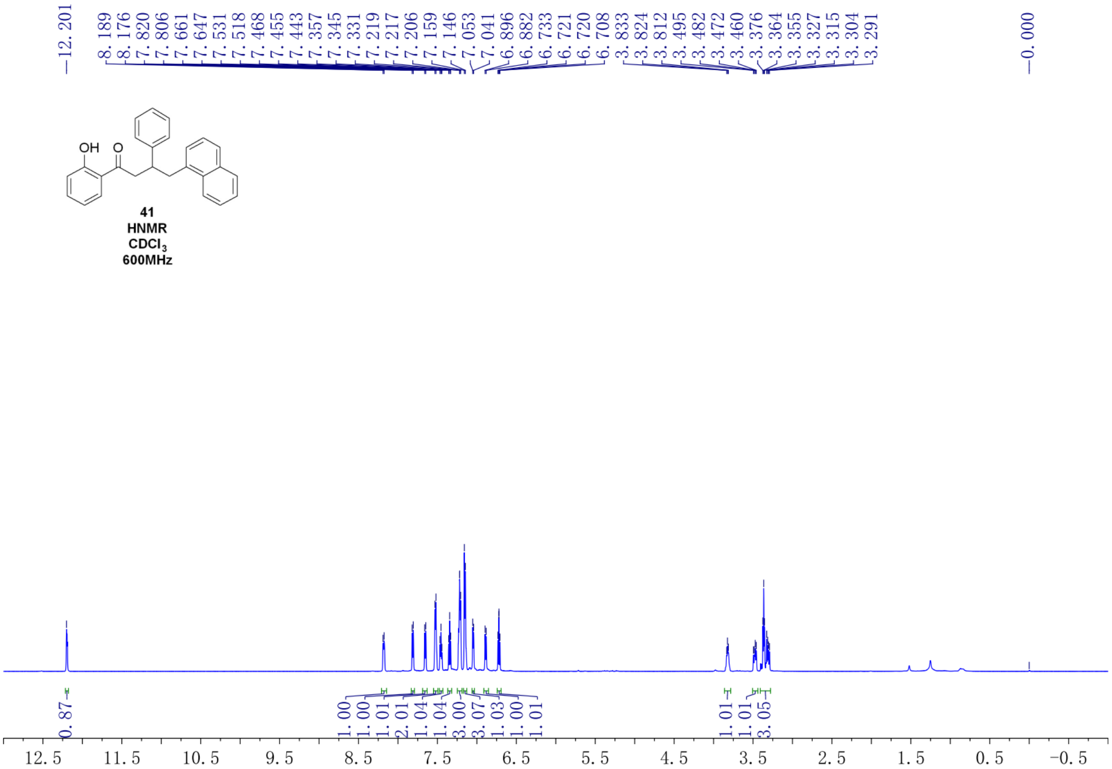
12.201
8.189
8.176
7.820
7.806
7.661
7.647
7.531
7.518
7.468
7.455
7.443
7.357
7.345
7.331
7.219
7.217
7.206
7.159
7.146
7.053
7.041
6.896
6.882
6.733
6.721
6.720
6.708
3.833
3.824
3.812
3.495
3.482
3.472
3.460
3.376
3.364
3.355
3.327
3.315
3.304
3.291
0.000
OH
O
41
HNMR
CDCl3
600MHz
0.87
1.00
1.00
1.01
2.01
1.04
1.04
3.00
3.07
1.03
1.00
1.01
1.01
1.01
3.05
12.5
11.5
10.5
9.5
8.5
7.5
6.5
5.5
4.5
3.5
2.5
1.5
0.5
-0.5

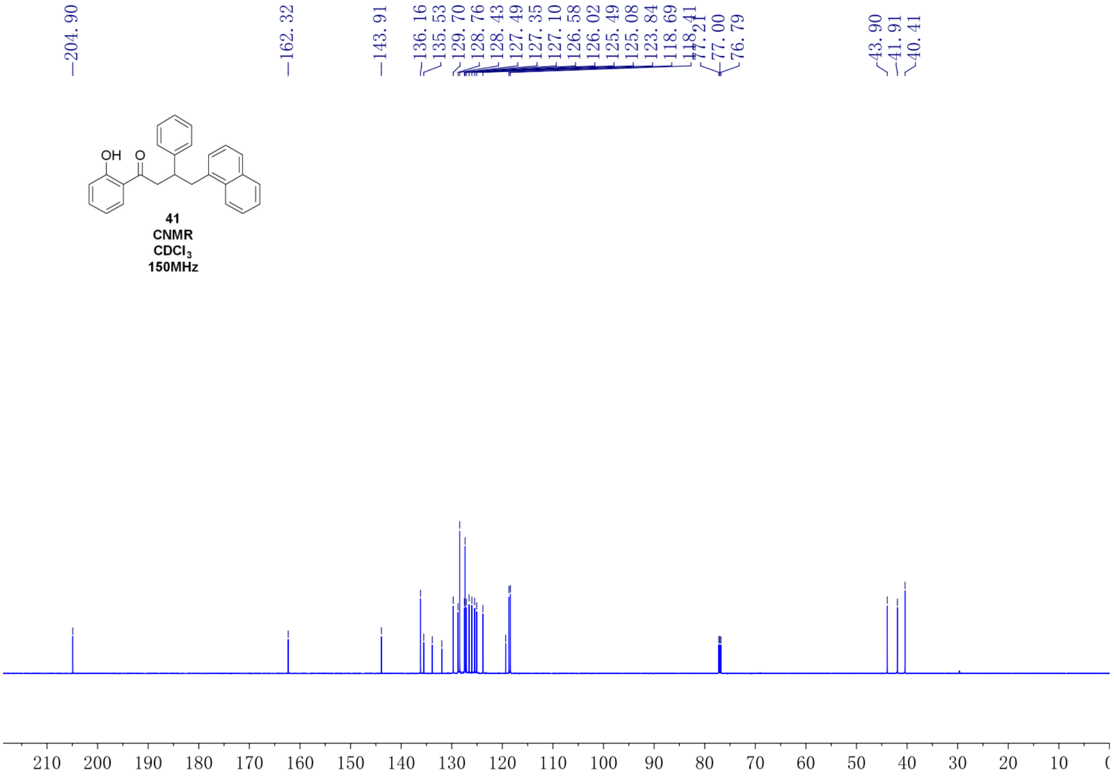
204.90
162.32
143.91
136.16
135.53
129.70
128.76
128.43
127.49
127.35
127.10
126.58
126.02
125.49
125.08
123.84
118.69
118.41
77.21
77.00
76.79
43.90
41.91
40.41
OH
O
41
CNMR
CDCl3
150MHz
210 200 190 180 170 160 150 140 130 120 110 100 90 80 70 60 50 40 30 20 10

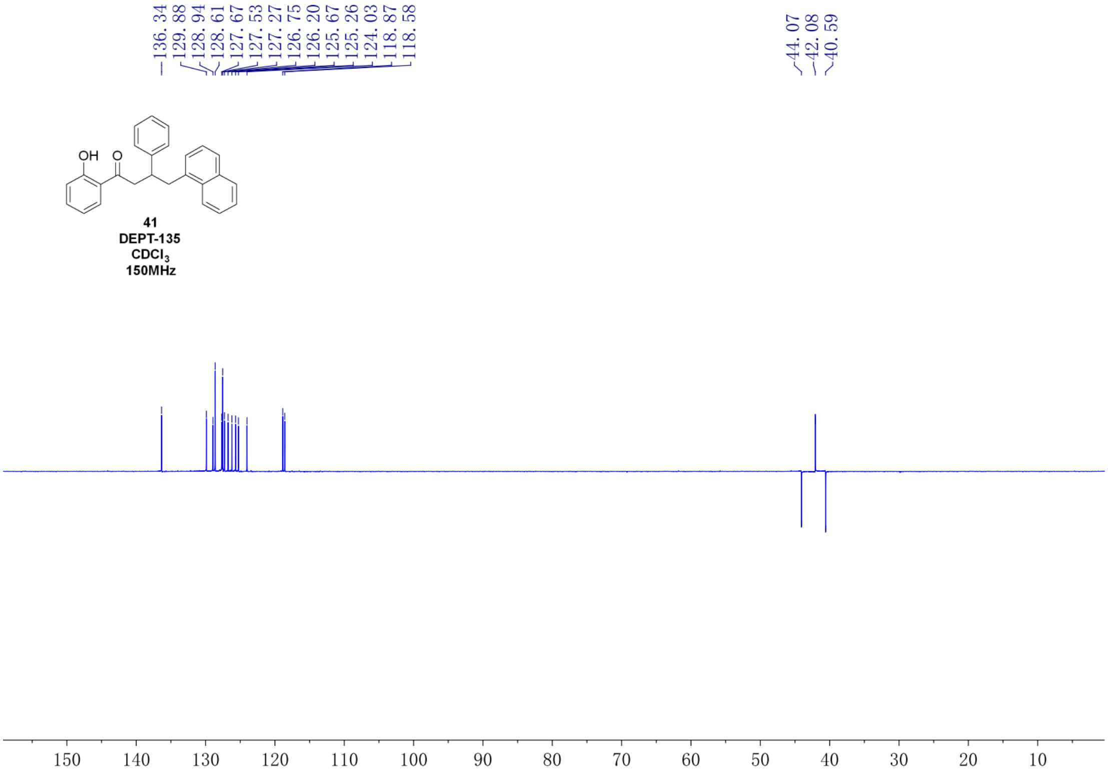
136.34
129.88
128.94
128.61
127.67
127.53
127.27
126.75
126.20
125.67
125.26
124.03
118.87
118.58
44.07
42.08
40.59
OH
O
41
DEPT-135
CDCl3
150MHz
150 140 130 120 110 100 90 80 70 60 50 40 30 20 10

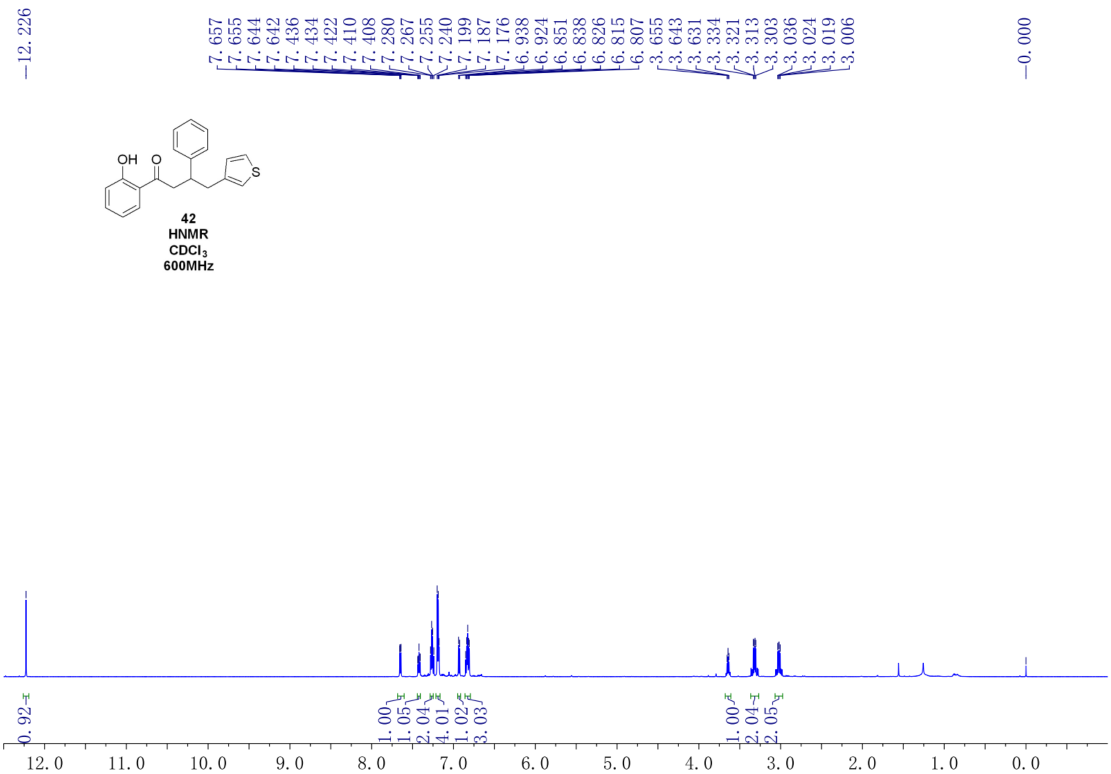
12.226
7.657 7.655 7.644 7.642 7.436 7.434 7.422 7.410 7.408 7.280 7.267 7.255 7.240 7.199 7.187 7.176 6.938 6.924 6.851 6.838 6.826 6.815 6.807 3.655 3.643 3.631 3.334 3.321 3.313 3.303 3.036 3.024 3.019 3.006
0.000
42
HNMR
CDCl3
600MHz
0.92 1.00 1.05 2.04 4.01 1.02 3.03 1.00 2.04 2.05
12.0 11.0 10.0 9.0 8.0 7.0 6.0 5.0 4.0 3.0 2.0 1.0 0.0

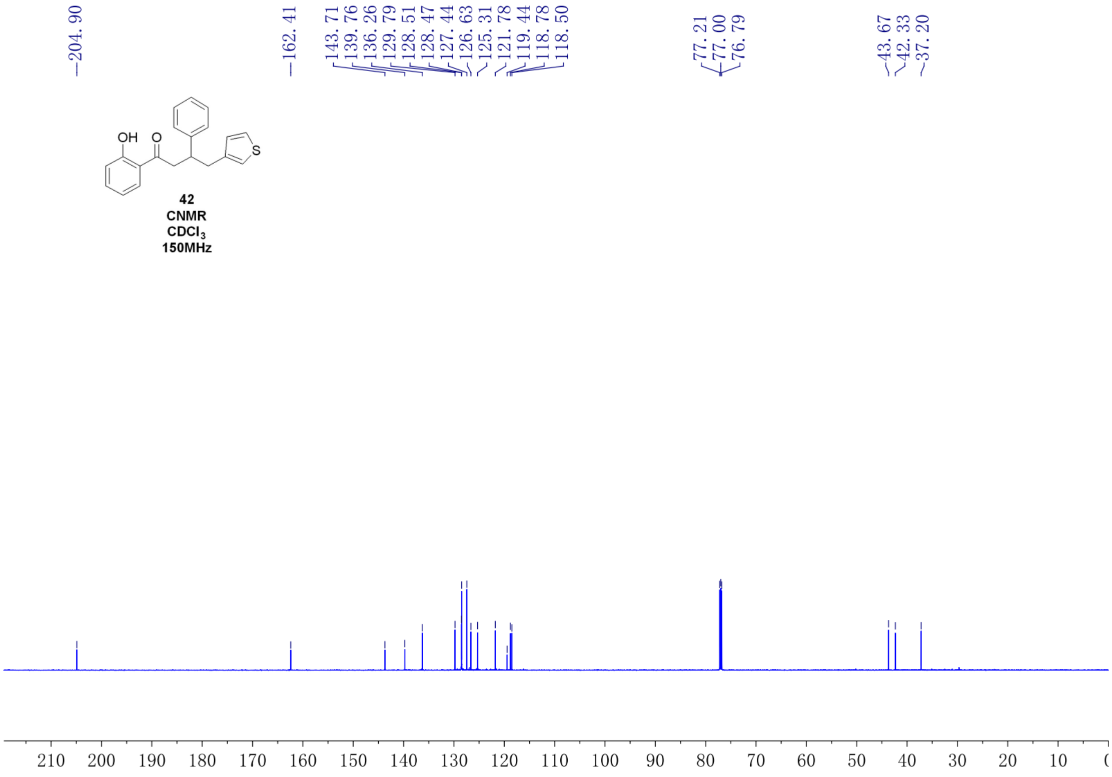
204.90
162.41
143.71 139.76 136.26 129.79 128.51 128.47 127.44 126.63 125.31 121.78 119.44 118.78 118.50
77.21 77.00 76.79
43.67 42.33 37.20
42
CNMR
CDCl3
150MHz
210 200 190 180 170 160 150 140 130 120 110 100 90 80 70 60 50 40 30 20 10

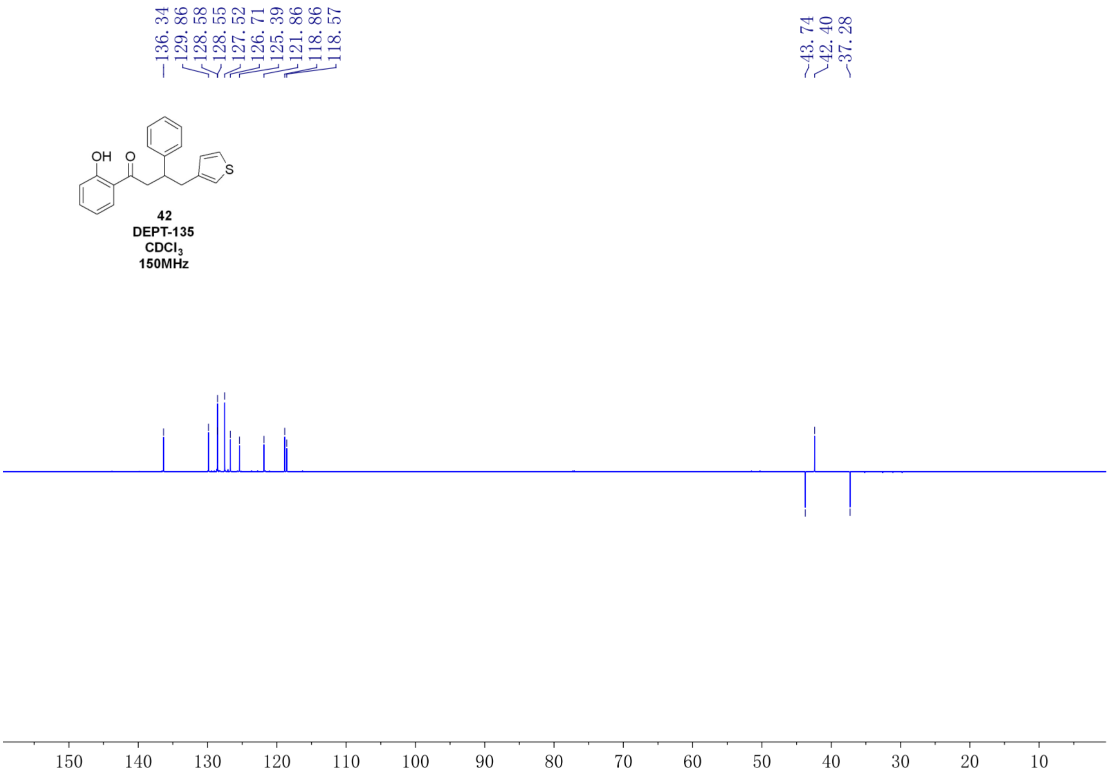
136.34
129.86
128.58
128.55
127.52
126.71
125.39
121.86
118.86
118.57
43.74
42.40
37.28
OH O
S
42
DEPT-135
$CDCl_3$
150MHz
150 140 130 120 110 100 90 80 70 60 50 40 30 20 10

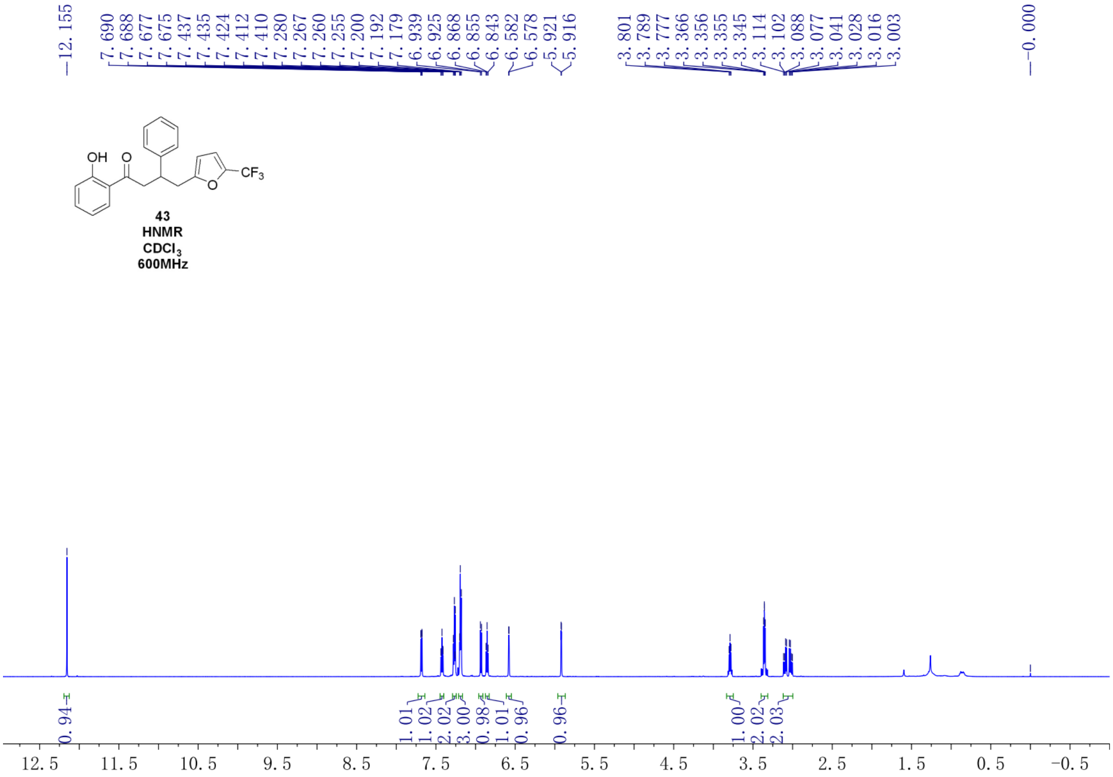
12.155
7.690
7.688
7.677
7.675
7.437
7.435
7.424
7.412
7.410
7.280
7.267
7.260
7.255
7.200
7.192
7.179
6.939
6.925
6.868
6.855
6.843
6.582
6.578
5.921
5.916
3.801
3.789
3.777
3.366
3.356
3.355
3.345
3.114
3.102
3.088
3.077
3.041
3.028
3.016
3.003
0.000
OH O
CF_3
O
43
HNMR
$CDCl_3$
600MHz
0.94
1.01
1.02
2.02
3.00
0.98
1.01
0.96
0.96
1.00
2.02
2.03
12.5 11.5 10.5 9.5 8.5 7.5 6.5 5.5 4.5 3.5 2.5 1.5 0.5 -0.5

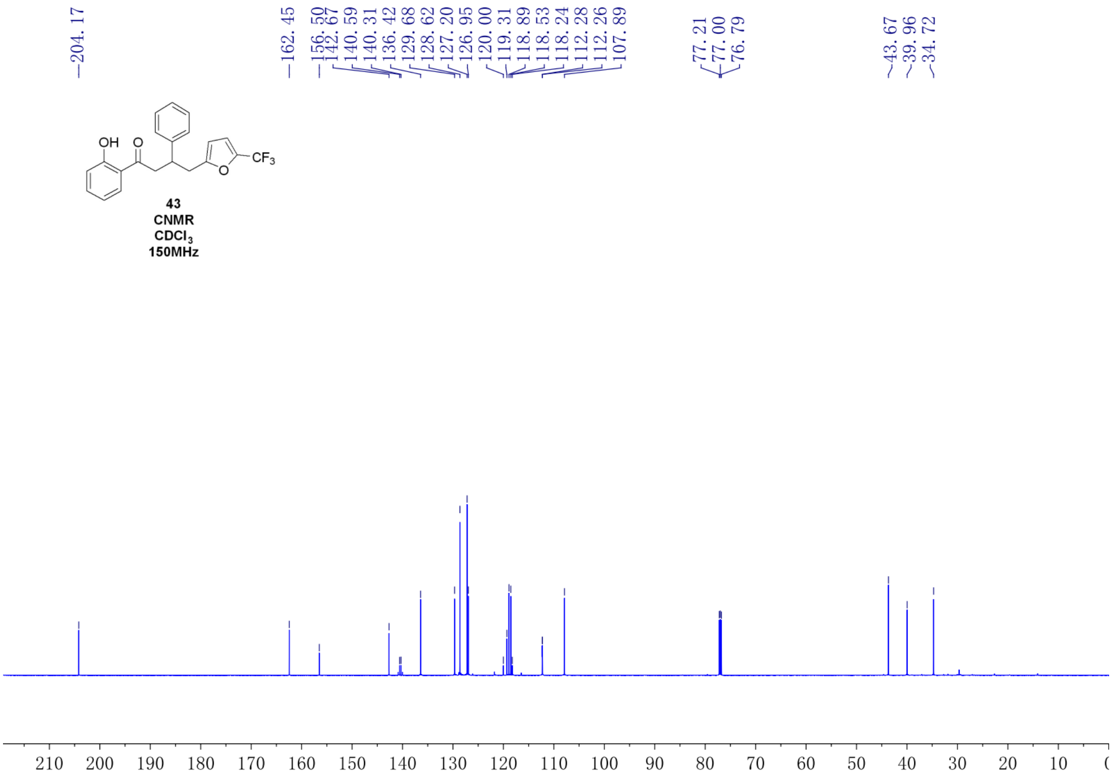
204.17
162.45
156.50
142.67
140.59
140.31
136.42
129.68
128.62
127.20
126.95
120.00
119.31
118.89
118.53
118.24
112.28
112.26
107.89
77.21
77.00
76.79
43.67
39.96
34.72
OH
O
CF3
O
43
CNMR
CDCl3
150MHz
210 200 190 180 170 160 150 140 130 120 110 100 90 80 70 60 50 40 30 20 10

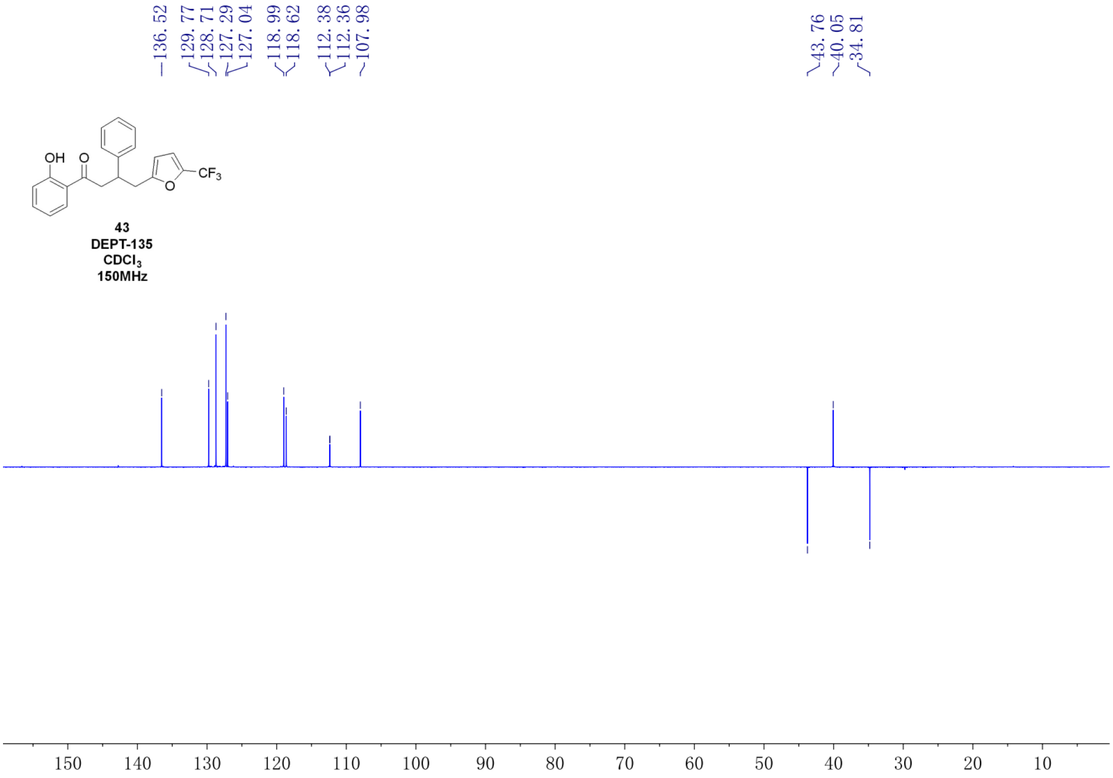
136.52
129.77
128.71
127.29
127.04
118.99
118.62
112.38
112.36
107.98
43.76
40.05
34.81
OH
O
CF3
O
43
DEPT-135
CDCl3
150MHz
150 140 130 120 110 100 90 80 70 60 50 40 30 20 10

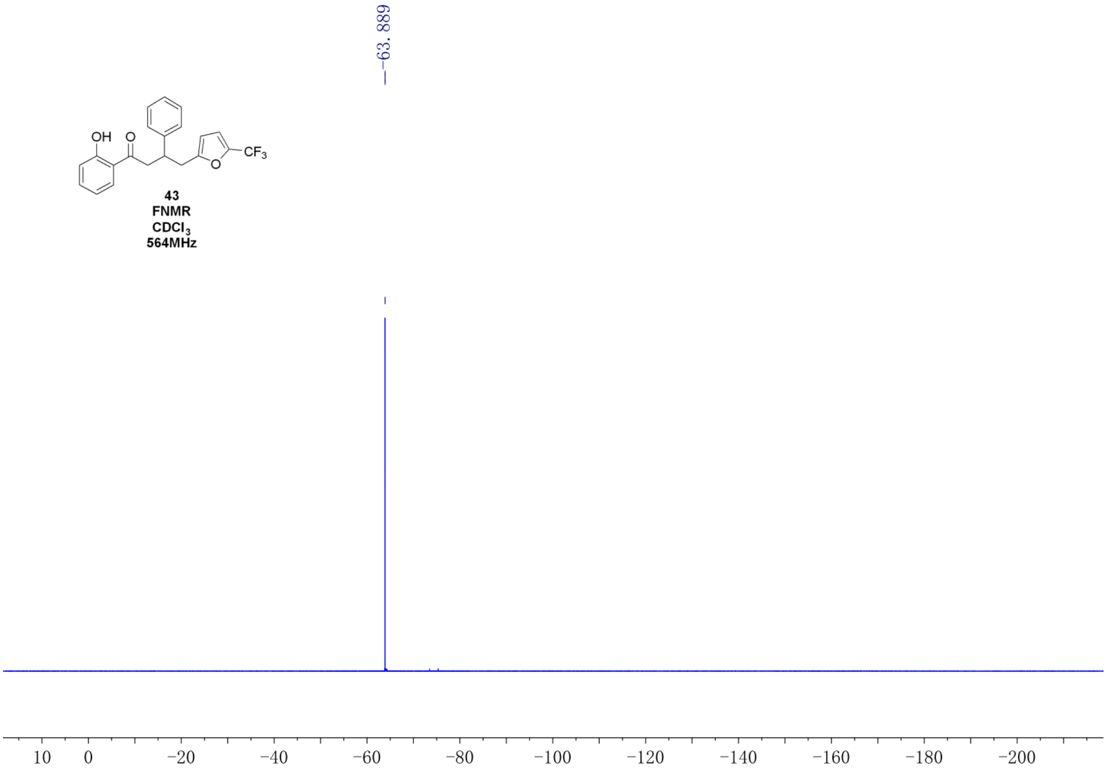
-63.889
OH
O
O
CF3
43
FNMR
CDCl3
564MHz
10 0 -20 -40 -60 -80 -100 -120 -140 -160 -180 -200

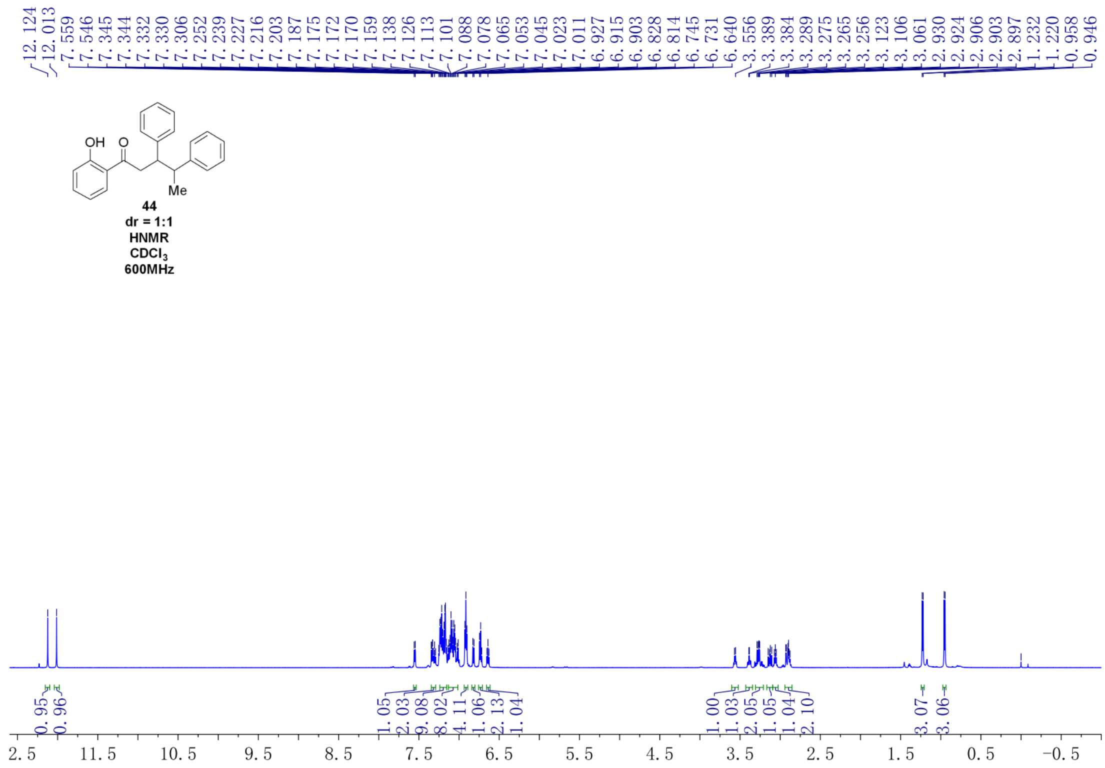
12.124
12.013
7.559
7.546
7.345
7.344
7.332
7.330
7.306
7.252
7.239
7.227
7.216
7.203
7.187
7.175
7.172
7.170
7.159
7.138
7.126
7.113
7.101
7.088
7.078
7.065
7.053
7.045
7.023
7.011
6.927
6.915
6.903
6.828
6.814
6.745
6.731
6.640
3.556
3.389
3.384
3.289
3.275
3.265
3.256
3.123
3.106
3.061
2.930
2.924
2.906
2.903
2.897
1.232
1.220
0.958
0.946
OH
O
Me
44
dr = 1:1
HNMR
CDCl3
600MHz
0.95
0.96
1.05
2.03
9.08
8.02
4.11
1.06
2.13
1.04
1.00
1.03
2.05
1.05
1.04
2.10
3.07
3.06
2.5 11.5 10.5 9.5 8.5 7.5 6.5 5.5 4.5 3.5 2.5 1.5 0.5 -0.5

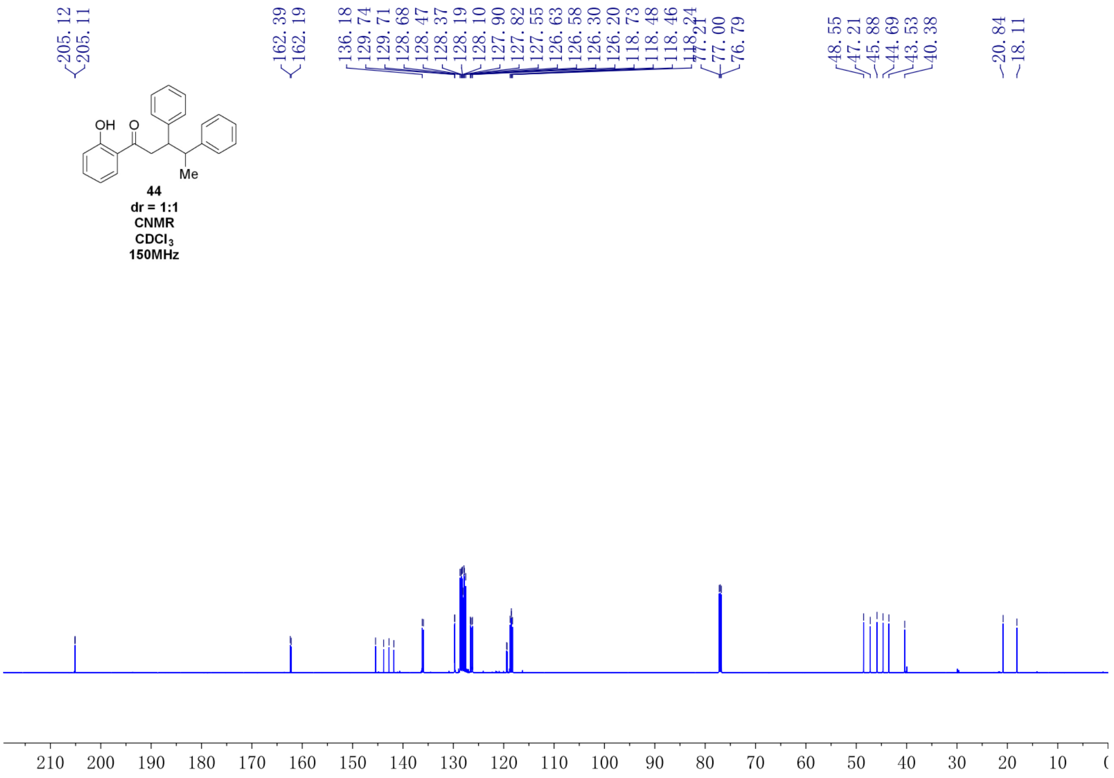
205.12
205.11
162.39
162.19
136.18
129.74
129.71
128.68
128.47
128.37
128.19
128.10
127.90
127.82
127.55
126.63
126.58
126.30
126.20
118.73
118.48
118.46
118.24
77.21
77.00
76.79
48.55
47.21
45.88
44.69
43.53
40.38
20.84
18.11
OH
O
Me
44
dr = 1:1
CNMR
CDCl3
150MHz
210 200 190 180 170 160 150 140 130 120 110 100 90 80 70 60 50 40 30 20 10

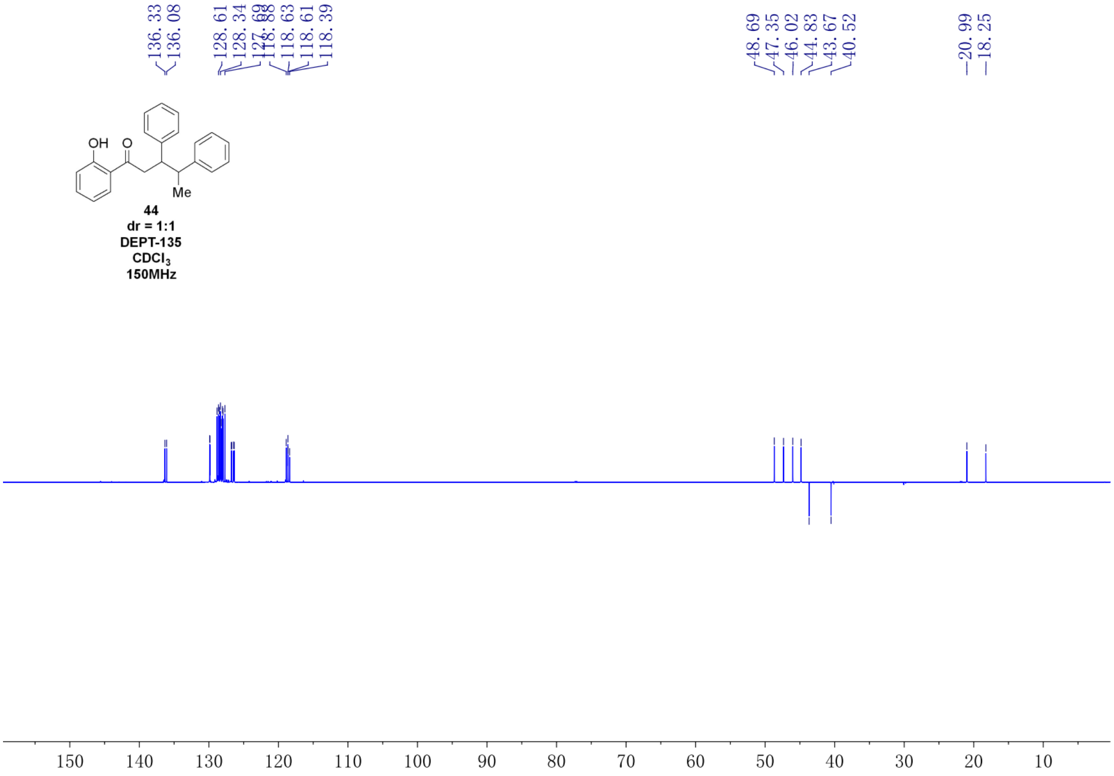
136.33
136.08
128.61
128.34
127.69
118.88
118.63
118.61
118.39
48.69
47.35
46.02
44.83
43.67
40.52
20.99
18.25
OH
O
Me
44
dr = 1:1
DEPT-135
CDCl3
150MHz
150 140 130 120 110 100 90 80 70 60 50 40 30 20 10

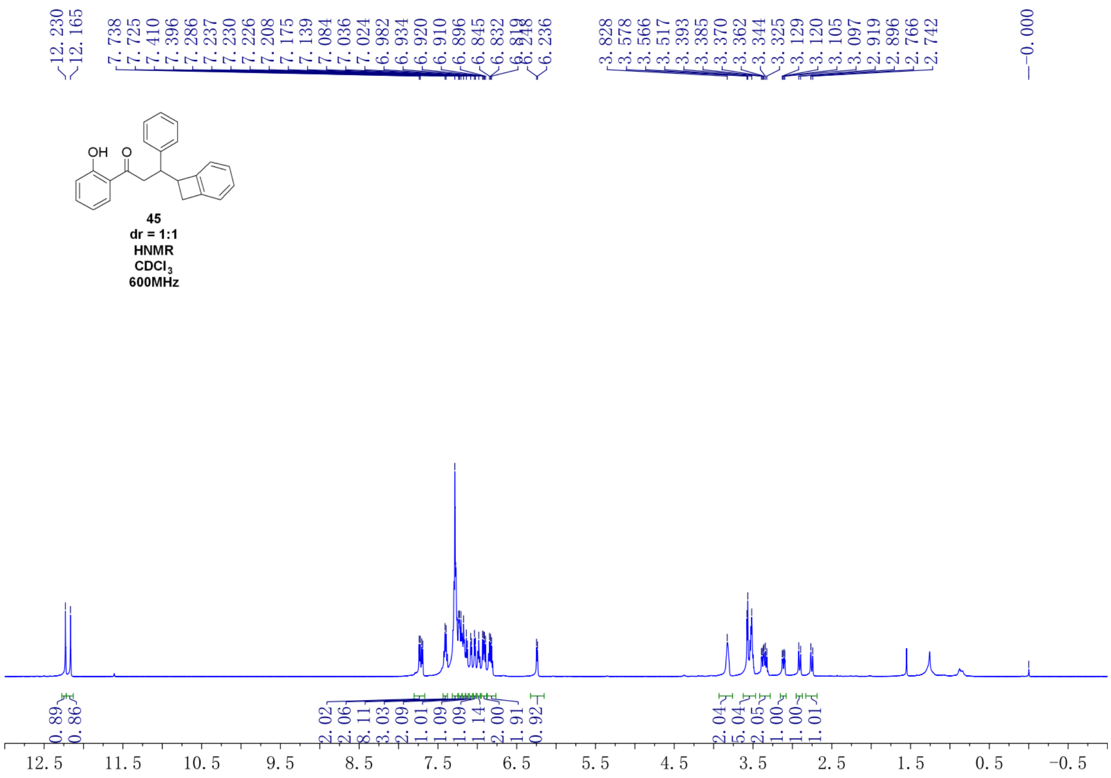
12.230
12.165
7.738
7.725
7.410
7.396
7.286
7.237
7.230
7.226
7.208
7.175
7.139
7.084
7.036
7.024
6.982
6.934
6.920
6.910
6.896
6.845
6.832
6.819
6.248
6.236
3.828
3.578
3.566
3.517
3.393
3.385
3.370
3.362
3.344
3.325
3.129
3.120
3.105
3.097
2.919
2.896
2.766
2.742
-0.000
OH
O
45
dr = 1:1
HNMR
CDCl3
600MHz
0.89
0.86
2.02
2.06
8.11
3.03
2.09
1.01
1.09
1.09
1.14
2.00
1.91
0.92
2.04
5.04
2.05
1.00
1.00
1.01
12.5
11.5
10.5
9.5
8.5
7.5
6.5
5.5
4.5
3.5
2.5
1.5
0.5
-0.5

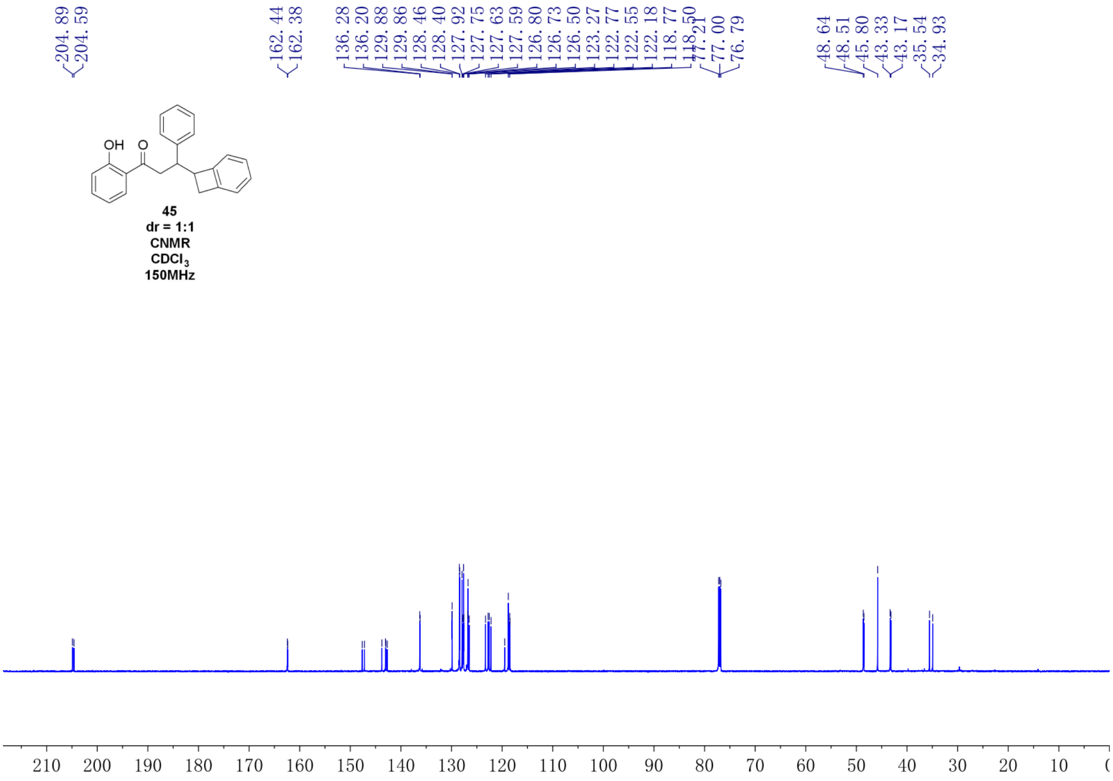
204.89
204.59
162.44
162.38
136.28
136.20
129.88
129.86
128.46
128.40
127.92
127.75
127.63
127.59
126.80
126.73
126.50
123.27
122.77
122.55
122.18
118.77
118.50
77.21
77.00
76.79
48.64
48.51
45.80
43.33
43.17
35.54
34.93
OH
O
45
dr = 1:1
CNMR
CDCl3
150MHz
210
200
190
180
170
160
150
140
130
120
110
100
90
80
70
60
50
40
30
20
10

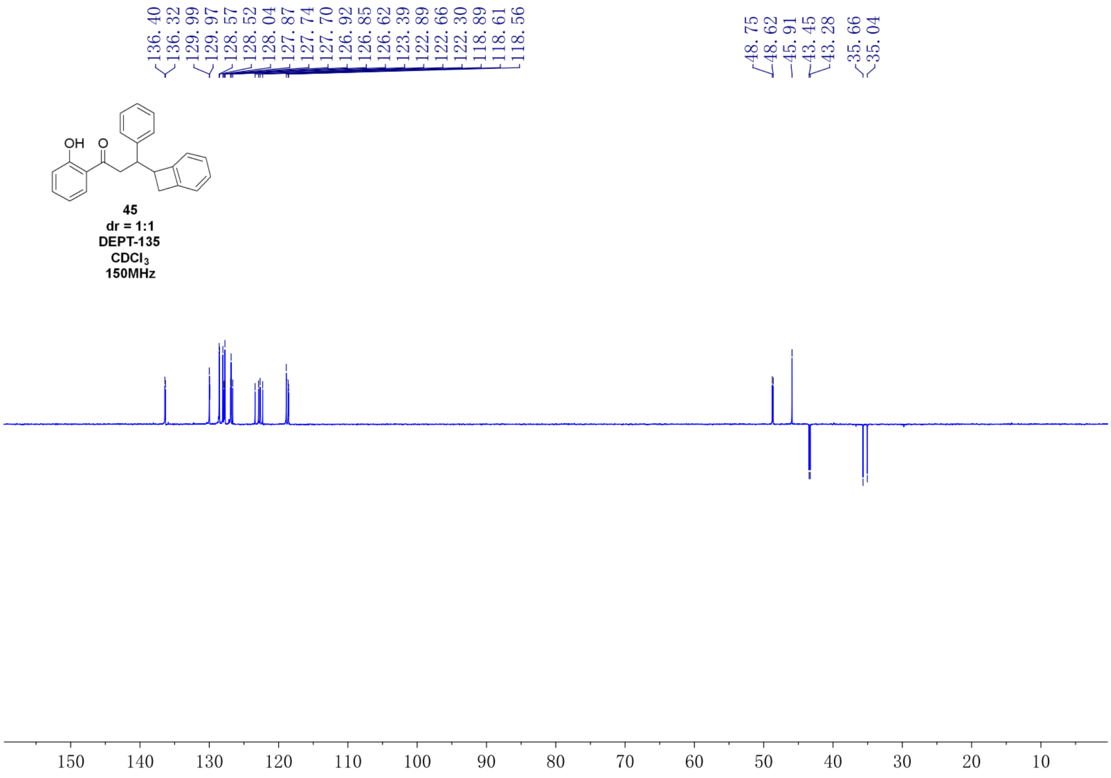
136.40
136.32
129.99
129.97
128.57
128.52
128.04
127.87
127.74
127.70
126.92
126.85
126.62
123.39
122.89
122.66
122.30
118.89
118.61
118.56
48.75
48.62
45.91
43.45
43.28
35.66
35.04
OH
O
45
dr = 1:1
DEPT-135
CDCl3
150MHz
150
140
130
120
110
100
90
80
70
60
50
40
30
20
10

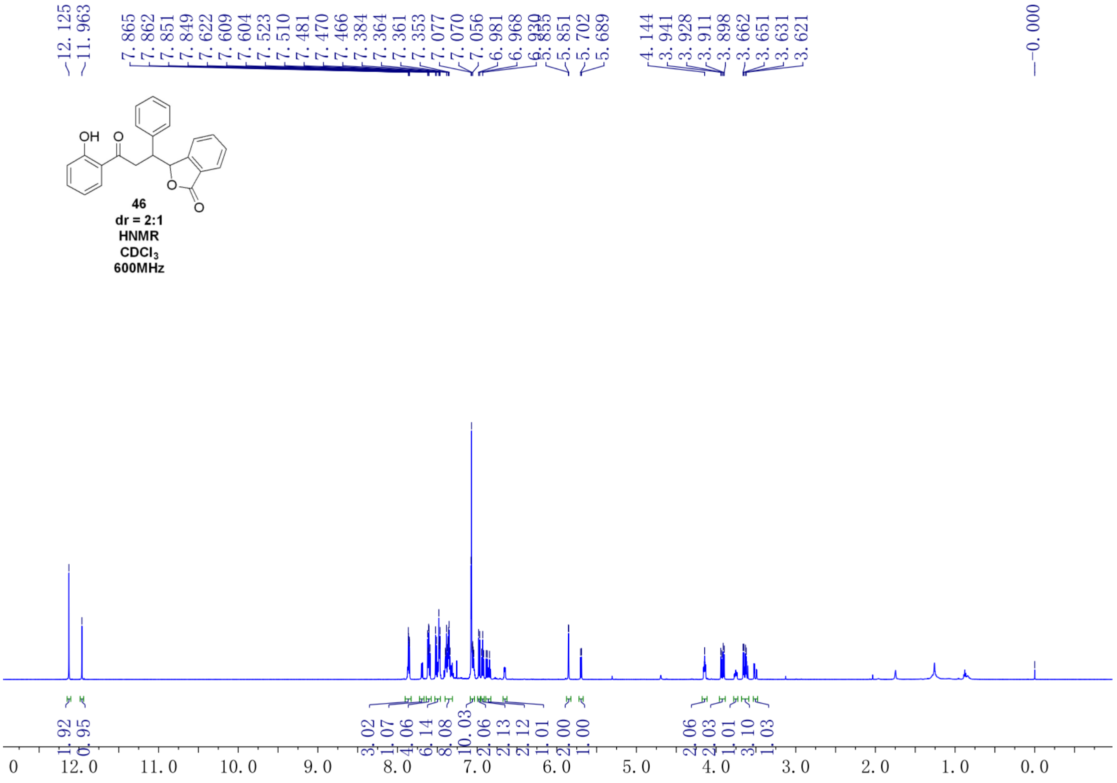
12.125
11.963
7.865
7.862
7.851
7.849
7.622
7.609
7.604
7.523
7.510
7.481
7.470
7.466
7.384
7.364
7.361
7.353
7.077
7.070
7.056
6.981
6.968
6.930
5.855
5.851
5.702
5.689
4.144
3.941
3.928
3.911
3.898
3.662
3.651
3.631
3.621
0.000
OH
O
O
O
46
dr = 2:1
HNMR
CDCl3
600MHz
1.92
0.95
3.02
1.07
4.06
6.14
8.08
10.03
2.06
2.13
2.12
1.01
2.00
1.00
2.06
2.03
1.01
3.10
1.03
0
12.0
11.0
10.0
9.0
8.0
7.0
6.0
5.0
4.0
3.0
2.0
1.0
0.0

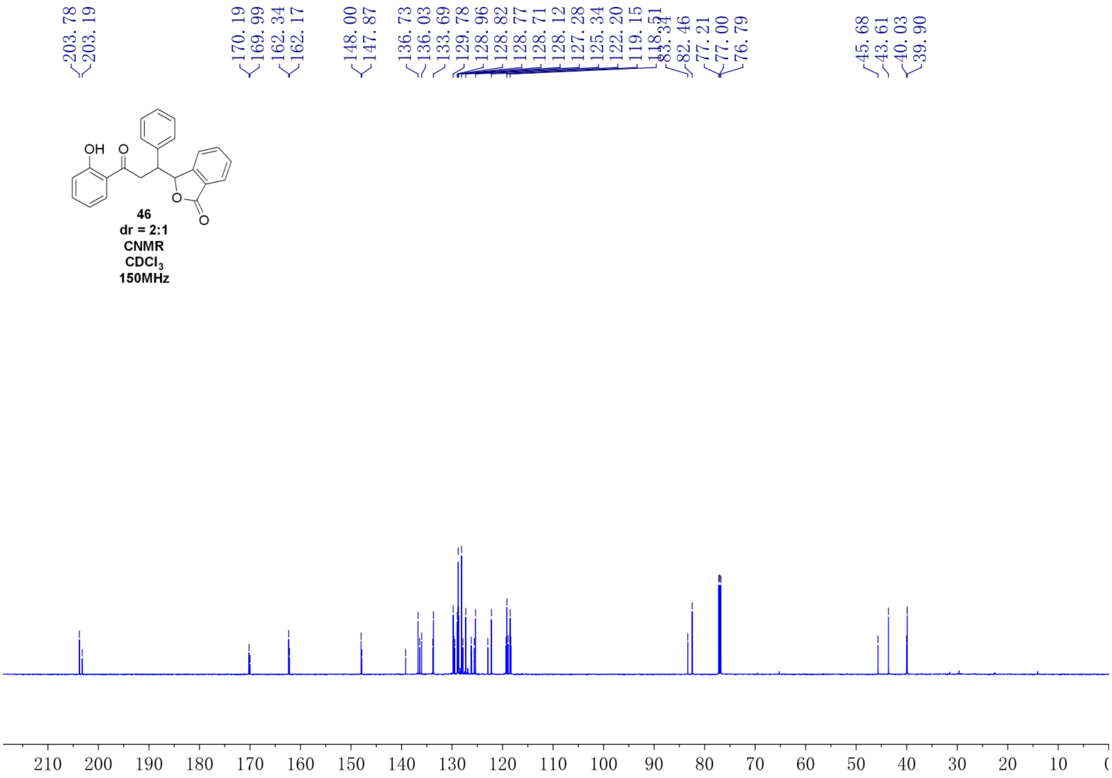
203. 78
203. 19
170. 19
169. 99
162. 34
162. 17
148. 00
147. 87
136. 73
136. 03
133. 69
129. 78
128. 96
128. 82
128. 77
128. 71
128. 12
127. 28
125. 34
122. 20
119. 15
118. 51
83. 34
82. 46
77. 21
77. 00
76. 79
45. 68
43. 61
40. 03
39. 90
OH
O
O
O
46
dr = 2:1
CNMR
CDCl3
150MHz
210
200
190
180
170
160
150
140
130
120
110
100
90
80
70
60
50
40
30
20
10

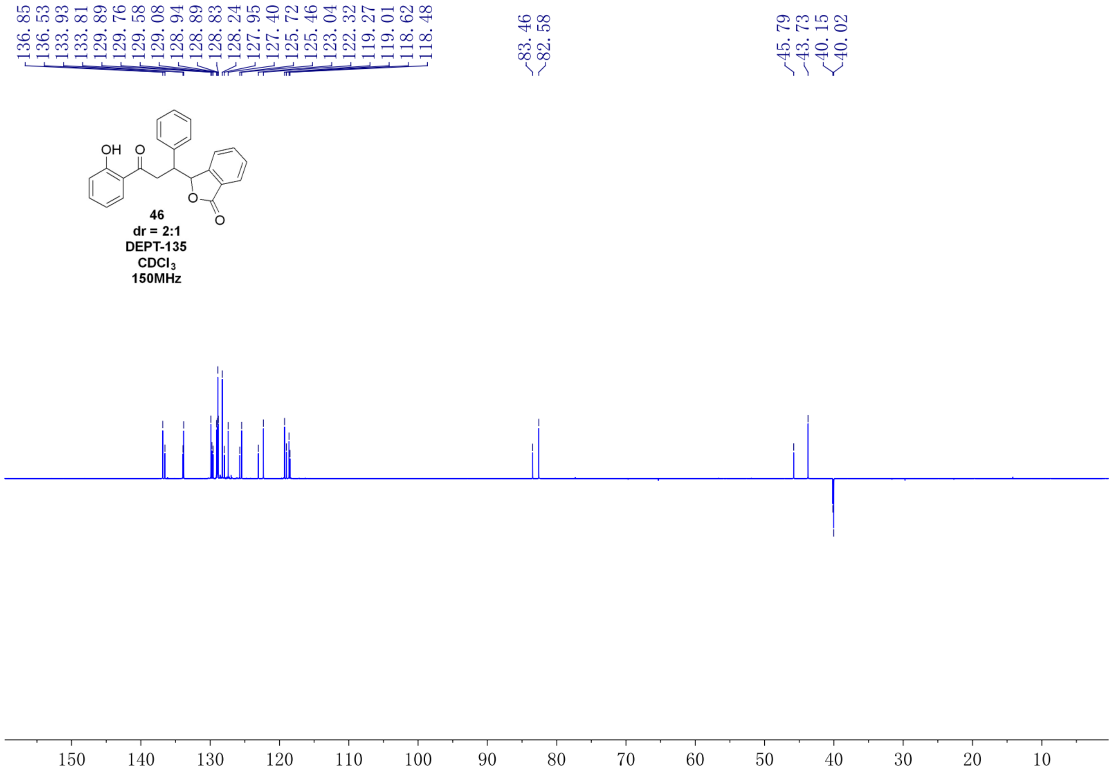
136. 85
136. 53
133. 93
133. 81
129. 89
129. 76
129. 58
129. 08
128. 94
128. 89
128. 83
128. 24
127. 95
127. 40
125. 72
125. 46
123. 04
122. 32
119. 27
119. 01
118. 62
118. 48
83. 46
82. 58
45. 79
43. 73
40. 15
40. 02
OH
O
O
O
46
dr = 2:1
DEPT-135
CDCl3
150MHz
150
140
130
120
110
100
90
80
70
60
50
40
30
20
10

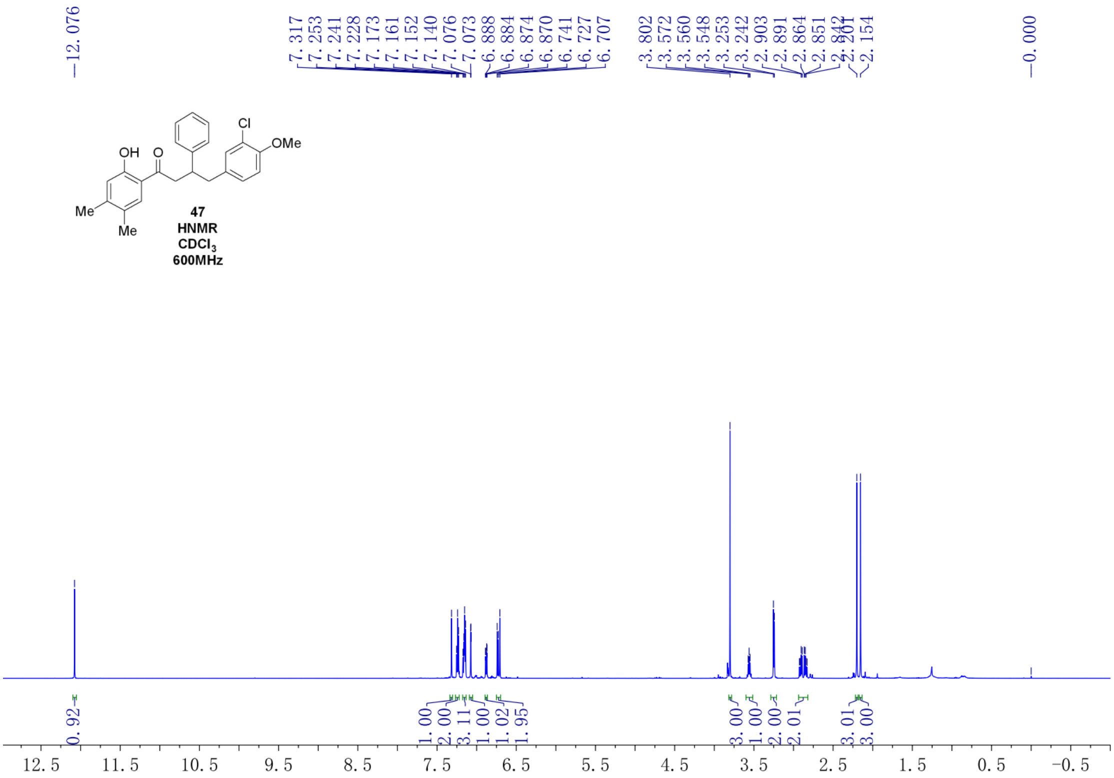
12.076
7.317
7.253
7.241
7.228
7.173
7.161
7.152
7.140
7.076
7.073
6.888
6.884
6.874
6.870
6.741
6.727
6.707
3.802
3.572
3.560
3.548
3.253
3.242
2.903
2.891
2.864
2.851
2.842
2.201
2.154
0.000
OH
O
Cl
OMe
Me
Me
47
HNMR
CDCl3
600MHz
0.92
1.00
2.00
3.11
1.00
1.02
1.95
3.00
1.00
2.00
2.01
3.01
3.00
12.5 11.5 10.5 9.5 8.5 7.5 6.5 5.5 4.5 3.5 2.5 1.5 0.5 -0.5

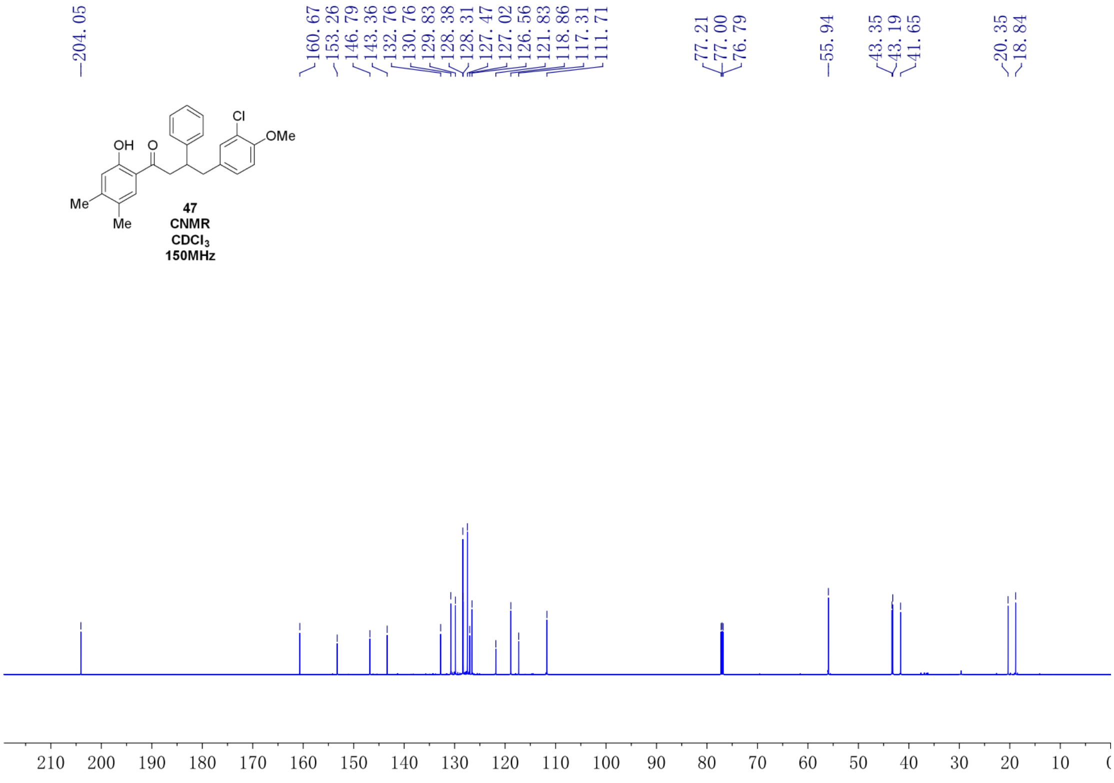
204.05
160.67
153.26
146.79
143.36
132.76
130.83
129.83
128.38
128.31
127.47
127.02
126.56
121.83
118.86
117.31
111.71
77.21
77.00
76.79
55.94
43.35
43.19
41.65
20.35
18.84
OH
O
Cl
OMe
Me
Me
47
CNMR
CDCl3
150MHz
210 200 190 180 170 160 150 140 130 120 110 100 90 80 70 60 50 40 30 20 10

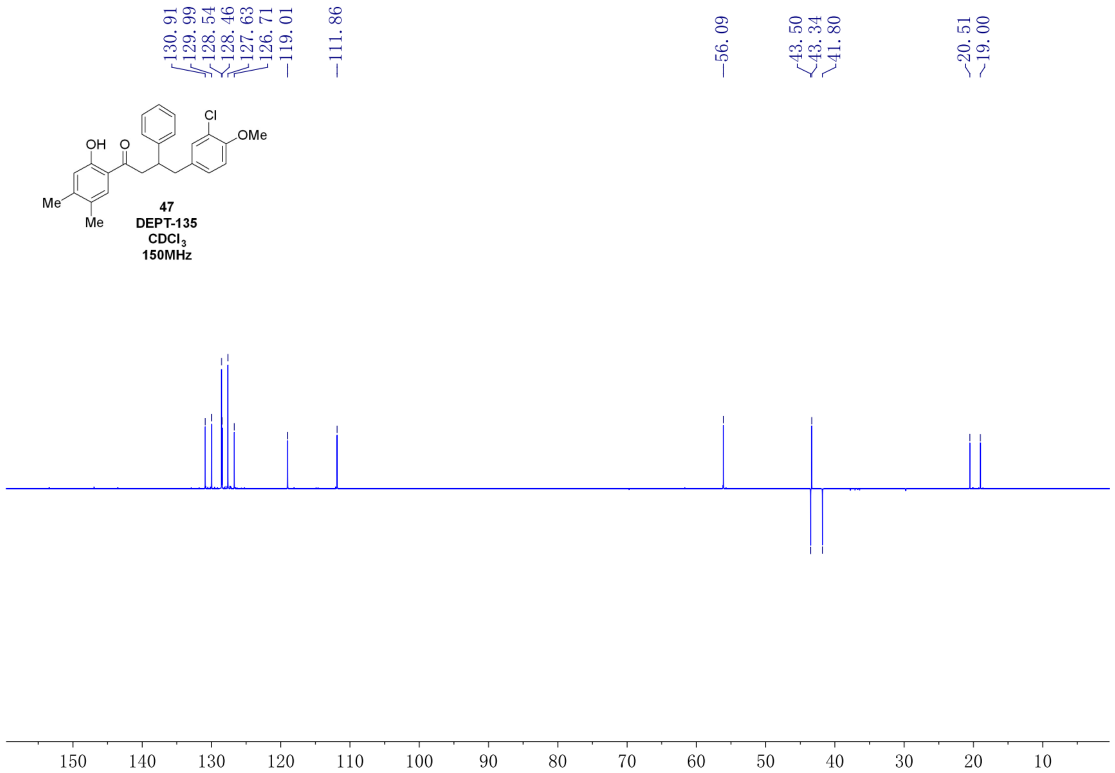
130.91
129.99
128.54
128.46
127.63
126.71
119.01
111.86
56.09
43.50
43.34
41.80
20.51
19.00
OH
O
Cl
OMe
Me
Me
47
DEPT-135
$CDCl_3$
150MHz
150 140 130 120 110 100 90 80 70 60 50 40 30 20 10

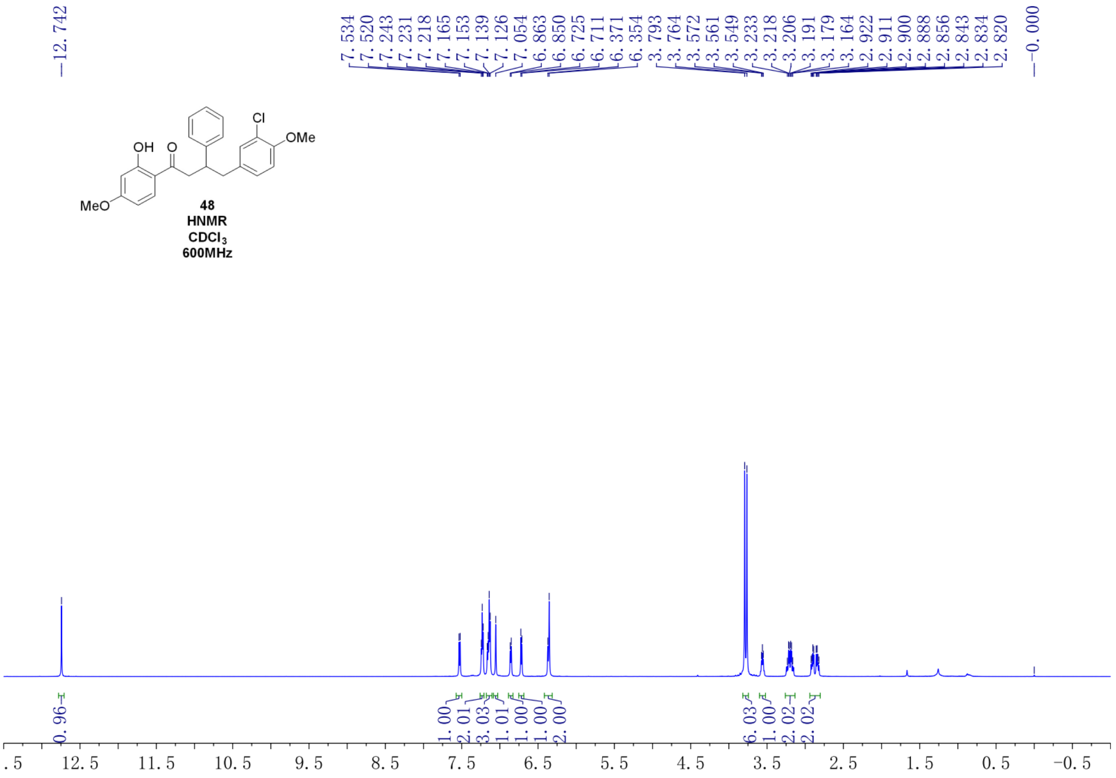
12.742
7.534
7.520
7.243
7.231
7.218
7.165
7.153
7.139
7.126
7.054
6.863
6.850
6.725
6.711
6.371
6.354
3.793
3.764
3.572
3.561
3.549
3.233
3.218
3.206
3.191
3.179
3.164
2.922
2.911
2.900
2.888
2.856
2.843
2.834
2.820
0.000
OH
O
Cl
OMe
MeO
48
HNMR
$CDCl_3$
600MHz
0.96
1.00
2.01
3.03
1.01
1.00
1.00
2.00
6.03
1.00
2.02
2.02
.5 12.5 11.5 10.5 9.5 8.5 7.5 6.5 5.5 4.5 3.5 2.5 1.5 0.5 -0.5

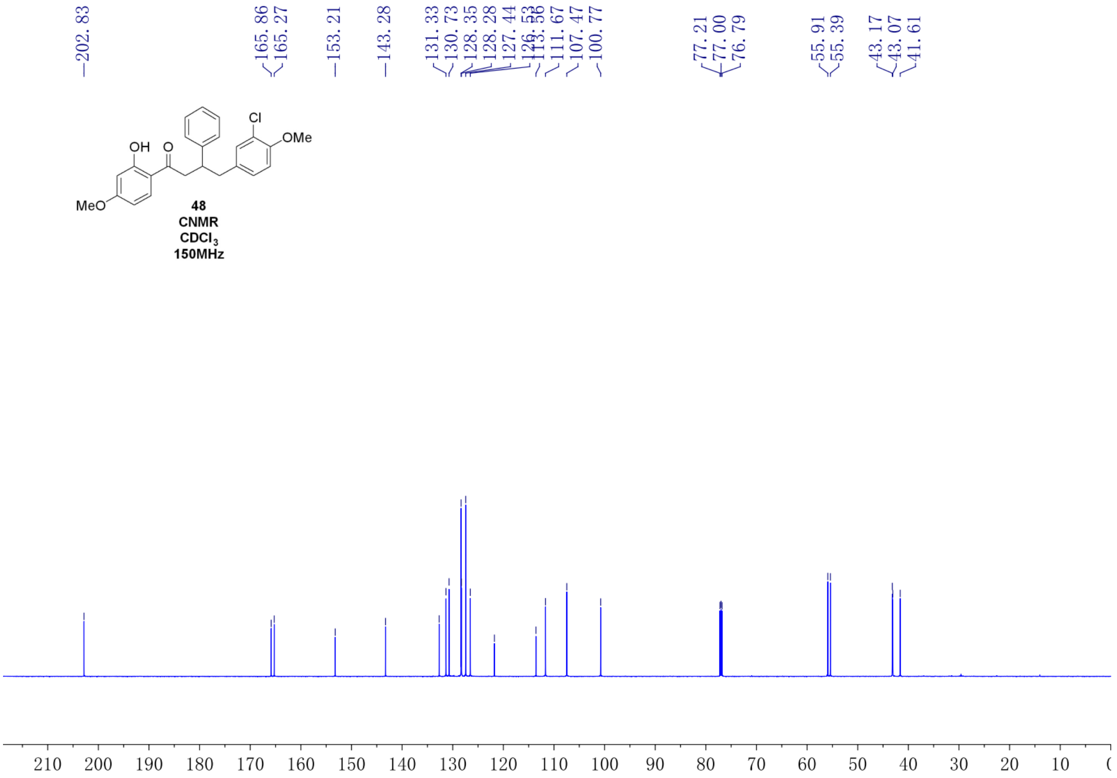
202.83
165.86
165.27
153.21
143.28
131.33
130.73
128.35
128.28
127.44
126.53
113.56
111.67
107.47
100.77
77.21
77.00
76.79
55.91
55.39
43.17
43.07
41.61
OH
O
Cl
OMe
MeO
48
CNMR
CDCl3
150MHz
210 200 190 180 170 160 150 140 130 120 110 100 90 80 70 60 50 40 30 20 10

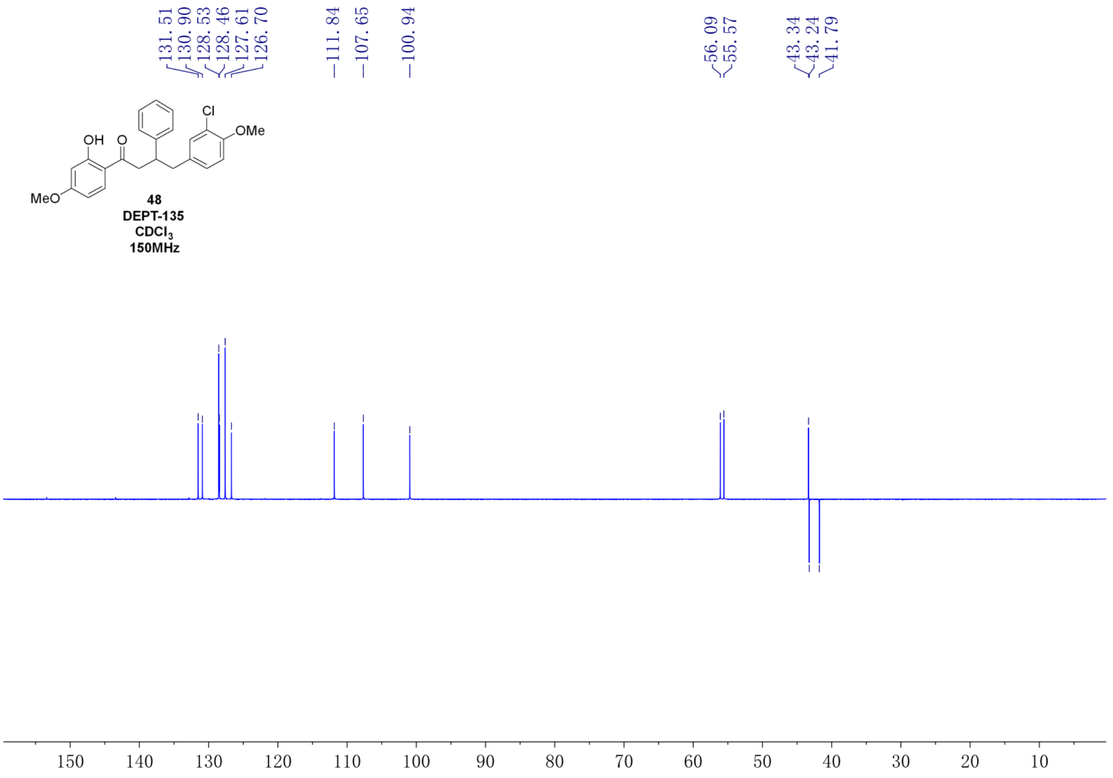
131.51
130.90
128.53
128.46
127.61
126.70
111.84
107.65
100.94
56.09
55.57
43.34
43.24
41.79
OH
O
Cl
OMe
MeO
48
DEPT-135
CDCl3
150MHz
150 140 130 120 110 100 90 80 70 60 50 40 30 20 10

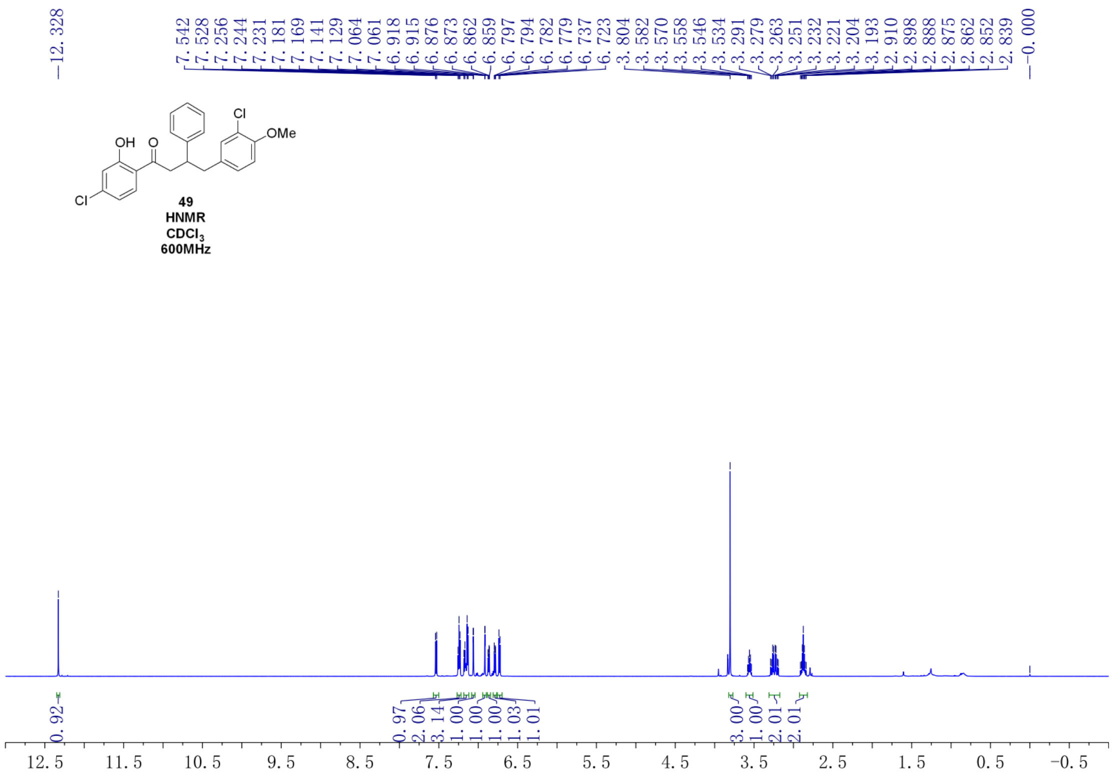
12.328
7.542
7.528
7.256
7.244
7.231
7.181
7.169
7.141
7.129
7.064
7.061
6.918
6.915
6.876
6.873
6.862
6.859
6.797
6.794
6.782
6.779
6.737
6.723
3.804
3.582
3.570
3.558
3.546
3.534
3.291
3.279
3.263
3.251
3.232
3.221
3.204
3.193
2.910
2.898
2.888
2.875
2.862
2.852
2.839
0.000
OH
O
Cl
OMe
Cl
49
HNMR
$CDCl_3$
600MHz
0.92
0.97
2.06
3.14
1.00
1.00
1.00
1.03
1.01
3.00
1.00
2.01
2.01
12.5
11.5
10.5
9.5
8.5
7.5
6.5
5.5
4.5
3.5
2.5
1.5
0.5
-0.5

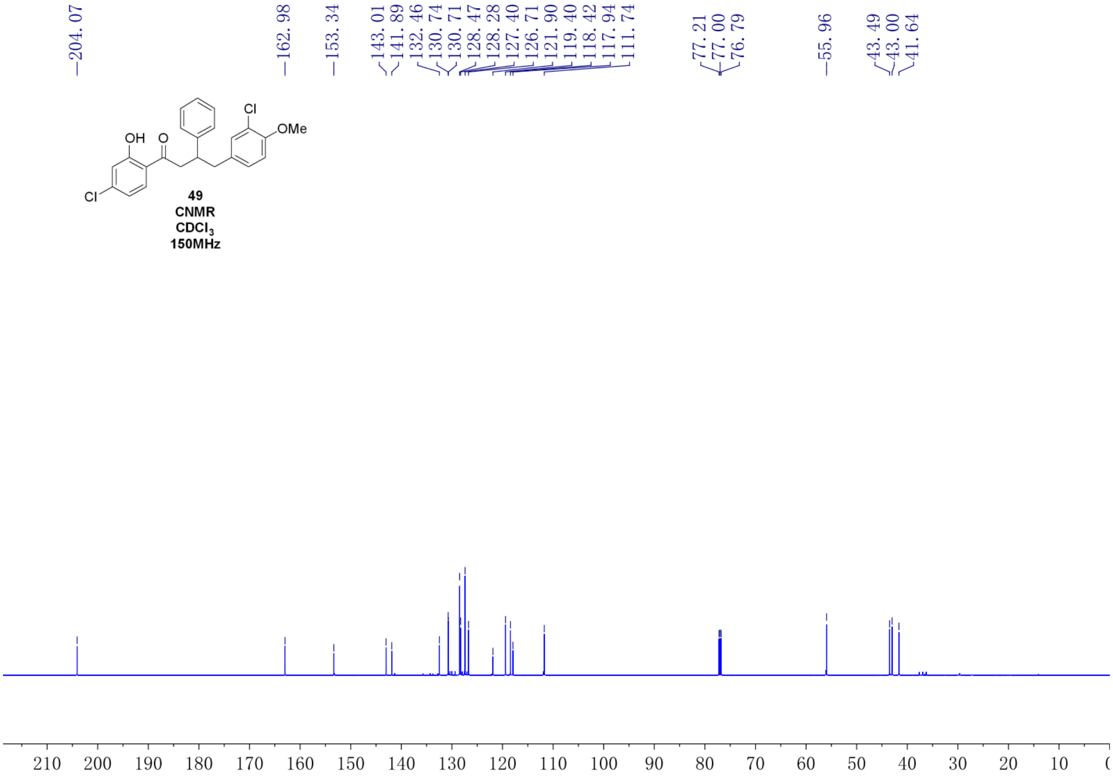
204.07
162.98
153.34
143.01
141.89
132.46
130.74
130.71
128.47
128.28
127.40
126.71
121.90
119.40
118.42
117.94
111.74
77.21
77.00
76.79
55.96
43.49
43.00
41.64
OH
O
Cl
OMe
Cl
49
CNMR
$CDCl_3$
150MHz
210
200
190
180
170
160
150
140
130
120
110
100
90
80
70
60
50
40
30
20
10

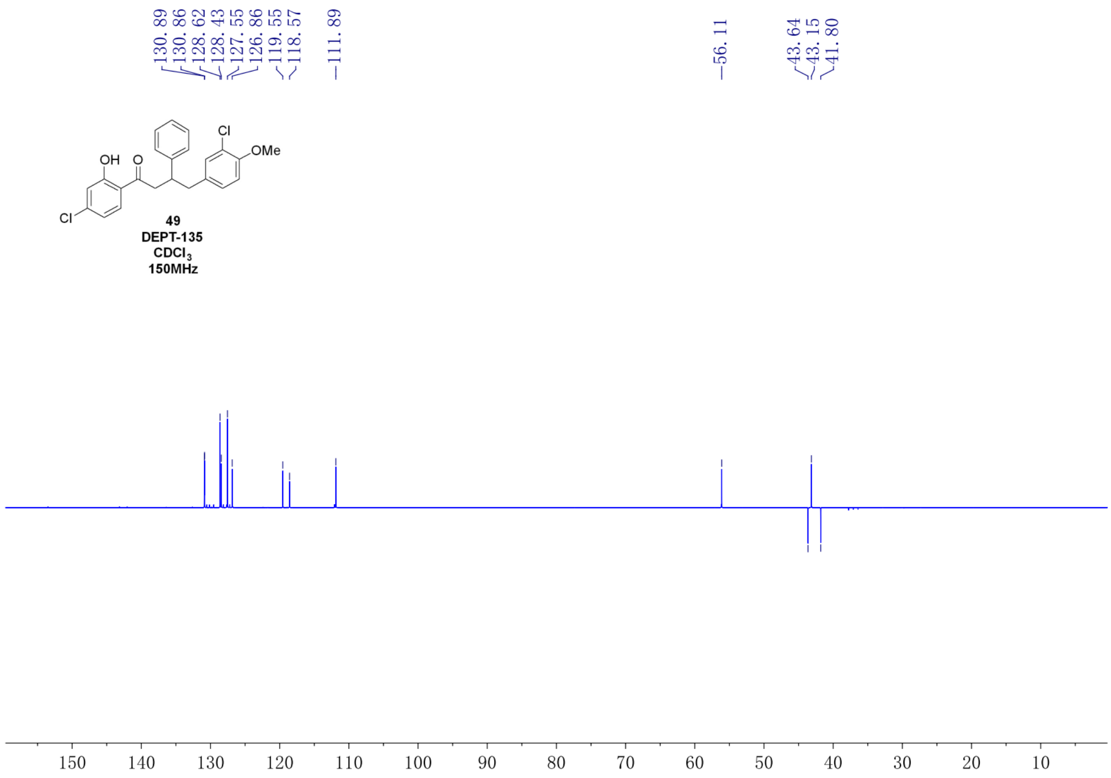
130.89
130.86
128.62
128.43
127.55
126.86
119.55
118.57
111.89
56.11
43.64
43.15
41.80
OH
O
Cl
OMe
Cl
49
DEPT-135
CDCl3
150MHz
150 140 130 120 110 100 90 80 70 60 50 40 30 20 10

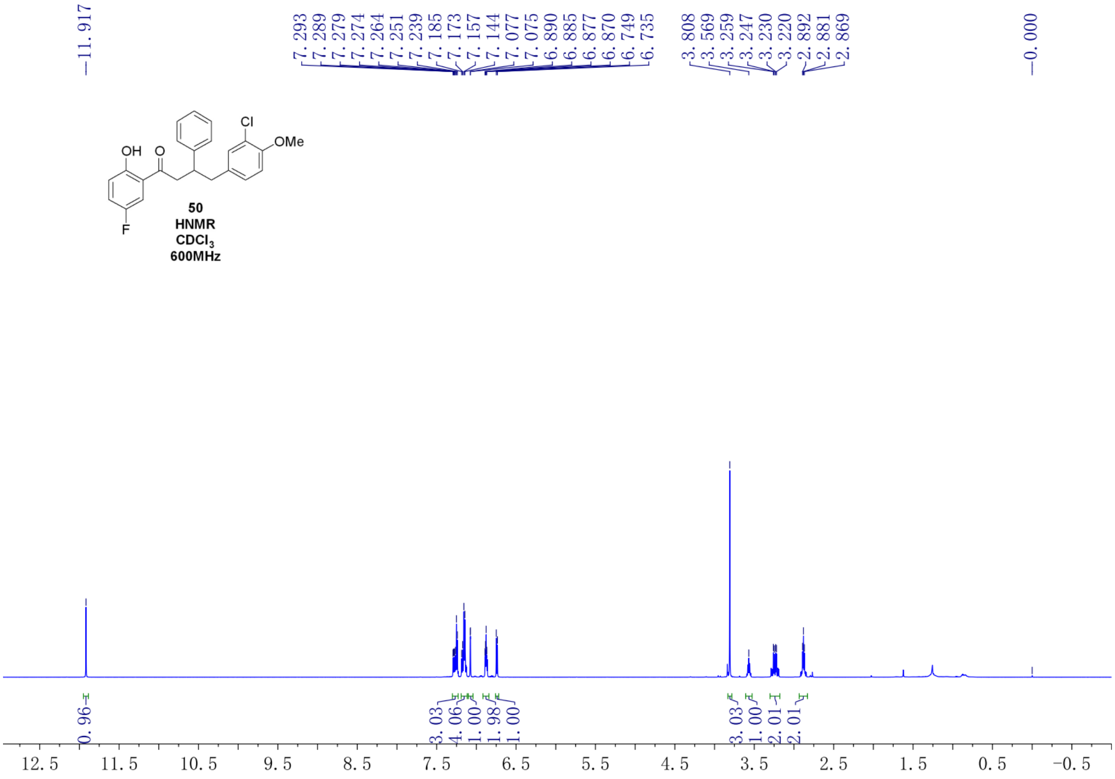
11.917
7.293
7.289
7.279
7.274
7.264
7.251
7.239
7.185
7.173
7.157
7.144
7.077
7.075
6.890
6.885
6.877
6.870
6.749
6.735
3.808
3.569
3.259
3.247
3.230
3.220
2.892
2.881
2.869
0.000
OH
O
Cl
OMe
F
50
HNMR
CDCl3
600MHz
0.96
3.03
4.06
1.00
1.98
1.00
3.03
1.00
2.01
2.01
12.5 11.5 10.5 9.5 8.5 7.5 6.5 5.5 4.5 3.5 2.5 1.5 0.5 -0.5

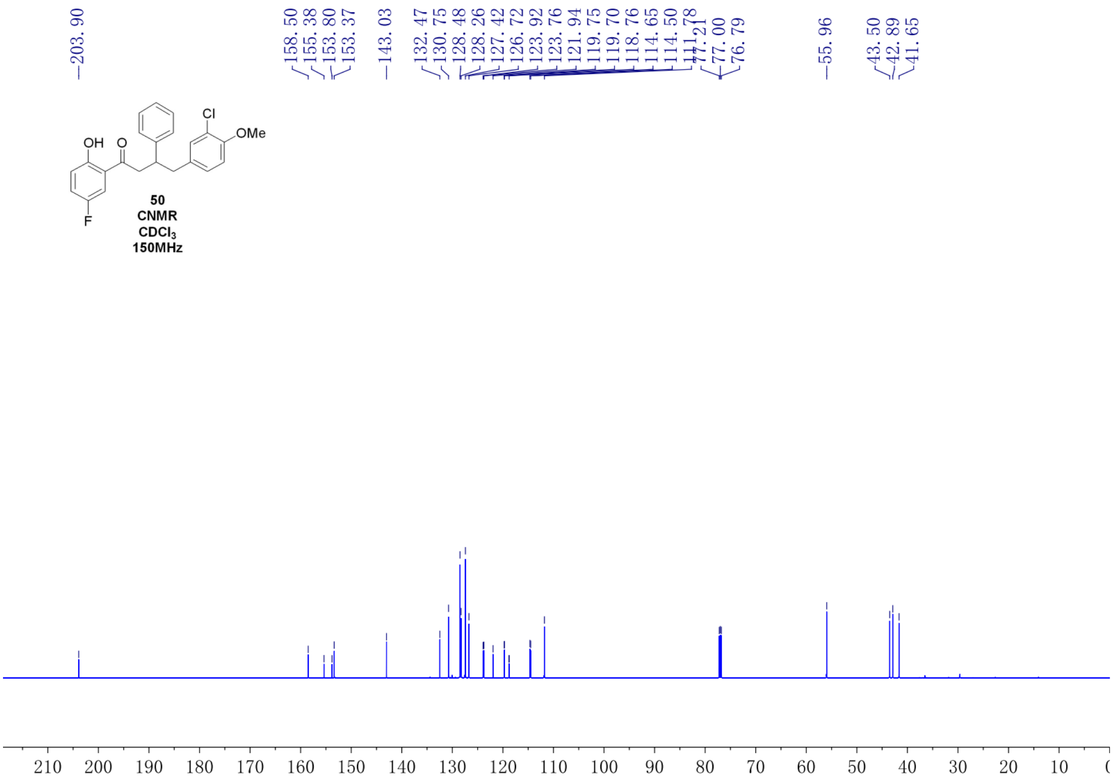

50
CNMR
$CDCl_3$
150MHz

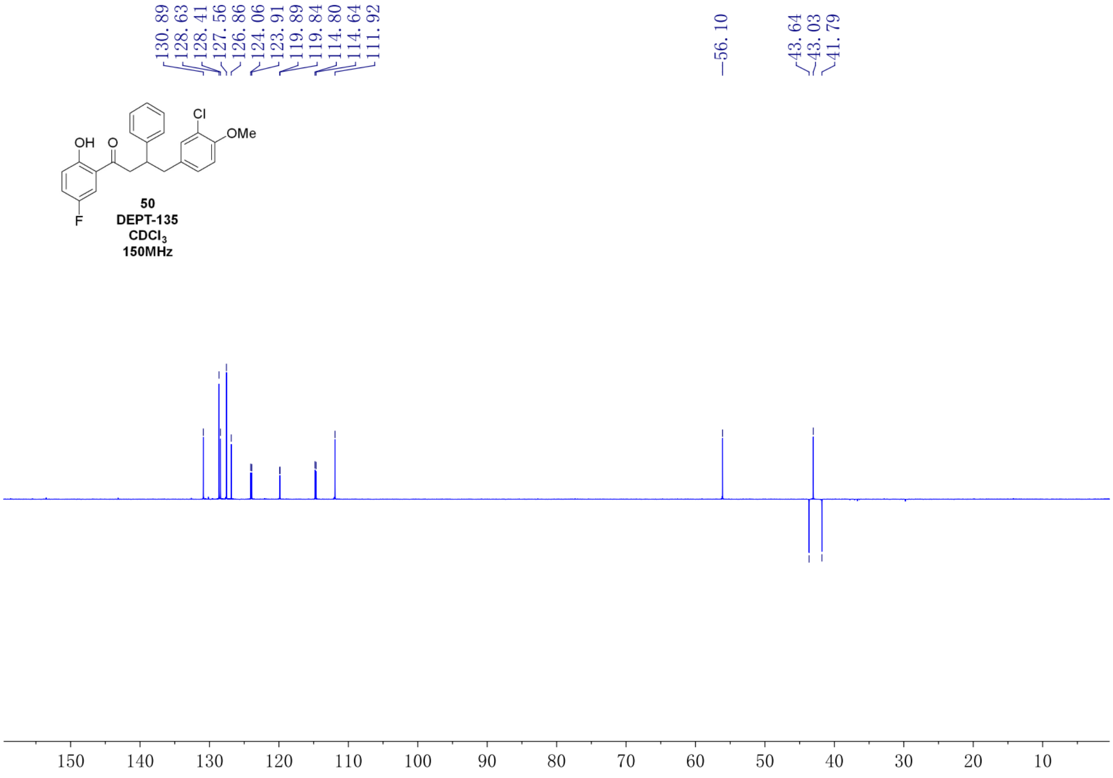

50
DEPT-135
$CDCl_3$
150MHz

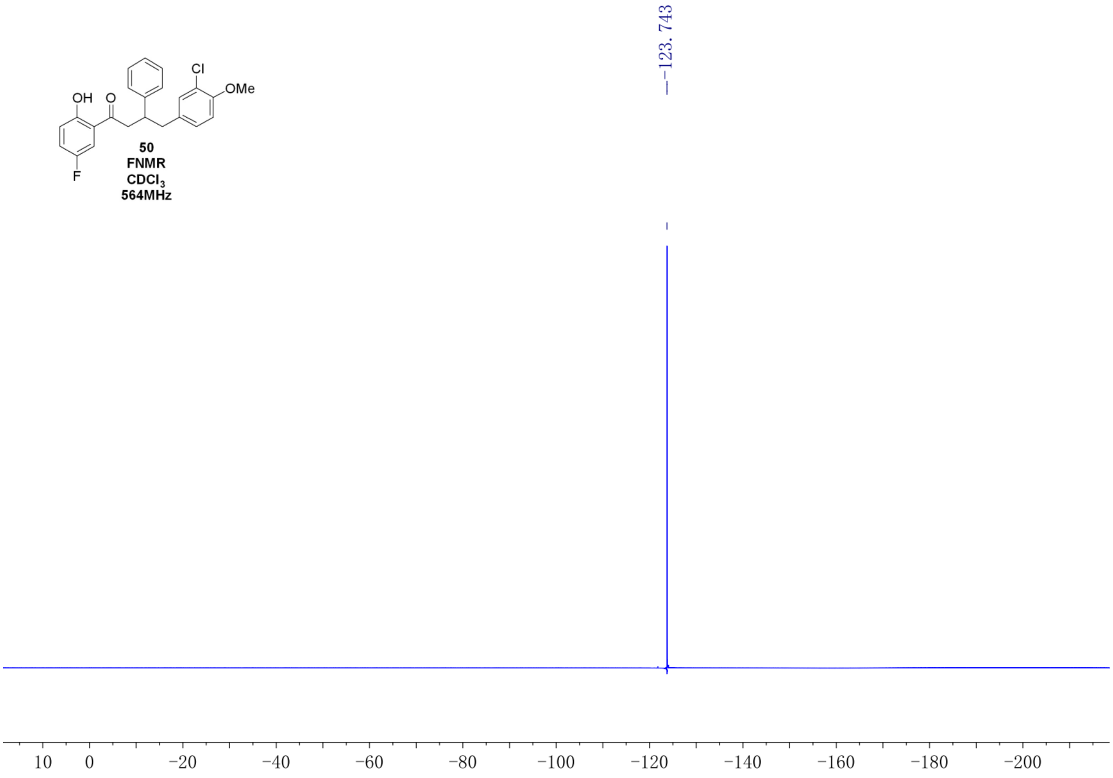

Cl
OMe
OH
O
F
50
FNMR
CDCl3
564MHz
-123.743
10 0 -20 -40 -60 -80 -100 -120 -140 -160 -180 -200

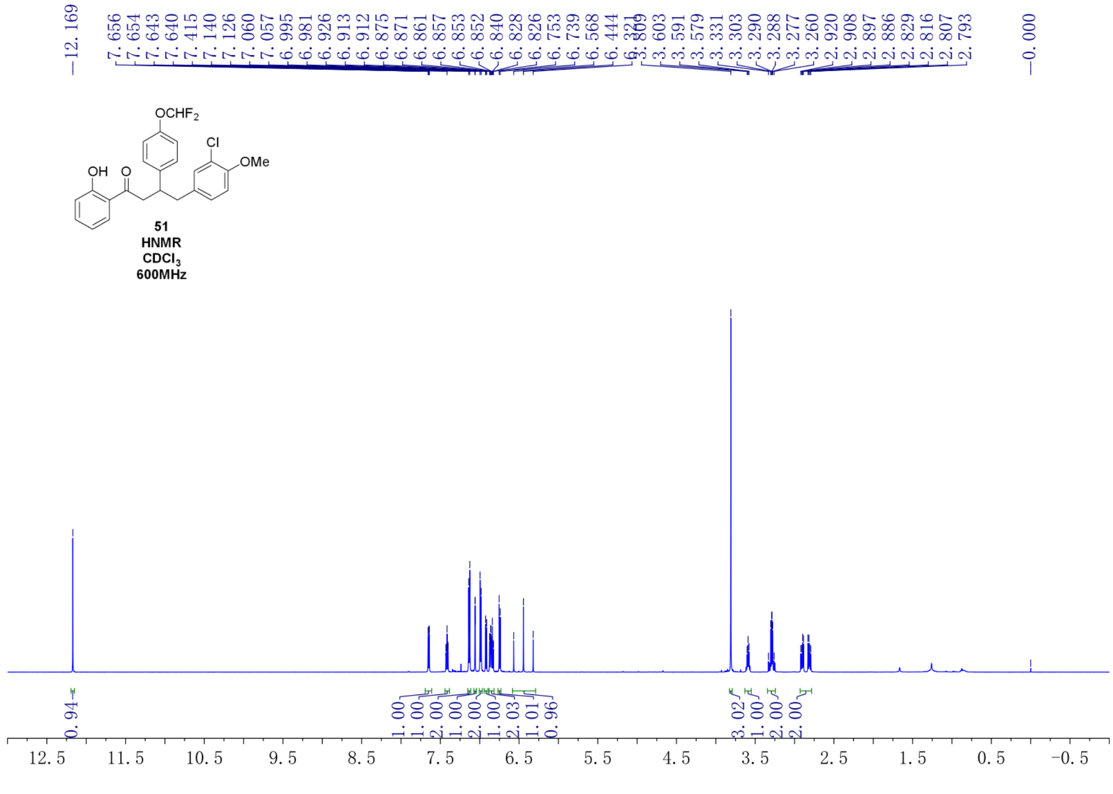

12.169
7.656 7.654 7.643 7.640 7.415 7.140 7.126 7.060 7.057 6.995 6.981 6.926 6.913 6.912 6.875 6.871 6.861 6.857 6.853 6.852 6.840 6.828 6.826 6.753 6.739 6.568 6.444 6.321 3.809 3.603 3.591 3.579 3.331 3.303 3.290 3.288 3.277 3.260 2.920 2.908 2.897 2.886 2.829 2.816 2.807 2.793
0.000
OCHF2
Cl
OMe
OH
O
51
HNMR
CDCl3
600MHz
0.94
1.00 1.00 2.00 1.00 2.00 1.00 2.03 1.01 0.96
3.02 1.00 2.00 2.00
12.5 11.5 10.5 9.5 8.5 7.5 6.5 5.5 4.5 3.5 2.5 1.5 0.5 -0.5

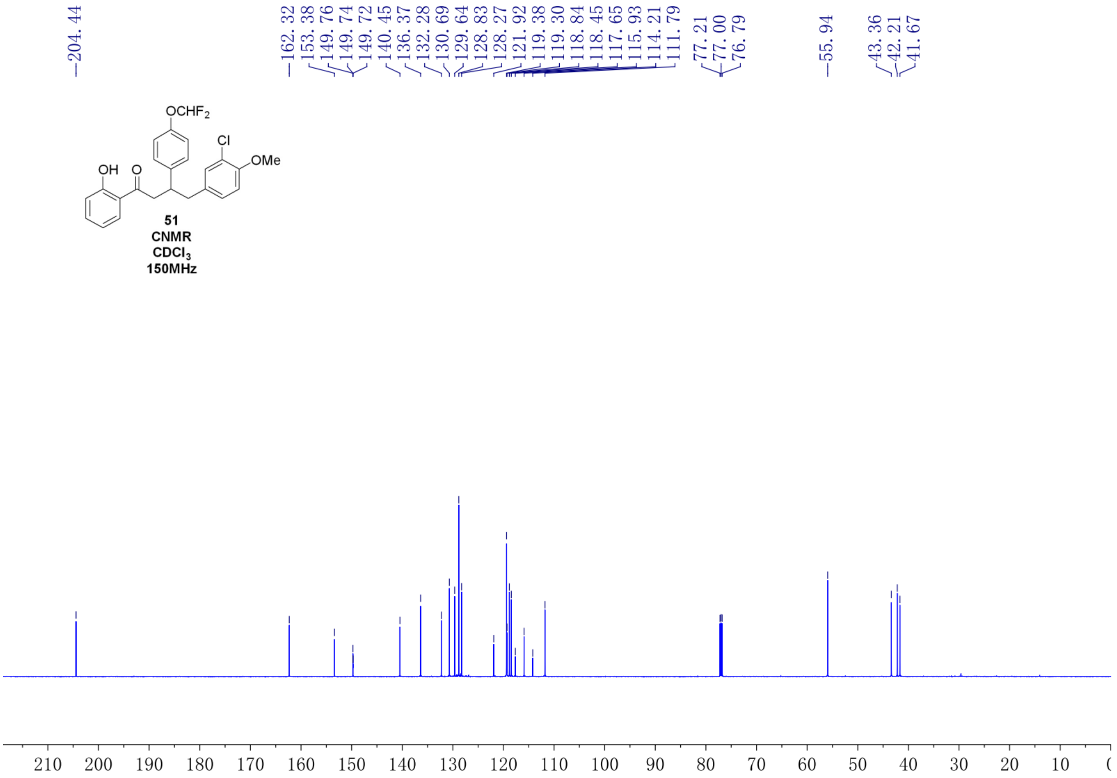
204.44
162.32
153.38
149.76
149.74
149.72
140.45
136.37
132.28
130.69
129.64
128.83
128.27
121.92
119.38
119.30
118.84
118.45
117.65
115.93
114.21
111.79
77.21
77.00
76.79
55.94
43.36
42.21
41.67
OCHF2
Cl
OMe
OH
O
51
CNMR
CDCl3
150MHz
210 200 190 180 170 160 150 140 130 120 110 100 90 80 70 60 50 40 30 20 10

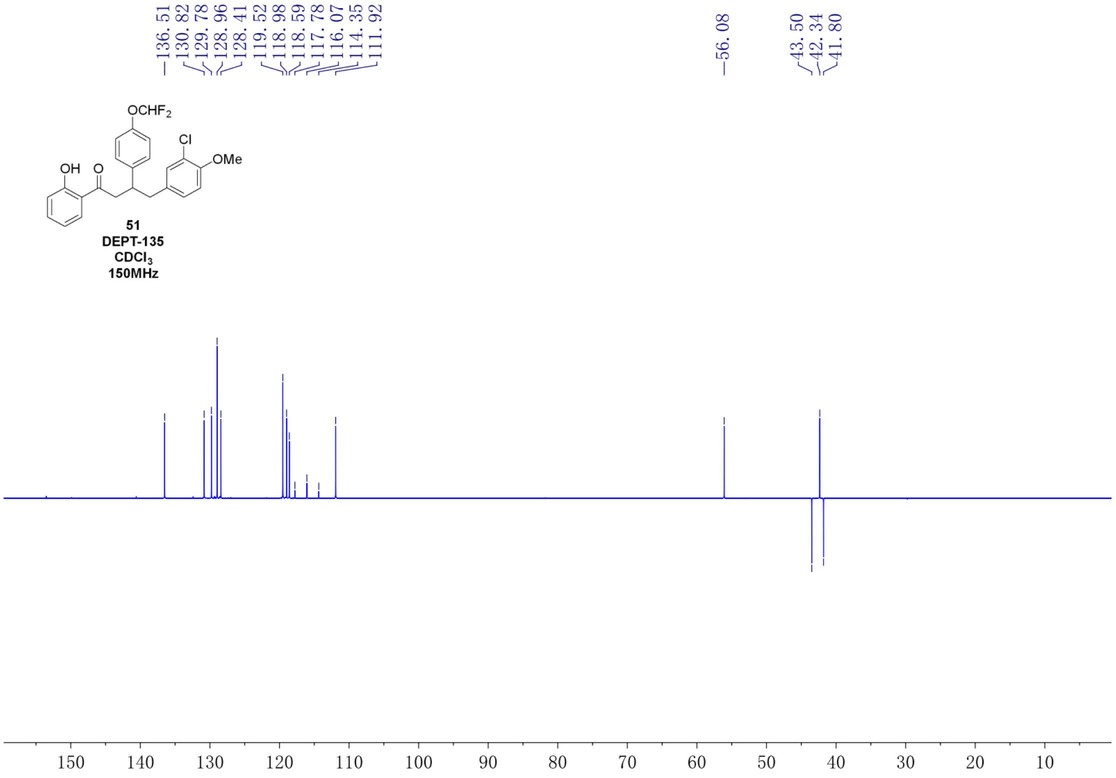
136.51
130.82
129.78
128.96
128.41
119.52
118.98
118.59
117.78
116.07
114.35
111.92
56.08
43.50
42.34
41.80
OCHF2
Cl
OMe
OH
O
51
DEPT-135
CDCl3
150MHz
150 140 130 120 110 100 90 80 70 60 50 40 30 20 10

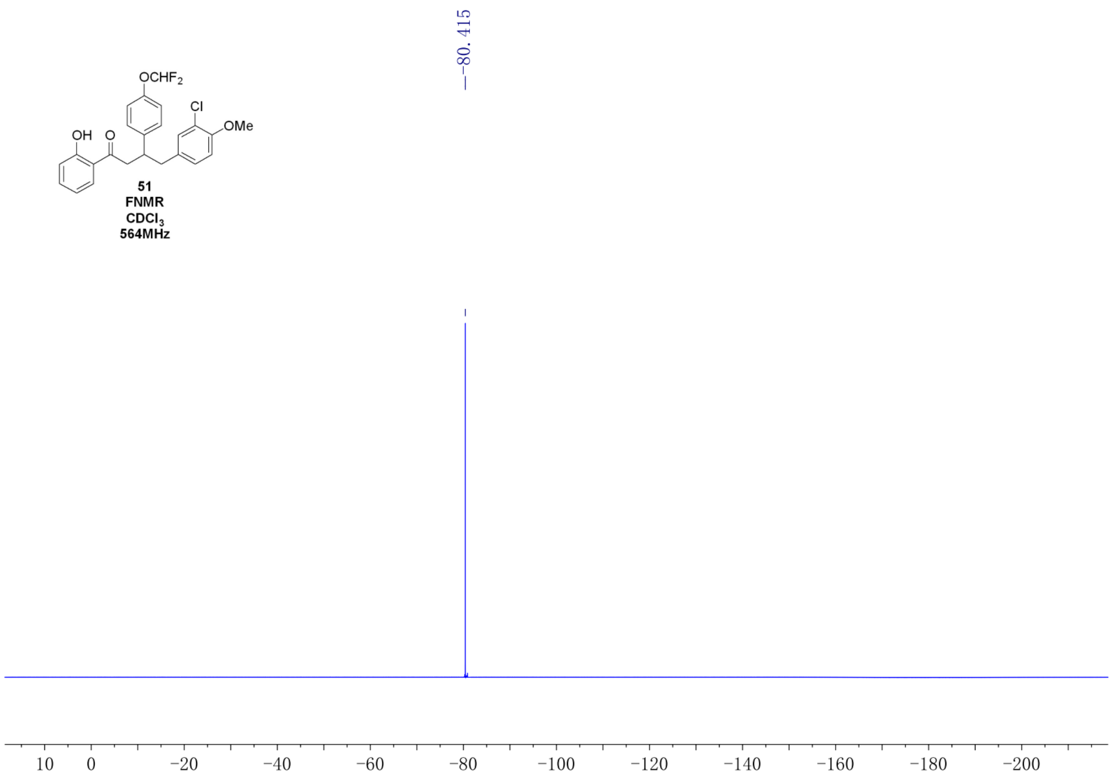
OCHF2
Cl
OMe
OH
O
51
FNMR
CDCl3
564MHz
-80.415
10 0 -20 -40 -60 -80 -100 -120 -140 -160 -180 -200

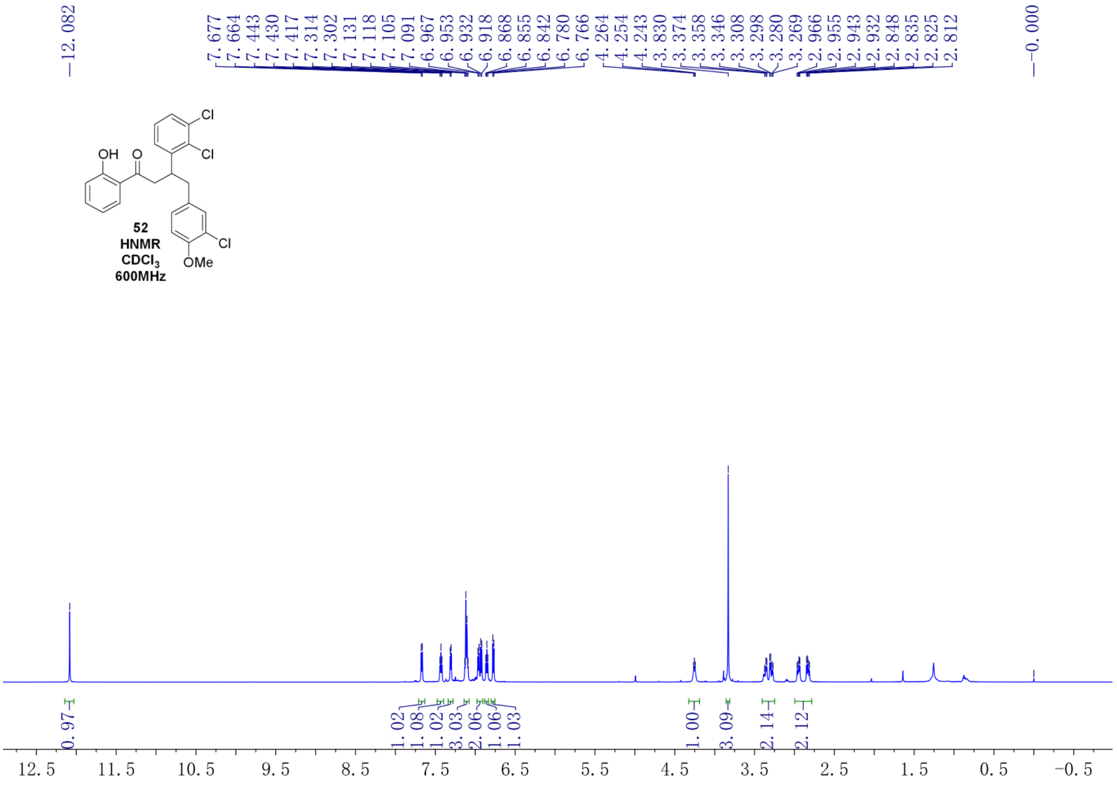
12.082
7.677 7.664 7.443 7.430 7.417 7.314 7.302 7.131 7.118 7.105 7.091 6.967 6.953 6.932 6.918 6.868 6.855 6.842 6.780 6.766
4.264 4.254 4.243 3.830 3.374 3.358 3.346 3.308 3.298 3.280 3.269 2.966 2.955 2.943 2.932 2.848 2.835 2.825 2.812
-0.000
Cl
Cl
OH
O
Cl
OMe
52
HNMR
CDCl3
600MHz
0.97 1.02 1.08 1.02 3.03 2.06 1.06 1.03 1.00 3.09 2.14 2.12
12.5 11.5 10.5 9.5 8.5 7.5 6.5 5.5 4.5 3.5 2.5 1.5 0.5 -0.5

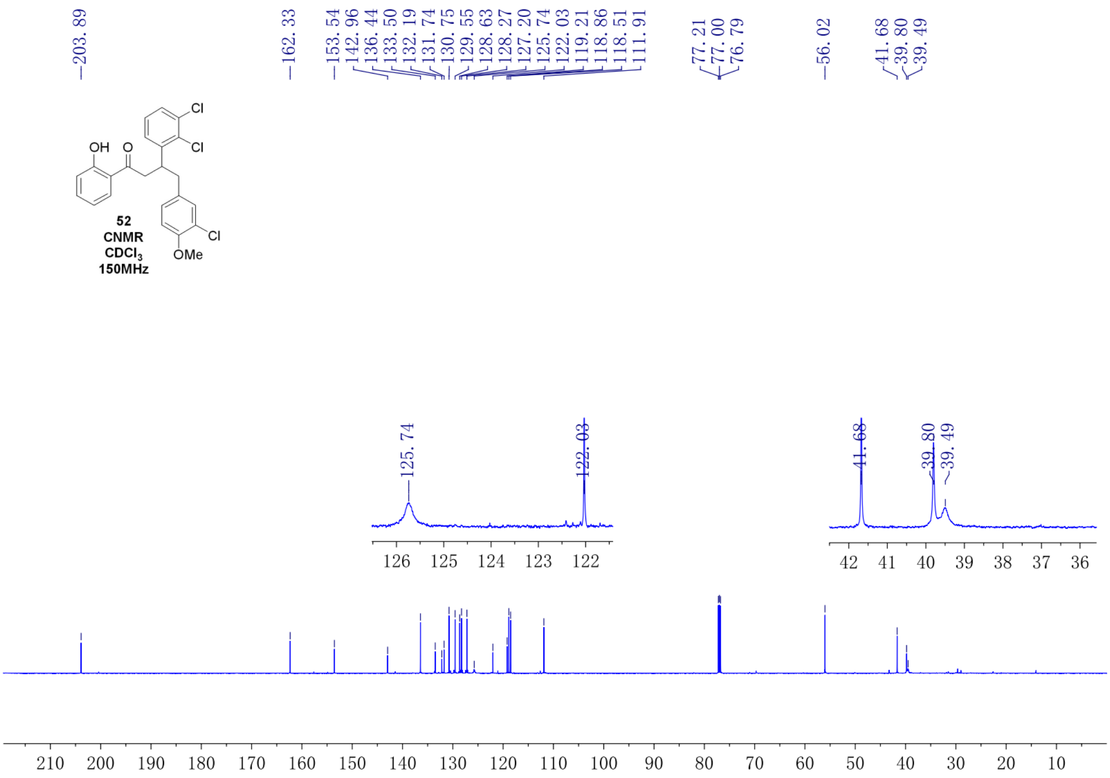
203.89
162.33
153.54
142.96
136.44
133.50
132.19
131.74
130.75
129.55
128.63
128.27
127.20
125.74
122.03
119.21
118.86
118.51
111.91
77.21
77.00
76.79
56.02
41.68
39.80
39.49
OH
O
Cl
Cl
Cl
OMe
52
CNMR
CDCl3
150MHz
125.74
122.03
41.68
39.80
39.49

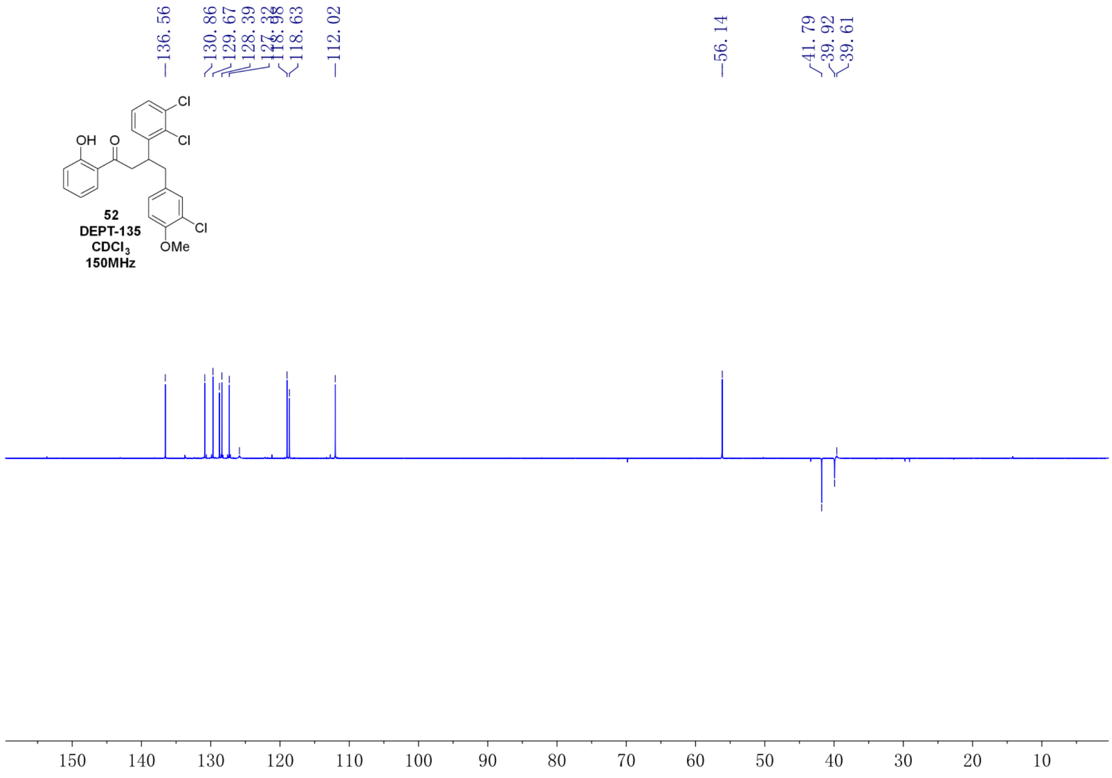
136.56
130.86
129.67
128.39
127.32
118.98
118.63
112.02
56.14
41.79
39.92
39.61
OH
O
Cl
Cl
Cl
OMe
52
DEPT-135
CDCl3
150MHz

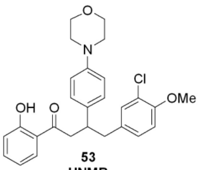

53
HNMR
$CDCl_3$
600MHz

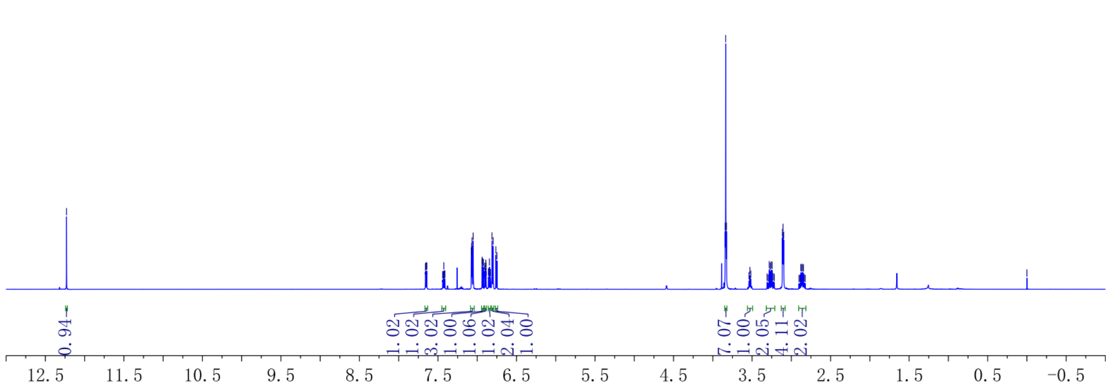

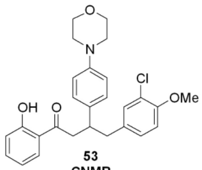

53
CNMR
$CDCl_3$
150MHz

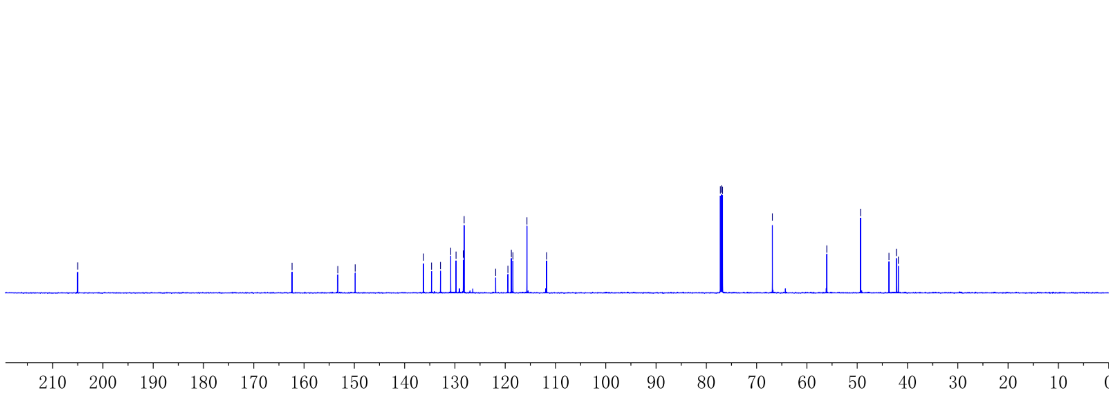

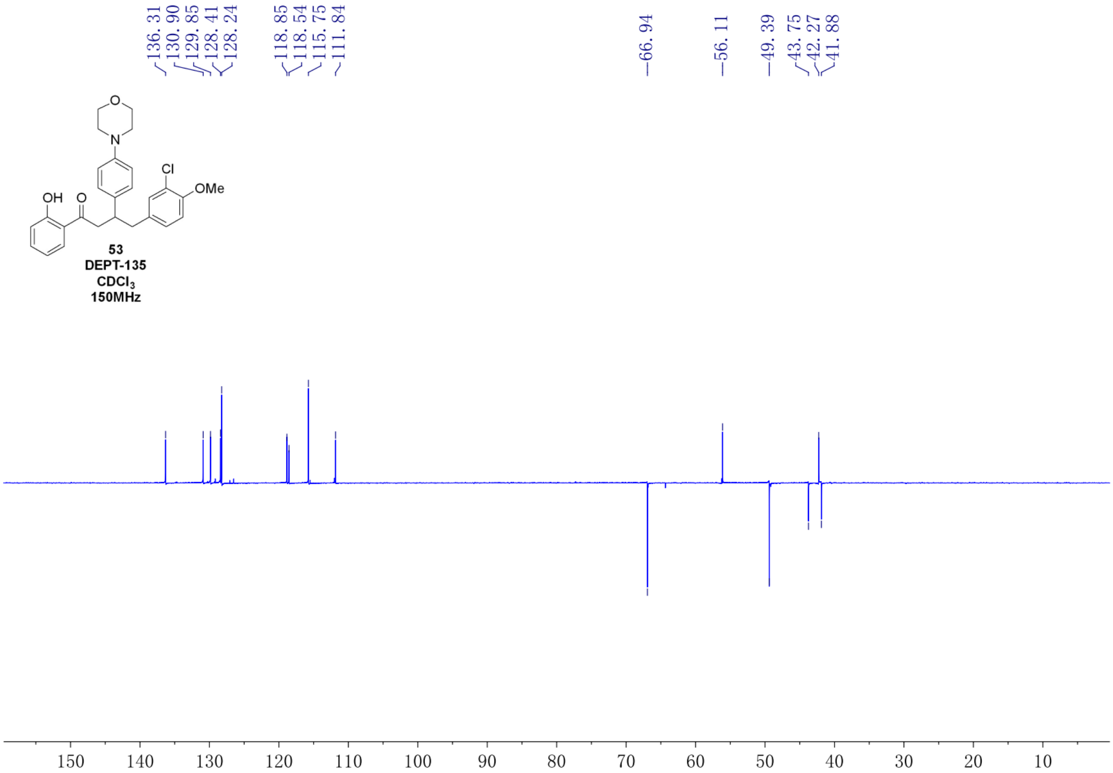
136.31
130.90
129.85
128.41
128.24
118.85
118.54
115.75
111.84
66.94
56.11
49.39
43.75
42.27
41.88
O
N
Cl
OMe
OH
O
53
DEPT-135
CDCl3
150MHz
150
140
130
120
110
100
90
80
70
60
50
40
30
20
10

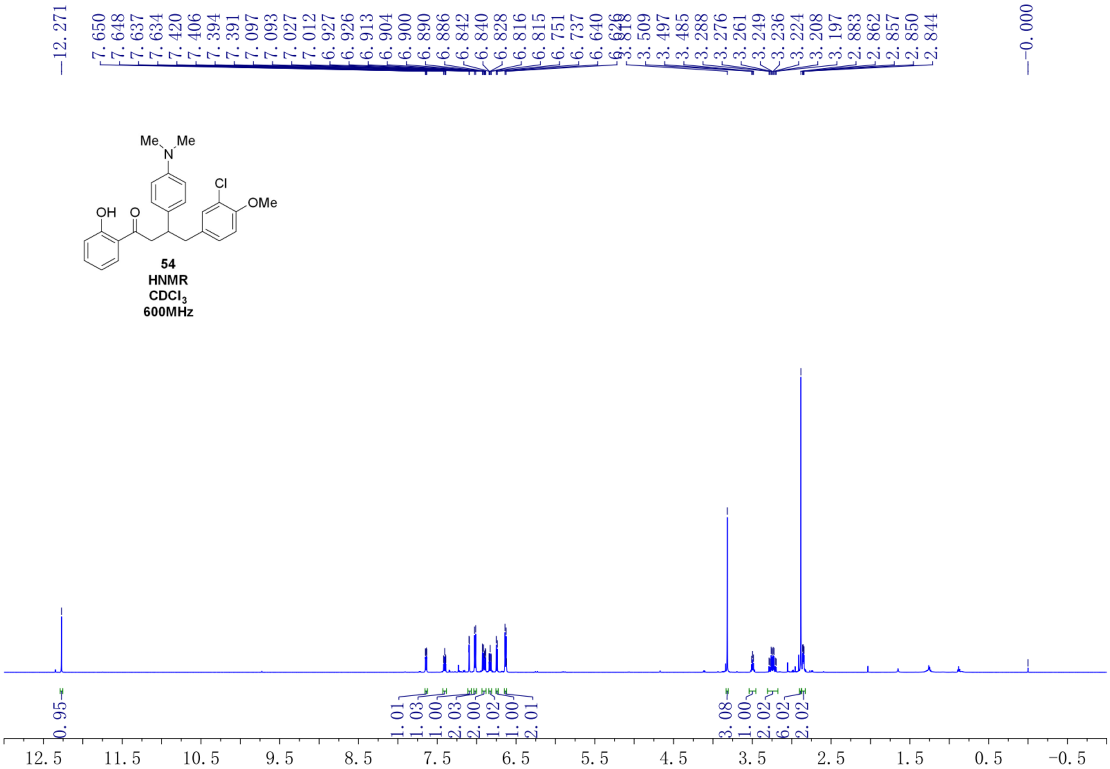
12.271
7.650
7.648
7.637
7.634
7.420
7.406
7.394
7.391
7.097
7.093
7.027
7.012
6.927
6.926
6.913
6.904
6.900
6.890
6.886
6.842
6.840
6.828
6.816
6.815
6.751
6.737
6.640
3.509
3.497
3.485
3.288
3.276
3.261
3.249
3.236
3.224
3.208
3.197
2.883
2.862
2.857
2.850
2.844
-0.000
Me
N
Me
Cl
OMe
OH
O
54
HNMR
CDCl3
600MHz
0.95
1.01
1.03
1.00
2.03
2.00
1.02
1.00
2.01
3.08
1.00
2.02
6.02
2.02
12.5
11.5
10.5
9.5
8.5
7.5
6.5
5.5
4.5
3.5
2.5
1.5
0.5
-0.5

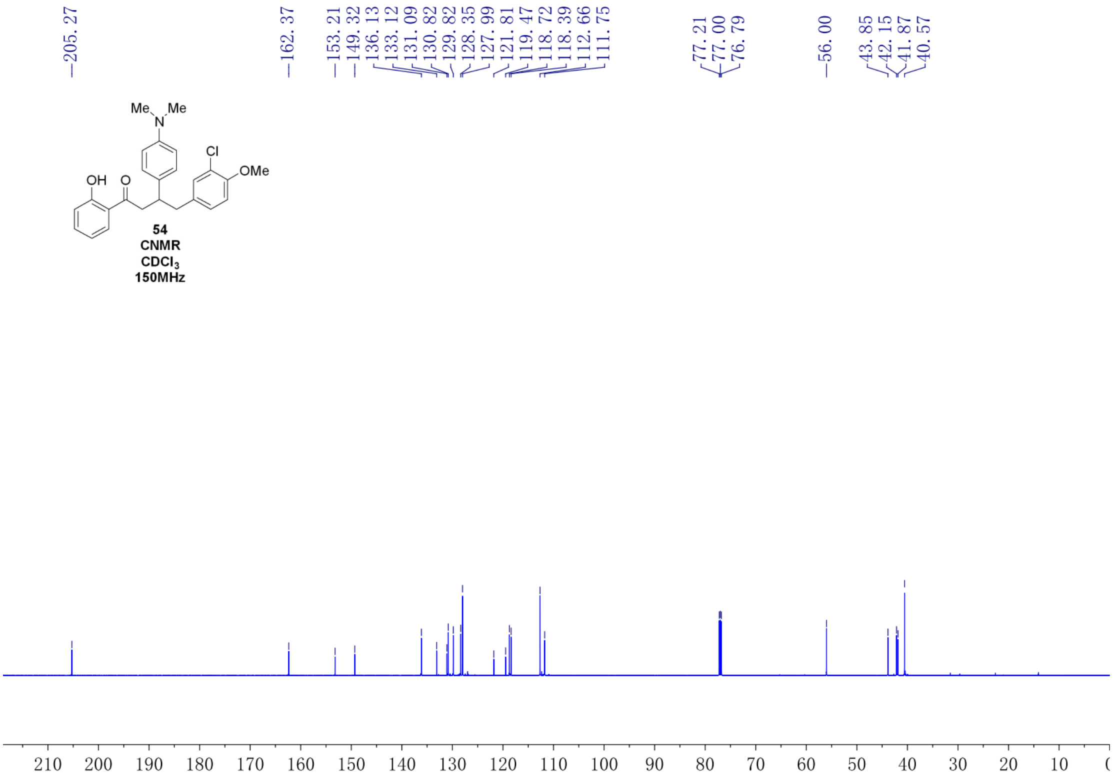
205.27
162.37
153.21
149.32
136.13
133.12
131.09
130.82
129.82
128.35
127.99
121.81
119.47
118.72
118.39
112.66
111.75
77.21
77.00
76.79
56.00
43.85
42.15
41.87
40.57
Me
N
Me
Cl
OMe
OH
O
54
CNMR
$CDCl_3$
150MHz
210 200 190 180 170 160 150 140 130 120 110 100 90 80 70 60 50 40 30 20 10

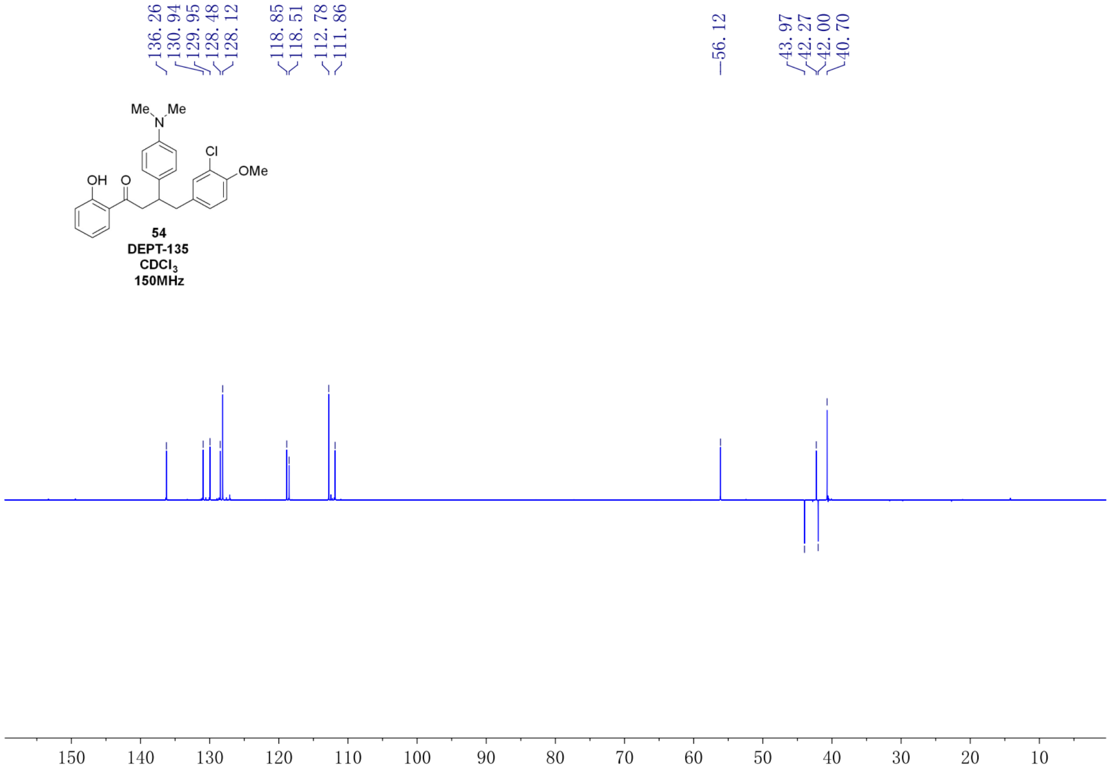
136.26
130.94
129.95
128.48
128.12
118.85
118.51
112.78
111.86
56.12
43.97
42.27
42.00
40.70
Me
N
Me
Cl
OMe
OH
O
54
DEPT-135
$CDCl_3$
150MHz
150 140 130 120 110 100 90 80 70 60 50 40 30 20 10

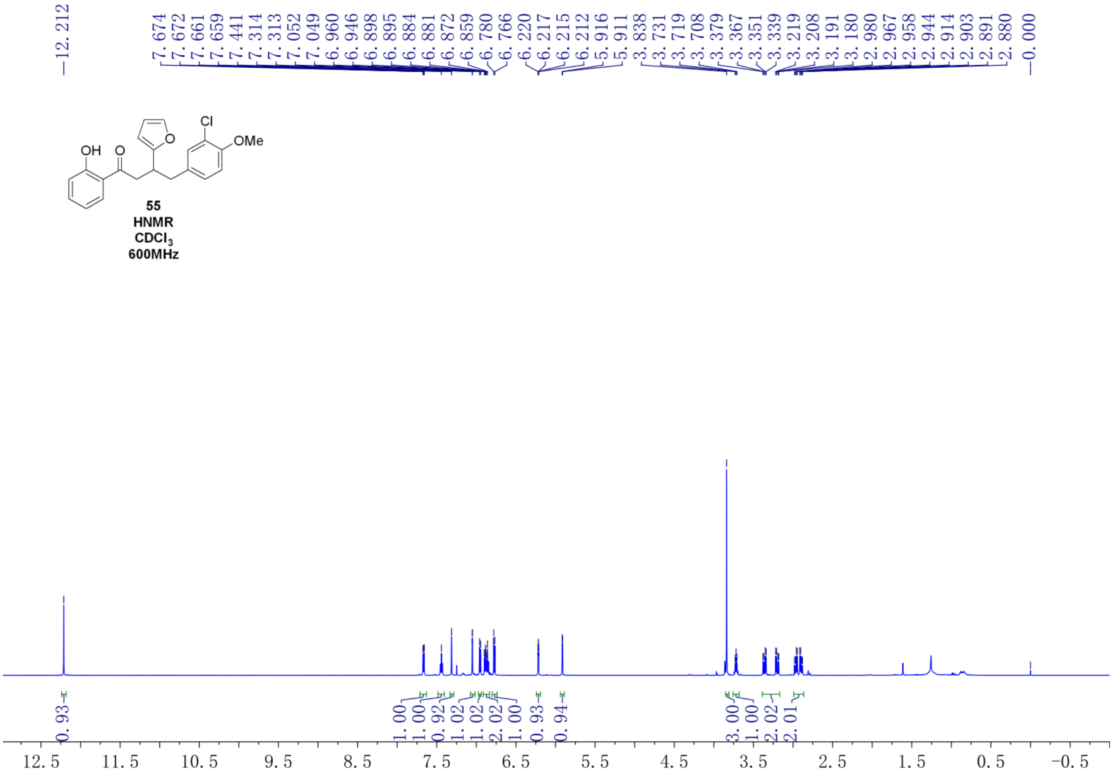
12.212
7.674 7.672 7.661 7.659 7.441 7.314 7.313 7.052 7.049 6.960 6.946 6.898 6.895 6.884 6.881 6.872 6.859 6.780 6.766 6.220 6.217 6.215 6.212 5.916 5.911 3.838 3.731 3.719 3.708 3.379 3.367 3.351 3.339 3.219 3.208 3.191 3.180 2.980 2.967 2.958 2.944 2.914 2.903 2.891 2.880 0.000
OH
O
Cl
OMe
55
HNMR
$CDCl_3$
600MHz
0.93
1.00 1.00 0.92 1.02 1.02 2.02 1.00 0.93 0.94
3.00 1.00 2.02 2.01
12.5 11.5 10.5 9.5 8.5 7.5 6.5 5.5 4.5 3.5 2.5 1.5 0.5 -0.5

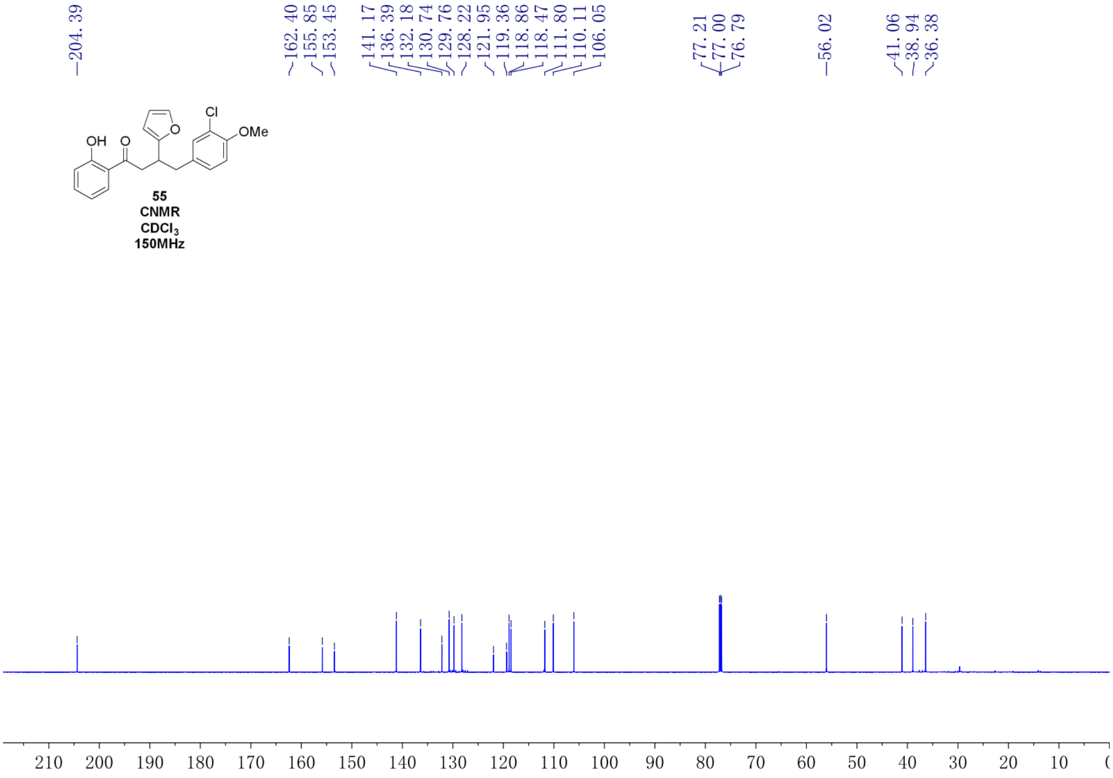
204.39
162.40 155.85 153.45
141.17 136.39 132.18 130.74 129.76 128.22 121.95 119.36 118.86 118.47 111.80 110.11 106.05
77.21 77.00 76.79
56.02
41.06 38.94 36.38
OH
O
Cl
OMe
55
CNMR
$CDCl_3$
150MHz
210 200 190 180 170 160 150 140 130 120 110 100 90 80 70 60 50 40 30 20 10 0

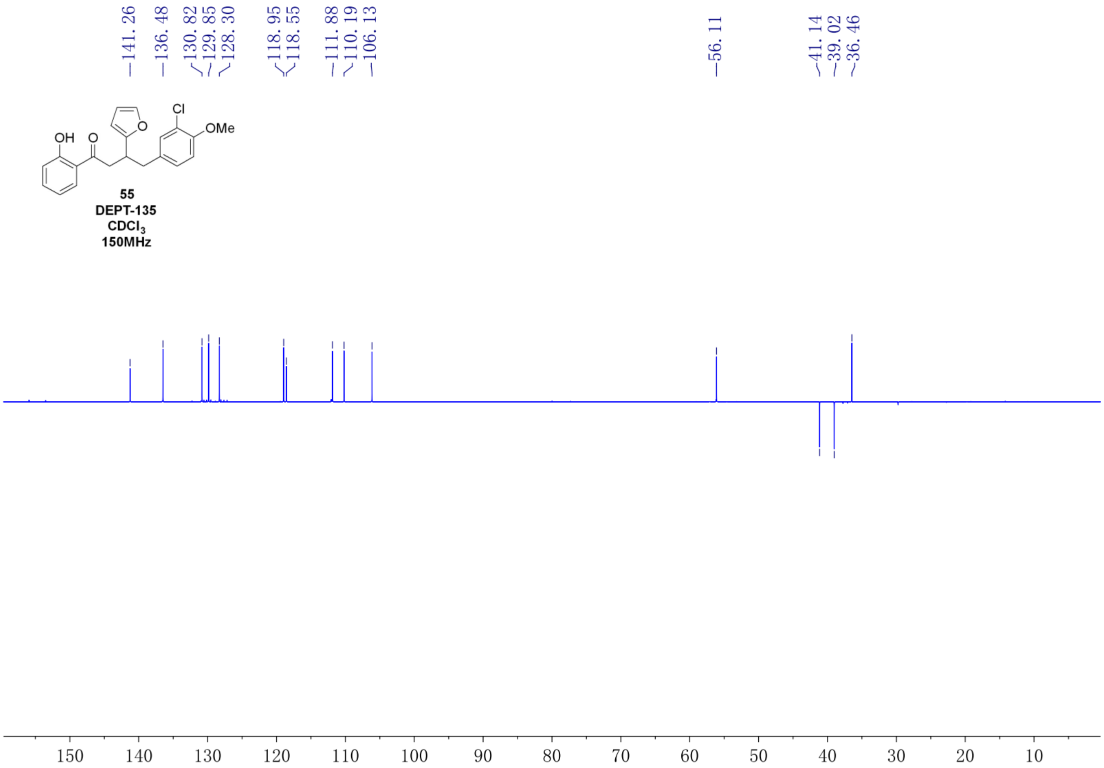
141.26
136.48
130.82
129.85
128.30
118.95
118.55
111.88
110.19
106.13
56.11
41.14
39.02
36.46
OH
O
O
Cl
OMe
55
DEPT-135
$CDCl_3$
150MHz
150 140 130 120 110 100 90 80 70 60 50 40 30 20 10

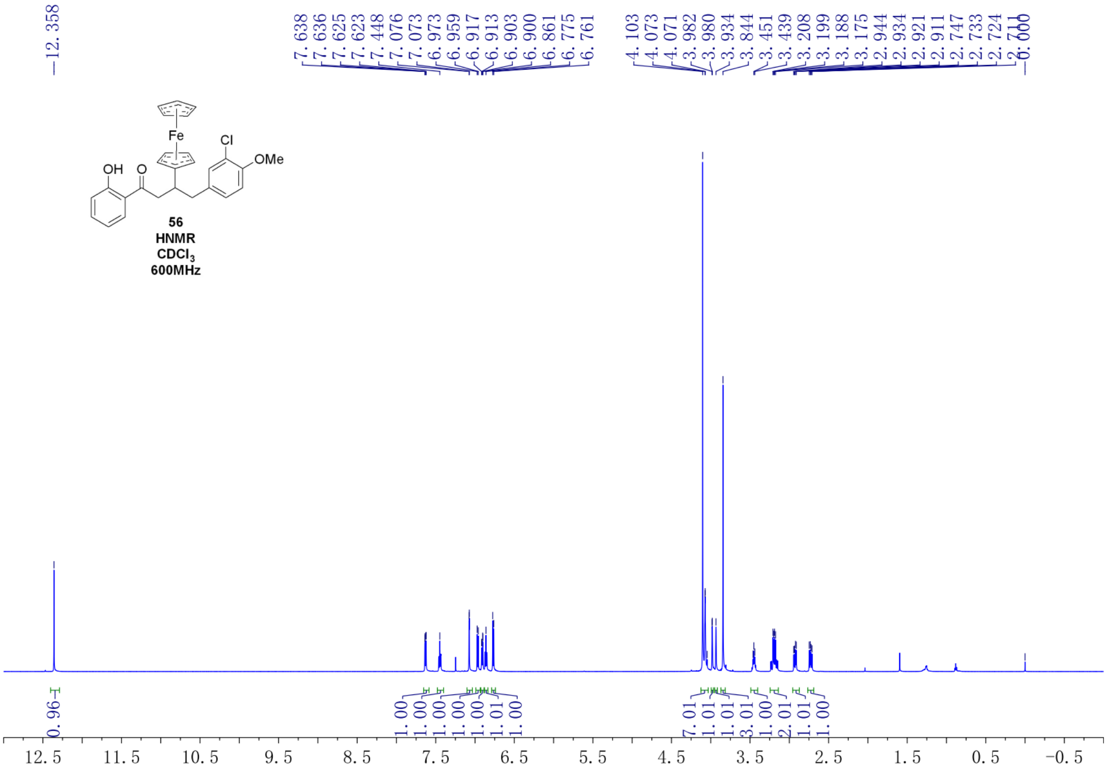
12.358
7.638
7.636
7.625
7.623
7.448
7.076
7.073
6.973
6.959
6.917
6.913
6.903
6.900
6.861
6.775
6.761
4.103
4.073
4.071
3.982
3.980
3.934
3.844
3.451
3.439
3.208
3.199
3.188
3.175
2.944
2.934
2.921
2.911
2.747
2.733
2.724
2.711
0.000
Fe
Cl
OH
O
OMe
56
HNMR
$CDCl_3$
600MHz
0.96
1.00
1.00
1.00
1.00
1.01
1.00
7.01
1.01
1.01
3.01
1.00
2.01
1.01
1.00
12.5 11.5 10.5 9.5 8.5 7.5 6.5 5.5 4.5 3.5 2.5 1.5 0.5 -0.5

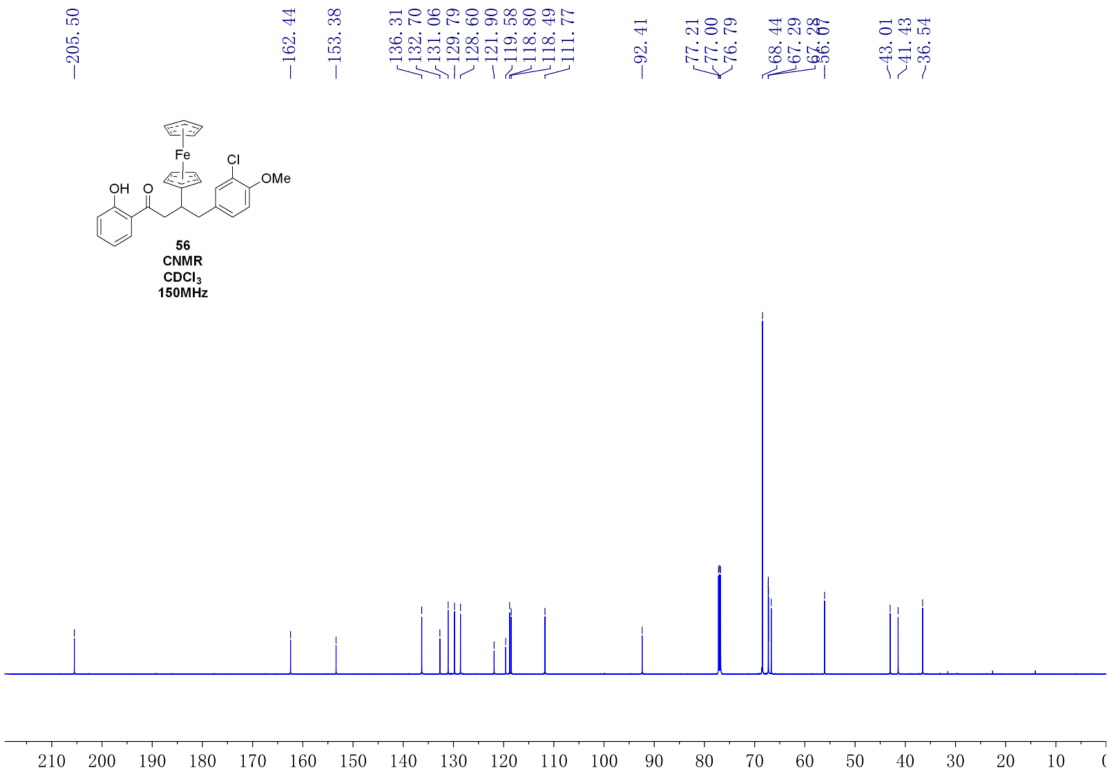
205.50
162.44
153.38
136.31
132.70
131.06
129.79
128.60
121.90
119.58
118.80
118.49
111.77
92.41
77.21
77.00
76.79
68.44
67.29
56.07
43.01
41.43
36.54
Fe
Cl
OMe
OH
O
56
CNMR
CDCl3
150MHz
210 200 190 180 170 160 150 140 130 120 110 100 90 80 70 60 50 40 30 20 10

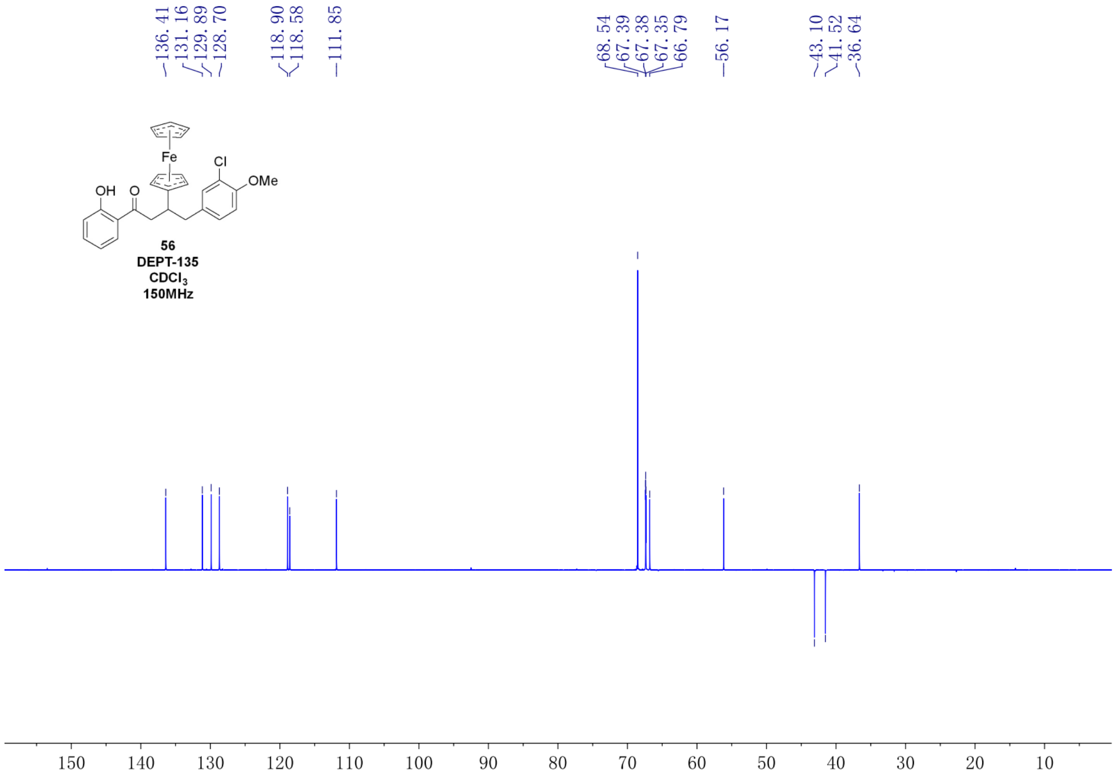
136.41
131.16
129.89
128.70
118.90
118.58
111.85
68.54
67.39
67.38
67.35
66.79
56.17
43.10
41.52
36.64
Fe
Cl
OMe
OH
O
56
DEPT-135
CDCl3
150MHz
150 140 130 120 110 100 90 80 70 60 50 40 30 20 10

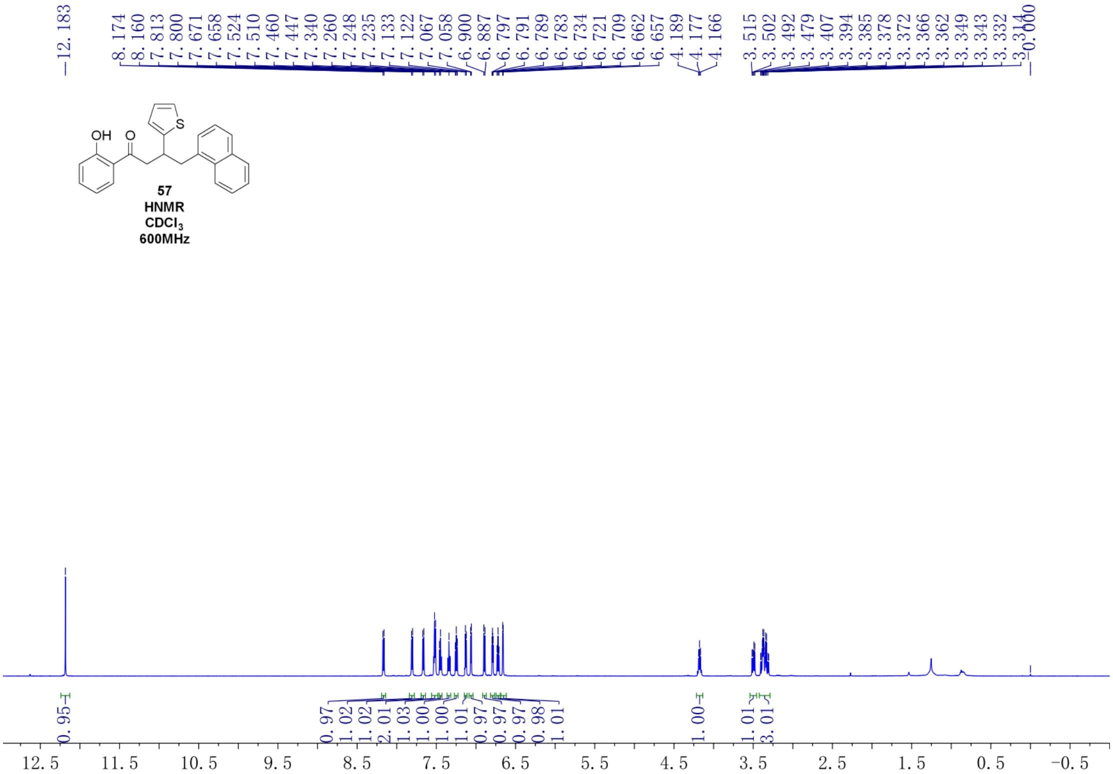
57
HNMR
CDCl3
600MHz
OH
O
S
12.183
8.174
8.160
7.813
7.800
7.671
7.658
7.524
7.510
7.460
7.447
7.340
7.260
7.248
7.235
7.133
7.122
7.067
7.058
6.900
6.887
6.797
6.791
6.789
6.783
6.734
6.721
6.709
6.662
6.657
4.189
4.177
4.166
3.515
3.502
3.492
3.479
3.407
3.394
3.385
3.378
3.372
3.366
3.362
3.349
3.343
3.332
3.314
0.000
0.95
0.97
1.02
1.02
2.01
1.03
1.00
1.00
1.01
0.97
0.97
0.97
0.98
1.01
1.00
1.01
3.01
12.5
11.5
10.5
9.5
8.5
7.5
6.5
5.5
4.5
3.5
2.5
1.5
0.5
-0.5

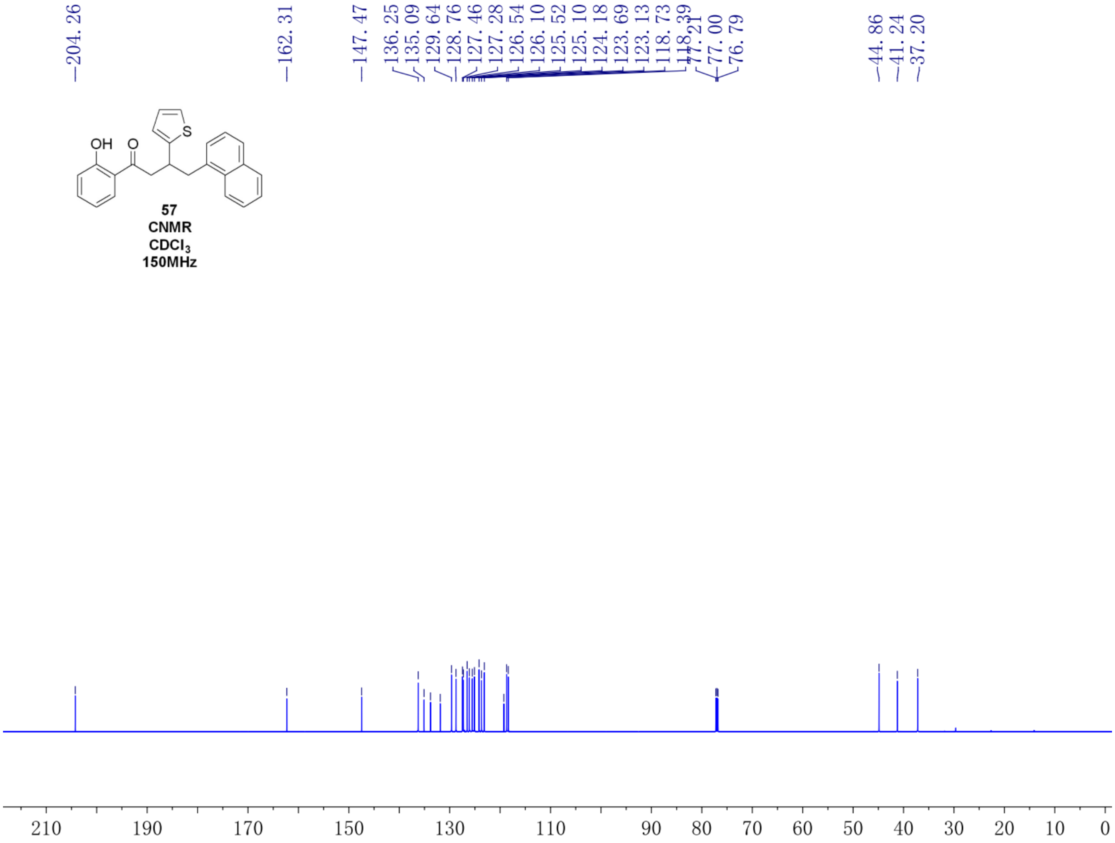
57
CNMR
CDCl3
150MHz
OH
O
S
204.26
162.31
147.47
136.25
135.09
129.64
128.76
127.46
127.28
126.54
126.10
125.52
125.10
124.18
123.69
123.13
118.73
118.39
77.21
77.00
76.79
44.86
41.24
37.20
210
190
170
150
130
110
90
80
70
60
50
40
30
20
10
0

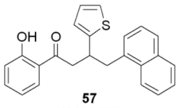

57
DEPT-135
$CDCl_3$
150MHz

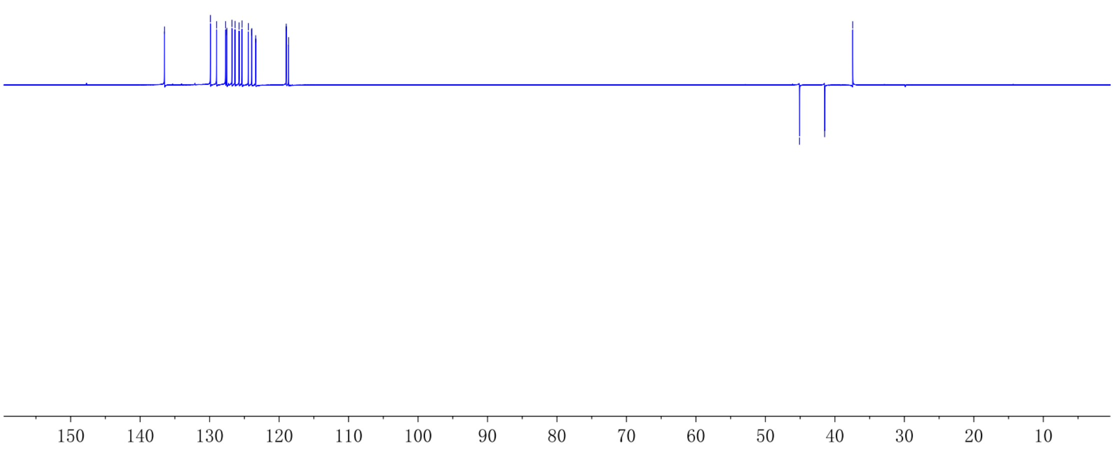

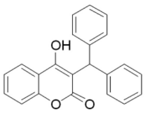

58
HNMR
$CDCl_3$
600MHz

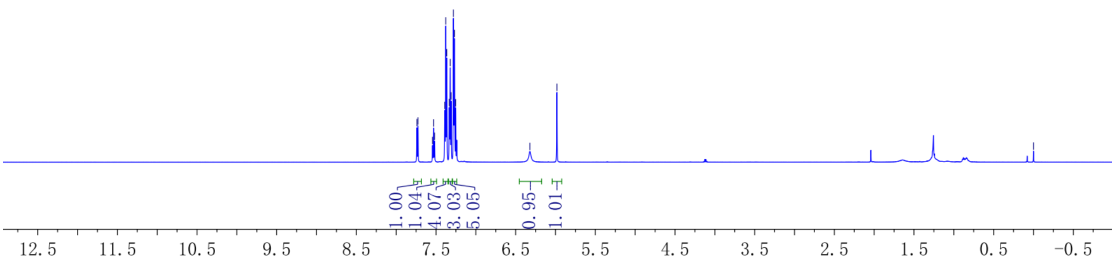

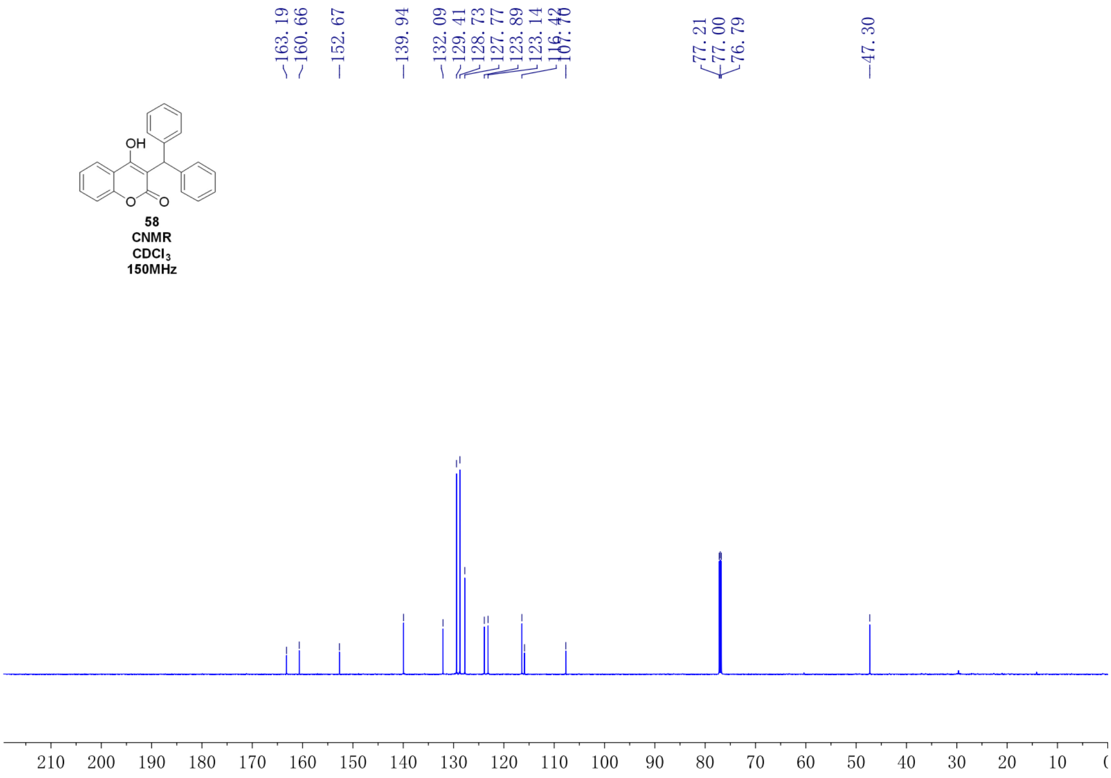
163.19
160.66
152.67
139.94
132.09
129.41
128.73
127.77
123.89
123.14
116.42
107.70
77.21
77.00
76.79
47.30
OH
O
O
58
CNMR
$CDCl_3$
150MHz
210 200 190 180 170 160 150 140 130 120 110 100 90 80 70 60 50 40 30 20 10

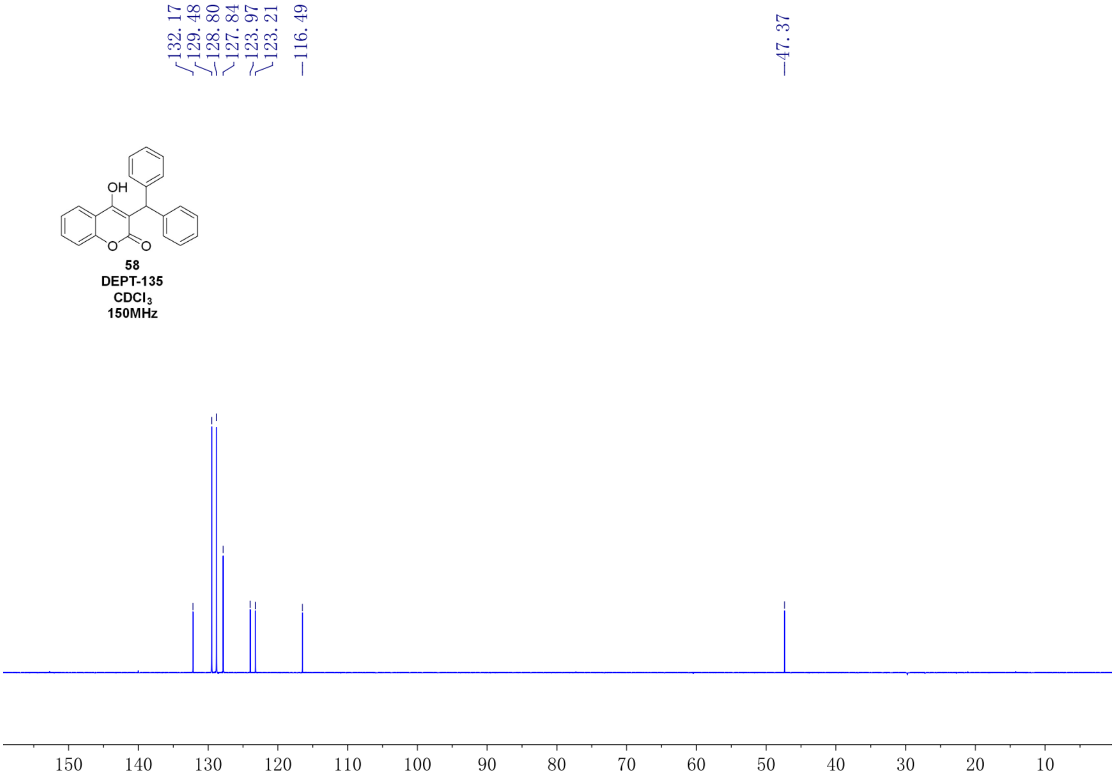
132.17
129.48
128.80
127.84
123.97
123.21
116.49
47.37
OH
O
O
58
DEPT-135
$CDCl_3$
150MHz
150 140 130 120 110 100 90 80 70 60 50 40 30 20 10

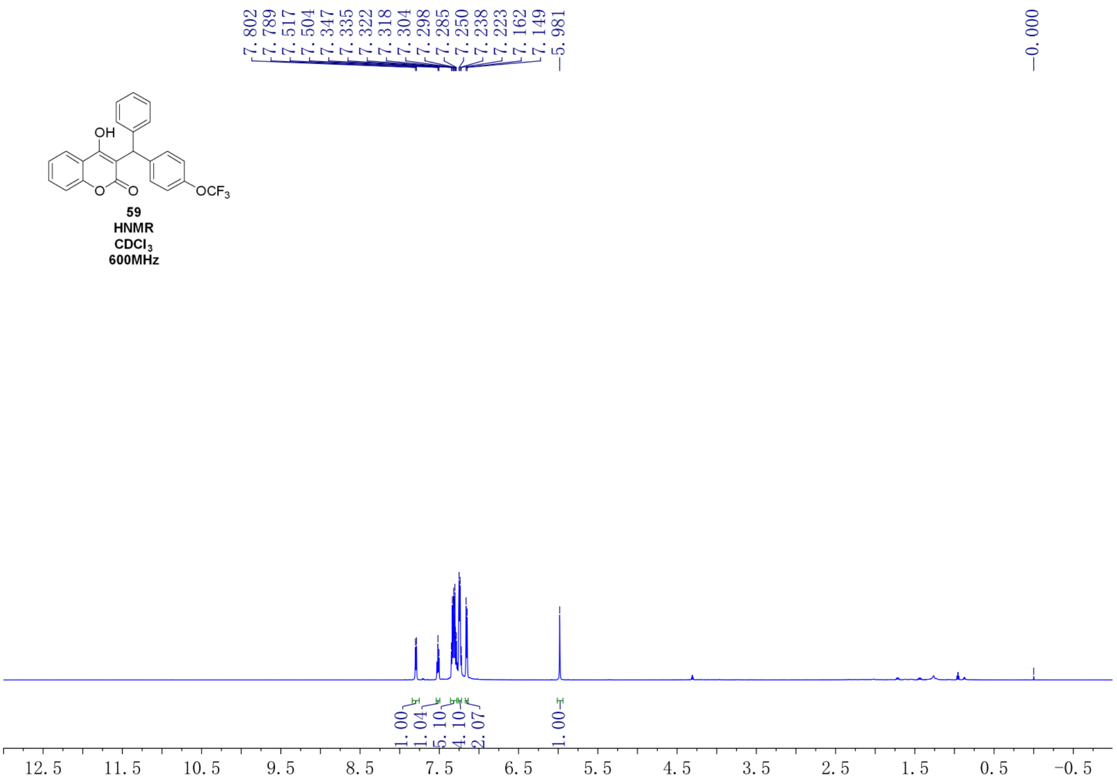
7.802
7.789
7.517
7.504
7.347
7.335
7.322
7.318
7.304
7.298
7.285
7.250
7.238
7.223
7.162
7.149
5.981
0.000
OH
OCF3
59
HNMR
CDCl3
600MHz
1.00
1.04
5.10
4.10
2.07
1.00
12.5
11.5
10.5
9.5
8.5
7.5
6.5
5.5
4.5
3.5
2.5
1.5
0.5
-0.5

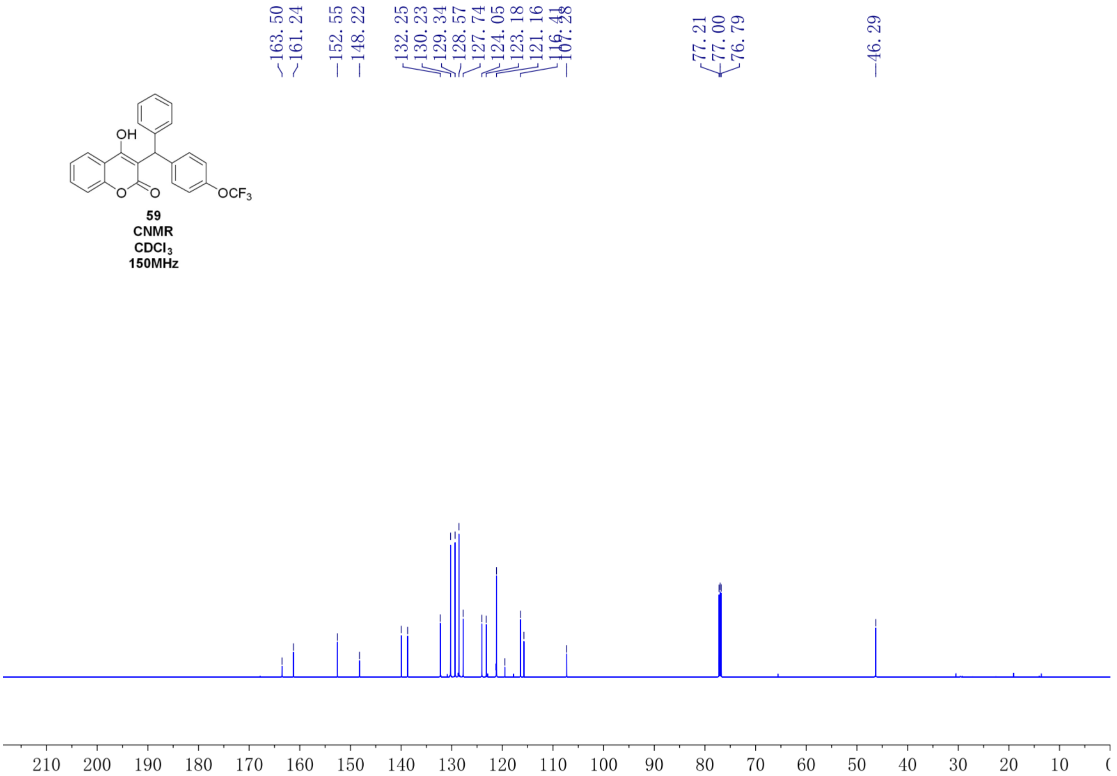
163.50
161.24
152.55
148.22
132.25
130.23
129.34
128.57
127.74
124.05
123.18
121.16
116.41
107.28
77.21
77.00
76.79
46.29
OH
OCF3
59
CNMR
CDCl3
150MHz
210
200
190
180
170
160
150
140
130
120
110
100
90
80
70
60
50
40
30
20
10

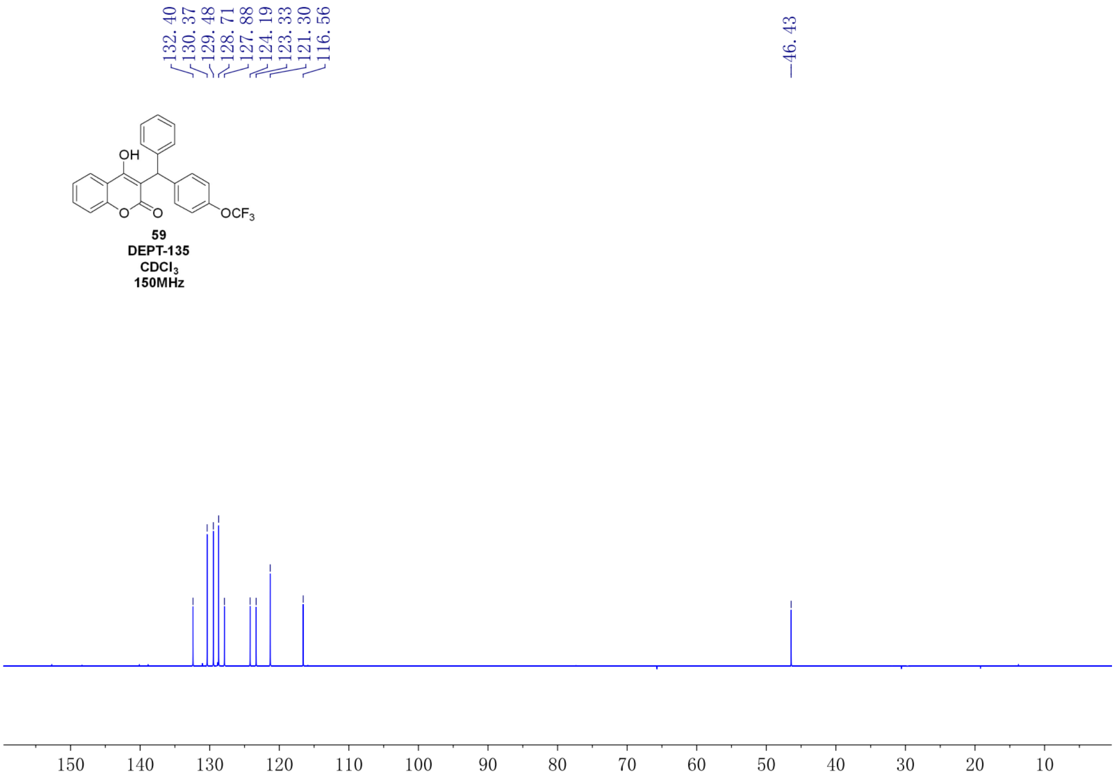

132.40
130.37
129.48
128.71
127.88
124.19
123.33
121.30
116.56
46.43
OH
O
O
OCF3
59
DEPT-135
CDCl3
150MHz
150
140
130
120
110
100
90
80
70
60
50
40
30
20
10

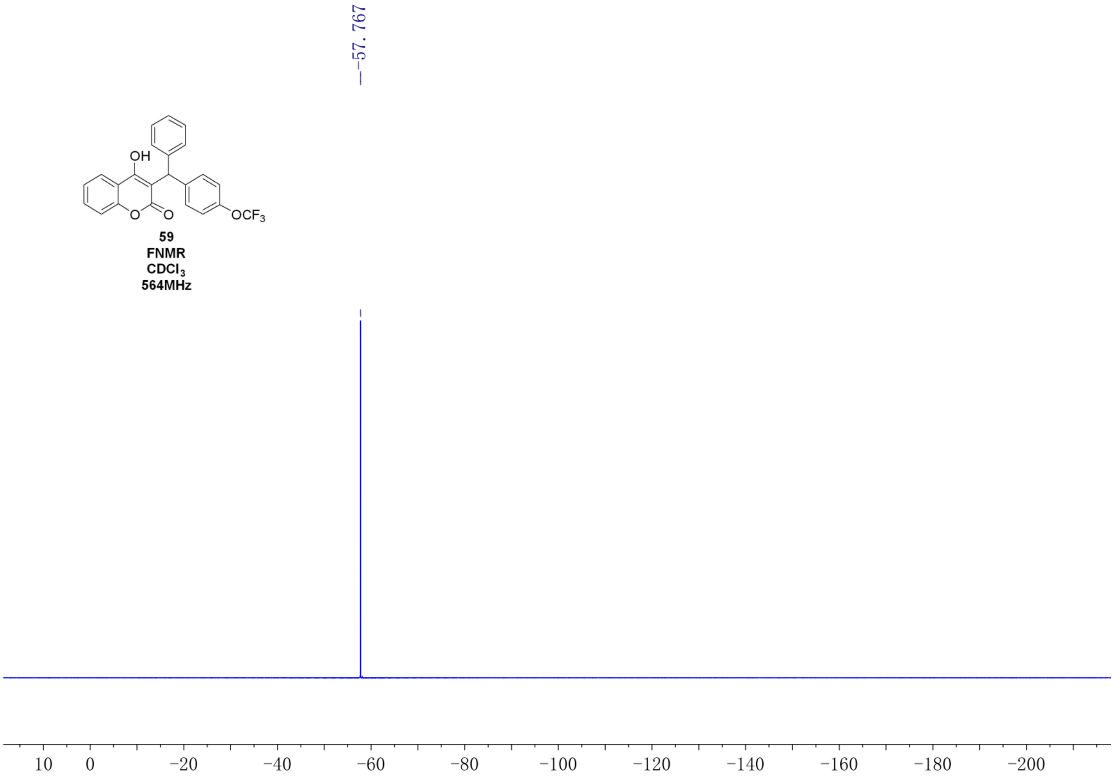

−57.767
OH
O
O
OCF3
59
FNMR
CDCl3
564MHz
10
0
−20
−40
−60
−80
−100
−120
−140
−160
−180
−200

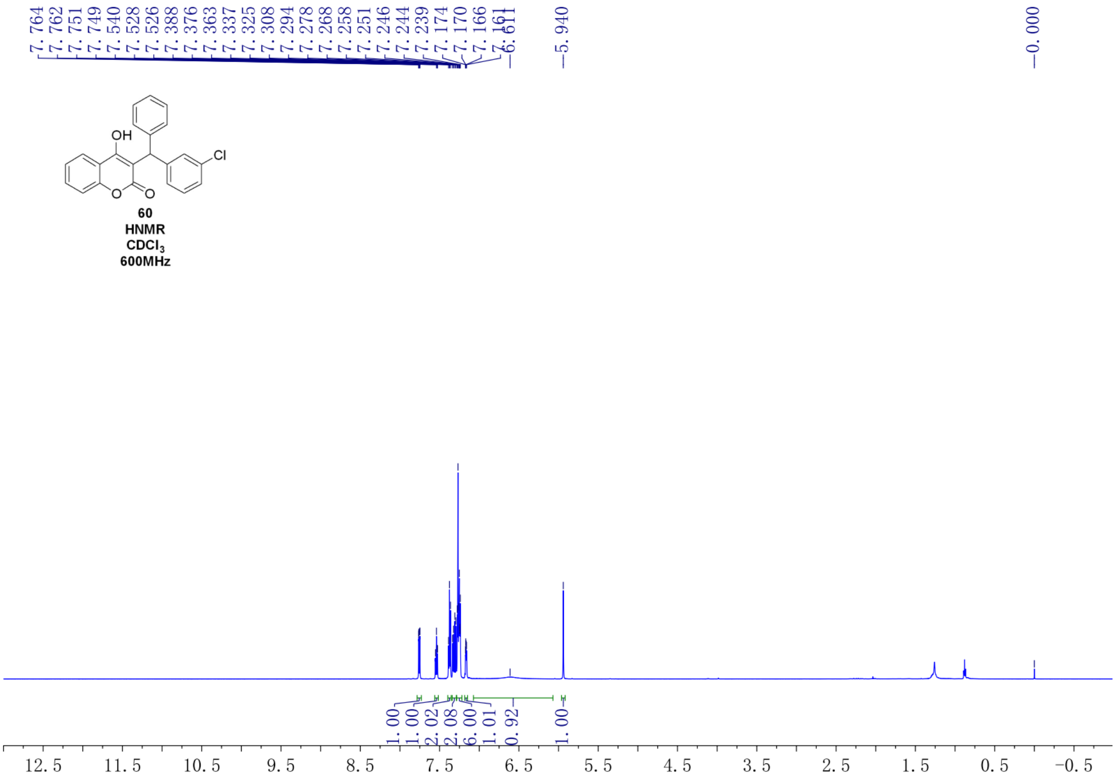
7.764
7.762
7.751
7.749
7.540
7.528
7.526
7.388
7.376
7.363
7.337
7.325
7.308
7.294
7.278
7.268
7.258
7.251
7.246
7.244
7.239
7.174
7.170
7.166
5.940
0.000
OH
Cl
O
O
60
HNMR
CDCl3
600MHz
1.00
1.00
2.02
2.08
6.00
1.01
0.92
1.00
12.5
11.5
10.5
9.5
8.5
7.5
6.5
5.5
4.5
3.5
2.5
1.5
0.5
-0.5

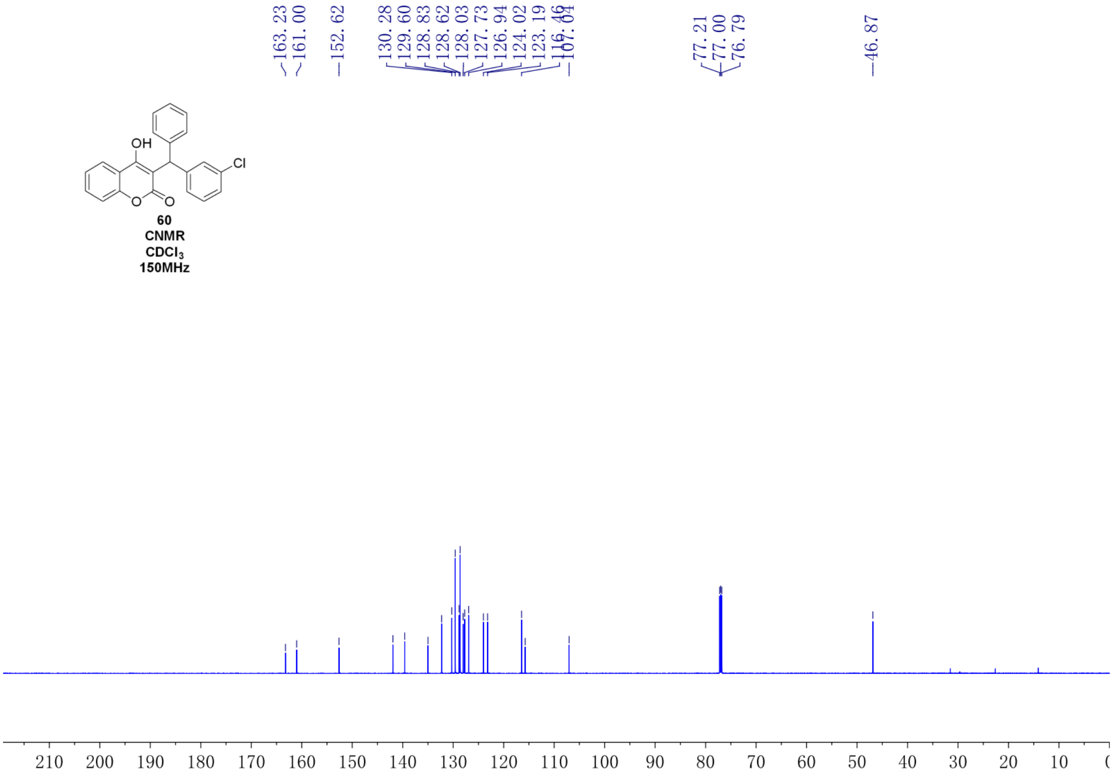
163.23
161.00
152.62
130.28
129.60
128.83
128.62
128.03
127.73
126.94
124.02
123.19
77.21
77.00
76.79
46.87
OH
Cl
O
O
60
CNMR
CDCl3
150MHz
210
200
190
180
170
160
150
140
130
120
110
100
90
80
70
60
50
40
30
20
10

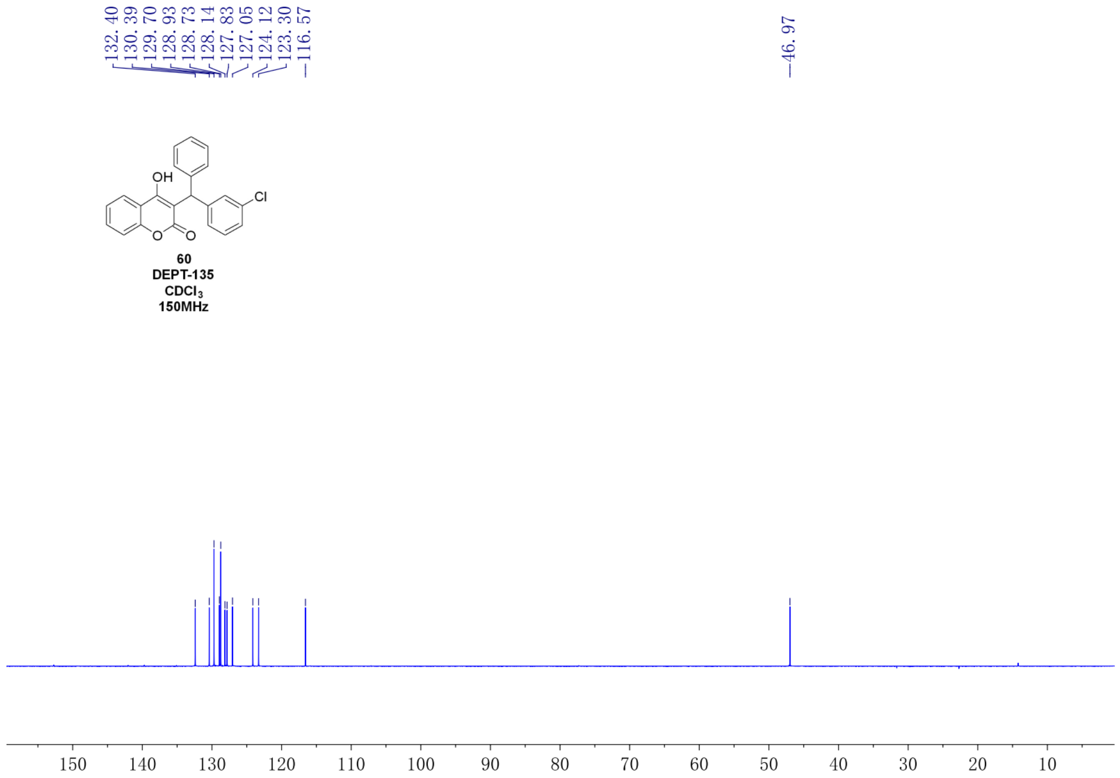
132.40
130.39
129.70
128.93
128.73
128.14
127.83
127.05
124.12
123.30
116.57
46.97
OH
Cl
O
O
60
DEPT-135
$CDCl_3$
150MHz
150
140
130
120
110
100
90
80
70
60
50
40
30
20
10

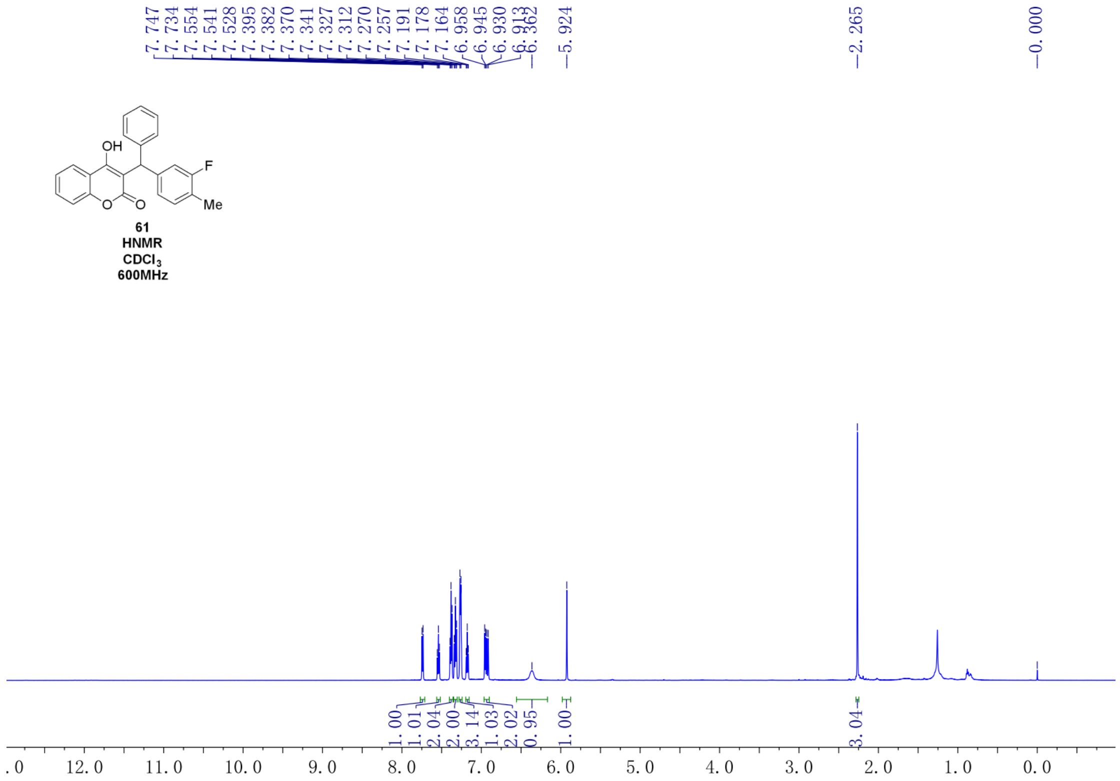
7.747
7.734
7.554
7.541
7.528
7.395
7.382
7.370
7.341
7.327
7.312
7.270
7.257
7.191
7.178
7.164
6.958
6.945
6.930
6.913
6.362
5.924
2.265
0.000
OH
F
Me
O
O
61
HNMR
$CDCl_3$
600MHz
1.00
1.01
2.04
2.00
3.14
1.03
2.02
0.95
1.00
3.04
12.0
11.0
10.0
9.0
8.0
7.0
6.0
5.0
4.0
3.0
2.0
1.0
0.0

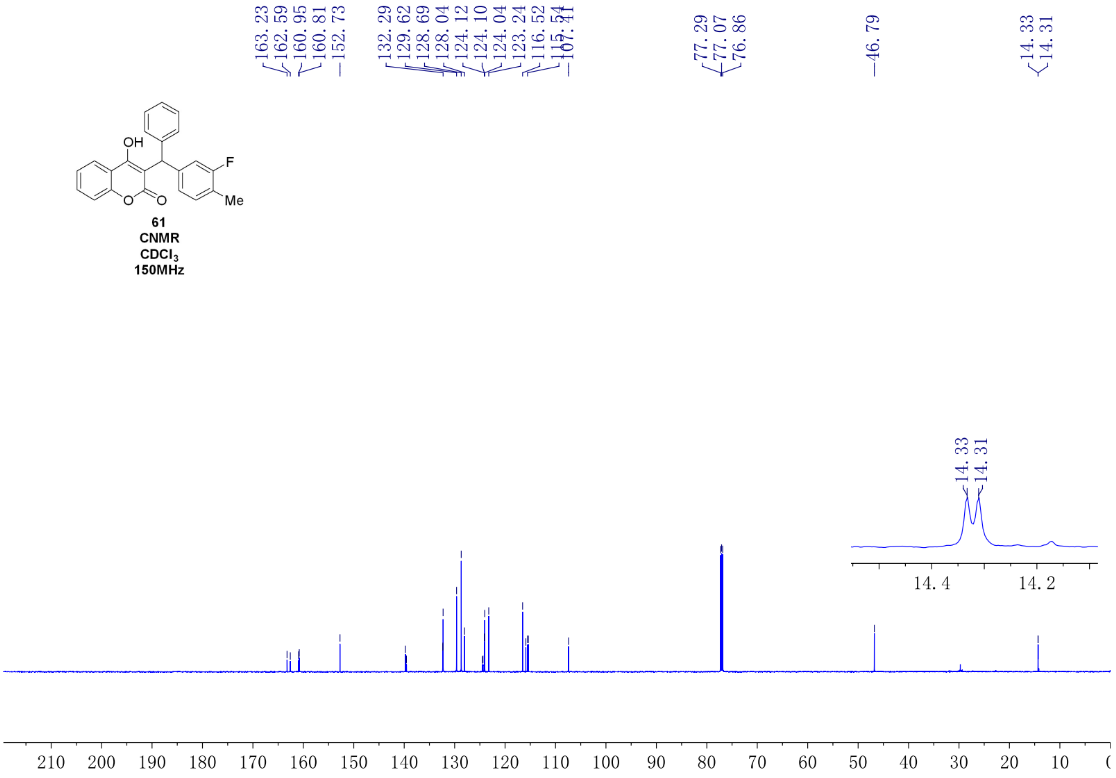
163.23
162.59
160.95
160.81
152.73
132.29
129.62
128.69
128.04
124.12
124.10
124.04
123.24
116.52
115.54
107.41
77.29
77.07
76.86
46.79
14.33
14.31
OH
F
O
O
Me
61
CNMR
$CDCl_3$
150MHz
14.33
14.31
14.4
14.2
210 200 190 180 170 160 150 140 130 120 110 100 90 80 70 60 50 40 30 20 10

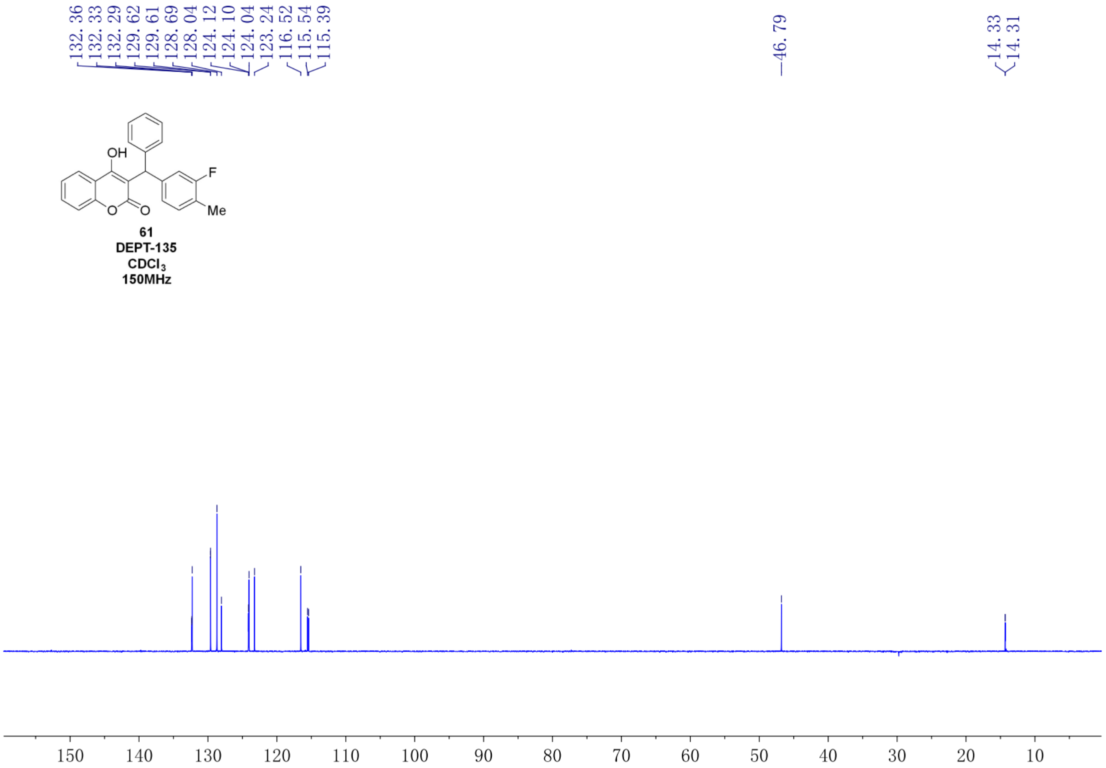
132.36
132.33
132.29
129.62
129.61
128.69
128.04
124.12
124.10
124.04
123.24
116.52
115.54
115.39
46.79
14.33
14.31
OH
F
O
O
Me
61
DEPT-135
$CDCl_3$
150MHz
150 140 130 120 110 100 90 80 70 60 50 40 30 20 10

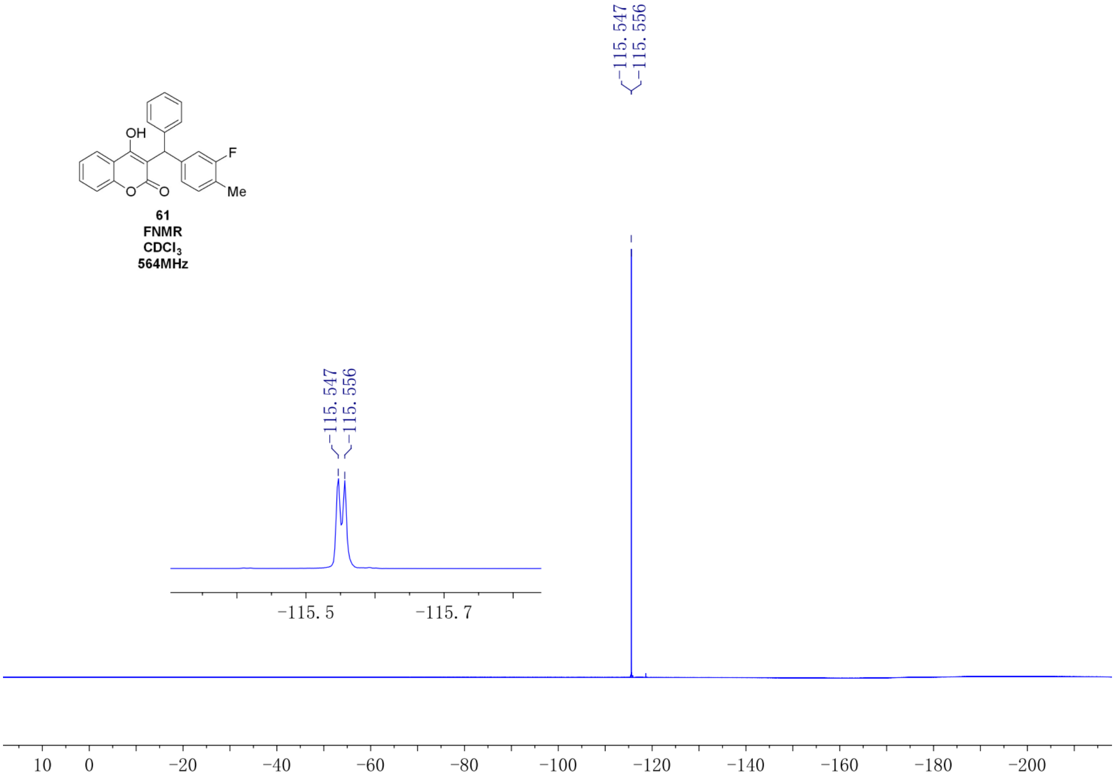

OH
F
Me
O
O
61
FNMR
CDCl3
564MHz
-115.547
-115.556
-115.5
-115.7
10
0
-20
-40
-60
-80
-100
-120
-140
-160
-180
-200

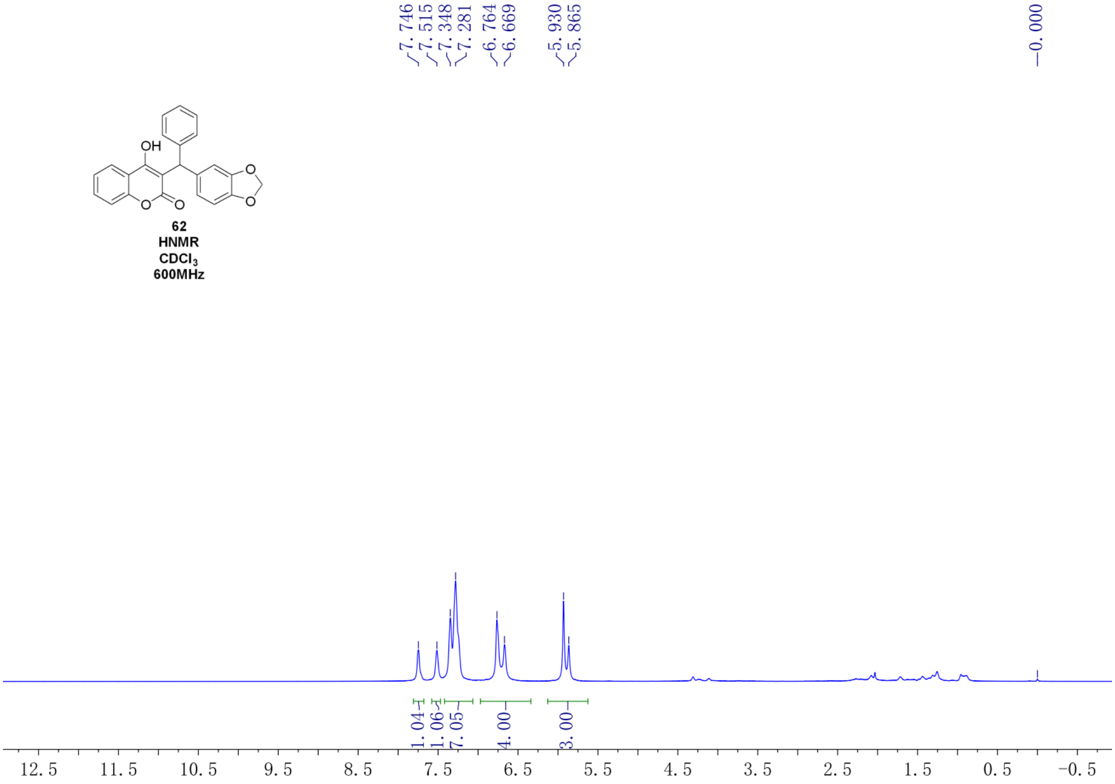

7.746
7.515
7.348
7.281
6.764
6.669
5.930
5.865
0.000
OH
O
O
O
O
62
HNMR
CDCl3
600MHz
1.04
1.06
7.05
4.00
3.00
12.5
11.5
10.5
9.5
8.5
7.5
6.5
5.5
4.5
3.5
2.5
1.5
0.5
-0.5

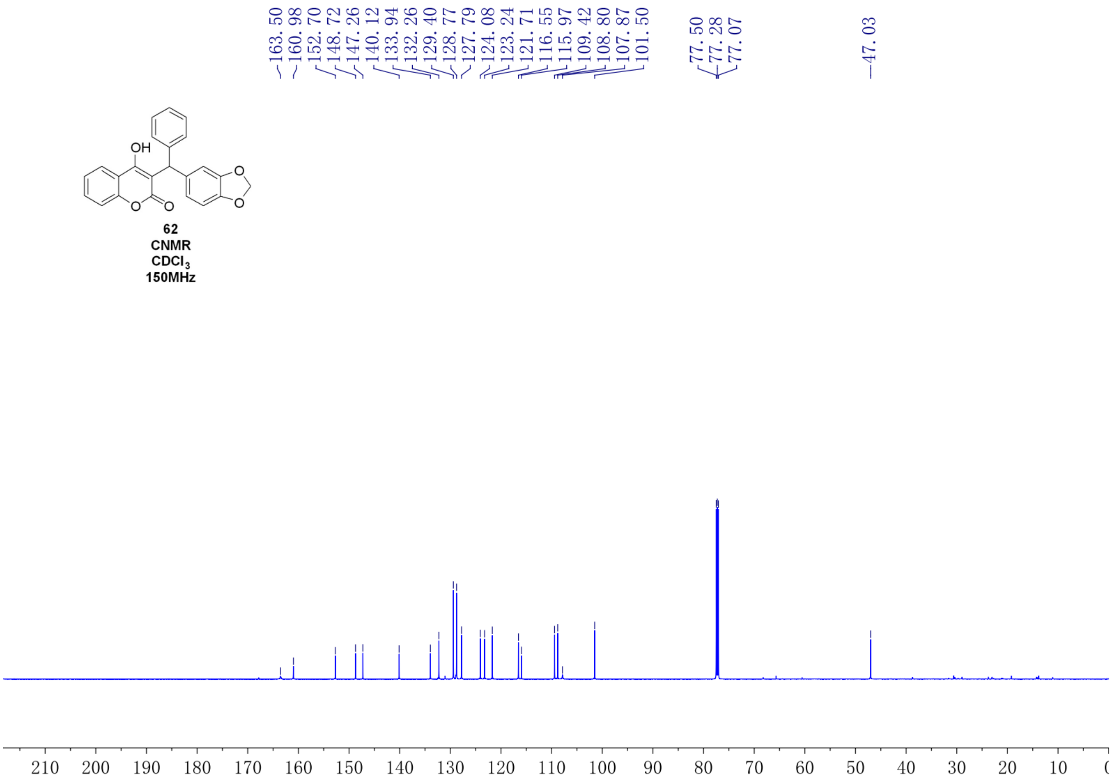
163.50
160.98
152.70
148.72
147.26
140.12
133.94
132.26
129.40
128.77
127.79
124.08
123.24
121.71
116.55
115.97
109.42
108.80
107.87
101.50
77.50
77.28
77.07
47.03
OH
O
O
O
O
62
CNMR
CDCl3
150MHz
210 200 190 180 170 160 150 140 130 120 110 100 90 80 70 60 50 40 30 20 10

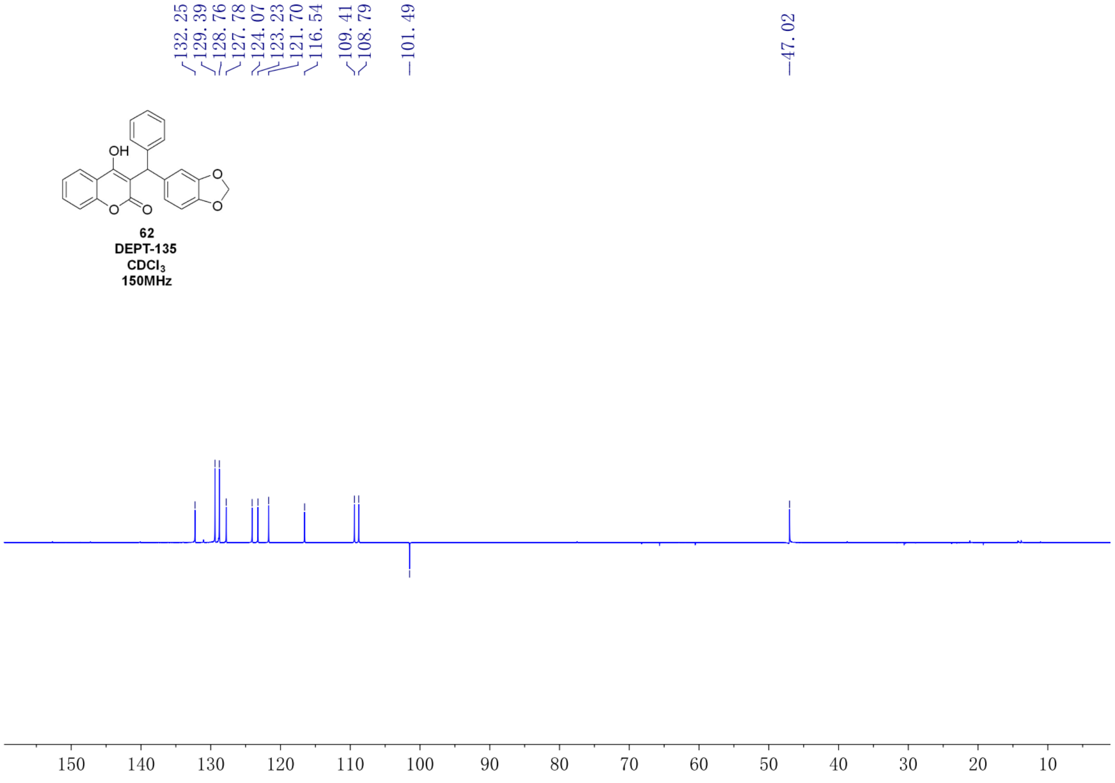
132.25
129.39
128.76
127.78
124.07
123.23
121.70
116.54
109.41
108.79
101.49
47.02
OH
O
O
O
O
62
DEPT-135
CDCl3
150MHz
150 140 130 120 110 100 90 80 70 60 50 40 30 20 10

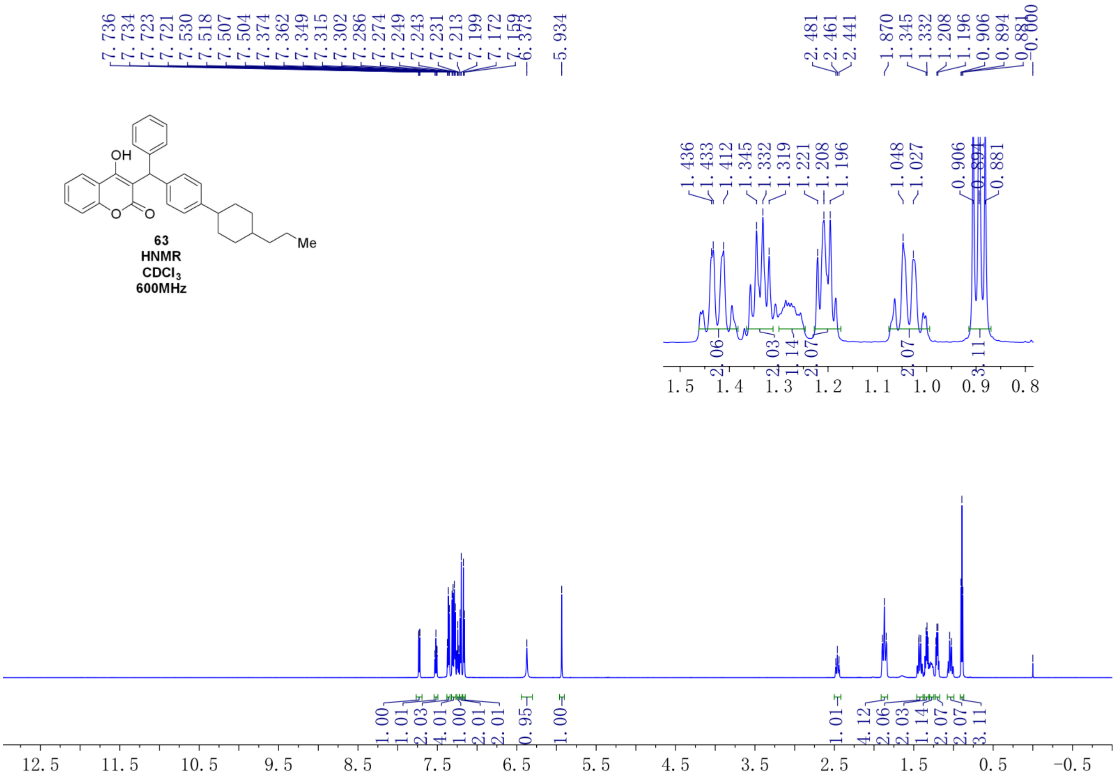
63
HNMR
CDCl3
600MHz
OH
Me
7.736 7.734 7.723 7.721 7.530 7.518 7.507 7.504 7.374 7.362 7.349 7.315 7.302 7.286 7.274 7.249 7.243 7.231 7.213 7.199 7.172 7.159 6.373 5.934
2.481 2.461 2.441 1.870 1.345 1.332 1.208 1.196 0.906 0.894 0.881 0.000
1.436 1.433 1.412 1.345 1.332 1.319 1.221 1.208 1.196 1.048 1.027 0.906 0.894 0.881

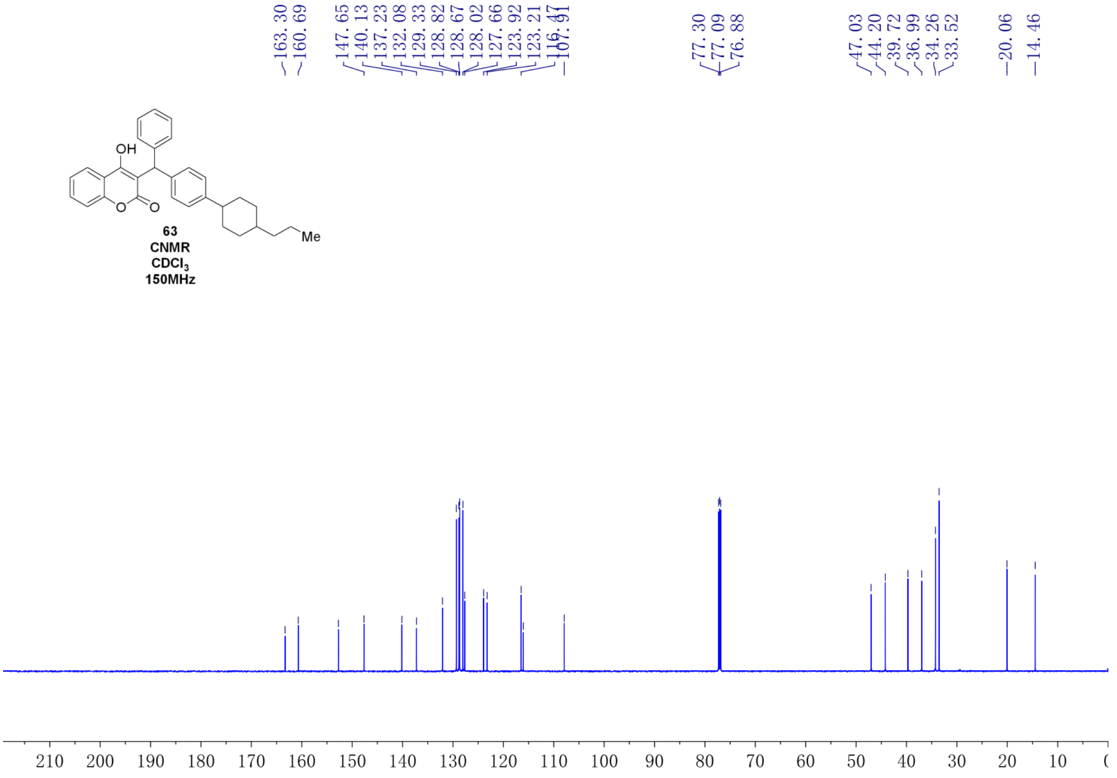
63
CNMR
CDCl3
150MHz
OH
Me
163.30 160.69 147.65 140.13 137.23 132.08 129.33 128.82 128.67 128.02 127.66 123.92 123.21 116.47 107.91
77.30 77.09 76.88
47.03 44.20 39.72 36.99 34.26 33.52 20.06 14.46

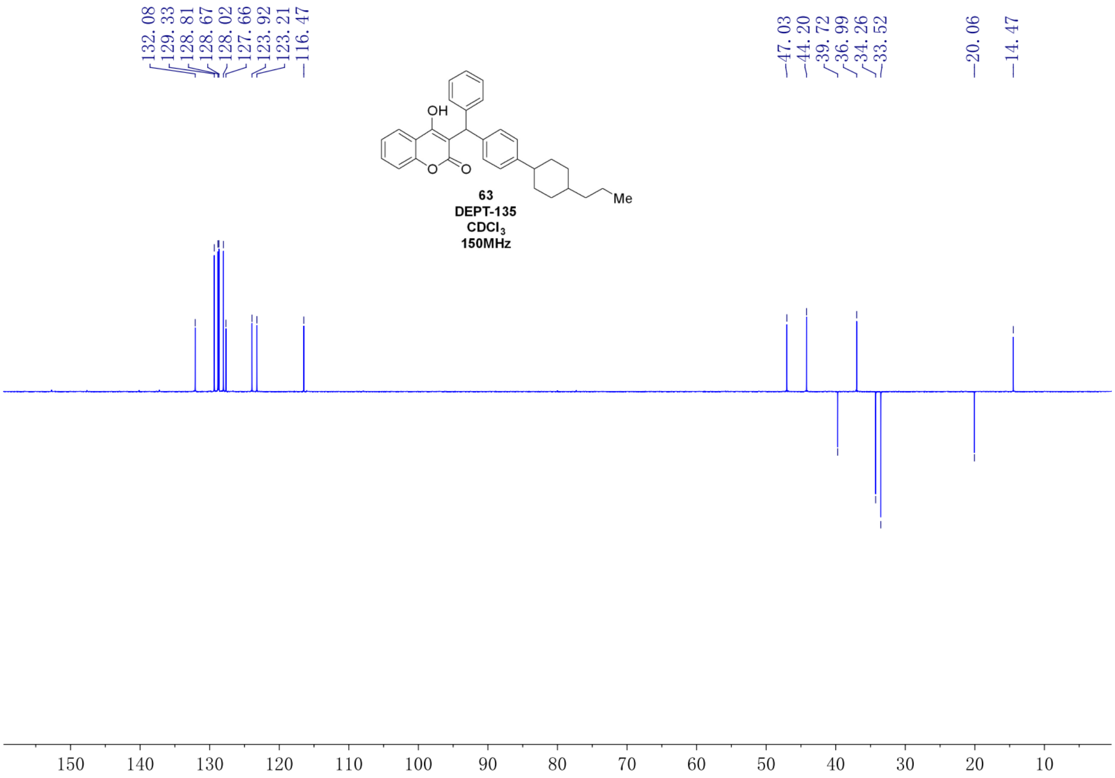
132.08
129.33
128.81
128.67
128.02
127.66
123.92
123.21
116.47
47.03
44.20
39.72
36.99
34.26
33.52
20.06
14.47
OH
O
O
Me
63
DEPT-135
CDCl3
150MHz
150 140 130 120 110 100 90 80 70 60 50 40 30 20 10

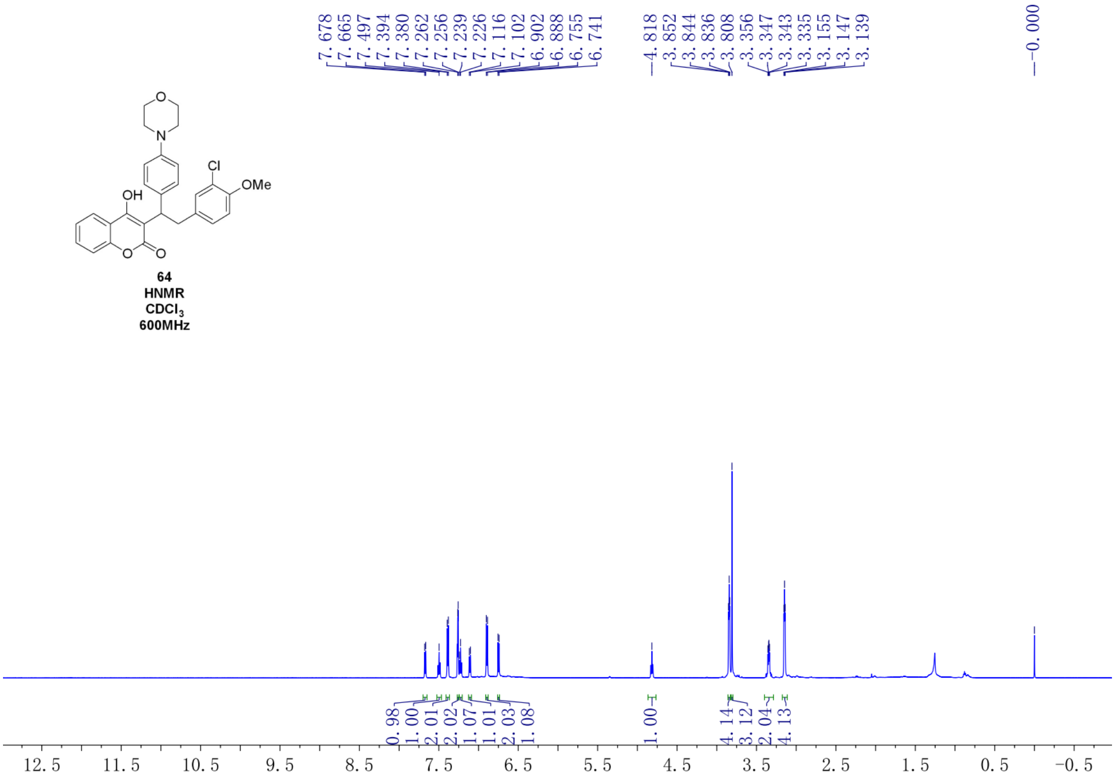
7.678
7.665
7.497
7.394
7.380
7.262
7.256
7.239
7.226
7.116
7.102
6.902
6.888
6.755
6.741
4.818
3.852
3.844
3.836
3.808
3.356
3.347
3.343
3.335
3.155
3.147
3.139
-0.000
O
N
Cl
OMe
OH
O
O
64
HNMR
CDCl3
600MHz
0.98
1.00
2.01
2.02
1.07
1.01
2.03
1.08
1.00
4.14
3.12
2.04
4.13
12.5 11.5 10.5 9.5 8.5 7.5 6.5 5.5 4.5 3.5 2.5 1.5 0.5 -0.5

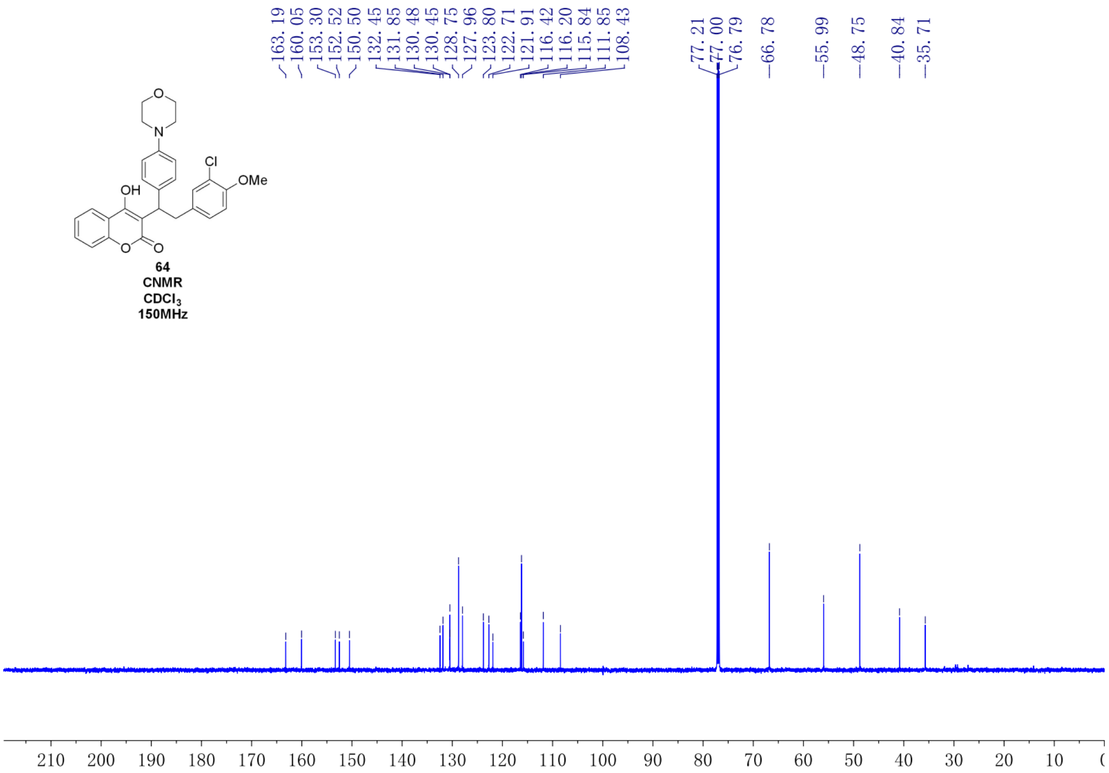
163.19
160.05
153.30
152.52
150.50
132.45
131.85
130.48
130.45
128.75
127.96
123.80
122.71
121.91
116.42
116.20
115.84
111.85
108.43
77.21
77.00
76.79
66.78
55.99
48.75
40.84
35.71
Cl
OMe
OH
O
N
64
CNMR
CDCl3
150MHz
210 200 190 180 170 160 150 140 130 120 110 100 90 80 70 60 50 40 30 20 10

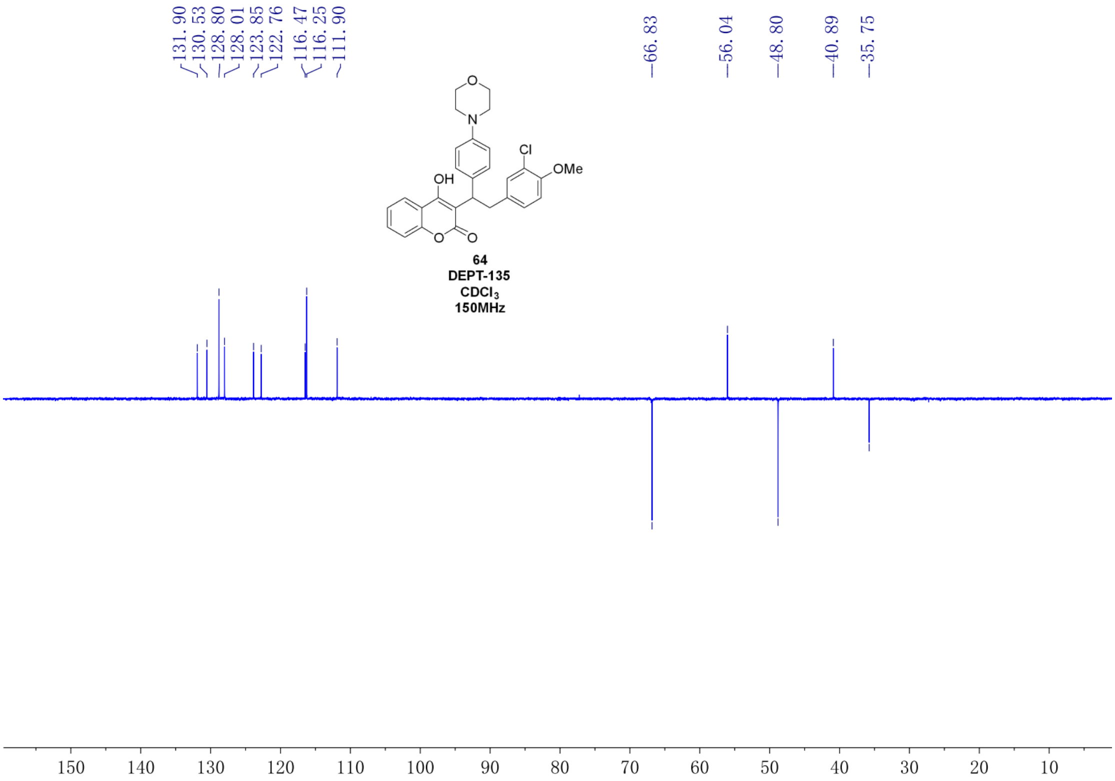
131.90
130.53
128.80
128.01
123.85
122.76
116.47
116.25
111.90
66.83
56.04
48.80
40.89
35.75
Cl
OMe
OH
O
N
64
DEPT-135
CDCl3
150MHz
150 140 130 120 110 100 90 80 70 60 50 40 30 20 10

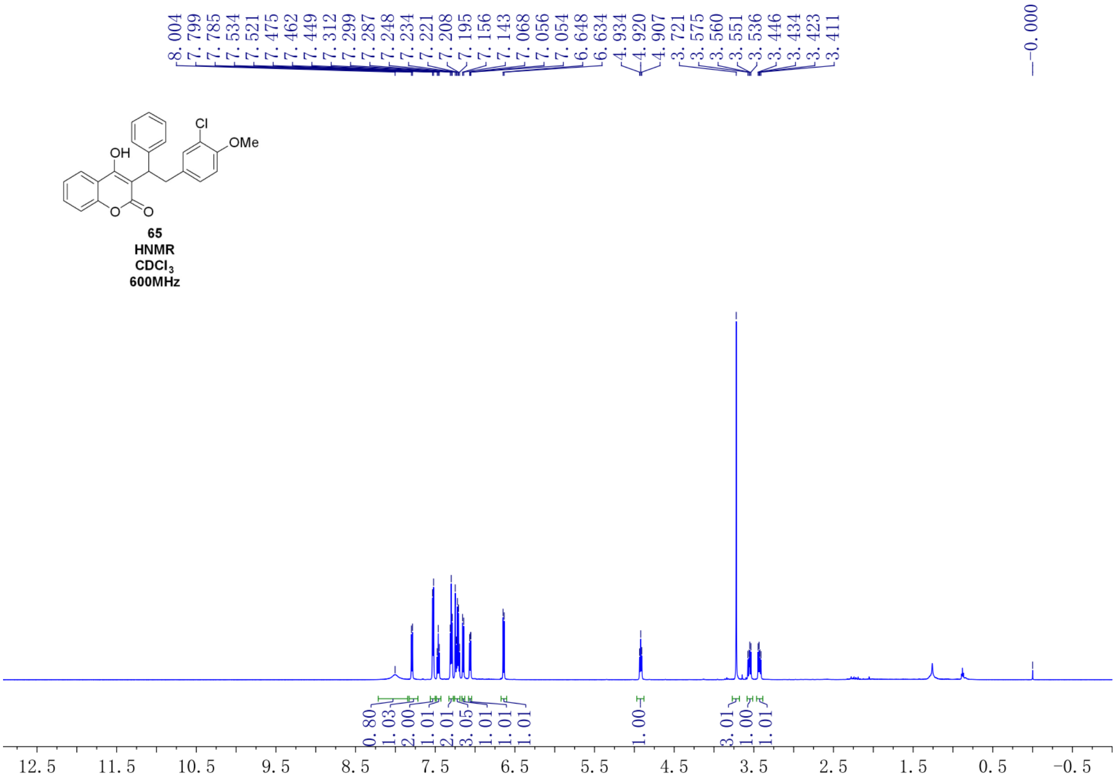
8.004
7.799
7.785
7.534
7.521
7.475
7.462
7.449
7.312
7.299
7.287
7.248
7.234
7.221
7.208
7.195
7.156
7.143
7.068
7.056
7.054
6.648
6.634
4.934
4.920
4.907
3.721
3.575
3.560
3.551
3.536
3.446
3.434
3.423
3.411
0.000
OH
Cl
OMe
O
O
65
HNMR
$CDCl_3$
600MHz
0.80
1.03
2.00
1.01
2.01
3.05
1.01
1.01
1.01
1.00
3.01
1.00
1.01
12.5
11.5
10.5
9.5
8.5
7.5
6.5
5.5
4.5
3.5
2.5
1.5
0.5
-0.5

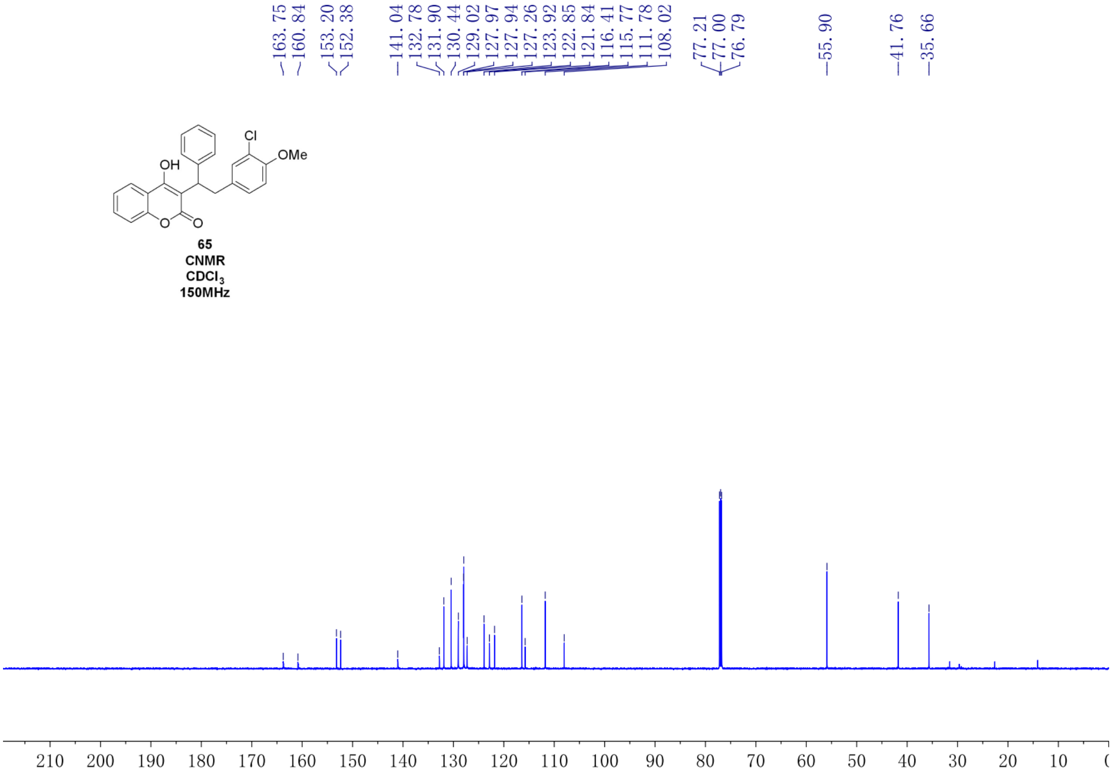
163.75
160.84
153.20
152.38
141.04
132.78
131.90
130.44
129.02
127.97
127.94
127.26
123.92
122.85
121.84
116.41
115.77
111.78
108.02
77.21
77.00
76.79
55.90
41.76
35.66
OH
Cl
OMe
O
O
65
CNMR
$CDCl_3$
150MHz
210
200
190
180
170
160
150
140
130
120
110
100
90
80
70
60
50
40
30
20
10

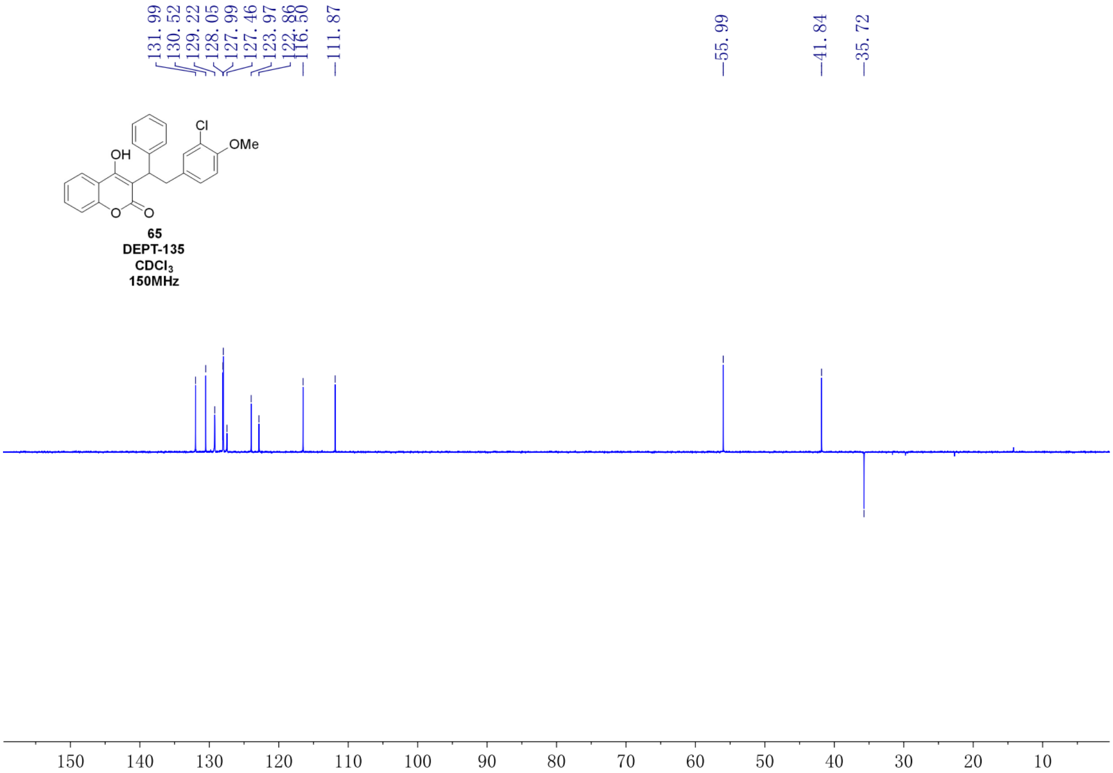
131.99
130.52
129.22
128.05
127.99
127.46
123.97
122.86
116.50
111.87
55.99
41.84
35.72
Cl
OMe
OH
O
O
65
DEPT-135
$CDCl_3$
150MHz
150 140 130 120 110 100 90 80 70 60 50 40 30 20 10

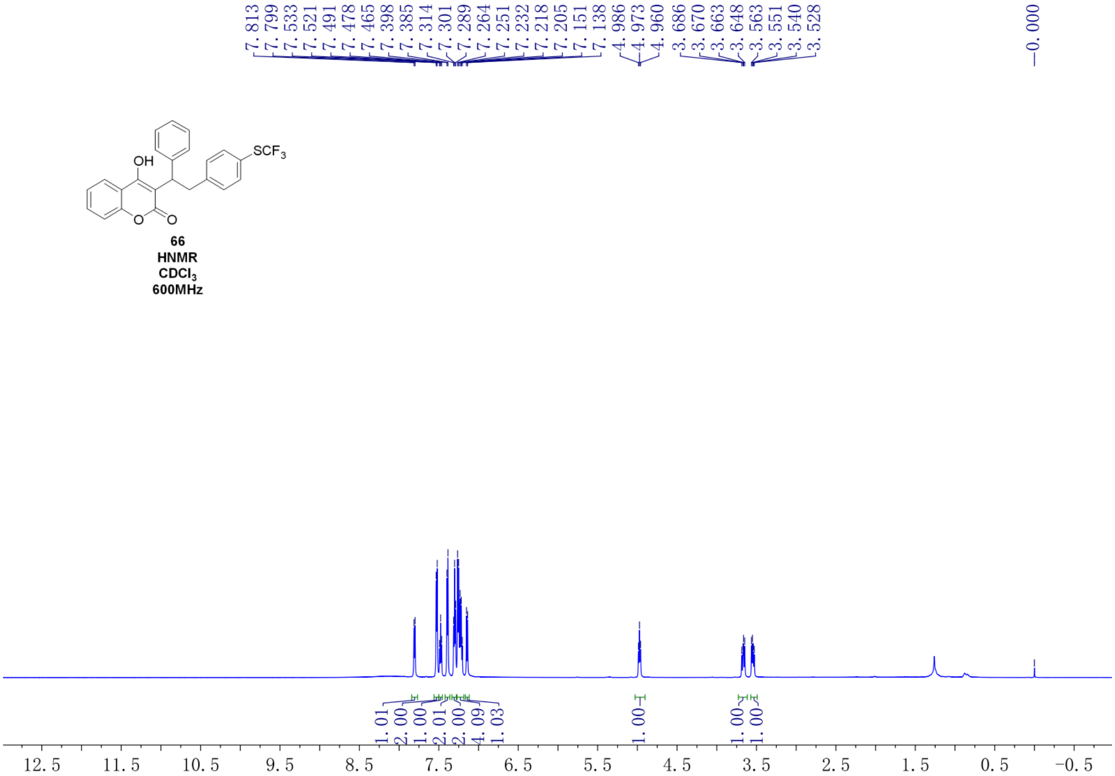
7.813
7.799
7.533
7.521
7.491
7.478
7.465
7.398
7.385
7.314
7.301
7.289
7.264
7.251
7.232
7.218
7.205
7.151
7.138
4.986
4.973
4.960
3.686
3.670
3.663
3.648
3.563
3.551
3.540
3.528
0.000
SCF_3
OH
O
O
66
HNMR
$CDCl_3$
600MHz
1.01
2.00
1.00
2.01
2.00
4.09
1.03
1.00
1.00
1.00
12.5 11.5 10.5 9.5 8.5 7.5 6.5 5.5 4.5 3.5 2.5 1.5 0.5 -0.5

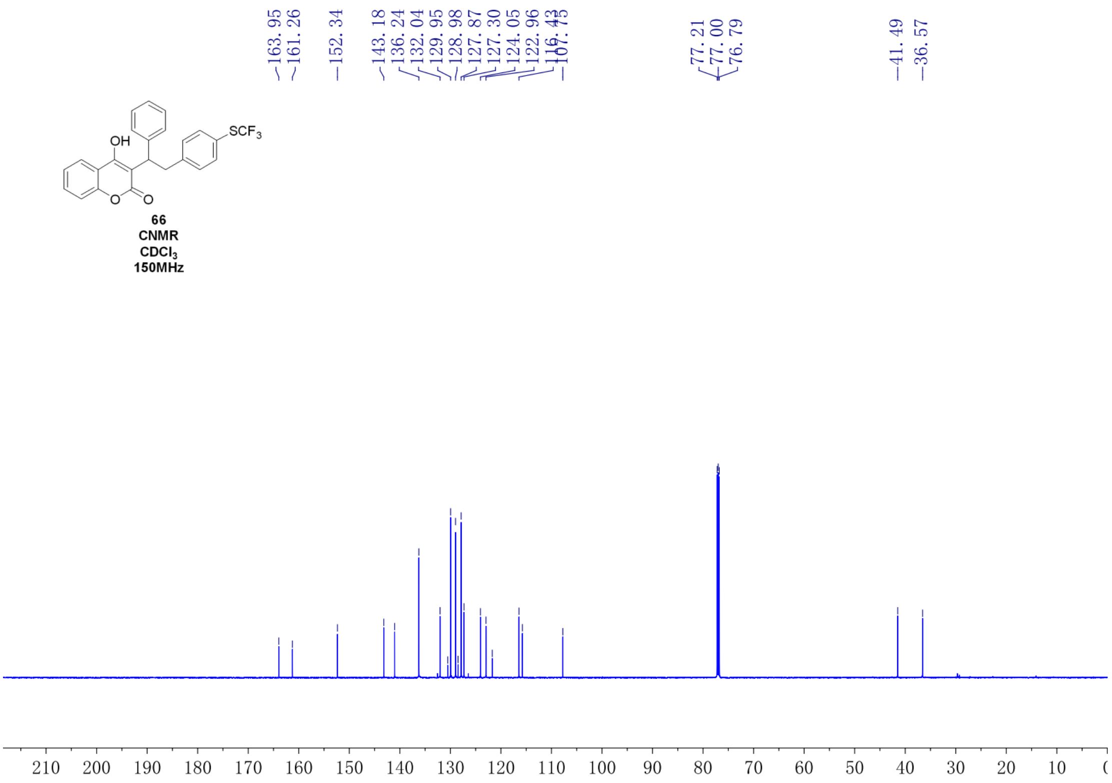
163.95
161.26
152.34
143.18
136.24
132.04
129.95
128.98
127.87
127.30
124.05
122.96
116.43
107.75
77.21
77.00
76.79
41.49
36.57
OH
SCF3
O
O
66
CNMR
CDCl3
150MHz
210 200 190 180 170 160 150 140 130 120 110 100 90 80 70 60 50 40 30 20 10

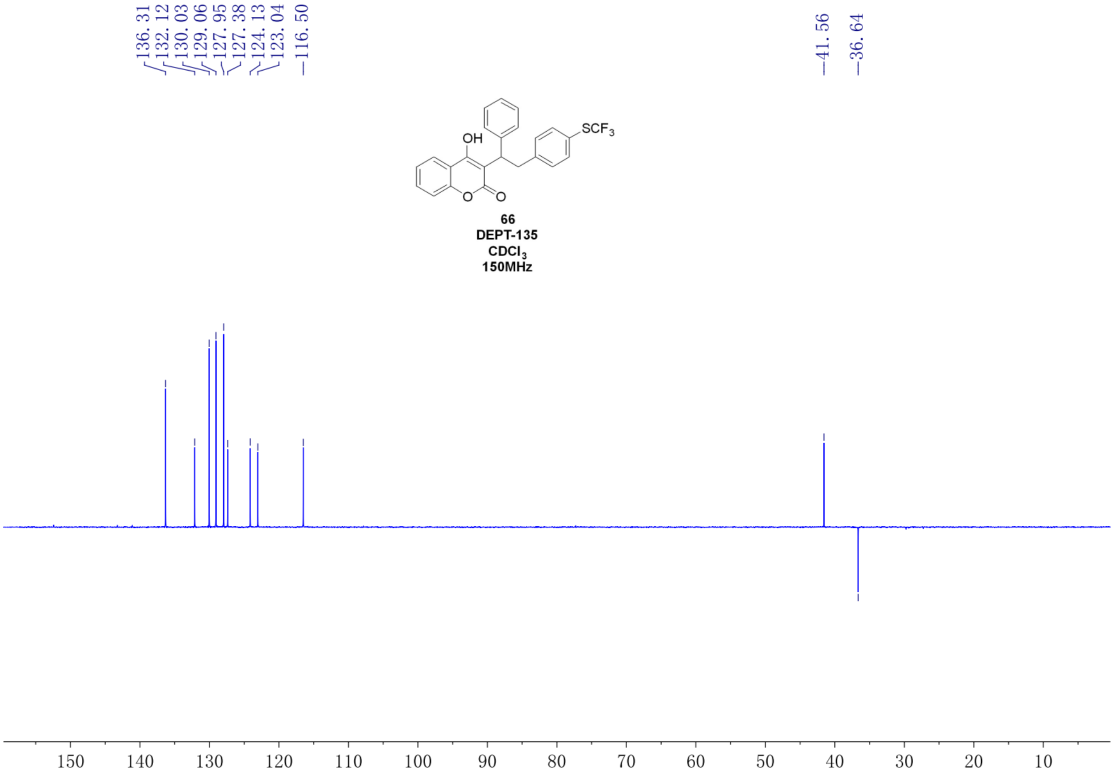
136.31
132.12
130.03
129.06
127.95
127.38
124.13
123.04
116.50
41.56
36.64
OH
SCF3
O
O
66
DEPT-135
CDCl3
150MHz
150 140 130 120 110 100 90 80 70 60 50 40 30 20 10

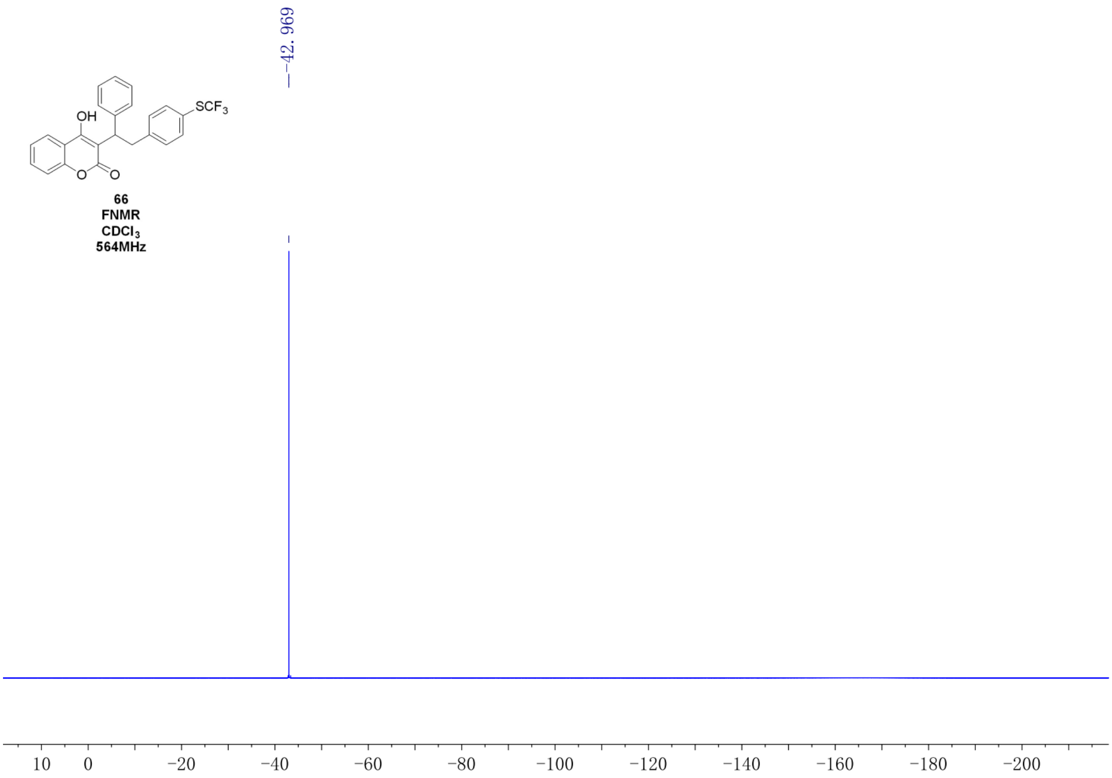
OH
SCF3
O
O
66
FNMR
CDCl3
564MHz
—42.969
10 0 −20 −40 −60 −80 −100 −120 −140 −160 −180 −200

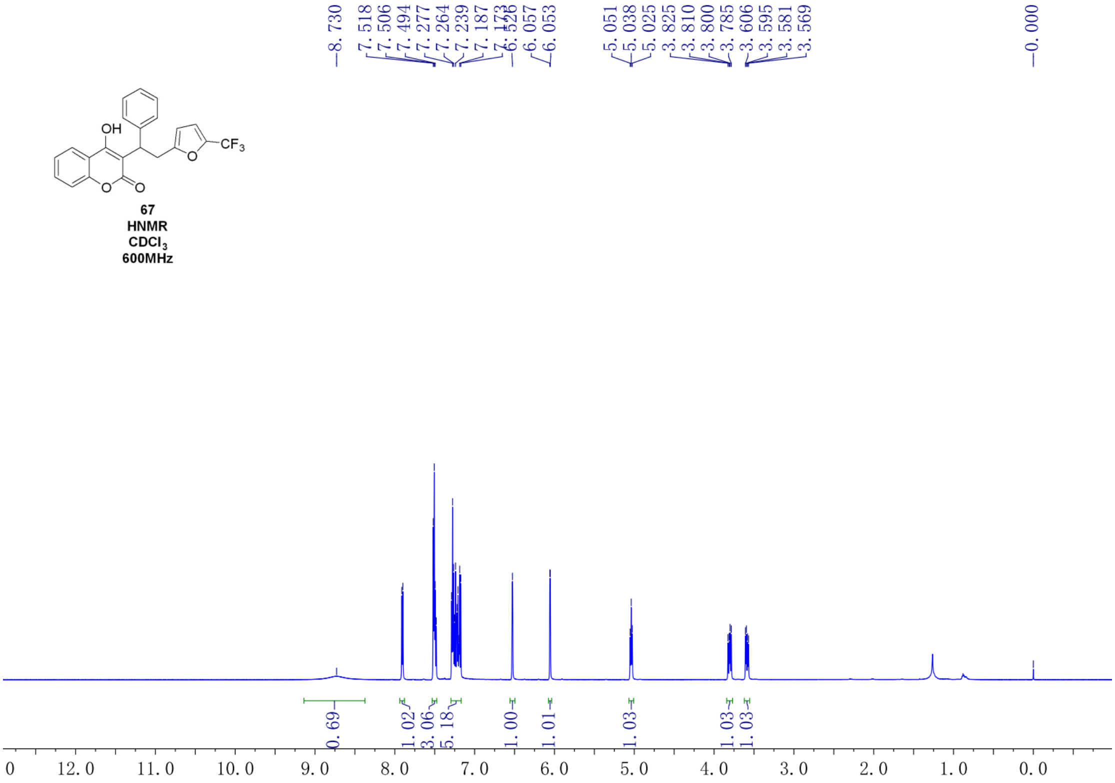
8.730
7.518
7.506
7.494
7.277
7.264
7.239
7.187
7.173
6.526
6.057
6.053
5.051
5.038
5.025
3.825
3.810
3.800
3.785
3.606
3.595
3.581
3.569
0.000
OH
O
CF3
O
O
67
HNMR
CDCl3
600MHz
0.69
1.02
3.06
5.18
1.00
1.01
1.03
1.03
1.03
0 12.0 11.0 10.0 9.0 8.0 7.0 6.0 5.0 4.0 3.0 2.0 1.0 0.0

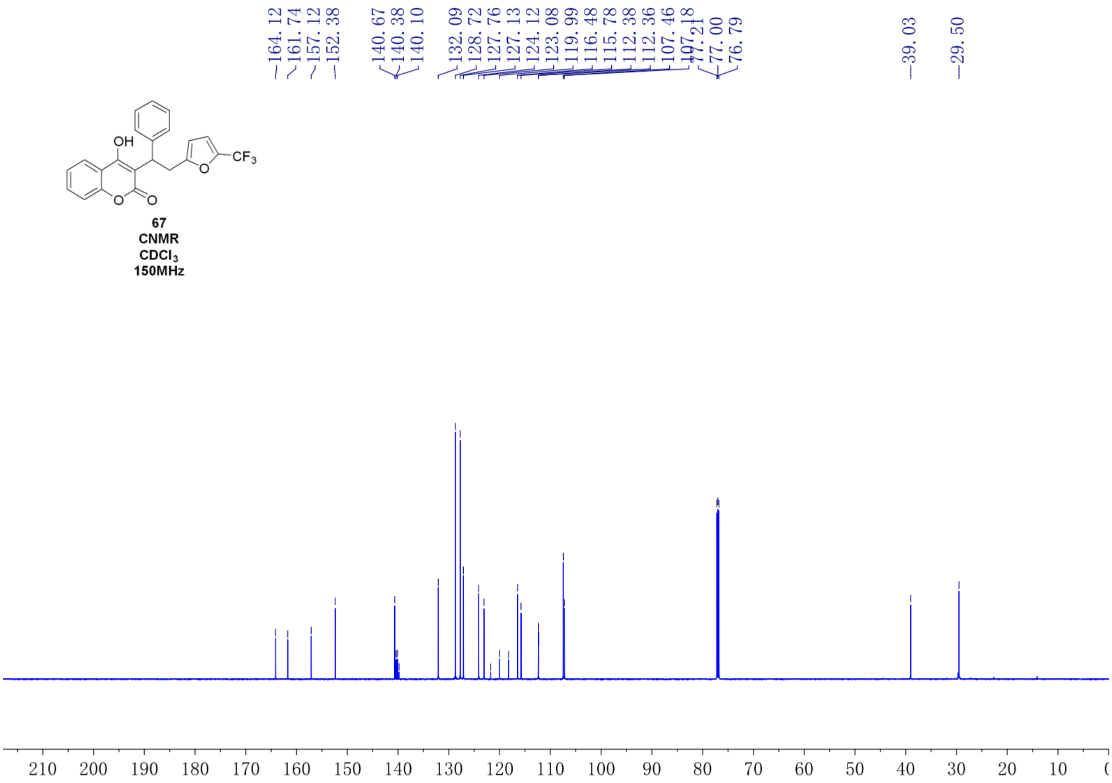
164.12
161.74
157.12
152.38
140.67
140.38
140.10
132.09
128.72
127.76
127.13
124.12
123.08
119.99
116.48
115.78
112.38
112.36
107.46
107.18
77.21
77.00
76.79
39.03
29.50
OH
CF3
O
O
O
67
CNMR
CDCl3
150MHz
210 200 190 180 170 160 150 140 130 120 110 100 90 80 70 60 50 40 30 20 10

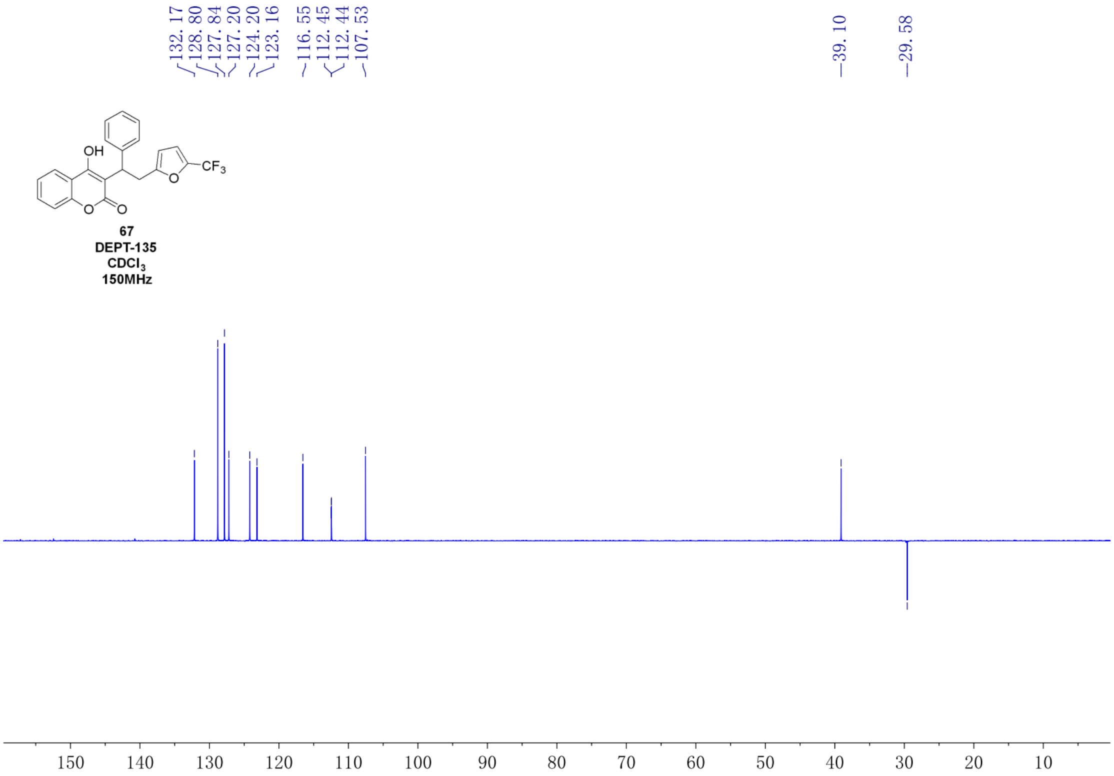
132.17
128.80
127.84
127.20
124.20
123.16
116.55
112.45
112.44
107.53
39.10
29.58
OH
CF3
O
O
O
67
DEPT-135
CDCl3
150MHz
150 140 130 120 110 100 90 80 70 60 50 40 30 20 10

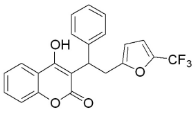

67
FNMR
$CDCl_3$
564MHz

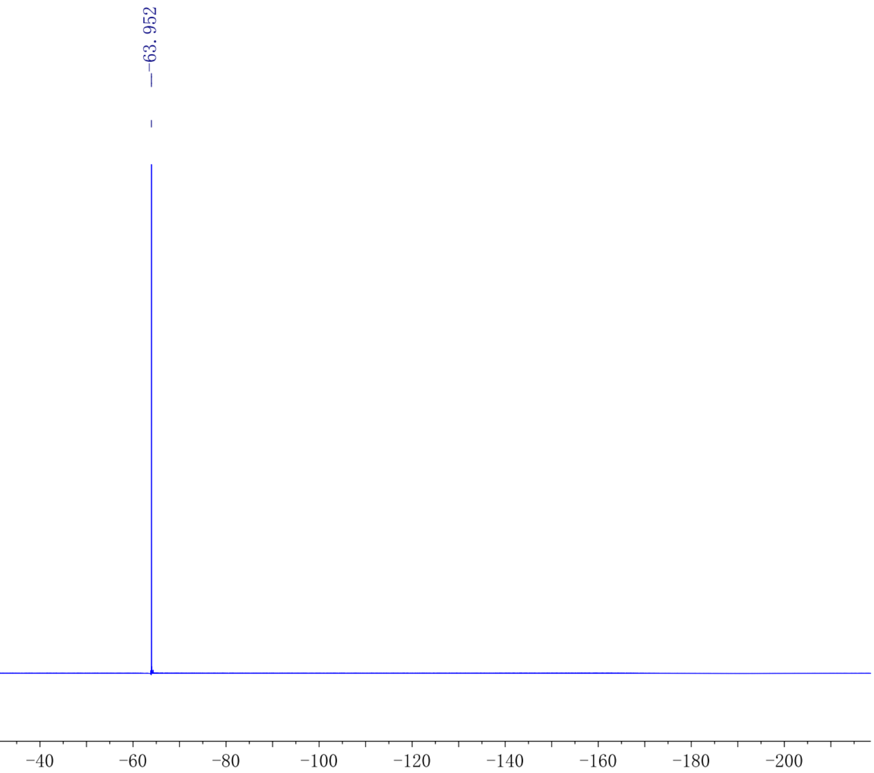

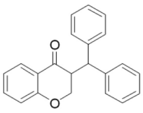

68
HNMR
$CDCl_3$
600MHz

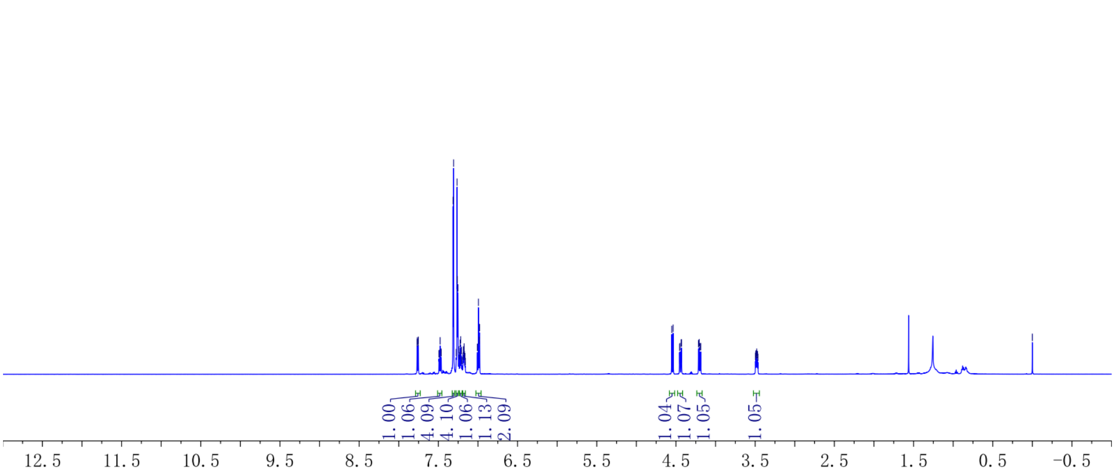

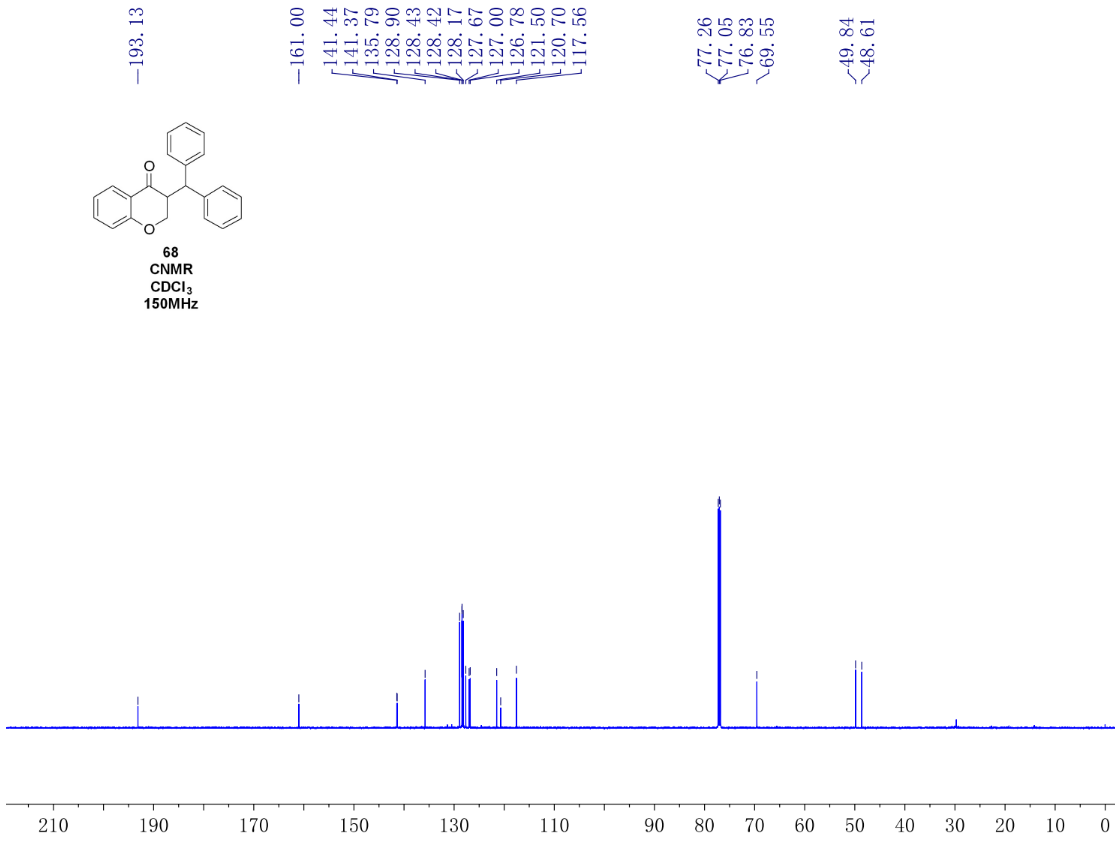
193.13
161.00
141.44
141.37
135.79
128.90
128.43
128.42
128.17
127.67
127.00
126.78
121.50
120.70
117.56
77.26
77.05
76.83
69.55
49.84
48.61
O
O
68
CNMR
$CDCl_3$
150MHz
210 190 170 150 130 110 90 80 70 60 50 40 30 20 10 0

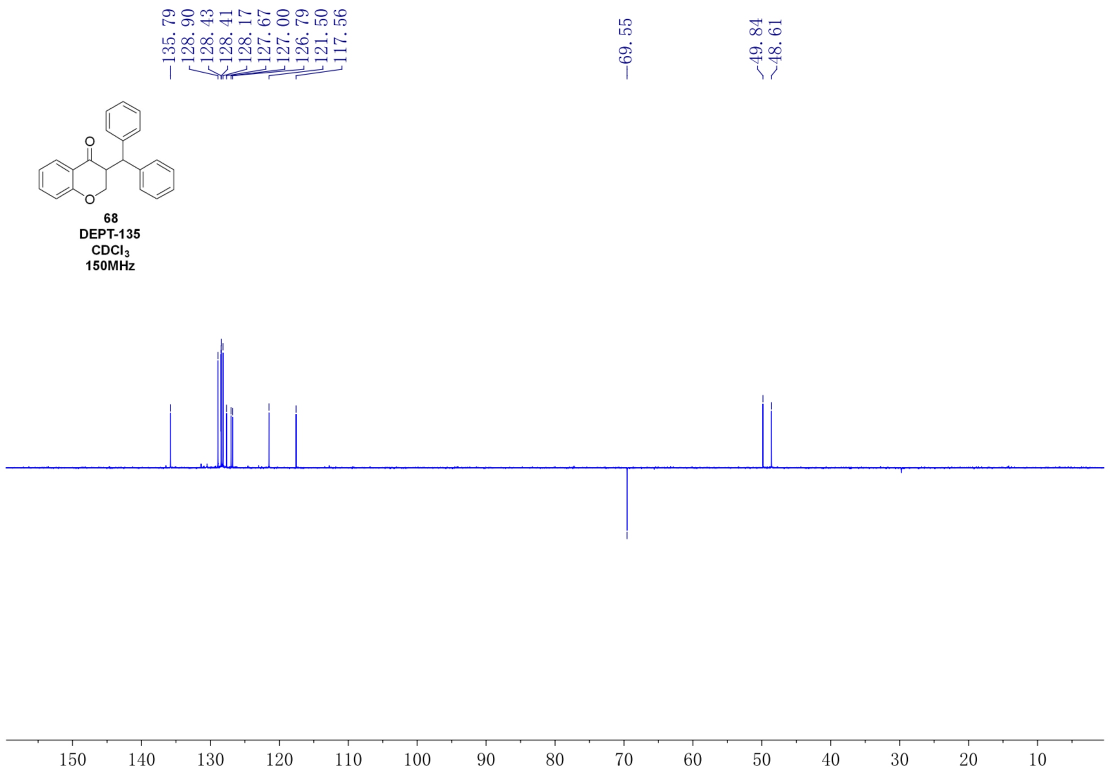
135.79
128.90
128.43
128.41
128.17
127.67
127.00
126.79
121.50
117.56
69.55
49.84
48.61
O
O
68
DEPT-135
$CDCl_3$
150MHz
150 140 130 120 110 100 90 80 70 60 50 40 30 20 10
