# Supplementary material for: Nickel-catalyzed β-arylation and benzylation of 2′-hydroxychalcones to access warfarin analogues
Source: Commun Chem. 2025 Nov 27;8:382. doi: 10.1038/s42004-025-01767-w (PMC12660831; doi:10.1038/s42004-025-01767-w)
Supplement: Supplementary file 3 — Reporting Summary [file 42004_2025_1767_MOESM3_ESM.pdf]

nature portfolio

Corresponding author(s): Cong Ma

Last updated by author(s): Sep 22, 2025

Reporting Summary

Nature Portfolio wishes to improve the reproducibility of the work that we publish. This form provides structure for consistency and transparency in reporting. For further information on Nature Portfolio policies, see our Editorial Policies and the Editorial Policy Checklist.

Statistics

For all statistical analyses, confirm that the following items are present in the figure legend, table legend, main text, or Methods section.

n/a	Confirmed	
☐	☒	The exact sample size (*n*) for each experimental group/condition, given as a discrete number and unit of measurement
☐	☒	A statement on whether measurements were taken from distinct samples or whether the same sample was measured repeatedly
☐	☒	The statistical test(s) used AND whether they are one- or two-sided<br>*Only common tests should be described solely by name; describe more complex techniques in the Methods section.*
☒	☐	A description of all covariates tested
☐	☒	A description of any assumptions or corrections, such as tests of normality and adjustment for multiple comparisons
☐	☒	A full description of the statistical parameters including central tendency (e.g. means) or other basic estimates (e.g. regression coefficient) AND variation (e.g. standard deviation) or associated estimates of uncertainty (e.g. confidence intervals)
☐	☒	For null hypothesis testing, the test statistic (e.g. F, t, r) with confidence intervals, effect sizes, degrees of freedom and P value noted<br>*Give P values as exact values whenever suitable.*
☒	☐	For Bayesian analysis, information on the choice of priors and Markov chain Monte Carlo settings
☒	☐	For hierarchical and complex designs, identification of the appropriate level for tests and full reporting of outcomes
☒	☐	Estimates of effect sizes (e.g. Cohen's *d*, Pearson's *r*), indicating how they were calculated

Our web collection on statistics for biologists contains articles on many of the points above.

Software and code

Policy information about availability of computer code

Data collection	No software was used to collect the data in this study.
Data analysis	No software was used to collect the data in this study.

For manuscripts utilizing custom algorithms or software that are central to the research but not yet described in published literature, software must be made available to editors and reviewers. We strongly encourage code deposition in a community repository (e.g. GitHub). See the Nature Portfolio guidelines for submitting code & software for further information.

Data

Policy information about availability of data

All manuscripts must include a data availability statement. This statement should provide the following information, where applicable:

- Accession codes, unique identifiers, or web links for publicly available datasets
- A description of any restrictions on data availability
- For clinical datasets or third party data, please ensure that the statement adheres to our policy

The authors declare that the data supporting the findings of this study are available within the paper and its Supplementary Information

Research involving human participants, their data, or biological material

Policy information about studies with human participants or human data. See also policy information about sex, gender (identity/presentation), and sexual orientation and race, ethnicity and racism.

Reporting on sex and gender	Not applicable. This study did not involve human participants, their data, or biological material.
Reporting on race, ethnicity, or other socially relevant groupings	Not applicable. This study did not involve human participants, their data, or biological material.
Population characteristics	Not applicable. This study did not involve human participants, their data, or biological material.
Recruitment	Not applicable. This study did not involve human participants, their data, or biological material.
Ethics oversight	Not applicable. This study did not involve human participants, their data, or biological material.

Note that full information on the approval of the study protocol must also be provided in the manuscript.

Field-specific reporting

Please select the one below that is the best fit for your research. If you are not sure, read the appropriate sections before making your selection.

☒ Life sciences ☐ Behavioural & social sciences ☐ Ecological, evolutionary & environmental sciences

For a reference copy of the document with all sections, see nature.com/documents/nr-reporting-summary-flat.pdf

Life sciences study design

All studies must disclose on these points even when the disclosure is negative.

Sample size	Sample size in our study was not predetermined by statistical calculation. Instead, the sample size (n = 3 per test compound) was chosen based on preliminary experiments, which demonstrated that the test compounds exhibited in vivo anticoagulant effects. Given the relatively large number of compounds (11 in total) and in accordance with the 3R principles of animal ethics (Replacement, Reduction, and Refinement), we aimed to minimize animal use while still enabling the detection of meaningful differences in this initial screening study. This approach allowed us to balance scientific objectives with ethical considerations.
Data exclusions	No animals or data were excluded from the analyses. All collected data were included in the reported results.
Replication	All experiments were conducted using independent biological replicates (n = 3 mice per test compound), with each animal serving as an independent data point. The experimental procedures, including compound administration, blood sampling, and measurement of bleeding time and blood loss, were standardized and performed consistently across all groups to minimize technical variability. As this study represents an initial in vivo screening of multiple compounds, no additional independent replication experiments were performed. However, the reproducibility of the findings within each group was supported by the consistency of results among the biological replicates. All data points are reported in the figures, and no outliers were excluded.
Randomization	Mice were randomly allocated to vehicle control, warfarin control, or test compound groups to minimize allocation bias.
Blinding	Investigators were blinded to group allocation during data collection and analysis.

Reporting for specific materials, systems and methods

We require information from authors about some types of materials, experimental systems and methods used in many studies. Here, indicate whether each material, system or method listed is relevant to your study. If you are not sure if a list item applies to your research, read the appropriate section before selecting a response.

Materials & experimental systems

n/a	Involved in the study
☒	☐ Antibodies
☒	☐ Eukaryotic cell lines
☒	☐ Palaeontology and archaeology
☐	☒ Animals and other organisms
☒	☐ Clinical data
☒	☐ Dual use research of concern
☒	☐ Plants

Methods

n/a	Involved in the study
☒	☐ ChIP-seq
☒	☐ Flow cytometry
☒	☐ MRI-based neuroimaging

Animals and other research organisms

Policy information about studies involving animals; ARRIVE guidelines recommended for reporting animal research, and Sex and Gender in Research

Laboratory animals	Male ICR (CD-1) mice (6-8 weeks) were used in our study.
Wild animals	This study did not involve wild animals.
Reporting on sex	All experiments in this study were conducted exclusively in male ICR mice. This consideration to use only one sex was made to minimize potential variability due to sex differences and to allow for a more controlled initial screening of the anticoagulant effects of the test compounds. Sex was therefore considered in the study design, and only male mice (6–8 weeks old) were included. No data were collected from female animals, and thus no sex-based analyses were performed.
Field-collected samples	This study did not involve samples or animals collected from the field.
Ethics oversight	All procedures related to animal welfare in this study were conducted in accordance with the animal welfare policies and guidelines of the Drug Safety Testing Center (DSC), Hong Kong. The study protocol was reviewed and approved by the DSC Institutional Animal Care and Use Committee (IACUC) (Ethics code: 24-019).

Note that full information on the approval of the study protocol must also be provided in the manuscript.

Plants

Seed stocks	Not applicable
Novel plant genotypes	Not applicable
Authentication	Not applicable
